# Regenerable Heterogeneous Palladium Single-Atom Catalysts for Direct C–H Arylation

**DOI:** 10.1021/jacs.6c11697

**Published:** 2026-10-16

**Authors:** Valentas Olikauskas, Marc-Eduard Usteri, Aram Bugaev, Milivoj Plodinec, Sharon Mitchell, Jerome Waser, Javier Pérez-Ramírez

**Affiliations:** † Laboratory of Catalysis and Organic Synthesis, Institute of Chemical Sciences and Engineering, 27218 École Polytechnique Fédérale de Lausanne, Avenue F. A. Forel 2, Lausanne 1015, Switzerland; ‡ Institute of Chemical and Bioengineering, Department of Chemistry and Applied Biosciences, 27219 ETH Zürich, Vladimir-Prelog-Weg 1, Zürich 8093, Switzerland; § 28498 Scherrer Institute, Forschungsstrasse 111, Villigen 5232, Switzerland; ∥ Scientific Center for Optical and Electron Microscopy, ETH Zürich, Otto-Stern-Weg 3, Zürich 8093, Switzerland; ⊥ Laboratory of Inorganic Chemistry, Department of Chemistry and Applied Biosciences, ETH Zürich, Vladimir-Prelog-Weg 2, Zürich 8093, Switzerland; # NCCR Catalysis, Zürich 8093, Switzerland

## Abstract

Single-atom catalysts (SACs) offer tunable, recoverable heterogeneous alternatives to homogeneous metal complexes in handle-based cross-coupling reactions. However, their application to direct C–H bond functionalization remains unexplored beyond photochemical methods, as the elevated temperature and demanding reaction conditions place stringent requirements on catalyst stability. Here, we demonstrate a Pd single-atom-catalyzed C–H arylation of indoles, extending heterogeneous SACs to thermal C–H functionalization and achieving cumulative turnover numbers above 200 across five cycles. Among diverse oxygen- and nitrogen-coordinated SACs, nitrogen-coordinated sites in Pd@C_3_N_4_ uniquely combined metal retention with high activity after phosphine activation. Recycling studies coupled with in situ X-ray absorption spectroscopy reveal Pd–I bond formation as the dominant catalyst deactivation pathway. Guided by this insight, postreaction treatment in aqueous ammonia overcomes iodide poisoning, enabling catalytic activity over multiple cycles. These findings establish design principles for balancing activity, stability, and regenerability in Pd SACs for demanding C–H functionalizations, advancing their potential for greener, more step-efficient chemical synthesis.

## Graphical abstract

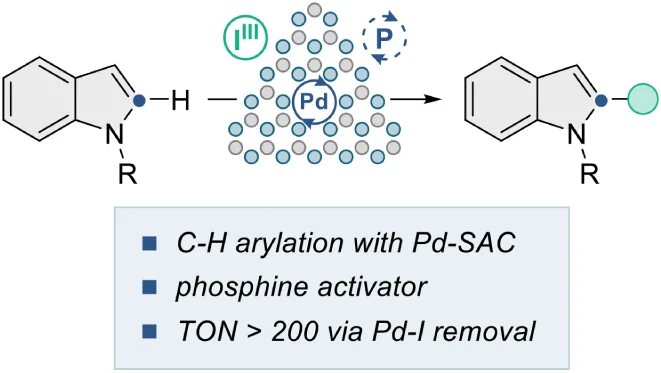

## 1 Introduction

Palladium-catalyzed cross-coupling reactions are a powerful methodology for assembling C–C bonds and linking functionalized building blocks.^1–3^ A vast portion of palladium-catalyzed cross-couplings relies on synthetic handles, such as (pseudo)­halides, in combination with organometallic species, such as organozinc reagents or boronic acids. While the strategy displays excellent regioselectivity, introducing the handle inherently diminishes the efficiency of the overall process. The prefunctionalization requires extra synthetic steps, and some ubiquitous handles, such as boronic acids, are known to be unstable to purification and storage.^4–6^ These limitations can be overcome by direct modification of prevalent C–H bonds (Figure 1a).^7–9^ The area of homogeneous transition-metal-catalyzed C–H functionalization emerged in the late 60s and grew rapidly in academic research, but there was little progress on process industrialization.^10–13^ Many existing catalytic C–H functionalizations require high precious metal loadings, which are difficult to recover after the transformation, leading to economic, environmental, and health-related concerns.

**Figure 1:**
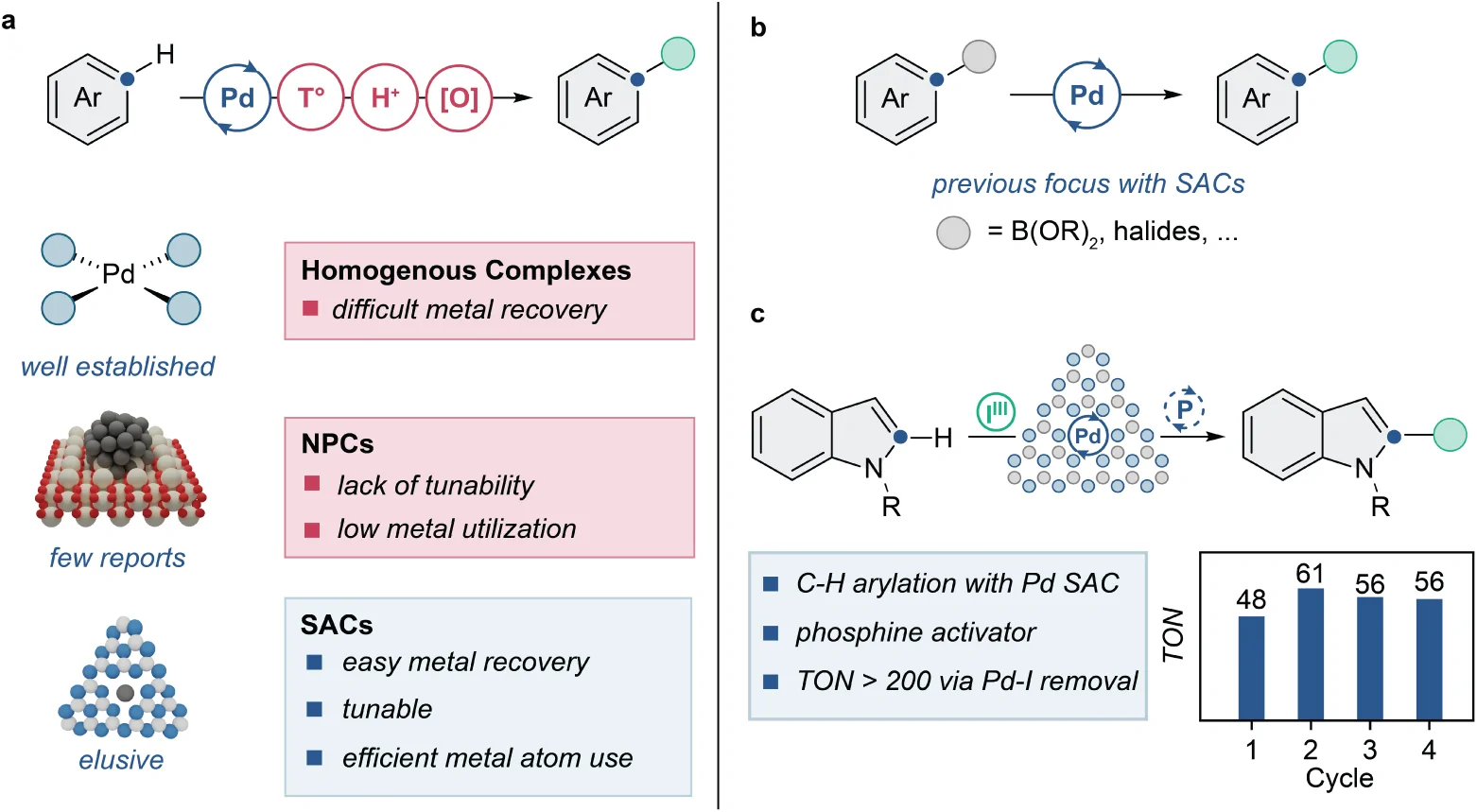
(a) Overview of the state of Pd catalysts developed for C–H functionalizations. (b) Previous focus of C–C bond forming reactions with SACs. (**c**) Pd@C_3_N_4_-catalyzed C–H arylation of indoles developed in the current study.

Palladium is the most often used metal catalyst, and regulatory restrictions limit its amount in APIs to below 10 ppm,^14^ while recent life cycle assessments of cross-couplings have shown that most of the impact is associated with the metal.^15^ Additionally, elevated temperatures, strong oxidative, acidic, or basic conditions are often needed to achieve efficient catalysis. Attempts to address these issues by supported metal nanoparticle catalysts (NPCs) or immobilized metal complexes often suffer from catalyst deactivation, either via metal leaching, aggregation, or other pathways.^16^ At the same time, NPCs lack tunability and display low metal utilization, as only surface atoms engage in transformations.

Recently, single-atom catalysts (SACs) have emerged as a new class of heterogeneous metal catalysts. SACs possess greater atomic precision that can be tuned by altering metal–support interactions and retain important properties of solid catalysts, such as easy separation from the reaction mixture and recyclability.^17–19^ Despite multiple potential advantages of these materials, previous reports in the field of organic reaction development with SACs mainly focused on classical, handle-based, cross-coupling reactions such as Suzuki-Miyaura, Buchwald-Hartwig, and Sonogashira-Hagihara (Figure 1b).^20,21^ Direct C–H bond transformations, however, remain unexplored beyond photochemical methods,^22–24^ potentially because the harsh reaction conditions often required can trigger catalyst degradation. Additionally, the conditions required to enable direct C–H functionalization may differ from those developed for handle-based cross-couplings. If realized, Pd single-atom-catalyzed C–H functionalizations could streamline the application of this synthetic strategy in process chemistry, where it is still sparsely applied.

In this study, we demonstrate the Pd single-atom-catalyzed C–H arylation of indoles (Figure 1c), bridging the gap between Pd-SACs and C–H functionalization. Comparative studies reveal an activity-stability trade-off across supports, with Pd@C_3_N_4_ (C_3_N_4_ = carbon nitride) exhibiting no detectable metal loss but only minor activity. Enhanced performance is unlocked by introducing substoichiometric amounts of phosphine, which, in contrast to previous SAC studies, does not act as a ligand but instead initiates catalytic turnover, most likely through a proposed acid-mediated pathway. However, the catalyst recovered after the reaction displays very low reactivity and cannot be reused. Catalytic evaluation combined with spectroscopic analyses reveals iodide coordination to Pd centers as the dominant deactivation pathway, previously not observed for single-atom catalysts in cross-coupling reactions. Guided by these insights, we develop a post-reaction regeneration strategy based on aqueous ammonia treatment, restoring activity over multiple cycles. These findings establish design principles to balance activity, metal retention, and regenerability, providing mechanistic foundations for extending SACs to direct thermal C–H functionalization reactions.

## 2 Results and Discussion

### 2.1 Identifying Active Palladium Species

We focused on the Pd-catalyzed arylation of indoles (Figure 2a) as a target transformation. This reaction has played an important role in the development of C–H functionalizations due to the preponderance of indoles in medicinal chemistry, and the relatively harsh reaction conditions reported (heating in some cases, acidic solvent, strong oxidants) are representative of the field.^25^ Furthermore, this transformation does not require directing groups and relies on the catalytic system to achieve regioselectivity. To start, we designed a comparative study based on the most commonly studied support materials for SACs: Al_2_O_3_, TiO_2_, CeO_2_, activated carbon (AC), thermally exfoliated carbon nitride (C_3_N_4_), and nitrogen-doped carbon (NC). Considering the different carrier properties, Pd was introduced via wet impregnation followed by thermal activation. Wet impregnation has the advantage that metal atoms reach an equilibrium between the solvated phase and available binding sites.^26–28^ Thus, the synthesis is compatible with different materials, scalable, and can readily be extended to other supports. The catalysts were thermally activated at 200 °C in static air, yielding a platform of materials based on metal oxide or carbon supports (denoted Pd@carrier).

**Figure 2:**
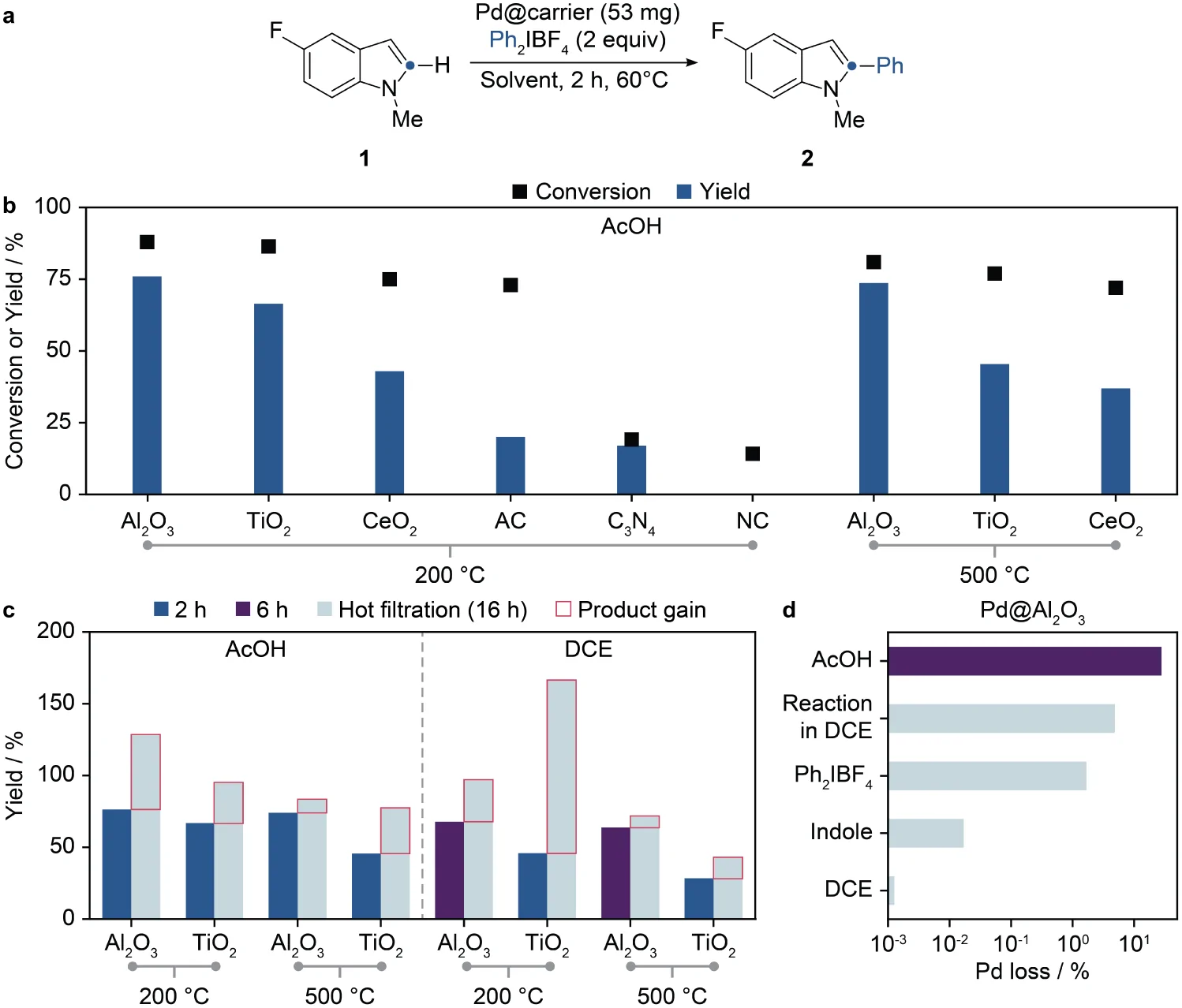
(a) Reaction scheme of the Pd-catalyzed C–H arylation of 1-methyl-5-fluoroindole **1** to 1-methyl-2-phenyl-5-fluoroindole **2**. (b) Comparative catalyst performance of the as-prepared catalysts. Conditions: 0.1 mmol indole, 2 equiv Ph_2_IBF_4_, 53 mg catalyst (see Table S1 for Pd amount in mol%), 0.8 mL solvent, 2 h, 60 °C (see Table S2 for a list of examined catalysts and solvents and Note S3 for a comment on solvent effect). (c) Hot filtration tests of selected catalysts in AcOH and DCE. Time before and after filtration as indicated. (d) Pd loss determined by ICP-MS for Pd@Al_2_O_3_ caused by different reaction components in DCE (light blue) and by AcOH (purple).

The resulting catalysts were evaluated in the C–H arylation of 1-methyl-5-fluoroindole (**1**), enabling reaction analysis by ^19^F NMR, using diphenyl iodonium tetrafluoroborate (Ph_2_IBF_4_) as an arylating agent to form product **2**, following conditions previously reported for a homogeneous palladium catalyst (Figure 2a).^25^ Interestingly, oxide-supported catalysts showed higher initial conversions, whereas nitrogen-binding environments (Pd@C_3_N_4_ and Pd@NC) exhibited moderate or no activity, respectively (Figure 2b).

To rationalize these differences, selected catalysts were analyzed by scanning transmission electron microscopy (STEM, Figures S1, S2) and X-ray absorption spectroscopy (XAS, Figure S3), showing the predominance of atomically dispersed Pd. Coexistence of clusters or particles cannot be fully excluded, as XAS can be insensitive to small populations of aggregates, particularly in polydisperse or amorphous systems.^29,30^ Nanoparticle contents up to 15% may therefore remain undetected. Similarly, machine-learning-driven atom detection^31^ across multiple micrographs indicates a 10–16% upper bound for multimer formation (Figure S2), although no larger aggregates are visible. X-ray absorption near-edge structure (XANES) indicates the prevalence of Pd­(II) in the catalyst. Coordination numbers and bond distances determined by extended X-ray absorption fine structure (EXAFS) fitting are consistent with Pd sites typical of single-atom catalysts (Tables S3 and S4).^32–34^ As such, activity differences between the catalysts could not be connected to differences in formal oxidation state or speciation and have to originate from differences in active site structure and metal binding environment, or accessibility that cannot be fully elucidated spectroscopically (Note S1).

Subsequently, we examined whether the catalytically active species remain attached to the surface or if the solid merely acts as a reservoir for Pd that releases the catalytically active homogeneous species over time.^35^ After hot filtration of the reaction mixture, no significant increase in yield was observed, suggesting a negligible contribution from catalytically active leached (CAL) species (Table S5, Note S2). However, inductively coupled plasma-mass spectrometry (ICP-MS) analysis of the filtrate revealed that the catalysts lost 1.3–30% of their Pd content when stirred in AcOH solvent (Table S6), indicating a possible discrepancy between reaction filtrate analysis and hot filtration results. Considering the precedence in homogeneous catalysis, this lack of reactivity was surprising, and we tentatively attributed it to loss or decomposition of the iodonium salt during filtration due to its reduced solubility. As a countermeasure, we developed a modified hot filtration protocol where fresh reagents were added after the filtration step (Figure S4). Under these conditions, the increase in yield by the leached Pd was captured and showed the formation of CAL species (Figure 2c, Note S2), highlighting that hot filtration control experiments need to be performed with care to avoid result misinterpretation. This modified methodology avoids kinetic constraints associated with conventional hot filtration, enables comparison across catalysts with different activities, and mitigates artifacts arising from reagent loss during filtration.

Next, our efforts focused on understanding the origin of Pd leaching with the goal of identifying active SACs. First, we discovered that Pd leaching was already initiated for most catalysts just by the AcOH solvent (Table S6), likely due to the limited stability of solid-bound Pd­(II) in acidic media.^36–38^ Replacing AcOH with less coordinating solvents reduced Pd loss, with ICP-MS analysis confirming negligible Pd leaching in pure DCE (Table S6). Nevertheless, oxide-supported catalysts continued to generate CAL Pd species under the reaction conditions even in DCE, as evidenced through modified hot filtration (Figure 2c, Table S7). Further analysis identified the aryliodonium salt as a main contributor to Pd mobilization and aggregation (Figure 2d, Table S8, Note S4). This may indicate that oxidation of Pd­(II) to Pd­(IV) leads to leaching with most catalyst systems, raising concern that this type of C–H functionalization cannot be achieved with oxygen-coordinated Pd single atoms.

### 2.2 In situ Activation of *N*-Coordinated Palladium Sites

Supports providing nitrogen anchoring sites (C_3_N_4_, NC) showed only minor activity, but they seemed to remain resistant to leaching and sintering in DCE, as evidenced by XAS spectra of the used catalysts (Figure S3) and ICP-MS analysis of the filtrate (Table S6). We hypothesized that in situ activation could unlock reactivity, inspired by prior reports using ligands and coordinating bases to alter and direct selectivity,^32,39,40^ activity,^32,33,39^ or stability.^41^ We therefore decided to attempt in situ activation by introduction of phosphines and found that PPh_2_Cy (**P1**) and PPh_3_ (**P2**) could efficiently activate Pd@C_3_N_4_, furnishing **2** in 73% and 65% ^19^F NMR yields, respectively (Figure 3a, entries 1 and 2, see Tables S10–S12 and Note S5 for details on reaction optimization) with the highest C2/C3 selectivity (8:1) using only 1.25 mol% of Pd@C_3_N_4_. The catalytic system showed superior activity under ambient atmosphere compared to inert conditions (entry 3), rendering it highly practical. Pd@C_3_N_4_ prepared from (NH_3_)_4_Pd­(NO_3_)_2_ (73%, entry 1) gave higher yields than Pd­(NO_3_)_2_-derived analogs (55%, entry 4), suggesting that amino ligands were important for high yields, while other ligands on the Pd precursor showed lower performance (33 – 55% yields, Table S13). Notably, phosphines could not efficiently activate Pd@NC (entry 5), the other nitrogen-coordinated Pd-SAC. Control experiments confirmed that both phosphine (entry 6) and Pd@C_3_N_4_ (entry 7) were required, while ICP-MS (Table S14) of the filtrate, hot filtration (Figure 3b, Table S15), and postreaction characterization indicated negligible Pd loss (ICP-OES, Table S1) with the absence of CAL species and no Pd aggregation as seen by high-angle annular dark-field scanning transmission electron microscopy (HAADF-STEM, Figure 3c). Direct comparison of the homogeneous and heterogeneous catalyst systems is complicated by differences in reaction conditions and substrate scope. Nevertheless, for substrates examined in both studies (e.g., product **10**), the average turnover frequency is of the same order of magnitude (Table S16), while the turnover number is higher for the heterogeneous system (58 vs 15). Under our optimized conditions, homogeneous Pd­(OAc)_2_ furnished only minimal amounts (<5%) of arylated product **2** even with a higher catalyst loading of 5 mol%.

**Figure 3:**
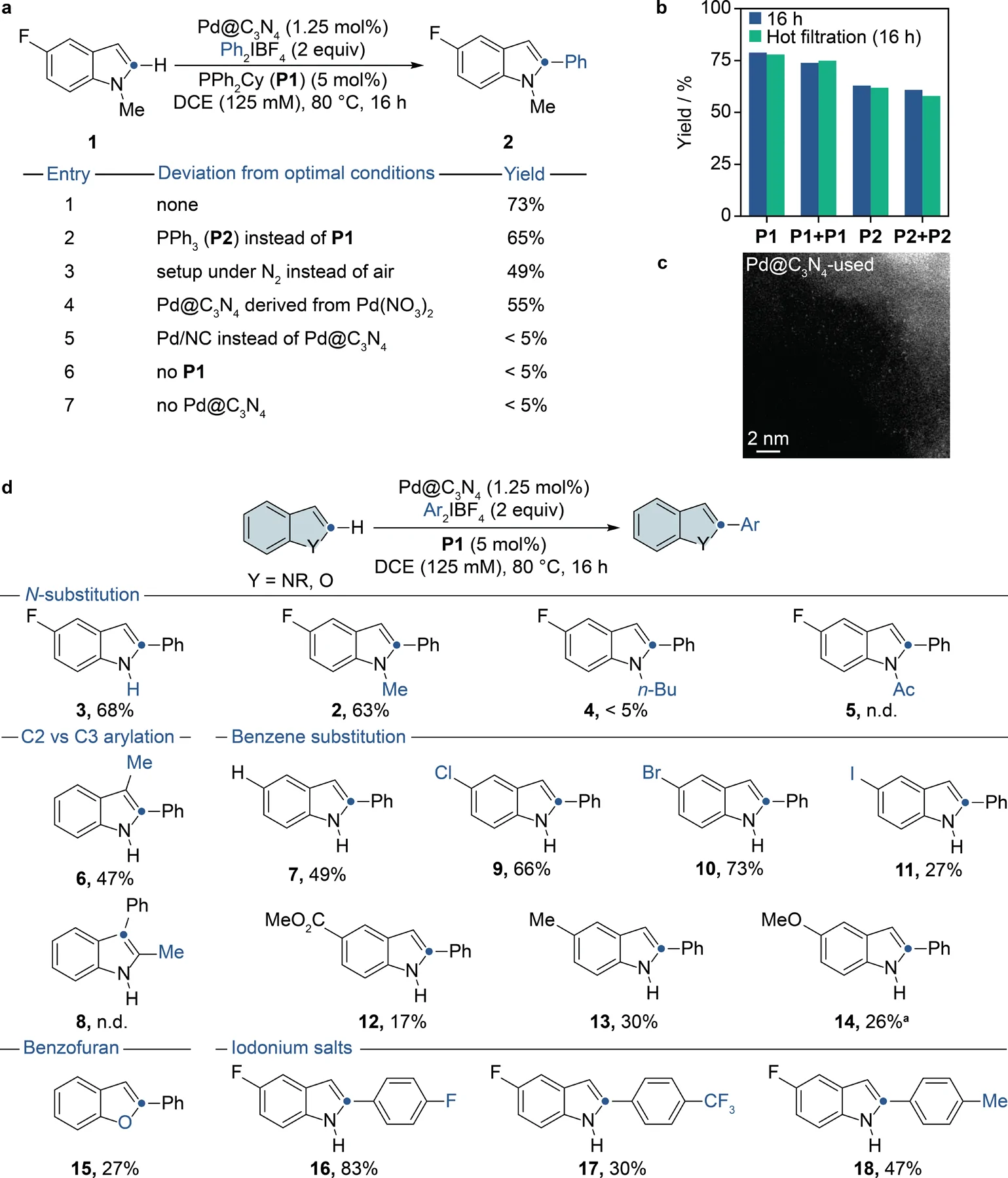
(a) Reaction optimization of the phosphine-promoted Pd-catalyzed C–H arylation of **1** to **2**. (b) Hot filtration tests using Pd@C_3_N_4_ with **P1** or **P2**. Adding more ligand after filtration (**P1**+**P1**) did not alter the hot filtration outcome. Conditions: 0.1 mmol **1**, 2 equiv Ph_2_IBF_4_, 26 mg catalyst (1.25 mol%), DCE (125 mM), 16 h, 80 °C. (c) HAADF-STEM image of used Pd@C_3_N_4_. (d) Influence of the structure of substrate and aryliodonium salt on the reaction efficiency. Conditions: 0.2 mmol heteroarene, 2 equiv Ar_2_IBF_4_, 53 mg catalyst (1.25 mol%), DCE (125 mM), 16 h, 80 °C. ^a^ 60 °C instead of 80 °C. Isolated yields are reported.

With the phosphine-activated catalytic system in hand, we investigated the substrate scope on a 0.2 mmol scale with the aim of understanding its fundamental characteristics. We began by assessing the substitution on the nitrogen of the indole. Increasing bulk on the N1 position resulted in gradually lower isolated yields (Figure 3d, *N*–H, **3**, 68%; *N*–Me, **2**, 63%) with negligible arylation product observed with *N*–*n*-Bu (**4**). These observations highlight a strong sensitivity to steric factors, suggesting steric hindrance at the active site of Pd@C_3_N_4_. An electron-withdrawing acetyl substituent resulted in no conversion to arylated product **5**, consistent with the electrophilic palladation mechanism proposed for similar homogeneous systems.^42^ *N*–Me substituted indole **2** was scaled up from 0.2 to 1 mmol scale with a slight increase in yield (70%). The tolerance of *N*-unsubstituted indoles is noteworthy, as it avoids the need for protecting groups. Hence, the remaining scope was investigated with *N*–H indoles to exploit this potential methodological advantage.

Despite the high sensitivity toward steric effects, a methyl substituent at the C3 position did not influence reactivity significantly (**6**, 47% compared to 49% for **7** with 3–H substitution). With methyl substitution at C2, C3-arylated product **8** was not obtained, although C3-arylation was observed as a side reaction during the optimization of the *N*–Me system and substrate scope exploration (3:1 to >20:1 C2/C3 selectivity range in crude mixtures; see Supporting Information for more details). Intermolecular competition experiments with the C2-deuterated indole for the synthesis of **3** gave an inverse kinetic isotope effect (KIE) of *k*_H_/*k*_D_ = 0.85, arguing against direct concerted metalation–deprotonation (CMD) as the kinetically relevant C–H activation pathway and remaining consistent with electrophilic palladation. Whether the initial palladation proceeds at the C3 or C2 position could not be distinguished by KIE (see Note S9, Table S17). However, as no C3-arylated product was observed for C2–Me substrate, electrophilic palladation at the C2 position appears more plausible.

Catalytic activity depended strongly on substitution of the benzene ring. Aromatic chloride (**9**, 66% yield) and bromide (**10**, 73% yield) substituents were well tolerated, showing the orthogonality of our transformation to classical cross-couplings. However, the presence of an aromatic iodide (**11**) resulted in a diminished yield of 27% with a significant recovery of the starting material, indicating that oxidative addition into the C–I bond might be a competing catalyst deactivation pathway (Section 2.5).

Both electron-withdrawing (5–CO_2_Me, **12**, 17%) and electron-donating substituents (5–Me, **13**, 30%; 5–OMe, **14**, 26%) gave lower yields compared to the halides. Electron-withdrawing substituents can impede the electrophilic palladation step, while free *N*–H indoles bearing an electron-donating group could undergo oxidative decomposition. The latter was supported by a higher ^1^H NMR yield obtained at 60 °C (39%) instead of 80 °C (31%) for **14**. Coordinating functionalities, such as free aliphatic alcohol (Figure S5, compound S1) and free aliphatic thiol (Figure S5, compound S2), were not tolerated, consistent with previous observations of Pd active site overbinding.^43,44^ Remarkably, benzofuran was also arylated under our optimized conditions to give **15**, albeit in a lower yield of 27%. Varying the electronics of the iodonium salt highly influenced the yields. Hammett-neutral *para*-F substitution afforded **16** in an excellent 83% yield, while electron-withdrawing (*para*-CF_3_, **17**, 30%) and electron-donating functionalities (*para*-Me, **18**, 47%) showed diminished yields. Interestingly, such a trend was not observed for the homogeneous system.

### 2.3 Catalyst Activation Mechanism

To understand the origin of catalyst activation, we first examined the reaction kinetics by ex situ ^19^F qNMR. Under the optimized reaction conditions, the formation of product **2** revealed a sigmoidal kinetic profile with an induction period (Figure 4a, Table S18). This behavior suggests an autocatalytic activation process, as Pd leaching was ruled out in previous experiments. We hypothesized that acid generation may be responsible for this effect, as the reaction formally releases one equivalent of HBF_4_. Injecting 5 mol% of HBF_4_·OEt_2_ at the start of the reaction resulted in immediate reactivity, albeit at a lower overall yield due to increased decomposition (Figure 4b, Table S18).

**Figure 4:**
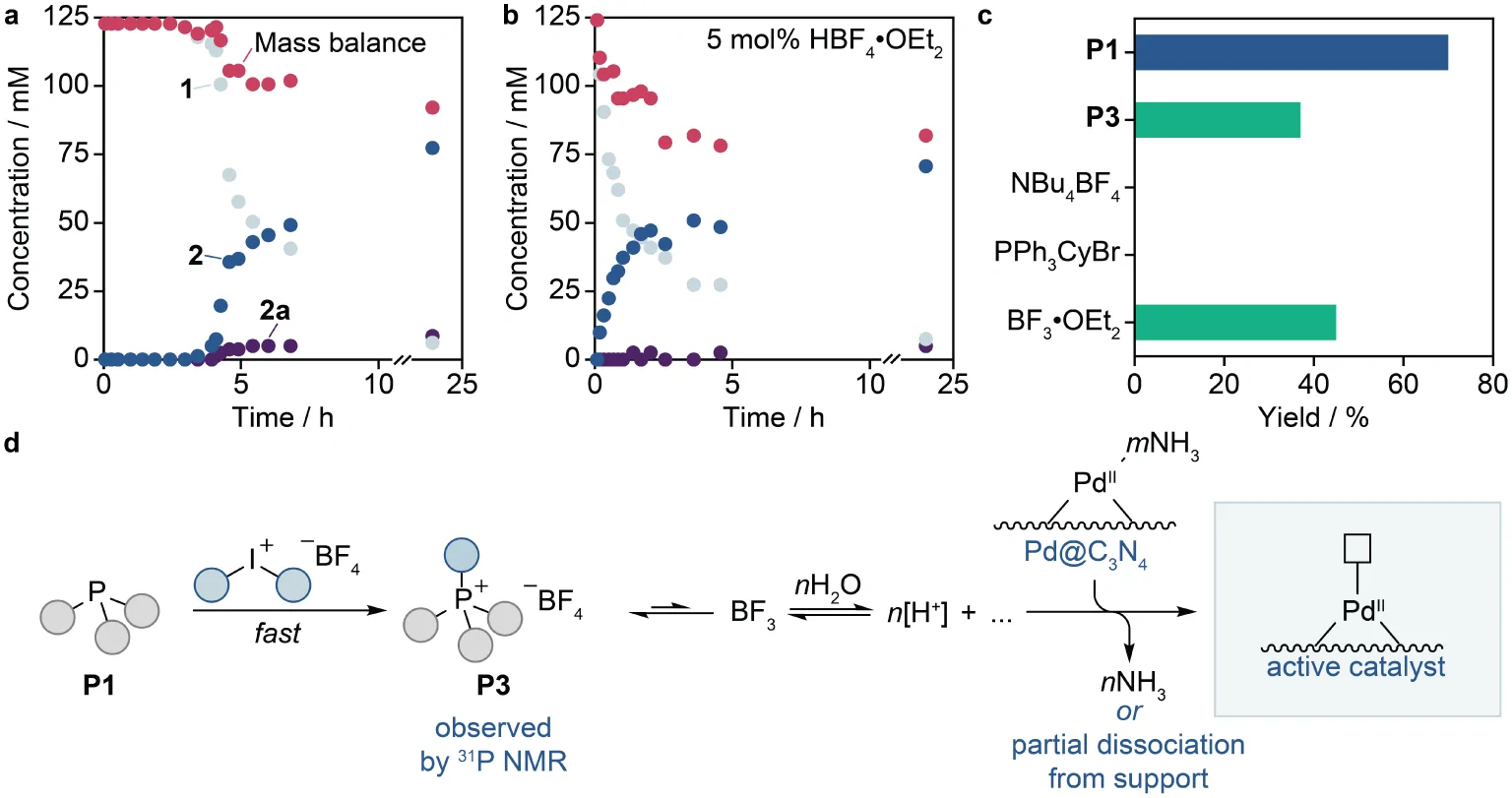
(a) Kinetic profile of the phosphine-promoted C2 arylation of **1** over Pd@C_3_N_4_. Conditions as in Figure 3d. **2a** refers to the C3 arylation product. (b) Kinetic profile with the addition of HBF_4_·OEt_2_ (5 mol%). (c) Investigation of the phosphine role by replacing it with other species. (d) Proposed mechanism of Pd@C_3_N_4_ activation by phosphine.

Nonetheless, the link between the acid-mediated autocatalytic pathway and the phosphine was not obvious, especially considering that 0.1 mol% was sufficient to achieve good yields (Table S19). ^31^P NMR analysis of the reaction mixture showed a single signal at +26.23 ppm (Figure S6), which we assigned to the phosphonium salt PPh_3_CyBF_4_ (**P3**) by in situ and ex situ comparison with exogenous PPh_3_CyBF_4_ (Figure S6). Interestingly, phosphonium **P3** was observed as the sole species in the ^31^P NMR after the first 15 min of reaction, while no significant formation of arylated product **2** was observed in the first 4 h. Phosphonium **P3** appeared as the most probable initiator for the reaction. Indeed, performing the reaction in the presence of 5 mol% of phosphonium **P3** instead of **P1** under optimized conditions gave the product in 37% yield (Figure 4c). The reaction did not proceed with stoichiometric phosphonium **P3** in the absence of phosphine **P1** and Ph_2_IBF_4_, showing that it is not an active aryl-transfer reagent (Table S19). Using (*n*-Bu)_4_NBF_4_ instead of **P1** under standard conditions did not yield any of the arylated product **2**, suggesting that phosphonium **P3** was also not acting as a phase-transfer catalyst. In addition, adding PPh_3_CyBr resulted in no reactivity, indicating the importance of combining BF_4_^–^ and PPh_3_Cy^+^. As noted during reaction optimization, reaction preparation under inert conditions resulted in diminished yield (Figure 3a, entry 2), but the addition of stoichiometric water partially restored activity (Table S19), suggesting that water was beneficial to reactivity. Thus, we hypothesized that phosphonium **P3** slowly releases BF_3_, which in the presence of water forms an acid that activates Pd@C_3_N_4_ (Figure 4d).^45–49^ Employing 5 mol% of BF_3_·OEt_2_ instead of **P1** furnished the arylated product **2** in 44% yield (Figure 4c), indirectly supporting our hypothesis. Screening other Lewis acids for catalyst activation led to some product formation but did not match the performance of the phosphine protocol, indicating that simple acid generation does not fully explain the activating role of the phosphine (see Table S20). These observations could partly explain the reactivity of Al_2_O_3_- and TiO_2_-supported catalysts in DCE without phosphine as both materials are known to contain protic surface hydroxyls.^50,51^

A plausible catalyst activation mechanism is proposed in Figure 4d. As (NH_3_)_4_Pd­(NO_3_)_2_ was a superior Pd@C_3_N_4_ precursor compared to Pd­(NO_3_)_2_ (Figure 3a, entry 4), ammonia may remain bound to Pd after synthesis or deprotonate sites on the support material to accommodate stable active Pd sites. This hypothesis is supported by ammonia temperature-programmed desorption experiments that show an NH_3_ desorption peak at 280 °C (Figure S7), 80 °C above the temperature used during catalyst synthesis. Trace amounts of formed acid could facilitate ammonia dissociation from Pd, creating a free coordination site, allowing substrate binding and rapid catalysis. Alternatively, the same role could be envisaged for a nitrogen-binding site on the support.

### 2.4 Catalyst Recycling: The Challenge of Iodide Poisoning

Catalyst recyclability is the key advantage of heterogeneous systems; however, Pd@C_3_N_4_ deactivated quickly after the first cycle (Figure 5a). As Pd is expected to carry the greatest environmental burden^15^ and the phosphine was fully converted to **P3**, recovery studies focused on the catalyst. Based on the preceding analyses, Pd loss or aggregation can be ruled out as major contributors to this behavior (Figure 5b, Figure S2, Table S14). A distinct color change from yellow to pink (Figure S8) was observed after reaction, suggesting fouling and poisoning as the two most probable options. In contrast to Pd@Al_2_O_3_, no significant mass increase of Pd@C_3_N_4_ could be observed gravimetrically (Note S6), and ^19^F NMR of the digested catalyst did not show quantifiable amounts of fluorinated deposits (Figure S9), excluding fouling by organic residues as the main deactivation pathway.

**Figure 5:**
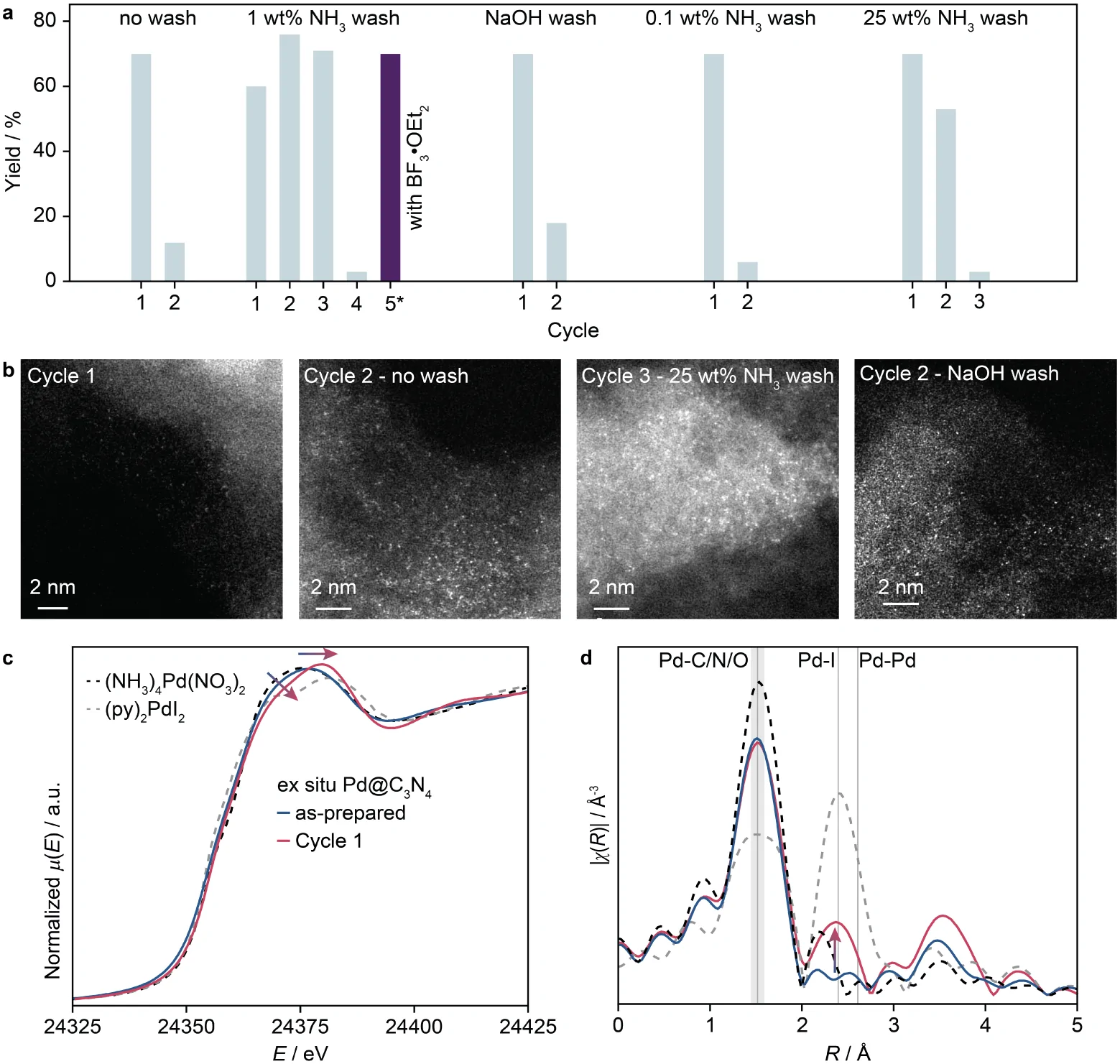
(a) Recycling tests of Pd@C_3_N_4_ using different washes between cycles. BF_3_·OEt_2_ (5 mol%) was added in cycle 5*. Initial conditions: 0.2 mmol indole, 2 equiv Ph_2_IBF_4_, 53 mg catalyst (1.25 mol%), DCE (125 mM), 16 h, 80 °C. Reaction scale was adjusted for each subsequent cycle. 1wt% aq. NH_3_ wash/reuse cycles shown were performed with **P2** (see Table S24 for **P1**). (b) HAADF STEM images of recovered catalysts having undergone different washes. (c,d) Ex situ XANES and EXAFS spectra of as-prepared and used Pd@C_3_N_4_ showing the effect of iodide poisoning. The used catalyst was not treated with aqueous ammonia before measurement.

When the used catalyst was investigated by ex situ XAS, clear changes in both the XANES (Figure 5c) and EXAFS (Figure 5d) were detected after the first cycle. Compared to the XANES spectrum of as-prepared Pd@C_3_N_4_, the whiteline intensity increased slightly and shifted to higher energy, accompanied by the emergence of a shoulder-like feature, characteristic of coordination by heavier elements (Figure S10, Note S7). These changes did not happen in the absence of phosphine, where no reactivity was observed (Figure S11). As microscopy (Figure 5b, Figure S2) did not show Pd sintering, iodide remains as the most likely option based on reaction conditions.

EXAFS analysis revealed the appearance of a new scattering path at approximately 2.4 Å (Figure 5d), which is consistent with Pd–I coordination, as confirmed by comparison with PdI_2_ and py_2_PdI_2_ reference compounds (Figure S10). Furthermore, EXAFS fitting including a Pd–I path yielded an average coordination number of ∼0.8, whereas Pd–Pd fits were not physically meaningful. The remaining bound iodide could explain both the pinkish color change of the used catalyst and the activity loss in the second cycle. The available evidence is consistent with poisoning arising from the reaction of Pd with the aryl iodide released from the iodonium salt, although the exact pathway of Pd–I formation cannot be established. Notably, such persistent Pd–I species have not been reported in cross-coupling applications over SACs despite the extensive use of aryl iodides. This further highlights the mechanistic distinction: in cross-coupling, the nucleophilic partner would displace the iodide ligand in the transmetalation step, which cannot occur during C–H functionalization.

Control experiments further support this assignment. Catalyst prepared from PdI_2_ gave a significantly reduced yield (Table S13), while addition of exogenous iodide completely suppressed activity (Table S19). Adding iodobenzene at the start of the reaction likewise reduced the yield (Table S19), consistent with iodide poisoning resulting from reaction with the aryl iodide. This may also explain the comparatively low yield and conversion observed for iodide-substituted product **11**, as oxidative addition into the C–I bond of 5-iodoindole provides an additional source of iodide.

Attempts to prevent iodide poisoning through reaction environment modulation were unsuccessful (Table S21). Iodide scavengers (e.g., AgOTf) proved ineffective, likely due to precipitation of AgI inside the catalyst pores or competing interactions with the catalyst. Instead, the use of asymmetric, sterically hindered iodonium salts to prevent oxidative addition into the aryl iodide hampered the initial arylation cycle from proceeding efficiently.

To probe the evolution of Pd species during catalysis, we performed in situ XAS measurements. During the induction period, analysis of the XANES shape (Figure 6a and b) indicates that the Pd sites exhibit a slightly electron-deficient character (Note S7), consistent with the activated catalytic state and formation of phosphonium species **P3**. As the reaction enters autocatalytic acceleration, linear combination analysis shows that this component diminishes and is replaced by a growing contribution from iodide-coordinated Pd species (Figure 6c). Thus, the iodide-containing fraction increases over time after the induction period, reaching significant levels before full conversion is achieved (Figure 6c, Table S22).

**Figure 6:**
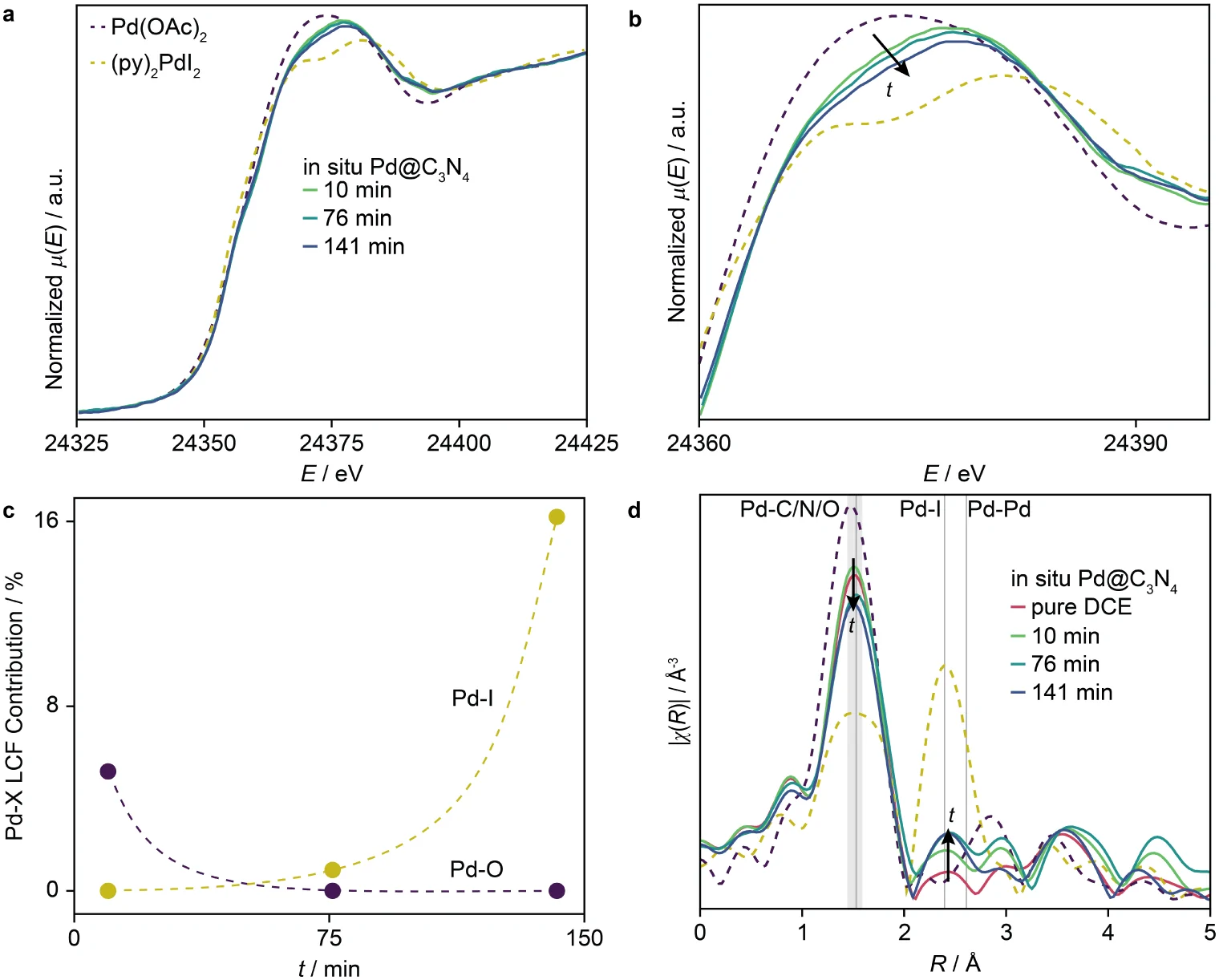
(a) In situ XANES spectra of Pd@C_3_N_4_ in the phosphine-promoted C2 arylation of indole **2**. Reaction conditions as in Figure 3. (b) Zoom of the whiteline region and (c) results of linear component fitting (LCF) of the XANES spectra. Pd–O and Pd–I designate electron-deficient Pd modeled by Pd­(OAc)_2_ and iodide coordination according to py_2_PdI_2_, respectively (Note S7). (d) In situ EXAFS spectra of Pd@C_3_N_4_ in the phosphine-promoted C2 arylation of indole **2**. Pd@C_3_N_4_ in pure DCE is included as a reference.

In the absence of indole, no change to the Pd signal is observed even after 200 min, confirming that iodide coordination arises under catalytic turnover and not from direct reaction with the iodonium salt (Figure S11, Table S22). These observations were further supported by EXAFS (Figure 6d). Under reaction conditions, the peak associated with the scattering path to C/N/O decreases while the Pd–I path becomes more prevalent. These qualitative changes are reflected in the coordination numbers obtained by EXAFS fitting (Table S23). As full conversion cannot be reached in this time frame, these results suggest that the Pd–I formation occurs throughout the reaction, not only toward the end.

### 2.5 Catalyst Regeneration via Mild Post-Treatment

Given that iodide poisoning cannot be prevented, catalyst regeneration remained the only viable strategy. To achieve that, regeneration would require displacement of iodide from the Pd coordination sphere by a ligand that would not subsequently poison the catalyst or lead to Pd leaching. We therefore sought a treatment capable of achieving this under mild conditions by exploiting the thermal or pH lability of Pd ligands. For example, amine-ligated Pd complexes are known to decompose in the desired temperature range^52^ or may dissociate from the metal under acidic conditions. The use of ammonia was further motivated by the superior performance obtained with (NH_3_)_4_Pd­(NO_3_)_2_ as the palladium precursor. Promisingly, 1% aqueous ammonia washes followed by catalyst treatment at 200 °C restored catalytic activity for up to three cycles (Figure 5a, Table S24), leading to total turnover numbers around 150. ICP-OES analysis confirmed substantial removal of iodine from the catalyst and its recovery in the wash solution (Table S25), while the XANES whiteline of the washed catalyst decreased to the intensity of the as-prepared catalyst (Figure S13). In contrast, a wash utilizing hydroxide ions, which exist in equilibrium with aqueous ammonia, was ineffective (Figure 5a), indicating that ammonia coordination rather than pure basicity enables regeneration. Consistently, treatment of the fresh catalyst with aqueous NaOH led to complete deactivation (Table S24), likely due to hydroxide overbinding at Pd sites as no sintering was observed (Figure 5b, Figure S2).

The efficiency of regeneration depends strongly on the NH_3_ concentration. While dilute treatments (0.1 wt% ammonia) are insufficient, higher concentrations (25 wt%) are also detrimental to the catalyst (Figure 5a). Even at 1 wt%, while reactivity can be restored after the second cycle, it is not sustained beyond the third. This behavior suggests that excessive ammonia treatment modifies the catalyst support, either through structural changes or depletion of surface protons. Indeed, diffuse reflectance infrared spectroscopy (DRIFTS) analysis reveals a reduction of *N*–H functionalities upon ammonia treatment (Figure S12), and surface area measurements by N_2_ sorption indicate losses in both surface area and pore volume (Table S26).

Motivated by the role of surface protons, we explored whether reintroduction of acidity could restore activity over multiple cycles. Based on our activation studies, 5 mol% of BF_3_·OEt_2_ recovered catalytic performance in the fifth cycle, giving a cumulative turnover number above 200. Here, BF_3_·OEt_2_ was selected as a precursor to slowly release protons in the presence of water,^45^ as the immediate presence of larger acid amounts generally resulted in lower yields due to accelerated decomposition pathways (Figure 4b).

These results show that iodide poisoning can be mitigated through targeted ligand exchange using ammonia, although long-term stability is limited by competing modifications of the support. The ability to partially restore activity through controlled acid addition highlights the dynamic interplay between coordination environment and catalytic performance in single-atom systems. More broadly, these findings underscore that regeneration of single-atom catalysts remains underexplored and inherently complex, governed by the coupled effects of ligand exchange, support chemistry, and reaction environment.

## 3 Conclusions

In this work, we demonstrated the first single-atom catalyzed thermal C–H functionalization through the direct arylation of indoles, achieving yields of up to 83%, with the aid of a phosphine additive. The phosphine does not act as a classical ligand but is transformed in situ into a phosphonium species that serves as an acid reservoir. In situ and ex situ XAS revealed the formation of persistent Pd–I bonds during the reaction, hindering direct recyclability of the catalyst. Such species have not previously been reported for single-atom catalysts in cross-coupling systems. Postreaction treatment with aqueous ammonia effectively restored catalytic activity via ligand exchange and iodide removal. However, repeated regeneration gradual modifies the C_3_N_4_ support, an effect that can be mitigated by controlled acid addition. These findings reveal the interplay between metal coordination, reaction medium, and support chemistry in determining catalyst performance, stability and regenerability. Beyond this specific transformation, this study establishes Pd single-atom catalysis as a viable approach for C–H arylation and highlights the importance of integrating reaction and regeneration strategies in the design of recyclable SACs for demanding liquid-phase transformations.

## Supplementary Material



## Data Availability

Data Statement Experimental procedures, characterization data, and spectra for all compounds are available free of charge. The data that support the findings of this study are openly available at Zenodo with reference number 20580576 at https://doi.org/10.5281/zenodo.20580576.
