# Supplementary material for: Regenerable Heterogeneous Palladium Single-Atom Catalysts for Direct C–H Arylation
Source: J Am Chem Soc. 2026 Oct 16;148(42):45600–9. doi: 10.1021/jacs.6c11697 (PMC13721157; doi:10.1021/jacs.6c11697)

Supporting Information

Regenerable Heterogeneous Palladium Single-Atom Catalysts for Direct C–H Arylation

Valentas Olikauskas,[a,f],§ *Marc-Eduard Usteri,*[b,f],§ *Aram Bugaev,*[c,f] *Milivoj Plodinec,*[d,e] *Sharon Mitchell,*[b,f] *Jérôme Waser,*[a,f],* *Javier Pérez-Ramírez,*[b,f],*

[a] V. Olikauskas, J. Waser, Laboratory of Catalysis and Organic Synthesis, Institute of Chemical Sciences and Engineering, École Polytechnique Fédérale de Lausanne, Avenue F. A. Forel 2, 1015 Lausanne, Switzerland

[b] M.-E. Usteri, S. Mitchell, J. Pérez-Ramírez, Institute of Chemical and Bioengineering, Department of Chemistry and Applied Biosciences, ETH Zurich, Vladimir-Prelog-Weg 1, 8093 Zurich, Switzerland

[c] A. Bugaev, Paul Scherrer Institute, Forschungsstrasse 111, 5232 Villigen, Switzerland

[d] M. Plodinec, Scientific Center for Optical and Electron Microscopy, ETH Zurich, Otto-Stern-Weg 3, 8093 Zurich, Switzerland

[e] M. Plodinec, Laboratory of Inorganic Chemistry, Department of Chemistry and Applied Biosciences, ETH Zurich, Vladimir-Prelog-Weg 2, 8093 Zurich, Switzerland

[f] V. Olikauskas, M.-E. Usteri, A. Bugaev, S. Mitchell, J. Pérez-Ramírez, J. Waser, NCCR Catalysis, 8093 Zurich, Switzerland

Table of Contents

Table of Contents 2

Supplementary Methods 3

Supplementary Notes 8

Supplementary Tables 15

Supplementary Figures 47

Supplementary Synthetic Methods 60

References 97

Spectra 100

Supplementary Methods

Materials

THF, Et_2O, CH_3CN, toluene, hexane and DCM were dried by passage over activated alumina under nitrogen atmosphere (H_2O content < 10 ppm, Karl-Fischer titration). All chemicals were purchased from Acros, Aldrich, Fluka, VWR, Aplichem, or Merck and used as such unless stated.

Catalyst synthesis

Carbon nitride (C_3N_4) was prepared following the previously reported polymerization of dicyandiamide.[1] Briefly, powdered dicyandiamide was dispersed in a ceramic crucible, covered with a second crucible, and polymerized at 550 °C for 4 h (2.3 °C min^{-1} ramp) in static air. The resulting material was ball-milled (10 min, 30 Hz), finely dispersed in a ceramic crucible, and thermally exfoliated at 500 °C for 5 h (5 °C min^{-1} ramp).

CeO_2 was prepared via the thermal decomposition of $Ce(NO_3)_3 \cdot 6H_2O$ at 400 °C for 5 h (5 °C min^{-1} ramp). **Al_2O_3** and **TiO_2** powders were obtained by crushing commercially available extrudates from Saint-Gobain and abcr, respectively.

Nitrogen-doped carbon (NC) was prepared from activated carbon (AC, Norit Rox 0.8) by mixing with concentrated nitric acid (1 g per 20 mL) and refluxing at 80 °C for 16 h. After pouring the suspension into ice-cooled deionized water (DIW), the powder was filtered off, thoroughly washed with DIW, and dried overnight (80 °C). The acid-treated AC was mixed with dicyandiamide (1:3 mass ratio) and ground in a mortar. The resulting solid mixture was carbonized via a two-step process (450 °C for 3 h, 600 °C for 2 h, 5 °C min^{-1} temperature ramp) in flowing nitrogen, yielding nitrogen-doped carbon.

Pd introduction: Palladium was stabilized by wet impregnation followed by thermal activation. Carrier material (2 g) was suspended in an aqueous solution of $(NH_3)_4Pd(NO_3)_2$ (284.4 mg, 10 wt% complex, 3.66 wt% Pd) diluted with DIW (10 mL) and stirred for 16 h. Then, the powder was isolated by centrifugation (10 min, 6000 rpm) and dried for 8 h at 70 °C

under vacuum. Finally, the catalyst was annealed at 200 °C, 500 °C or 800 °C (3 °C min^{-1} ramp) in static air for 12 h. When PdI_2 was used as a precursor for the impregnation procedure, excess KI (ca. 100 mg) was added to the aqueous solution to dissolve PdI_2 in the form of soluble $K_2[PdI_4]$. Complexation was not possible without excess KI. The catalyst was washed with DIW after annealing to remove unbound KI.

Catalyst characterization

A Horiba Ultra 2 instrument equipped with a photomultiplier tube detector was used for inductively coupled plasma-optical emission spectrometry (ICP-OES). To determine the metal content in as-prepared and used catalysts, the powders were mixed with an appropriate acid solution (**Table S1**) and digested at 240 °C for 30 min using an Anton Paar Multiwave 7000.

Fluorine content in the used catalysts was determined through ^{19}F nuclear magnetic resonance (NMR) spectroscopy by adding a known amount of $NaBF_4$ as an internal standard and D_2O to the digested catalyst. ^{19}F NMR spectra were acquired with a Bruker 300 MHz spectrometer at room temperature.

Pd leaching was determined by inductively coupled plasma-mass spectrometry (ICP-MS), where the EPFL mass analysis service team performed sample preparation and measurements. Briefly, 2.0 mL of concentrated HNO_3 was added to each sample dried out in glass vials. The vials were vortexed for 2 min, and 1.8 mL of the obtained solutions were transferred into individual PP digestion tubes to perform acidic digestion using a heating block system (DigiPREP Jr. 15 mL, 40 Pos, SCP Science). Digestion was achieved by heating to 100 °C over 15 min and holding at 100 °C for 55 min. After the digestion, samples were diluted to a final volume of 5.0 mL with Milli-Q water. A further dilution of 50 times with 2% HNO_3 solution was performed for each sample. The ICP-MS analysis was run in KED mode with He as a collision gas on the NexIon 350 D ICP-MS instrument (PerkinElmer). Y was added as an

internal standard at a concentration of 2 ppb to all solutions, and absolute quantification was performed using an external calibration curve with standards in the 0.05-50 ppb range.

High-angle annular dark field scanning transmission electron microscopy (HAADF-STEM) images were acquired on a scanning transmission electron microscope (STEM) JEOL GrandARM operated at 300 kV.

X-ray diffraction (XRD) diffractograms were acquired with a Rigaku SmartLab equipped with a Cu source in the 2θ range of 10-70°.

Temperature-programmed desorption experiments were conducted on a Micromeritics Autochem III unit equipped with a thermal conductivity detector (TCD). The catalyst was stabilized in a U-shaped tube at the height of the thermocouple with quartz wool. The catalyst was pretreated with a 20 mL min^{-1} He flow and heated to 200 °C for 2 h to remove any adsorbates from the surface. After cooling the catalyst bed to 40 °C, He was bubbled through a heated flask (70 °C) containing the desired solvent and flown through the catalyst bed until the TCD showed a stable signal, indicating no further adsorption. The catalyst was heated under pure He flow to 600 °C at a rate of 5 °C min^{-1} to monitor solvent desorption.

X-ray absorption spectroscopy

Pd *K*-edge (E_0 = 24.3503 keV) X-ray absorption spectroscopy (XAS) measurements were conducted at the SuperXAS beamline of the Swiss Light Source at the Paul Scherrer Institute (PSI), Villigen, Switzerland.[2] The incident photon beam provided by a 2.9 T superbend magnet was selected using a Si(311) channel-cut Quick-EXAFS monochromator oscillating at 1 Hz.[2] The rejection of higher harmonics was done by Pt-coated mirror. Ex situ reference compounds were thoroughly mixed with cellulose and pressed into pellets while fresh and used catalysts were packed inside quartz capillaries. For in situ measurements, the reaction mixture was stirred and held at 80 °C for the desired time before transferring to the motorized measurement stage. V-shaped vials made from regular glass were used to minimize absorption through the walls

and disturbances due to catalyst settling. The vials were aligned perpendicular to the direction of the focused X-ray beam (slit size ca. 0.3-0.6 mm) while the capillaries were aligned along the beam to maximize the optical pathway through the sample. Spectra were acquired for 20 min at a rate of 2 spectra per second, followed by averaging to optimize the signal-to-noise ratio. The signals were collected in transmission mode through ionization chambers filled with Ar/N_2 (0.4 bar to 1.6 bar) with a background absorption of *ca*. 6%. The raw data were processed using the ProQEXAFS software package,[3] and the averaged spectra were further analyzed using the Demeter package.[4] X-ray absorption near-edge structure (XANES) and extended X-ray absorption fine structure (EXAFS) portions of the spectra were analyzed by linear combination fitting (LCF) and EXAFS fitting, respectively. Statistical validity of the models was ascertained using the *R*-factor, and the procedure is exemplified in **Figure S16**. Fitting parameters are reported in **Table S3**, **Table S4**, **Table S22**, and **Table S23**.

Catalyst evaluation

General procedure S1 (**GP-S1**): To an oven-dried conic vial with Pd-SAC (53 mg, **Table S1** for Pd mol%), Ph_2IBF_4 (73.6 mg, 200 µmol, 2.00 equiv) a solution of 5-fluoro-1-methylindole (14.9 mg, 100 µmol, 1.00 equiv) in a solvent (800 µL, 125 mM) was added under air. The vial was sealed and stirred (1000 rpm) on a pre-heated hotplate at 60 °C for 2 h, or as specified. Afterward, the mixture was filtered through a silica plug (ca. 1 g) and washed with DCM. The mixture was concentrated under reduced pressure (45 °C, 500 mbar), diluted with $CDCl_3$, and analyzed by 19F NMR with 1,4-difluorobenzene (11.4 mg, 10.3 µL, 100 µmol, 1.00 equiv) or 1-fluoronaphthalene (14.6 mg, 12.9 µL, 100 µmol, 1.00 equiv) as an internal standard. All reactions were carried out in oven-dried glassware under air unless stated otherwise. If additives, such as phosphines or bases, were used, they were added before (if solid) or after (if liquid) solvent addition in the specified amounts.

Product quantification/characterization

Chromatographic purification was performed by flash chromatography using Macherey-Nagel silica 40-63, 60 Å, with the indicated solvents as eluents under 0.1-0.5 bar pressure. For quantitative flash chromatography, technical grade solvents were used. For analysis by flash chromatography, HPLC grade solvents from Sigma-Aldrich were used. TLC was performed on Merck silica gel 60 F254 TLC glass plates or aluminum plates and visualized with UV light, permanganate stain, CAN stain, or Anisaldehyde stain. Melting points were measured on a Büchi B-540 melting point apparatus using open glass capillaries; the data is uncorrected. ^{1}H NMR spectra were recorded on a Bruker DPX-400 400 MHz spectrometer in $CDCl_3$ or DMSO; all signals are reported in ppm with the internal chloroform signal at 7.26 ppm, and the internal DMSO signal at 2.50 ppm as standards. The data is being reported as (s = singlet, d = doublet, t = triplet, q = quadruplet, qi = quintet, m = multiplet or unresolved, bs = broad signal, app = apparent, coupling constant(s) in Hz, integration, interpretation). ^{13}C NMR spectra were recorded with ^{1}H-decoupling on a Bruker DPX-400 100 MHz spectrometer in $CDCl_3$ or DMSO, all signals are reported in ppm with the internal chloroform signal at 77.16 ppm, and the internal DMSO signal at 39.52 ppm as standards. High-resolution mass spectrometric measurements were performed by the mass spectrometry service of ISIC at the EPFL on a MICROMASS (ESI) Q-TOF Ultima API. Infrared spectra were recorded on a JASCO FT-IR B4100 spectrophotometer with an ATR PRO410-S, a ZnSe prism, and are reported as cm^{-1} (w = weak, m = medium, s = strong, br = broad).

Supplementary Notes

Note S1 | Comment on performance of SACs beyond carbon nitride. Activity differences presented in **Figure 2b** can have multiple origins, including Pd sites lacking available binding sites for substrates, more rigid binding environments, or incompatible electronic states. Some of these aspects may be investigated by XAS. As catalysts based on CeO_2 and AC show poor selectivity for the target product (**Table S2**), they were not considered for further analysis.

Qualitative comparisons of the XANES portion of the XAS spectra show a higher similarity of the Al_2O_3- and TiO_2-based systems with the PdO reference (PdO_4 environment), while Pd@C_3N_4 and Pd@NC are more like the tetraamine palladium nitrate complex (PdN_4 environment). This agrees with the elemental composition of the carriers and the expected coordination environment. Consequently, the whiteline intensity suggests an initial Pd(II) oxidation state in all cases, ruling out distinct oxidation states as the origin of activity differences.

The true composition cannot be elucidated further, as EXAFS analysis cannot differentiate between C, N, and O (NC and C_3N_4) or different oxygen functionalities (Al_2O_3 and TiO_2). Bond lengths to second-period elements (C/N/O) are within the expected range for all catalysts. Surprisingly, Pd@NC showed the lowest coordination number at 3 (**Table S4**), which could be interpreted to imply the existence of a free coordination site and higher activity. However, this coordination number only refers to the static value in ex situ conditions and does not give any further insight towards the behavior under reaction conditions.

Beyond the first shell, Pd@Al_2O_3-500, Pd@C_3N_4 and Pd@NC do not show paths associated with Pd-Pd or Pd-O-Pd. Instead, Pd@TiO_2-500 shows scattering paths typically associated with PdO, albeit with a much lower coordination number than the Pd-O path compared to the bulk oxide (**Table S3** and **Table S4**). Combined with STEM imaging (**Figure S1**), that shows the existence of Pd-containing clusters in Pd@TiO_2-500, this supports the coexistence of Pd single

atoms with PdO clusters. For the other catalysts, microscopy supports predominantly atomic Pd dispersion, consistent with the XAS analysis. As such, the low activity of N-containing carriers cannot originate from speciation differences.

The observed speciation difference could explain why TiO_2-based systems were the only ones affected by annealing temperature. In contrast, Al_2O_3 and CeO_2-based systems essentially showed the same performance; the yield and conversion dropped for TiO_2 as the activation temperature increased (**Table S2**). This trend continued even when the temperature increased to 800 °C (**Table S2**). Thus, the activity loss was likely caused by loss of Pd active sites, which did not happen for the other catalysts.

Note S2 | Comment on hot filtration procedures and catalytically active leached Pd. With the standard hot filtration method (**Figure S4**), minimal changes in yield were observed in the 16 h after filtration (**Table S5**), while conversion increased in some cases. Nonetheless, the conversion levels reached after hot filtration were lower than those reached in 16 h tests without filtration (**Table S2**). Together, these results would suggest a parallel decomposition of **1** with the absence of catalytically active leached (CAL) Pd.

However, ICP-MS analysis of the filtrate showed that the catalysts lost between 1.3%-30% of their initial Pd content when stirred in the AcOH solvent (**Table S6**). As hot filtration can only identify CAL Pd but not leaching overall, we evaluated an amount of $Pd(OAc)_2$ equivalent to the amount lost by Pd@Al_2O_3, achieving 64% conversion and 40% yield in 16 hours (**Note S8**), which is half the yield over an eight times longer period than Pd@Al_2O_3.Thus, the formation of CAL Pd with associated yield increase should have been possible for all catalysts. As discussed in **Section 2.2**, adding fresh reagents after filtration could solve this problem and capture the contribution of CAL Pd.

Adding fresh reagents after filtration restores the initial reagent concentrations for the leached Pd, allowing its contribution to the overall yield to be estimated. By matching the reaction times

before and after filtration at 2 h, the results indicate a significant heterogeneous contribution to both reaction kinetics and product yield (**Table S7**).

Note S3 | Comment on solvent overbinding. Although various solvents were evaluated **(Table S2** and **Table S10)**, only halogenated solvents like DCE showed good performance. The halogenated solvents are sufficiently polar to fully dissolve all reagents and prevent the formation of a solid-solid-liquid reaction. At the same time, they are generally not considered to coordinate to metal atoms like nitrogen-containing solvent.[5,6] As the solvent atoms are present in large excess, strong coordination of the solvent could completely prevent the catalytic cycle from starting. The difference in solvent binding could be exemplified by temperature-programmed desorption experiments of MeCN and DCE over Pd@Al_2O_3-500 (**Figure S14**). Under standard conditions, both solvents have a boiling point in the range of 80-85 °C.[7,8] Instead, when the desorption curves are compared, DCE still evaporates at its boiling point while MeCN shows three distinct desorption temperatures with the lowest one at 160 °C. Thus, it appears that DCE mostly physisorbs to the catalyst surface whereas MeCN chemisorbs to it, highlighting the N-Pd coordination of the solvent.

Note S4 | Comment on Pd aggregation over metal oxide systems. Hot filtration results shown in **Figure 2c** show that higher annealing temperatures lead to better Pd anchoring, as evidenced by the 25-88% lower yield gain after filtration (e.g., 29% yield increase reduced to 8% when comparing Pd@Al_2O_3 with Pd@Al_2O_3-500, **Figure S4**, **Table S7**). Unfortunately, some Pd sites remained unstable, as evidenced by XAS measurements of the used catalysts (**Figure S3**). For both Al_2O_3 and TiO_2 systems, a reduction in the XANES whiteline intensity corresponding to Pd reduction could be observed, while EXAFS showed Pd sintering through reduction of the Pd-O path and associated coordination number (**Table S4**) as well as an increase of Pd-Pd and Pd-O-Pd contributions (**Table S4**), respectively. We note that XRD measurements could not capture these species (**Figure S15**).

Note S5 | Comment on the use of Ph_2IPF_6. Following the results shown in **Table S12**, use of PF_6^- might seem like a good choice to improve yield and selectivity towards the main product **2**, in the arylation of indole **1**. Unfortunately, further analysis by ICP-MS (**Table S14**) showed that this led to significantly more Pd leaching compared to BF_4^-. Thus, the higher yield is partially due to catalytically active leached Pd, which seems to have better selectivity to the product. Hence, Ph_2IPF_6 was not used further despite its better performance.

Note S6 | Comment on apparent Pd loss and catalyst fouling. Based on ICP-OES measurements of as-prepared and used Pd@Al_2O_3-500 catalyst, it appears that ca. 38% of the Pd is lost due to the reaction (**Table S1**). However, ICP-MS analysis of the filtrate shows a Pd amount equaling a 14% loss (**Table S6**). Catalyst fouling can explain this discrepancy. It was gravimetrically determined that the catalyst mass increases by ca. 26% post reaction after catalyst drying. By adding both values, the ICP-OES Pd "loss" can be reobtained within error. While this discrepancy was not as extreme for Pd@C_3N_4 thanks to low fouling amounts, accurate gravimetric analysis is limited by quantitative catalyst recovery after the reaction.

Further insight into the nature of deposits could be gained from XRD and ^{19}F NMR analysis. The XRD diffractogram of used Pd@Al_2O_3 (**Figure S15**) shows a sharp reflection at 27°, typically associated with the (100) reflection of graphite or carbonaceous deposits.[9] Furthermore, as Pd@Al_2O_3 could be fully digested with a 25 wt% NaOH, the digested mixture could be analyzed ^{19}F NMR. As shown in **Figure S9**, digestion hydrolyzed all fluorine bonds releasing F^-. Thus, the origin of the fluorine cannot be tracked to indole or Ph_2IBF_4. As none of the BF_4^- was left, $NaBF_4$ was added as an internal standard to quantify the amount of deposited fluorine. 1.88 $\mu mol_F\ mg_{Cat}^{-1}$ were found, equal to 13.3 wt% of the total deposits as well as 13.4% of the total fluorine added to the reaction.

A similar XRD analysis was not possible for both C_3N_4 and CeO_2 as they have reflections at 27° (**Figure S15**). ^{19}F NMR was not possible for TiO_2 due to the digestion method (HF is

needed) and did not show any results for C_3N_4 (**Figure S9**). While this could be due to absence of fluorine deposits, the acidic digestion treatment could also lead to volatile HF that was not retained in the solution.

Note S7 | Comment on Pd XANES and analysis of in situ samples. Measurement of various reference compounds and complexes (**Figure S10**) shows that Gaussian-shaped whitelines are typical for Pd atoms surrounded purely by second-period elements, such as $Pd(OAc)_2$ and $(NH_3)_4Pd(NO_3)_2$. Instead, the presence of binding partners from higher periods always leads to shoulder-like features (e.g., py_2PdI_2, $(PPh_3)_2Pd(NO_3)_2$) or even a distinct additional oscillation (e.g., Pd foil), irrespective of the formal Pd oxidation state (**Figure S10**).

The XANES spectra of all in situ catalysts showed two features (**Figure S11**). Firstly, their whiteline intensities were higher than ex situ Pd@C_3N_4 or any reference compound measured except for $Pd(OAc)_2$ (**Figure S10** and **Figure S11**). Secondly, the shape of the whitelines were non-gaussian and all showed onset of shoulder formation, including Pd@C_3N_4 in pure DCE. This observation could be due to different interactions between air (O_2) and DCE (Cl) with the Pd atoms. However, understanding them would require a dedicated XAS study outside the scope of this work. Additionally, it would not help explain reaction-induced changes. Therefore, the spectrum of Pd@C_3N_4 in DCE was used as a reference for further analysis of reaction-induced changes in in situ experiments instead of Pd@C_3N_4 or $(NH_3)_4Pd(NO_3)_2$, following procedures in previous studies.[1,10]

To model the positive polarization or partial oxidation of Pd in the initial stages of the reaction, $Pd(OAc)_2$ had to be used as no other suitable reference compound could be found. Importantly, this does not imply coordination of acetate, which is not present in the reaction mixture.

Note S8 | Low $Pd(OAc)_2$ loading in AcOH

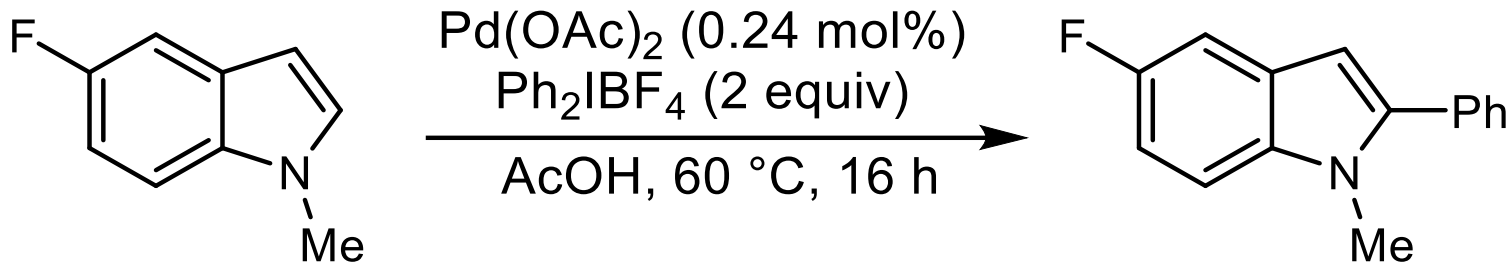

1 **2**

40% yield
64% conversion

*Stock solution **A***. To an oven-dried MW vial, $Pd(OAc)_2$ (5.30 mg, 23.6 µmol, 24.0 mol%) was added. The vial was evacuated and refilled with argon (3x), followed by the addition of acetic acid (10.0 mL).

To an oven-dried MW vial under argon with 5-fluoro-1-methylindole (14.9 mg, 100 µmol, 1.00 equiv) and Ph_2IBF_4 (73.6 mg, 200 µmol, 2.00 equiv), stock solution **A** containing $Pd(OAc)_2$ (100 µL, 53.0 µg, 236 nmol, 0.240 mol%) was added under argon atmosphere followed by acetic acid (700 µL). The resulting solution was stirred on a pre-heated hotplate at 60 °C for 16 h. Afterward, the mixture was filtered through a silica plug (ca. 1 g) and washed with DCM. The mixture was concentrated under reduced pressure (45 °C, 500 mbar), diluted with $CDCl_3$, and analyzed by ^{19}F qNMR with 1-fluoronaphthalene (14.6 mg, 12.9 µL, 100 µmol, 1.00 equiv) as an internal standard.

Note S9 | Possible mechanistic pathways for C-H metalation

Three possible mechanistic pathways are commonly proposed in the literature for electron-rich heteroarene C-H functionalization: direct concerted metalation-deprotonation (CMD, A), electrophilic C2 metalation (B), and electrophilic C3 metalation followed by Pd migration to C2 (C3). The measured $k_H/k_D = 0.85$ is inconsistent with pathway A, where a primary KIE would be expected. However, pathways B and C cannot be distinguished only using KIE measurements. For the proposed pathway C for a similar system,[11] the previously reported value of $k_H/k_D = 1.2$ is inconsistent with our findings. However, this cannot exclude pathway C, as the relative rates of C3 metalation and migration steps, and thus the observed KIE, may vary

between different systems. Additionally, we measured KIE via intermolecular competition instead of direct rate constant comparison, which was used to obtain $k_H/k_D = 1.2$ in the above-mentioned study. **Note**. While C-H metalation may not be a turnover-limiting step, measuring KIE by intermolecular competition gives information about the steps before the substrate-committing step.

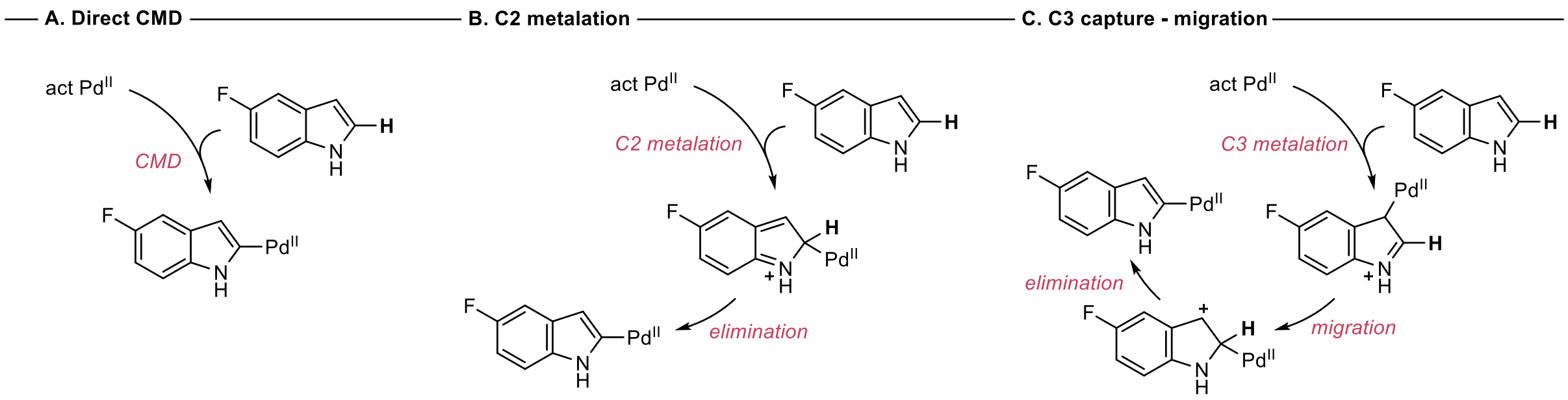

Supplementary Tables

Table S1. ICP-OES quantification of the Pd content of the as-prepared and selected used catalysts used in this study.

**Catalyst**	**Digestion mixture**	**Pd content as-prepared / wt%**	**Pd equivalents[a] / mol%**	**Pd content used/ wt%[c]**
Pd@Al_2O_3	25 wt% NaOH[b]	0.30	1.5	n.d.
Pd@CeO_2	Aqua regia	0.32	1.6	n.d.
Pd@TiO_2	48 wt% HF	0.59	2.9	n.d.
Pd@AC	Aqua regia	0.35	1.7	n.d.
Pd@NC	Aqua regia	1.00	5.0	n.d.
Pd@C_3N_4	Aqua regia	0.52	2.6	0.48[c], 0.52[d], 0.51[e], 0.48[f]
Pd@Al_2O_3-500	25 wt% NaOH[b]	0.28	1.4	0.17[g,h]
Pd@CeO_2-500	Aqua regia	0.30	1.5	n.d.
Pd@TiO_2-500	48 wt% HF	0.60	3.0	n.d.

[a] Refers to the Pd equivalents used in the reaction. [b] Aqueous solution of NaOH. [c] After three cycles in DCE with **P2**. [d] After one catalytic cycle in DCE with **P2** and one NaOH wash. [e] After one catalytic cycle in DCE with **P1** and one ammonia wash. [f] After three catalytic cycles in DCE with **P1** and ammonia washes between cycles. n.d.-not detected. [g] The measured Pd loss does not correspond to the actual loss due to catalyst fouling (**Note S6**). [h] After one cycle in DCE.

Table S2. Solvent and catalyst screening of the C2-arylation of substrate **1**.

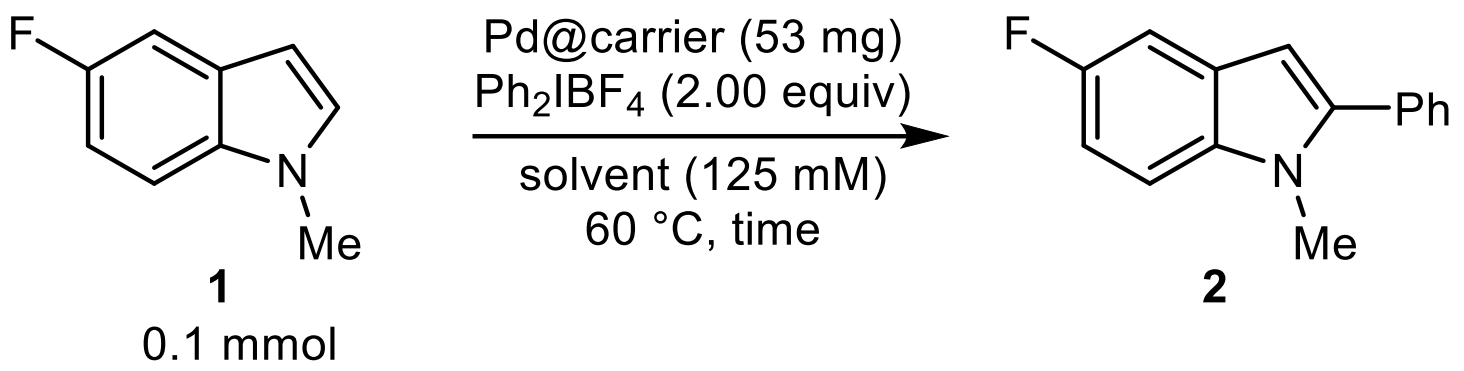

Entry[a]	Catalyst	Solvent	Time / h	X_1 / %	Y_2 / %
1	Pd@Al_2O_3	AcOH	2	88(1)[b]	76(2)
2	Pd@TiO_2	AcOH	2	87(3)	67(3)
3	Pd@CeO_2	AcOH	2	75	43
4	Pd@AC	AcOH	2	73	20
5	Pd@C_3N_4	AcOH	2	20(2)	17(1)
6	Pd@NC	AcOH	2	14	0
7	Pd@Al_2O_3-500	AcOH	2	81(1)	74(4)
8	Pd@TiO_2-500	AcOH	2	77(8)	46(4)
9	Pd@CeO_2-500	AcOH	2	72	37
10	Pd@Al_2O_3-800	AcOH	2	85	62
11	Pd@TiO_2-800	AcOH	2	21	3
12	Pd@Al_2O_3	AcOH	16	100	87
13	Pd@TiO_2	AcOH	16	100	50
14	Pd@CeO_2	AcOH	16	83	47
15	Pd@AC	AcOH	16	79	21
16	Pd@NC	AcOH	16	36	0
17	Pd@Al_2O_3-500	AcOH	16	94	79
18	Pd@TiO_2-500	AcOH	16	98	42
19	Pd@CeO_2-500	AcOH	16	81	52
20	Pd@Al_2O_3-800	AcOH	16	96	96
21	Pd@TiO_2-800	AcOH	16	35	12
22	Pd@Al_2O_3	DCE	16	70	52
23	Pd@TiO_2	DCE	16	100	43
24	Pd@CeO_2	DCE	16	12	0
25	Pd@AC	DCE	16	86	20
26	Pd@C_3N_4	DCE	16	9	2
27	Pd@NC	DCE	16	13	0

28	Pd@Al_2O_3-500	DCE	16	68.5	58
29	Pd@TiO_2-500	DCE	16	100	26
30	Pd@CeO_2-500	DCE	16	3	0
31	Pd@Al_2O_3-800	DCE	16	64	46
32	Pd@TiO_2-800	DCE	16	23	0
33	Pd@Al_2O_3	DMF	16	22	0
34	Pd@TiO_2	DMF	16	51	0
35	Pd@CeO_2	DMF	16	21	0
36	Pd@AC	DMF	16	19	0
37	Pd@NC	DMF	16	7	0
38	Pd@Al_2O_3-500	DMF	16	7	0
39	Pd@TiO_2-500	DMF	16	2	0
40	Pd@CeO_2-500	DMF	16	1	0
41	Pd@Al_2O_3-800	DMF	16	2	0
42	Pd@TiO_2-800	DMF	16	12	0
43	Pd@AC	MeCN	16	51	0
44	Pd@NC	MeCN	16	57	0
45	Pd@Al_2O_3-500	MeCN	16	12	0
46	Pd@TiO_2-500	MeCN	16	15	0
47	Pd@CeO_2-500	MeCN	16	14	0
48	Pd@AC	THF	16	84	14
49	Pd@NC	THF	16	25	0
50	Pd@Al_2O_3-500	THF	16	89	47
51	Pd@TiO_2-500	THF	16	87	29
52	Pd@CeO_2-500	THF	16	84	0

[a] Data were obtained following the **GP-S1**. [b] Standard deviation given between parentheses.

Table S3. EXAFS fitting parameters of different Pd reference compounds. The fit was performed in *R*-space relevant to the used paths using $k^{1,2,3}$-weighted data, Fourier-transformed in 3–10 Å^{-1} *k*-range. Reference materials were fitted using reference crystal structures obtained from ICSD[12–18] with variable damping (S_0^2), zero-potential correction (ΔE_0), interatomic distance (*R*), and Debye-Waller parameter (σ^2). The coordination number (*N*) was assigned to the theoretical value.

**Path**	**S_0^2 / -**	**ΔE_0 / eV**	***R* / Å**	**N^a / -**	**σ^2 / Å^2**	***R*-factor / %**
Pd foil (*R*-space: 1.5–3.4 Å)						
Pd-Pd[15]	0.79(9)	4.2(8)	2.739(5)	12	0.0050(8)	0.75
PdO (*R*-space: 1–4 Å)						
Pd-O[16]	0.74(7)	6(1)	2.017(9)	4	0.001(1)	
Pd-O-Pd1	0.74(7)	$0(2)^b$	$3.019(9)^c$	4	$0.004(1)^b$	0.83
Pd-O-Pd2	0.74(7)	$0(2)^b$	$3.41(1)^c$	8	$0.004(1)^b$	
$(NH_3)_4Pd(NO_3)_2$ (*R*-space: 1–2.5 Å)						
Pd-N[17]	0.81(8)	7(1)	2.040(9)	4	0.001(1)	0.60
PdI_2 (*R*-space: 1.5–3.2 Å)						
Pd-I[18]	0.78(6)	2.7(5)	2.609(4)	4	0.004(1)	0.42

[a] Fixed value. [b] Grouped variable used for ΔE_0 and σ^2. [c] Change to the ideal bond length was modelled using a single proportionality variable multiplying the ideal bond lengths.

Table S4. EXAFS fitting parameters from ex situ measurements of different Pd catalysts. The fit was performed in *R*-space relevant to the used paths as indicated using $k^{1,2,3}$-weighted data, Fourier-transformed in 3–10 $Å^{-1}$ *k*-range. The catalysts were fitted using the same paths used for the reference materials in **Table S3** with coordination number (*N*), zero-potential correction (ΔE_0), interatomic distance (*R*), and Debye-Waller parameter (σ^2). The damping (S_0^2) was fixed at 0.8 based on the value of a foil reference measured during the same beamtime.

System	Path	ΔE_0 / eV	*R* / Å	*N* / -	σ^2 / $Å^2$	*R*-factor / %
Pd@C_3N_4	Pd-N[a]	0(5)[b]	2.00(5)	3.3[c]	0.005(4)	0.96
	Pd-Cl[a]	0(5)[b]	2.17(3)	0.7[c]	0.002(7)	
Pd@C_3N_4-used	Pd-N[a]	-3(5)[b]	1.97(5)	2.9[c]	0.004(3)	1.51
	Pd-Cl[a]	-3(5)[b]	2.20(4)	1.1[c]	0.005(5)	
Pd@C_3N_4-used-PPh_3	Pd-N[a]	-9(5)[b]	1.91(7)	1.7[c]	0.003(7)	1.80
	Pd-Cl[a]	-9(5)[b]	2.17(3)	1.5[c]	0.001(2)	
	Pd-I	-9(5)[b]	2.64(4)	0.8[c]	0.004(3)	
Pd@NC	Pd-N	7(1)	2.020(8)	3.2(3)	0.003(1)	0.48
Pd@NC-used	Pd-N	8(1)	2.04(1)	4.0(4)	0.006(1)	0.66
Pd@NC-used-PPh_3	Pd-N	9(1)	2.05(1)	4.2(5)	0.006(2)	0.86
Pd@TiO_2-500	PdO	5.8(7)	2.013(5)[d]	3.5(2)	0.001(1)[b]	0.71
	Pd-O-Pd1	1(2)[b]	3.023(8)[d]	1.2(2)[e]	0.001(1)[b]	
	Pd-O-Pd2	1(2)[b]	3.412(9)[d]	2.4(2)[e]	0.001(1)[b]	
Pd@TiO_2-500-used	Pd-O	6.0(7)	2.020(5)[d]	3.3(2)	0.003(1)[b]	0.41
	Pd-O-Pd1	4(1)[b]	3.033(8)[d]	1.6(2)[e]	0.003(1)[b]	
	Pd-O-Pd2	4(1)[b]	3.423(9)[d]	3.2(2)[e]	0.003(1)[b]	
	Pd-Pd	1(4)	2.753(7)[d]	0.3(1)	0.003(1)[b]	
Pd@Al_2O_3-500	Pd-O	8(2)	2.02(2)	3.7(7)	0.003(3)	2.49
Pd@Al_2O_3-500-used	Pd-O	5(1)	2.010(6)[d]	2.0(2)	0.003(1)[b]	0.86
	Pd-Pd	0(1)	2.740(9)[d]	3.4(6)	0.003(1)[b]	

[a] A double contribution typical of Pd@C_3N_4 was observed in the EXAFS signal.[1,10] A Pd-Cl path was used to model. As no Cl or other 3rd period element was present during synthesis, it is more likely that this corresponds to a long Pd-N bond, which could be related to the structure of the binding site in the

heptazine moiety of C_3N_4. As these bonds are highly correlated, they should not be compared individually but against the Pd-I contribution. [b] Single grouped variable for the paths. [c] Total coordination number was fixed to 4. [d] Change to the ideal bond length was modelled using a single proportionality variable multiplying the ideal bond lengths. [e] Ratio of coordination numbers fixed to 2.

Standard hot filtration experiments (without additional starting materials)

General procedure S2 (**GP-S2**) for the original hot filtration. Starting from the same conditions as **GP-S1** under an N_2 atmosphere (Pd-SAC (53.2 mg), Ph_2IBF_4 (2.00 equiv), 5-fluoro-1-methylindole (0.100 mmol, 1.00 equiv), 1-fluoronaphthalene (1.00 equiv), acetic acid (0.800 mL)), the reaction mixture was stirred on a pre-heated hotplate at 60 °C for 2 h. After that time,

- An aliquot was taken and worked up.
- The reaction was filtered through a syringe filter (0.22 μm) under N_2 atmosphere and added to an oven-dried MW vial under N_2. The resulting mixture was stirred for another 16 h at 60 °C and then worked up.

Work-up: The mixture was filtered through a silica plug (ca. 1 g) and washed with DCM. The mixture was concentrated under reduced pressure (45 °C, 500 mbar), diluted with $CDCl_3$, and analyzed by ^{19}F qNMR.

Table S5. Catalyst performances and yields via the original hot filtration with different Pd-SACs in the C2-arylation of indole **1** in AcOH.

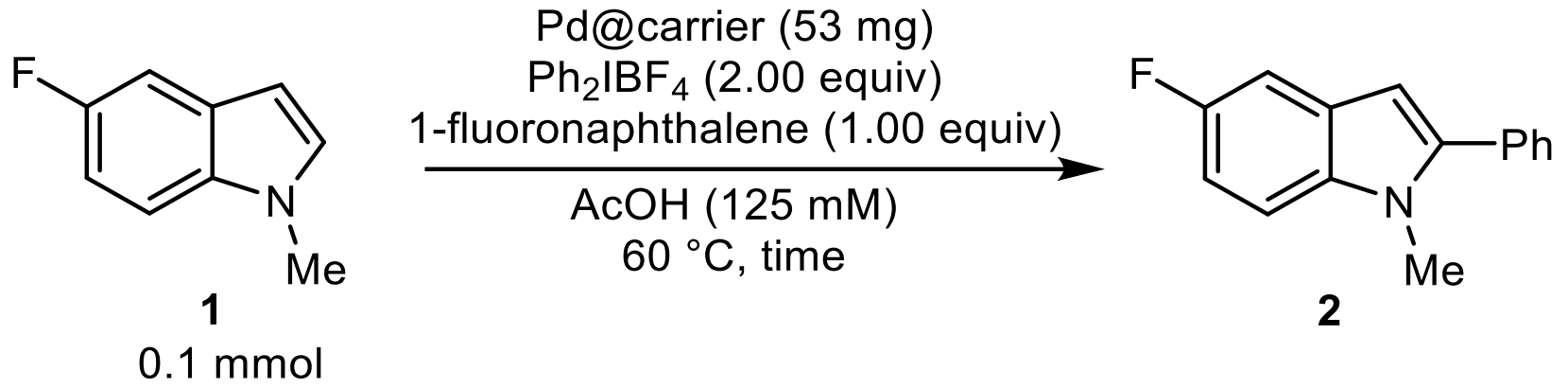

**Entry**[a]	**Catalyst**	**Time / h**	Y_2 **/ %**
1	Pd@Al_2O_3	2[b]	76(2)
1-HF		16[c]	77
2	Pd@TiO_2	2[b]	67(3)
2-HF		16[c]	67
3	Pd@CeO_2	2[b]	43
3-HF		16[c]	40
4	Pd@AC	2[b]	20
4-HF		16[c]	8
5	Pd@NC	2[b]	0
5-HF		16[c]	4
6	Pd@TiO_2-500	2[b]	46(4)
6-HF		16[c]	49
7	Pd@CeO_2-500	2[b]	37
7-HF		16[c]	43
8	Pd@Al_2O_3-800	2[b]	62
8-HF		16[c]	70
9	Pd@TiO_2-800	2[b]	3
9-HF		16[c]	11

[a] Data were obtained following the **GP-S2**. [b] Before filtration. [c] After filtration.

Catalyst stability in different solvents

To an oven-dried MW vial with Pd-SAC, AcOH (0.800 mL) or DCE (0.800 mL) was added in air. The resulting mixture was stirred (1000 rpm) on a preheated hotplate at 60 °C for 16 h. Afterward, the mixture was filtered through a syringe filter (0.22 μm), concentrated under reduced pressure, and analyzed by ICP-MS to determine the amount of leached Pd.

Table S6. ICP-MS quantification of leached Pd in the solvent filtrates.

**Catalyst**	**AcOH / μg**	**Pd loss[a] / %**	**DCE / ng**	**Pd loss[a] / %**
Pd@Al_2O_3	8.39	27.7	Below LOD[b]	-
Pd@TiO_2	42.24	64.0	16.50	0.005
Pd@CeO_2	2.52	8.8	Below LOD	-
Pd@AC	2.40	7.3	12.31	0.008
Pd@C_3N_4[c]	10.08[d]	3.8	Below LOD	-
Pd@NC	1.46	1.4	Below LOD	-
Pd@Al_2O_3-500	4.06	14.3	Below LOD	-
Pd@TiO_2-500	6.16	10.3	9.84	0.003
Pd@CeO_2-500	3.08	9.5	Below LOD	-
Pd@Al_2O_3-800	-[e]	-	Below LOD	-
Pd@TiO_2-800	-[e]	-	Below LOD	-

[a] Relative Pd loss of the solid catalyst. [b] Limit of detection = 2 ng. [c] 80 °C under **GP-S1** conditions. [d] 53 mg scale. [e] Not measured.

Modified hot filtration experiments (with additional starting materials after filtration)

General procedure S3 (**GP-S3**) for the modified hot filtration. Starting from the same conditions as **GP-S1** (Pd-SAC (53.2 mg), Ph_2IBF_4 (2.00 equiv), 5-fluoro-1-methylindole (100 μmol, 1.00 equiv), 1-fluoronaphthalene (1.00 equiv), acetic acid (800 μL)), the reaction mixture was stirred on a preheated hotplate at 60 °C for 2 h. After that time,

- An aliquot (ca. 50 μL) was taken and worked up.
- The remaining reaction mixture was filtered through a syringe filter (0.22 μm) and added to an oven-dried MW vial with 5-fluoro-1-methylindole (14.9 mg, 100 μmol, 1.00 equiv) and Ph_2IBF_4 (73.6 mg, 200 μmol, 2.00 equiv). The resulting mixture was stirred (1000 rpm) for another 16 h at a desired temperature and then worked up.

Work-up: The mixture was filtered through a silica plug (ca. 1 g) and washed with DCM. The mixture was concentrated under reduced pressure (45 °C, 500 mbar), diluted with $CDCl_3$, and analyzed by ^{19}F qNMR.

Table S7. Catalyst performances and yields via the modified hot filtration with different Pd-SACs in the C2-arylation of indole **1**.

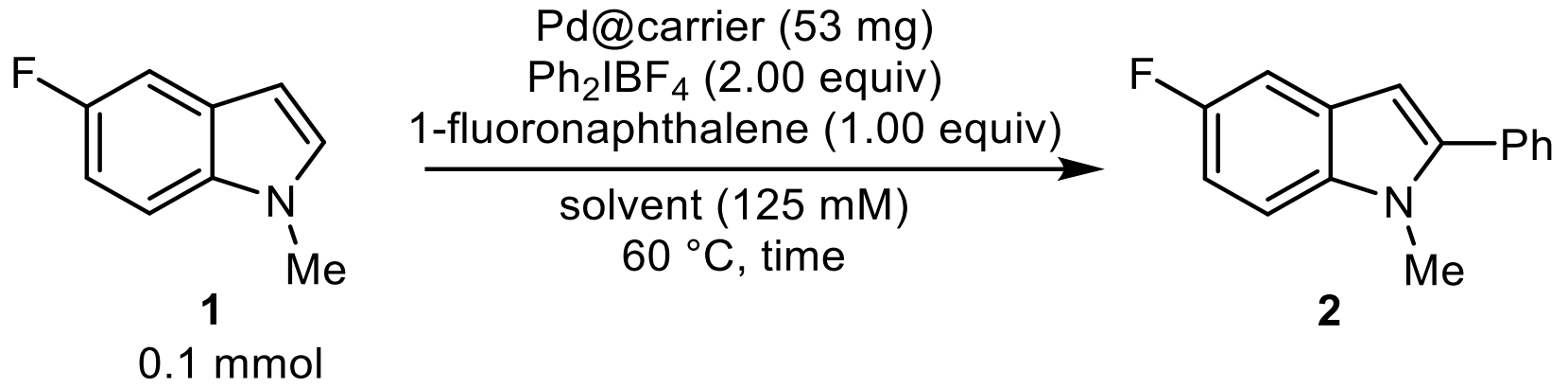

Entry[a]	Catalyst	Time / h	Solvent	Y_2 / %
1	$Pd@Al_2O_3$	2[b]	AcOH	76(2)
1-HF		16[c]		128
2	$Pd@TiO_2$	2[b]	AcOH	67(3)
2-HF		16[c]		95
3	$Pd@Al_2O_3$-500	2[b]	AcOH	74(1)
3-HF		16[c]		87
4	$Pd@TiO_2$-500	2[b]	AcOH	46(4)
4-HF		16[c]		77
5	$Pd@Al_2O_3$-500	2[b]	AcOH	74(1)
5-HF		2[c]		85
6	$Pd@Al_2O_3$	6[b]	DCE	68
6-HF		16[c]		97
7	$Pd@TiO_2$	2[b]	DCE	46
7-HF		16[c]		167
8	$Pd@Al_2O_3$-500	6[b]	DCE	64
8-HF		16[c]		72
9	$Pd@TiO_2$-500	2[b]	DCE	29
9-HF		16[c]		43
10	$Pd@Al_2O_3$-500	2[b]	DCE	0
10-HF		2[c]		0
11	$Pd@TiO_2$-500	2[b]	DCE	29
11-HF		2[c]		32
12	$Pd@TiO_2$-500	6[b]	DCE	53
12-HF		16[c]		67
13	$Pd@C_3N_4$	6[b]	DCE	0
13-HF		16[c]		0

[a] Data were obtained following the **GP-S3**. [b] Before filtration. [c] After filtration.

Catalyst stability to different reaction components of C-H arylation of indoles in DCE

- **Entry 1**. To an oven-dried MW vial with Pd@Al_2O_3 (53.2 mg) and Ph_2IBF_4 (2.00 equiv) under N_2 atmosphere, a solution of 5-fluoro-1-methylindole (100 μmol, 1.00 equiv) and 1-fluoronaphthalene (1.00 equiv) in DCE (800 μL) was added. The resulting mixture was stirred (1000 rpm) on a pre-heated hotplate at 60 °C for 16 h. After that time, the mixture was filtered through a syringe filter (0.22 μm), concentrated under reduced pressure, and analyzed by ICP-MS to determine the amount of leached Pd.
- **Entry 2**. Same as 1 without Ph_2IBF_4 and 1-fluoronaphthalene.
- **Entry 3**. Same as 1 without 5-fluoro-1-methylindole and 1-fluoronaphthalene.
- **Entry 4**. Same as 1 without 5-fluoro-1-methylindole and Ph_2IBF_4.

Table S8. ICP-MS analysis results of solutions obtained from the indole **1** arylation reaction or by heating Pd@Al_2O_3 in DCE with each reagent individually at 60 °C for 16 h.

Entry	Conditions	Leached Pd mass / ng
1	Indole **1** C-H C2-arylation reaction in DCE, 60 °C, 16 h	7706
2	Indole **1** in DCE, 60 °C, 16 h	27
3	Ph_2IBF_4 in DCE, 60 °C, 16 h	2674
4	1-fluoronaphthalene in DCE, 60 °C, 16 h	26

Solvent and catalyst screening with substoichiometric phosphine

General procedure S4 (**GP-S4**) for catalyst screening with substoichiometric phosphine. To an oven-dried MW vial with Pd-SAC (53.2 mg, **Table S1** for Pd mol%), Ph_2IBF_4 (73.6 mg, 200 µmol, 2.00 equiv), and phosphine (15.0 µmol, 15.0 mol%), a solution of 5-fluoro-1-methylindole (14.9 mg, 100 µmol, 1.00 equiv) in a solvent (800 µL, 125 mM) was added under Ar atmosphere. Then, a desired amount of air or water was injected into the vial via a syringe. The vial was sealed and stirred (1000 rpm) on a pre-heated hotplate for 16 h. After that time, the mixture was filtered through a silica plug (ca. 1 g) and washed with DCM. The mixture was concentrated under reduced pressure (45 °C, 500 mbar), diluted with $CDCl_3$, and analyzed by ^{19}F qNMR with 1,4-difluorobenzene (11.4 mg, 10.3 µL, 100 µmol, 1.00 equiv) or 1-fluoronaphthalene (14.6 mg, 12.9 µL, 100 µmol, 1.00 equiv) as an internal standard.

Table S9. Percentage composition of mixtures after the C2-arylation of **1** with Pd-SACs and PPh_3 phosphine (15 mol%) in DCE.

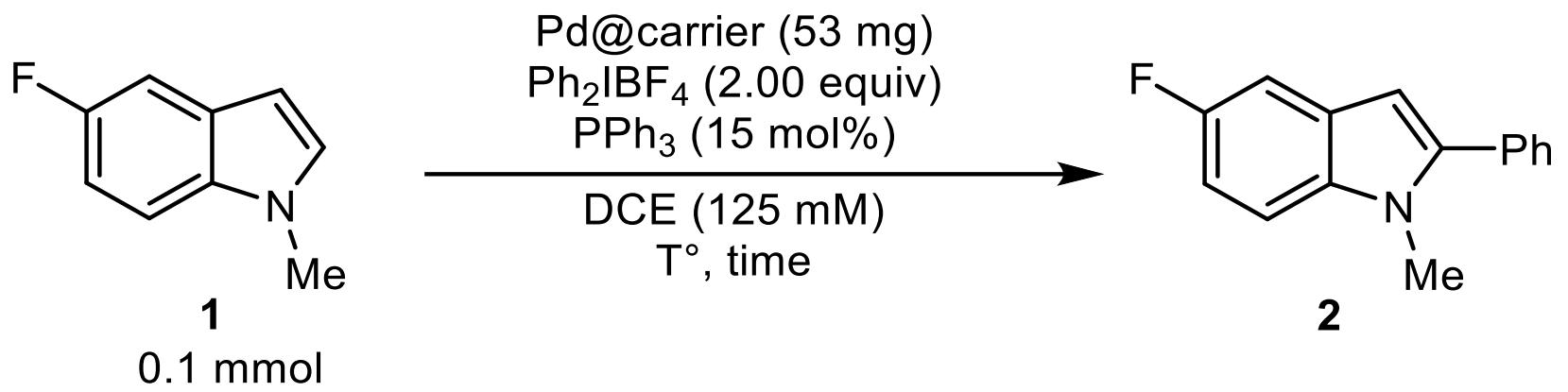

**Entry**[a]	**Catalyst**	**Time / h**	***T* / °C**	**Modification**	**X_1 / %**	**Y_2 / %**
1	Pd@NC	16	60	In air	26	8
2	Pd@CeO_2-500	16	60	In air	85	29
3	Pd@C_3N_4	16	60	In air	58.7	34
4	Pd@C_3N_4	16	60	0.15 equiv H_2O	54	21
5	Pd@C_3N_4	16	60	0.50 equiv H_2O	56	26
6	Pd@C_3N_4	16	60	0.75 equiv H_2O	53	21
7	Pd@C_3N_4	16	60	50 µL air	59	25
8	Pd@C_3N_4	16	60	100 µL air	49	18
9	Pd@C_3N_4	16	60	500 µL air	52	21
10	Pd@C_3N_4	72	60	In air	90	67
11	Pd@C_3N_4	16	80	In air	95	64.5
12	Pd@C_3N_4	16	100	In air	95	60

[a] Data were obtained following the **GP-S4**.

Table S10. Reaction optimization of the phosphine-promoted arylation of indole **1** with Pd@C_3N_4.

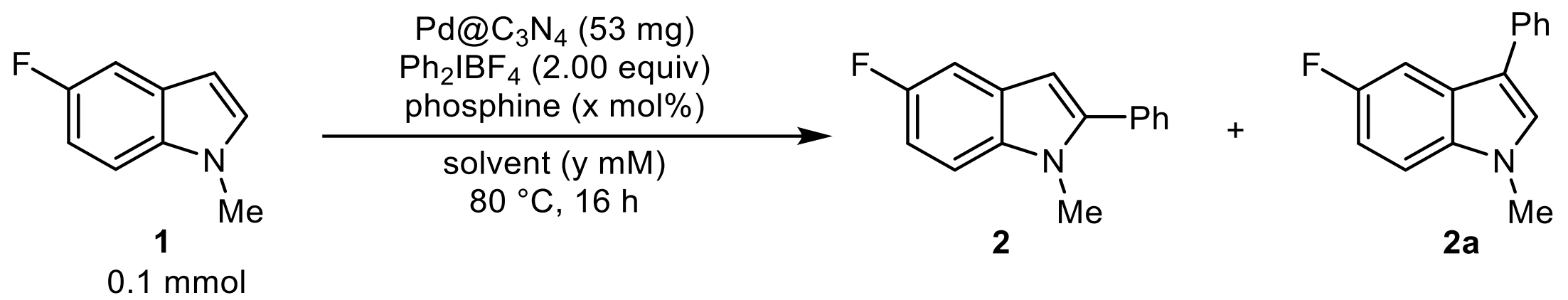

Entry[a]	Solvent	Phosphine	Volume / mL	Phosphine / mol%	X_1 / %	Y_2 / %	Y_{2a} / %
1	DCE	PPh_3	0.8	15	93(4)	62(5)	8.7(4)
2	AcOH	PPh_3	0.8	15	100	38	1
3	THF	PPh_3	0.8	15	93	8	4
4	MeOH	PPh_3	0.8	15	73	40	12
5	EtOAc	PPh_3	0.8	15	94	27	10
6	$CHCl_3$	PPh_3	0.8	15	83	54	9
7	MeCN	PPh_3	0.8	15	20	0	0
8	DMF	PPh_3	0.8	15	11	3	0
9	Acetone	PPh_3	0.8	15	90	10	2
10	Toluene	PPh_3	0.8	15	57	29	10
11	TFE	PPh_3	0.8	15	95	65	3
12	1,4-dioxane	PPh_3	0.8	15	66	39	14
13	DCE	PPh_3	0.5	15	94	61	9
14	DCE	PPh_3	1	15	90	60	11
15	DCE	PPh_3	1.5	15	87	60	12
16	DCE	PPh_3	2	15	85	57	13
17	DCE	PPh_2Cy	0.8	15	100(0)	73.5(1.5)	8(0)
18	DCE	CPhos	0.8	15	3	0	0
19	DCE	P(2-furyl)$_3$	0.8	15	11	0	0
20	DCE	P(p-OMePh)$_3$	0.8	15	92	60	8
21	DCE	PhDavePhos	0.8	15	16	4	1
22	DCE	P(OEt)Ph_2	0.8	15	76	39	8
23	DCE	P(o-MePh)$_3$	0.8	15	8	0	0
24	DCE	P(p-FPh)$_3$	0.8	15	98	62	8
25	DCE	P(p-MePh)$_3$	0.8	15	97	65	8
26	DCE	$P(C_6F_5)_3$	0.8	15	73	41	5

27	DCE	P(2,4,6-OMePh)$_3$	0.8	15	19	2	0
28	DCE	P(p-ClPh)$_3$	0.8	15	98	75	8
29	DCE	P(p-CF$_3$Ph)$_3$	0.8	15	11	0	0
30	DCE	P(m-MePh)$_3$	0.8	15	97	65	8
31	DCE	OPPh$_3$	0.8	15	11	0	0
32	DCE	PPhCy$_2$	0.8	5	11	5	0
33	DCE	PPh$_3$	0.8	0.1	100	66	7
34	DCE	PPh$_3$	0.8	5	97	67	8
35	DCE	PPh$_3$	0.8	10	93	66	8
36	DCE	PPh$_3$	0.8	100	63	40	6

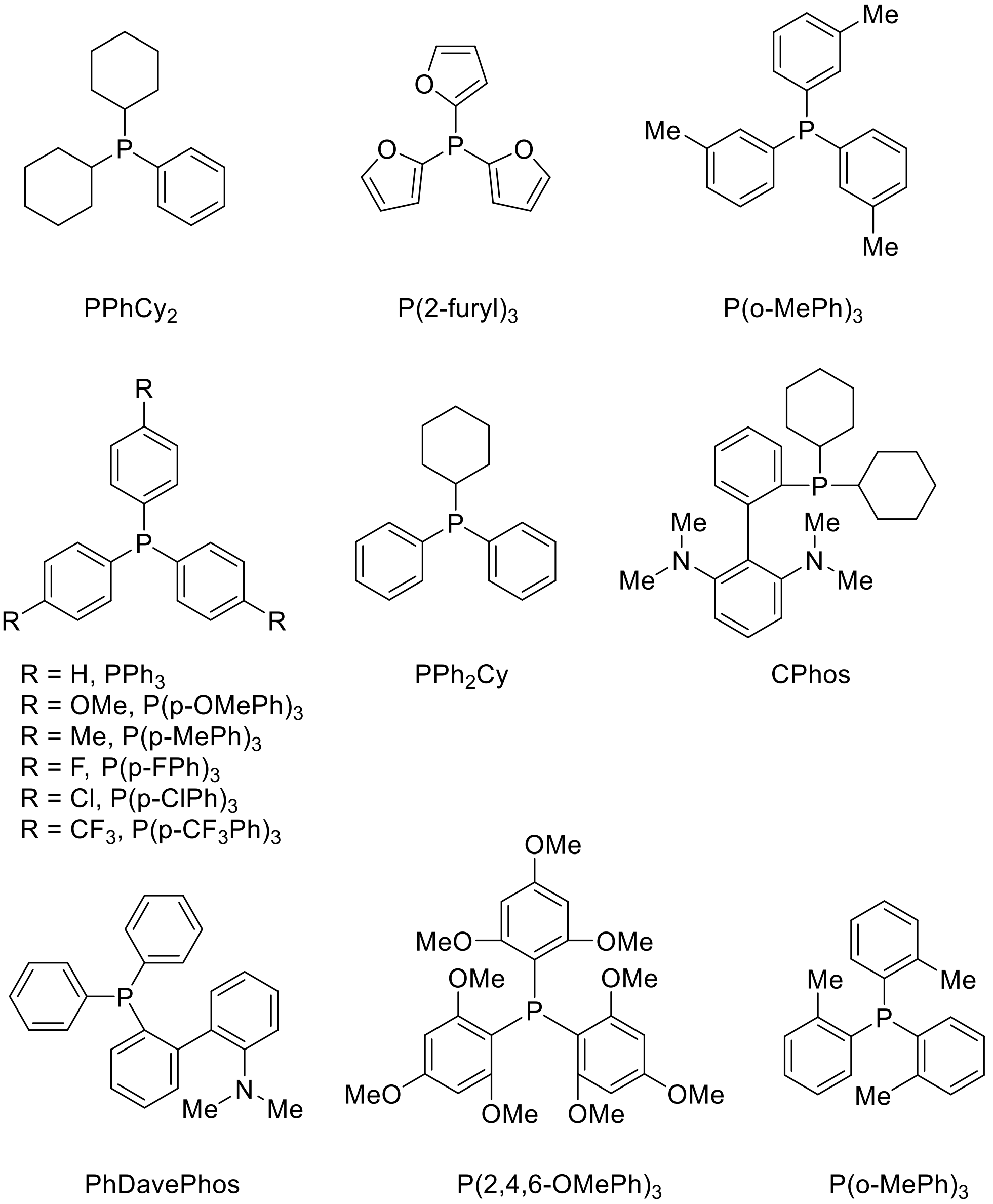

[a] Data were obtained following the **GP-S4** in air (80 °C, 16 h).

Table S11. Investigation of basic additives in the phosphine-promoted arylation of indole **1** with Pd@C_3N_4.

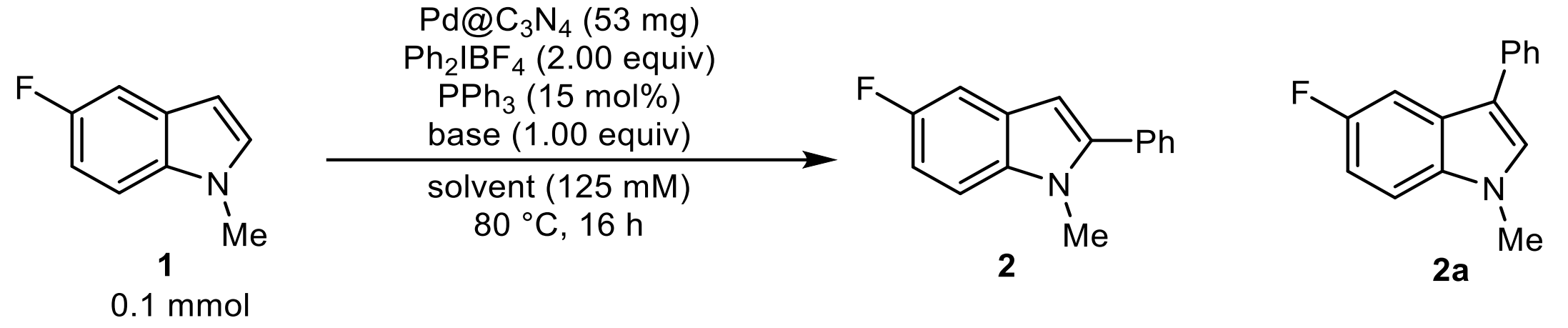

Entry[a]	Base[b]	Phosphine[c]	X_1 / %	Y_2 / %	Y_{2a} / %
1	DBU	PPh_3	23	0	0
2	TBD	PPh_3	16	0	0
3	TMG	PPh_3	21	0	0
4	P1-*t*-Bu	PPh_3	20	0	0
5	DABCO	PPh_3	6	0	0
6	dtbpy	PPh_3	37	16	7
7	Na_2CO_3	PPh_3	61	1	0
8	0.2 mL of sat. aq. K_2CO_3	PPh_3	17	0	0
9	0.2 mL of 1% aq. NaOH	PPh_3	24	0	0
10	4-Medtbpy	PPh_3	23	5	2
11	$NaHCO_3$	PPh_3	33	0	0
12	NaH_2PO_4	PPh_3	22	7	2
13	KOAc	PPh_3	25	0	0

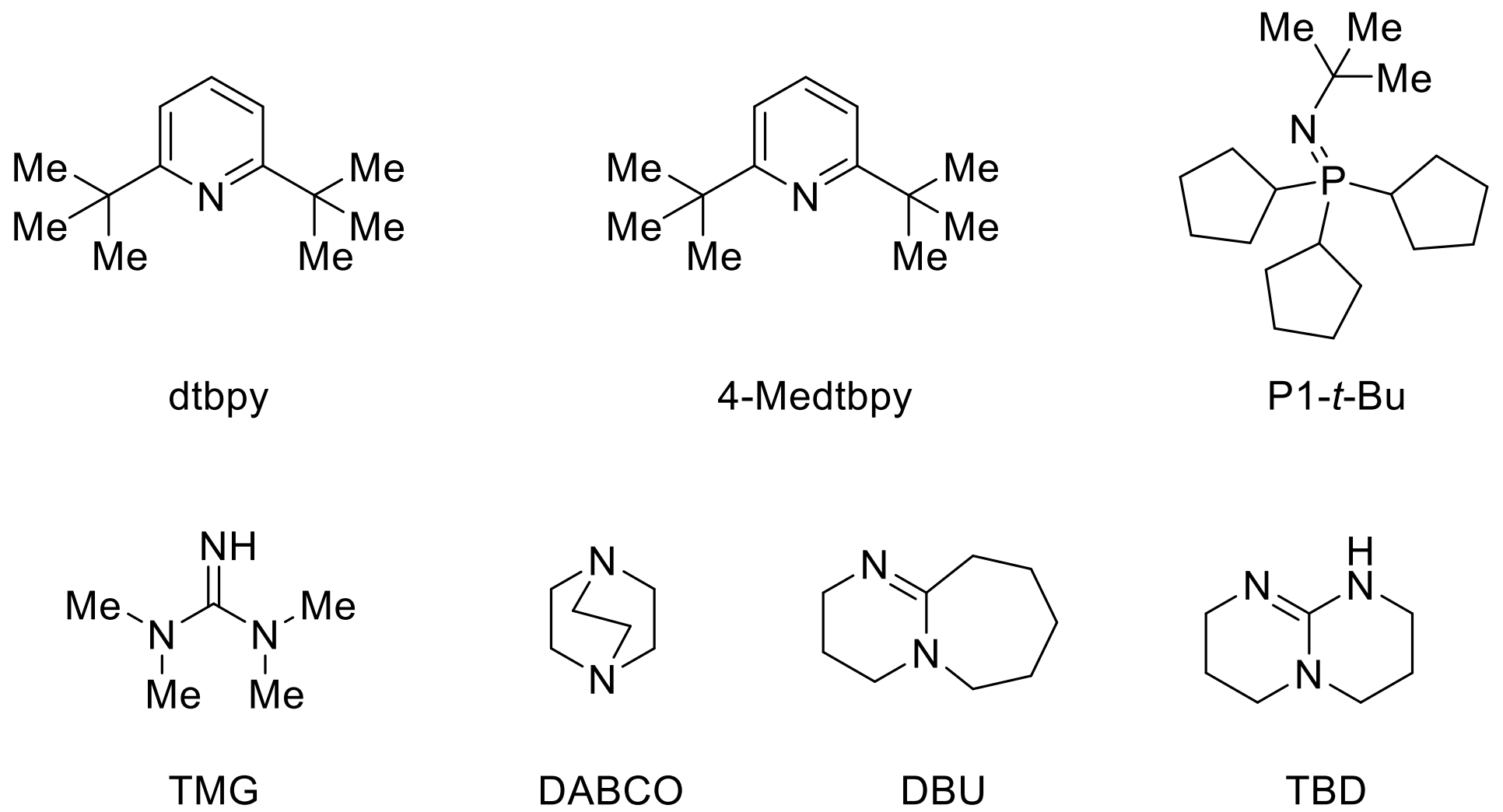

[a] Data were obtained following the **GP-S4** in air (DCE, 80 °C, 16 h). [b] 1 equiv. [c] 15 mol%.

Table S12. Investigation of different iodonium salt counterions for the phosphine-promoted arylation of indole **1** with Pd@C_3N_4.

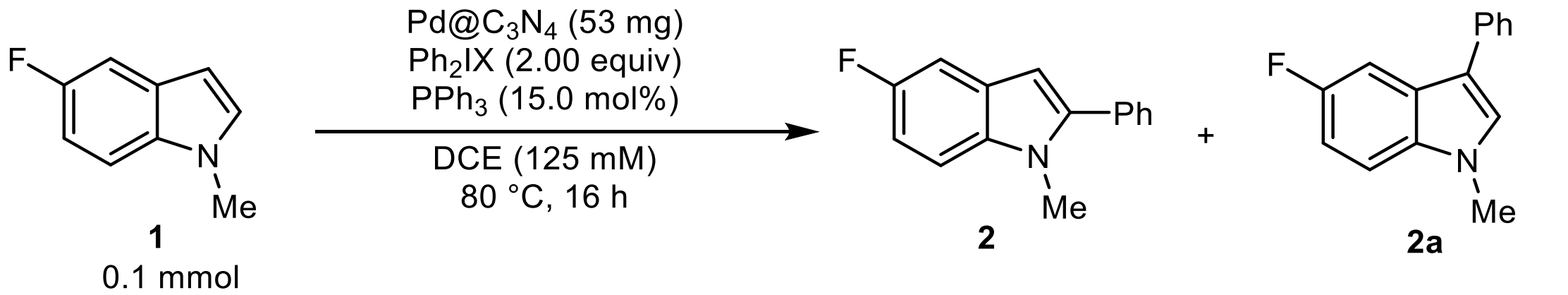

Entry[a]	Iodonium Salt	Phosphine[b]	X_1 / %	Y_2 / %	Y_{2a} / %
1	Ph_2IBF_4	PPh_3	93(4)	62(5)	8.7(4)
2	Ph_2IOTf	PPh_3	71	37	4
3	Ph_2IPF_6	PPh_3	99	80	10
4	Ph_2ICl	PPh_3	9	0	0

[a] Data were obtained following the **GP-S4** in air (80 °C, 16 h, DCE). [b] 15 mol%.

Table S13. Investigation of the effect of the Pd precursor used during $Pd@C_3N_4$ synthesis for the phosphine-promoted arylation of indole **1**.

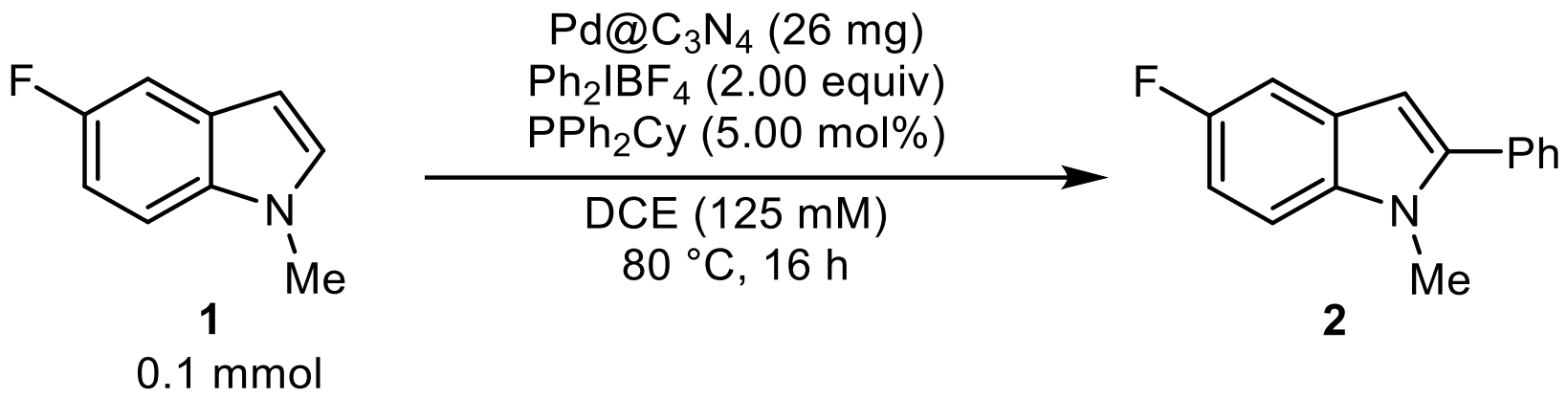

Entry[a]	Metal precursor in $Pd@C_3N_4$ synthesis	Phosphine[b]	X_1 / %	Y_2 / %
1	$(NH_3)_4Pd(NO_3)_2$	PPh_2Cy	100(0)	73.5(1.5)
2	$Pd(NO_3)_2$	PPh_2Cy	98	55
3	$Pd(SO_4)_2$	PPh_2Cy	75	33
4	$(MeCN)_4Pd(BF_4)_2$	PPh_2Cy	98	53
5	PdI_2	PPh_2Cy	40	22

[a] Data were obtained following the **GP-S4** in air (80 °C, 16 h, DCE).

Table S14. ICP-MS results of indole **1** C-H arylation reactions with $Pd@C_3N_4$ with different iodonium salts.

Entry[a]	Iodonium salt	Phosphine	Leached Pd / µg	Pd loss[b] / ppm
1[c]	Ph_2IBF_4	PPh_3[e]	0.05	181
2[c]	Ph_2IPF_6	PPh_3[e]	1.31	4753
3[d]	Ph_2IBF_4	PPh_2Cy[f]	below LOD[g]	< 15

[a] Data were obtained following the **GP-S4** in air (80 °C, 16 h, DCE). [b] Pd loss was calculated with respect to the amount of Pd added to the reaction, i.e. the metal content determined by ICP-OES multiplied by the catalyst mass. [c] 0.1 mmol scale, 53 mg $Pd@C_3N_4$. [d] 0.1 mmol scale, 26 mg $Pd@C_3N_4$. [e] 15 mol%. [f] 5 mol%. [g] Limit of detection = 2 ng.

Table S15. Percentage composition of the mixtures obtained via the modified hot filtration with $Pd@C_3N_4$ in the C2-arylation of indole **1** in DCE with different ligands.

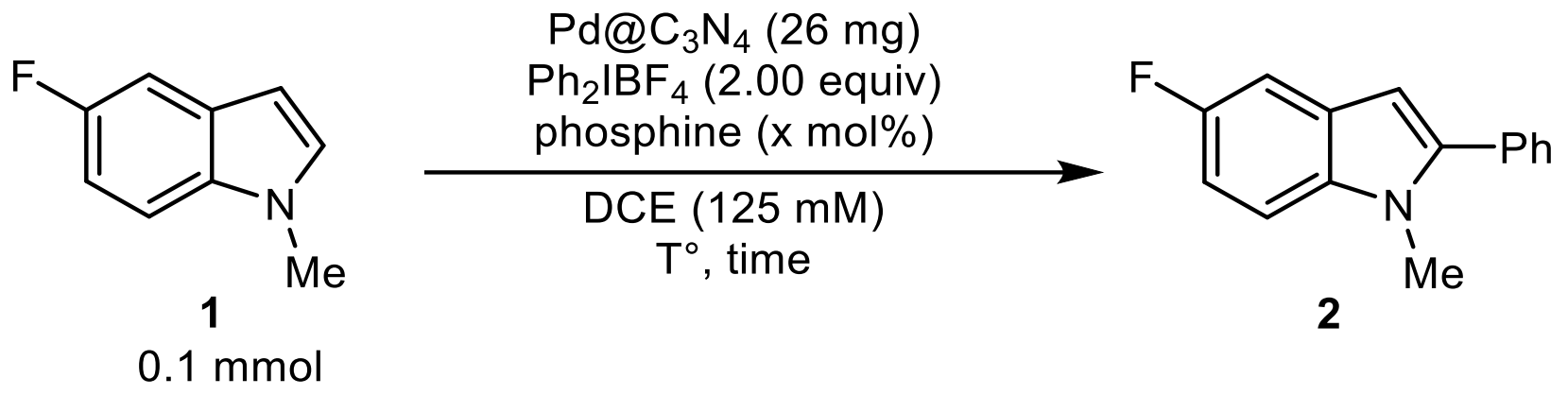

Entry[a]	Phosphine	Phosphine amount / mol%	Time / h	*T* / °C	Y_2 / %
1	PPh_3	15	16[b]	60	40
1-HF		0	16[c]		40
2	PPh_3[d]	15	16[b]	80	62
2-HF		15	16[c]		58
3	PPh_3	15	16[b]	80	62
3-HF		0	16[c]		62
4	PPh_2Cy[d]	5	16[b]	80	79
4-HF		5	16[c]		78
5	PPh_2Cy	5	16[b]	80	74
5-HF		0	16[c]		75

[a] Data were obtained following the **GP-S3**. [b] Before filtration. [c] After filtration. [d] Additional phosphine added after filtration.

Scope of single-atom-catalyzed C2-arylation

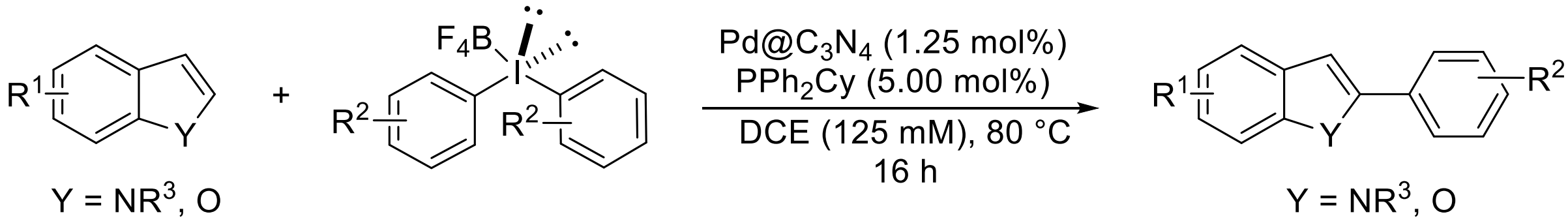

General procedure S6 (**GP-S6**) for single-atom-catalyzed C2-arylation. An oven-dried vial was charged with a heteroarene (200 µmol, 1.00 equiv), iodonium salt (400 µmol, 2.00 equiv), Pd@C_3N_4 (53.2 mg, 2.50 µmol, 1.25 mol%), PPh_2Cy (2.68 mg, 10.0 µmol, 5.00 mol%), and DCE (1.60 mL) under air. The vial was then sealed and stirred (1000 rpm) on a preheated hotplate at 80 °C for 16 h. The reaction mixture was then filtered through a SiO_2 plug (1 g), eluting with DCM, and concentrated under reduced pressure. The crude residue was purified by FCC to afford the arylated compound.

Glassware used: Biotage Microwave Reaction Kit, 0.5-2 mL (cat. no. 352016).

C2/C3 ratios in crude mixture were determined by ^{1}H or ^{19}F NMR. Attempts to isolate the minor regioisomer were generally unsuccessful.

Table S16. Performance comparison of the reported homogeneous IMesPd$(OAc)_2$ system from reference[19] with the Pd@C_3N_4 system developed in this study.[a]

Substrate	Arylating agent	*T*/ °C	*TON*[b]	*TOF*[c] / h^{-1}	Source
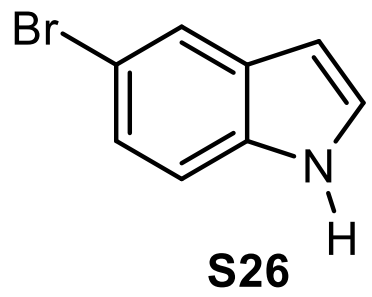 **S26**	Ph_2IBF_4	60	15	1.0	Reference[19]
		80	58	3.7	This work
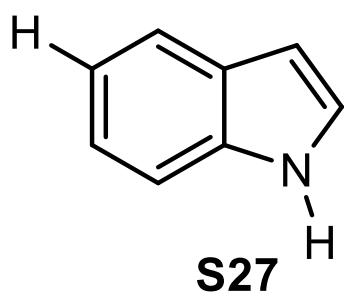 **S27**	Ph_2IBF_4	25	16	1.0	Reference[19]
		80	39	2.5	This work
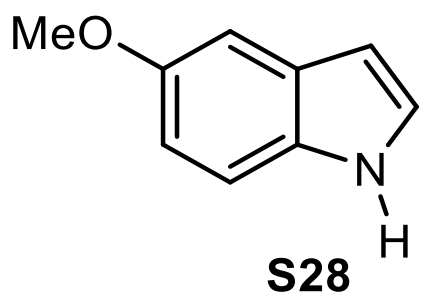 **S28**	Ph_2IBF_4	25	12	0.8	Reference[19]
		60	31[d]	2.0	This work
		80	25[d]	1.6	This work

[a] Data were obtained following the **GP-S6**. [b] Turnover number. [c] Average turnover frequency. [d] Based on NMR yield.

KIE measurements

Due to the autocatalytic profile of the reaction, extracting rate constants proved challenging. Thus, intermolecular competition experiments were chosen to determine the KIEs at the C2 position of indole. Accurate determination of KIE values at the C3 position was not possible due to a fast H/D exchange under acidic reaction conditions.

Entries 1-3. An oven dried vial was charged with stock solution (0.50 mL) containing **S12** and ***d*-S12** (100 μmol total, 1.00 equiv) and 1-fluoronaphthalene (14.6 mg, 12.9 μL, 100 μmol, 1.00 equiv) in DCE (0.50 mL), Ph_2IBF_4 (73.6 mg, 200 μmol, 2.00 equiv), Pd@C_3N_4 (26.6 mg, 1.25 μmol, 1.25 mol%), PPh_2Cy (1.34 mg, 5.00 μmol, 5.00 mol%), and DCE (0.30 mL) under air. The vial was then sealed and stirred (1000 rpm) on a preheated hotplate at 80 °C. An aliquot was taken from the reaction mixture, diluted with DCM and d_6-DMSO, and analyzed by ^{19}F qNMR to determine conversion (F). ***d*-S12**/**S12** ratios (R_0 and R) were determined by HRMS. The measurements were performed in duplicate using a Waters Xevo G2-S QTOF mass spectrometer. The instrument was operated in positive ionization mode using the ZSpray ESI source. The TOF spectra were acquired in sensitivity mode, and leucine enkephalin was used as a lock mass to improve mass accuracy. For data processing, the spectra were analyzed using the deconvolution tool available in ChemCalc. The analysis was focused on determining the ratio between the two isotopic peaks of interest.

Entry 4. The reaction was prepared according to **GP-S6** using a desired ratio of **S12** and ***d*-S12** (total 200 μmol) and run for a desired amount of time. The reaction mixture was then filtered through a SiO_2 plug (1 g), eluting with DCM, and concentrated under reduced pressure. The conversion was determined by ^{1}H qNMR with CH_2Br_2 as an internal standard in $CDCl_3$. The crude residue was purified by FCC (SiO_2, 2-10% EtOAc/hexane) to afford the remaining indole starting material. ***d*-S12**/**S12** ratios (R_0 and R) were determined by ^{1}H qNMR in d_6-DMSO.

See **Note S9** for the result interpretation.

$$R = \frac{[\boldsymbol{dS12}]}{[\boldsymbol{S12}]} \tag{S1}$$

$$\frac{k_H}{k_D} = \frac{\ln\left((1-F)\left(\frac{1+R_0}{1+R}\right)\right)}{\ln\left((1-F)\left(\frac{R}{R_0}\right)\left(\frac{1+R_0}{1+R}\right)\right)} \tag{S2}$$

Table S17. Results of intermolecular competition experiments used to determine the kinetic isotope effect (KIE).

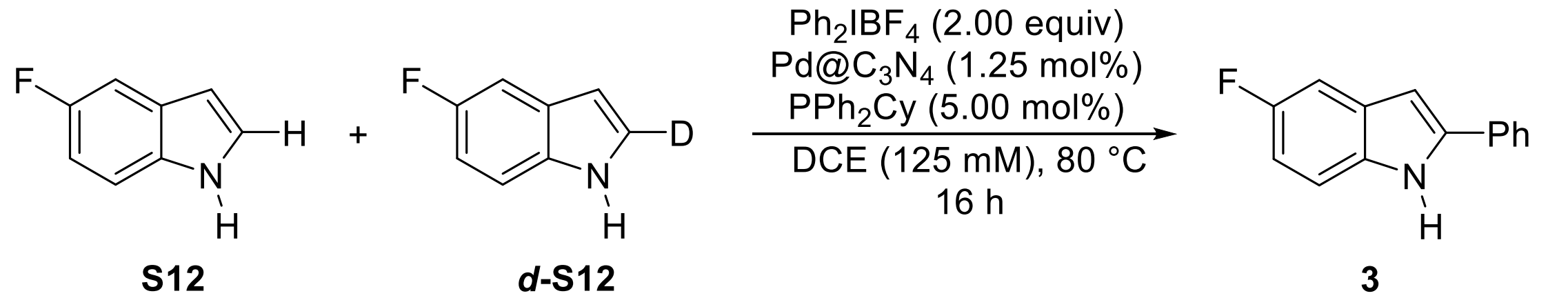

Entry	R_0	R	F	k_H/k_D
1	0.965(3)	0.678(17)	0.88	0.848
2	0.965(3)	0.622(9)	0.92	0.842
3	0.965(3)	0.672(59)	0.90	0.856
4	1.041	0.906	0.61	0.863
Average	-	-	-	**0.852(9)**

Kinetic data and mechanistic insights

Table S18. Kinetic plots and data of the phosphine-promoted C2 arylation of indole **1** with $Pd@C_3N_4$, with or without an acid additive.

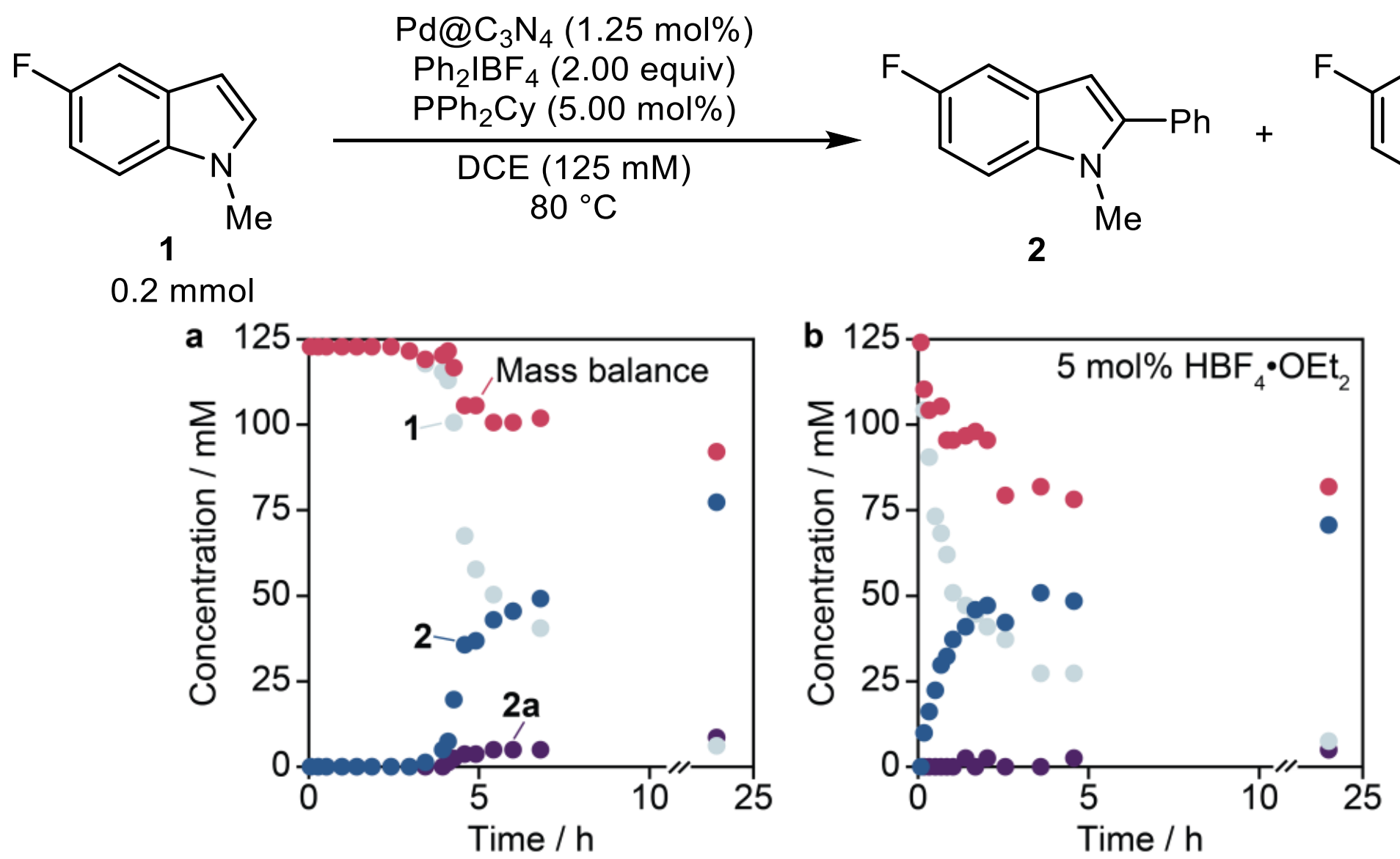

Time / h	Additive	1 / mM	2 / mM	2a / mM	Mass balance / mM
0.06		123.20	0.00	0.00	123.20
0.28		123.20	0.00	0.00	123.20
0.53		123.20	0.00	0.00	123.20
1.00		123.20	0.00	0.00	123.20
1.42		123.20	0.00	0.00	123.20
1.87		123.20	0.00	0.00	123.20
2.43		123.20	0.00	0.00	123.20
2.96		121.96	0.00	0.00	121.96
3.43	none	118.27	1.23	0.00	119.50
3.93		115.80	4.93	0.00	120.73
4.09		113.34	7.39	1.23	121.96
4.26		101.02	19.71	2.46	117.04
4.58		67.76	35.73	3.70	105.95
4.91		57.90	36.96	3.70	105.95
5.43		50.51	43.12	4.93	101.02
6.00		45.58	45.58	4.93	101.02
6.80		40.65	49.28	4.93	102.25

23.93		6.16	77.61	8.62	92.40
0.08		124.44	0.00	0.00	124.44
0.18		104.53	9.95	0.00	110.75
0.33		90.84	16.18	0.00	104.53
0.50		73.42	22.40	0.00	88.35
0.67		68.44	29.86	0.00	105.77
0.84		62.22	32.35	0.00	95.82
1.02	5 mol% HBF_4•OEt_2 added initially	51.02	37.33	0.00	95.82
1.40		47.29	41.06	2.49	97.06
1.69		44.80	46.04	0.00	98.30
2.03		41.06	47.29	2.49	95.82
2.56		37.33	42.31	0.00	79.64
3.59		27.38	51.02	0.00	82.13
4.58		27.38	48.53	2.49	78.40
23.53		7.47	70.93	4.98	82.13

The data were obtained following **GP-S6** (scale: 0.2 mmol) with 1-fluoronaphthalene (1 equiv) internal standard added from the beginning of the reaction and HBF_4•OEt_2 (5 mol%) added where specified. The sampled aliquots were immediately diluted with DCM (3 mL), filtered through a syringe filter (0.22 μm), concentrated under reduced pressure, and diluted with d_6-DMSO before ^{19}F qNMR analysis.

Table S19. Summary of varying standard conditions to probe the reaction mechanism.

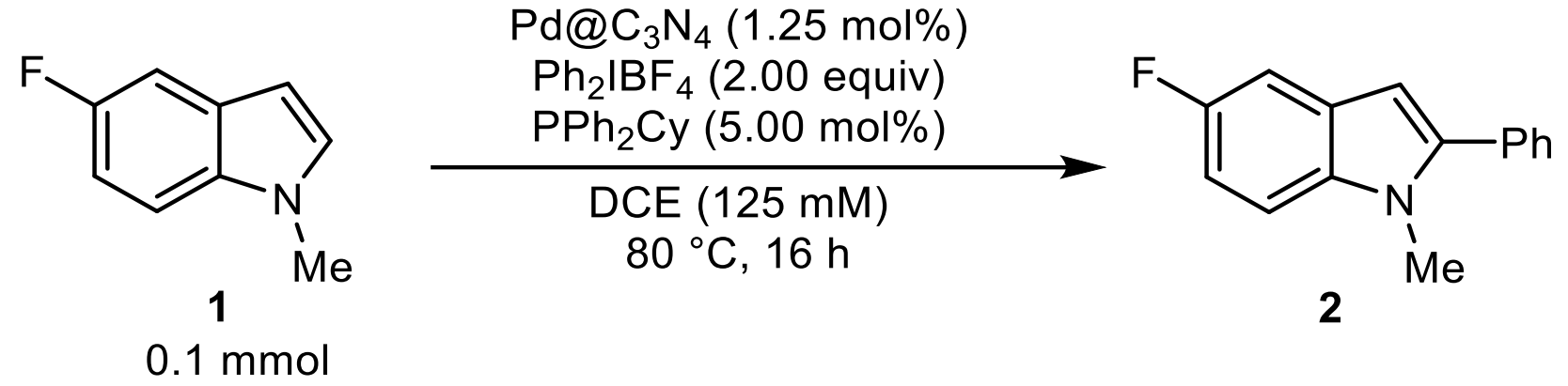

Entry	Deviation from standard conditions	X_1 / %	Y_2 / %
1	none	100(0)	73.5(1.5)
2	Additional TBAI (1 equiv)	48	n.d.
3	Additional TBABr (5 mol%)	13	8
4	$TBABF_4$ (20 mol%) instead of PPh_2Cy	11	n.d.
5	PPh_3CyBF_4 (2 equiv) instead of PPh_2Cy and Ph_2IBF_4	9	n.d.
6	PPh_3CyBr (5 mol%) instead of PPh_2Cy	0	n.d.
7	PPh_3CyBF_4 (5 mol%) instead of PPh_2Cy	85	37
8	PPh_3 (0.1 mol%) instead of PPh_2Cy	100	66
9	Reaction setup in N_2 glovebox instead of under air	90	49
10	Reaction setup in N_2 glovebox instead of under air with additional H_2O (1.80 µL, 0.1 mmol, 1.0 equiv)	83	56
11	BF_3•OEt_2 (5 mol%) instead of PPh_2Cy	90	44
12	$Pd(OAc)_2$ (5 mol%) instead of $Pd@C_3N_4$	100	4
13	Additional PhI (1 equiv)	93	47

[a] Data were obtained following the **GP-S6** with PPh_2Cy (5 mol%) or modifications as indicated in the table. Scale: 0.1 mmol.

Exploration of Lewis acids as alternatives to phosphine

Table S20. Study of Lewis acids as potential alternatives to phosphine.

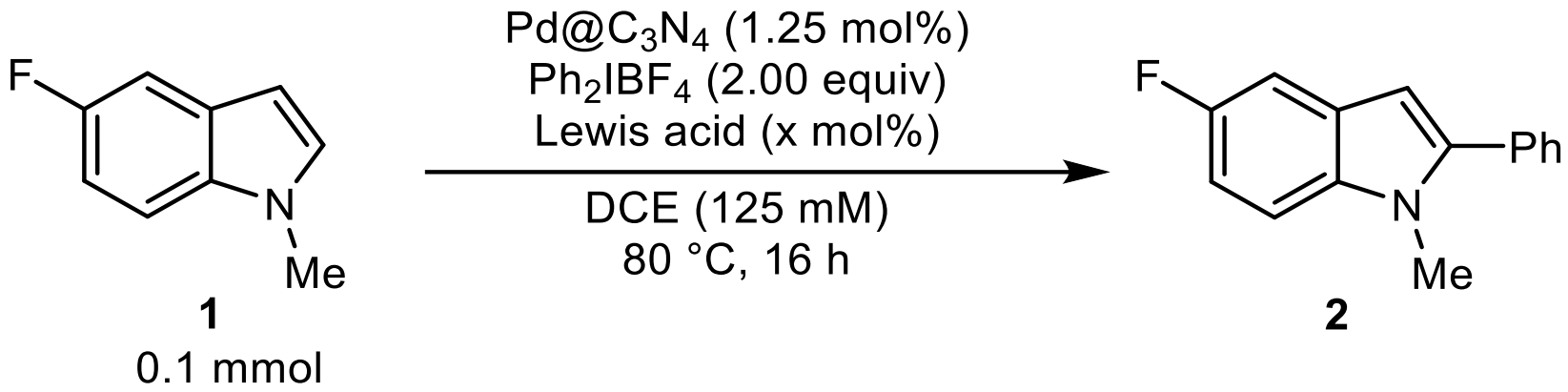

Entry	Lewis acid	Amount / mol%	X_1 / %	Y_2 / %
1	BF_3•OEt_2	5 mol%	90	44
2	$Zn(OTf)_2$	5 mol%	100	41
3	$AlCl_3$	5 mol%	93	21
4	$TiCl_4$	5 mol%	100	n.d.
5	TMSCl	5 mol%	100	n.d.
6	$Sc(OTf)_3$	5 mol%	100	32
7	$LaCl_3$	5 mol%	92	22
8	BF_3•OEt_2	10 mol%	95	50
9	BF_3•OEt_2	1 mol%	92	33
10	BF_3•OEt_2	0.1 mol%	18	n.d.

[a] Data were obtained following the **GP-S6**, using the Lewis acids indicated instead of PPh_2Cy. Scale: 0.1 mmol.

Attempts to prevent iodide poisoning in situ

Table S21. Summary of attempts to avoid iodide poisoning in situ by altering standard reaction conditions.

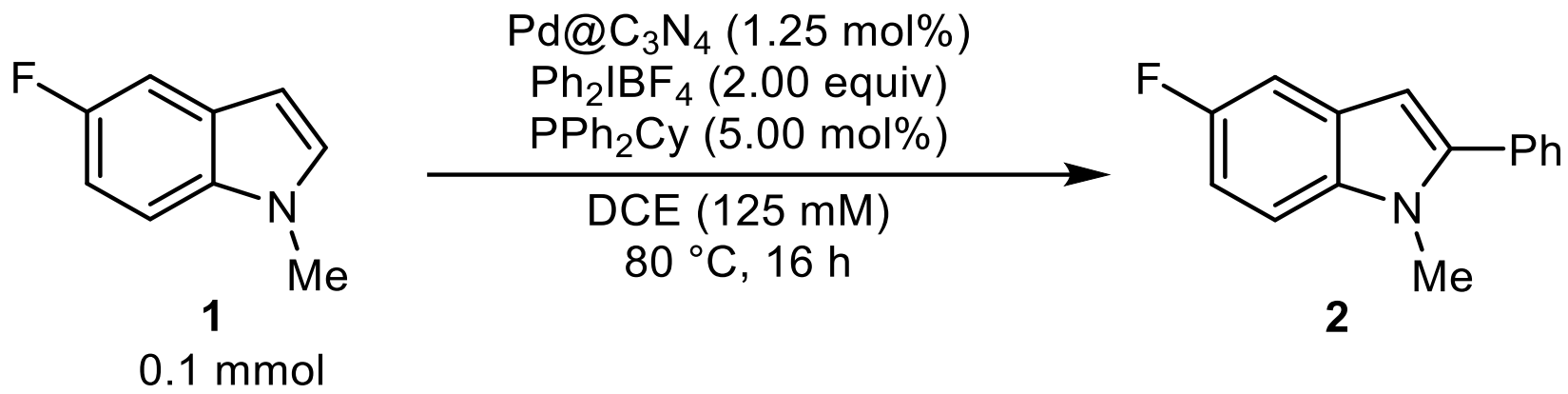

Entry	Phosphine	Solvent	Deviation from standard conditions	Y_2 / %
1	PPh_2Cy	DCE	none	72.5(1.4)
2	PPh_2Cy	DCE	Additional AgOTf (1 equiv)	9
3	PPh_2Cy	DCE	$MesIPhBF_4$ instead of Ph_2IBF_4	8
4	PPh_2Cy	DCE	PhOTf instead of Ph_2IBF_4	n.d.
5	PPh_2Cy	DCE	PhN_2OTf instead of Ph_2IBF_4	n.d.

[a] Data were obtained following the **GP-S6** in air (0.100 mmol, 80 °C, 16 h) with modifications as indicated in the table.

In situ XANES and EXAFS fitting parameters

Table S22. XANES linear component fitting parameters from in situ measurements of Pd@C_3N_4 under optimized conditions. The fit was performed for the normalized XANES spectra in the 24335-24405 eV region.

System	Fitting model[a]	Contribution / %				R-factor / %
		Pd-N[b]	Pd-Pd[c]	Pd-I[d]	Pd-O[e]	
IPh_2BF_4 200 min	1	100.0(3)	0.0(3)	-	-	0.0076
	2	100.0(1.4)	0.0(5)	0.0(1.3)	-	0.0076
	3	100.0(1.0)	0.0(3)	-	0.0(9)	0.0076
Reaction 10 min	1	100.0(4)	0.0	-	-	0.015
	2	100(2)	0.0(7)	0(2)	-	0.015
	3	94.8(1.3)	0.0(4)	-	5.2(1.2)	0.013
Reaction 76 min	1	98.9(1.2)	1.1(3)	-	-	0.0057
	2	99.1(1.4)	0.0	0.9(7)	-	0.0064
	3	100.0(1.5)	0.0	-	0.0(9)	
Reaction 142 min	1	95.1(4)	4.9(6)	-	-	0.015
	2	83.8(7)	0.0	16.2(7)	-	0.0057
	3	95.5(1.4)	4.5(2)	-	0.0(1.3)	0.015

[a] Fitting model 1 considers only Pd-N and Pd-Pd components, while models 2 and 3 additionally include a Pd-I or Pd-O contribution. The following references were used: [b] Pd@C_3N_4 in DCE, [c] Pd foil, [d] $(py)_2PdI_2$, [e] $Pd(OAc)_2$.

Table S23. EXAFS fitting parameters from in situ measurements of Pd@C_3N_4 in DCE, in DCE with Ph_2IBF_4 and under optimized reaction conditions (**GP-S4**) at different time points. The fit was performed using $k^{1,2,3}$-weighted data, Fourier-transformed in 3–10 Å^{-1} k-range. Reference materials were fitted using reference crystal structures with coordination number (N), zero-potential correction (ΔE_0), interatomic distance (R), and Debye-Waller parameter (σ^2). The dampening (S_0^2) was fixed at 0.8 based on the value of the foil.

System	Path	ΔE_0 / eV	R / Å	N / -	σ^2 / Å^2	R-factor / %
DCE	Pd-N	-6(4)[b]	1.96(5)	2[c]	0.005(6)	1.13
	Pd-Cl[a]	-6(4)[b]	2.18(3)	2[c]	0.006(3)	
Ph_2IBF_4 200 min	Pd-N	-9(3)	1.94(4)	2.5[c]	0.006(2)	2.09
	Pd-Cl[a]	-9(3)	2.16(2)	1.5[c]	0.003(2)	
Reaction 10 min	Pd-N	-3(6)[b]	1.97(6)	3[c]	0.004(4)	1.70
	Pd-Cl[a]	-3(6)[b]	2.19(4)	1[c]	0.001(5)	
Reaction 70 min	Pd-N	-7(7)[b]	1.9(1)	1.5	0.007(7)	1.06
	Pd-Cl[a]	-7(7)[b]	2.17(4)	2	0.003(3)	
	Pd-I	-7(7)[b]	2.64(5)	0.5	0.000(4)	
Reaction 142 min	Pd-N	-6(4)[b]	1.94(4)	1.5[c]	0.001(4)	1.71
	Pd-Cl[a]	-6(4)[b]	2.19(3)	1.9[c]	0.006(3)	
	Pd-I	-6(4)[b]	2.62(4)	0.6[c]	0.004(5)	

[a] The Pd-Cl path was used to model the double contribution observed in the pure Pd@C_3N_4 (**Table S4**) and does not imply the existence of Pd-Cl. This path is highly correlated with Pd-N, and coordination values should not be compared individually. [b] Grouped variable. [c] Total coordination number constrained to 4. [d] Fixed value.

Pd@C_3N_4 recycling with ex situ catalyst washes

General procedure S5 (**GP-S5**) for Pd@C_3N_4 recycling with catalyst washes. The reaction set-up is modified from **GP-S4** in the following ways: 0.2 mmol scale, 5 mol% phosphine, air atmosphere. After 16 h of reaction, the mixture was diluted with DCM (5 mL) and centrifuged to collect the solid catalyst. The catalyst was then washed with DCM (3 x 5 mL), the desired washing solution (1 x 5 mL), water (2 x 5 mL), and dried at 200 °C for 16 h. The reaction cycle was repeated as many times as desired. The reaction scale was adjusted to the amount of recovered catalyst.

Table S24. Investigation of different washing solutions for recycling Pd@C_3N_4 after the C-H arylation of indole **1**.

Entry[a]	Washing solution	Y_2 / %				
		**Cycle 1**	**Cycle 2**	**Cycle 3**	**Cycle 4**	**Cycle 5**
1	None	70(4)	12	-	-	-
2	1% aq. NaOH	70(4)	18	-	-	-
3	1% aq. NaOH wash of fresh catalyst	6	-	-	-	-
4	10% aq. $Na_2S_2O_3$	70(4)	n.d.	-	-	-
5	Sat. AgOTf in DCM	70(4)	22	-	-	-
6	25% aq. NH_3	70(4)	53	3	-	-
7	10% aq. NH_3	70(4)	58	-	-	-
8[c]	1% aq. NH_3 [b]	60	76	71	2	70[c]
9	1% aq. NH_3	70(4)	71	46	3	55[c]
10	0.1% aq. NH_3	70(4)	6	-	-	-

[a] Data were obtained following the **GP-S5**. Yields were determined by ^{19}F qNMR with 1-fluoronaphthalene as an internal standard. Initial scale: 0.2 mmol. Then, the scale was adjusted according to the amount of recovered catalyst. [b] With PPh_3 instead of PPh_2Cy. [c] With 5 mol% $BF_3 \cdot OEt_2$. [d] Fluctuations in yield were completely reproducible across two independent runs.

Table S25. ICP-OES quantification of iodine in the used and washed catalysts.

Catalyst	Digestion mixture	I content / wt%	I concentration / ppm
Pd@C_3N_4-C1	25 wt% NaOH[a]	0.71	-
Pd@C_3N_4-washed[b]	25 wt% NaOH[a]	0.07	-
Wash solution	-	-	36.9[b]

[a] 25 wt% aqueous solution of NaOH. [b] 53 mg catalyst was washed with 5 mL of 1 wt% NH_3 solution. The wash solution was recovered and diluted to 20 mL with Milli-Q water and measured by ICP-OES.

Table S26. N_2 physisorption results of as-prepared and washed carbon nitride. The specific surface area S_{BET} was determined using the Brunauer-Emmett-Teller (BET) method. The pore volume V_{pore} was determined at a reduced pressure $p/p^0 = 0.98$.

Entry	Wash	S_{BET} / m^2 g^{-1}	V_{pore} / cm^3 g^{-1}	Loss S_{BET} / %	Loss V_{pore} / %
1	None	159	0.32	-	
2	1% NH_3	143	0.27	10	16
3	25% NH_3	145	0.26	9	19

Supplementary Figures

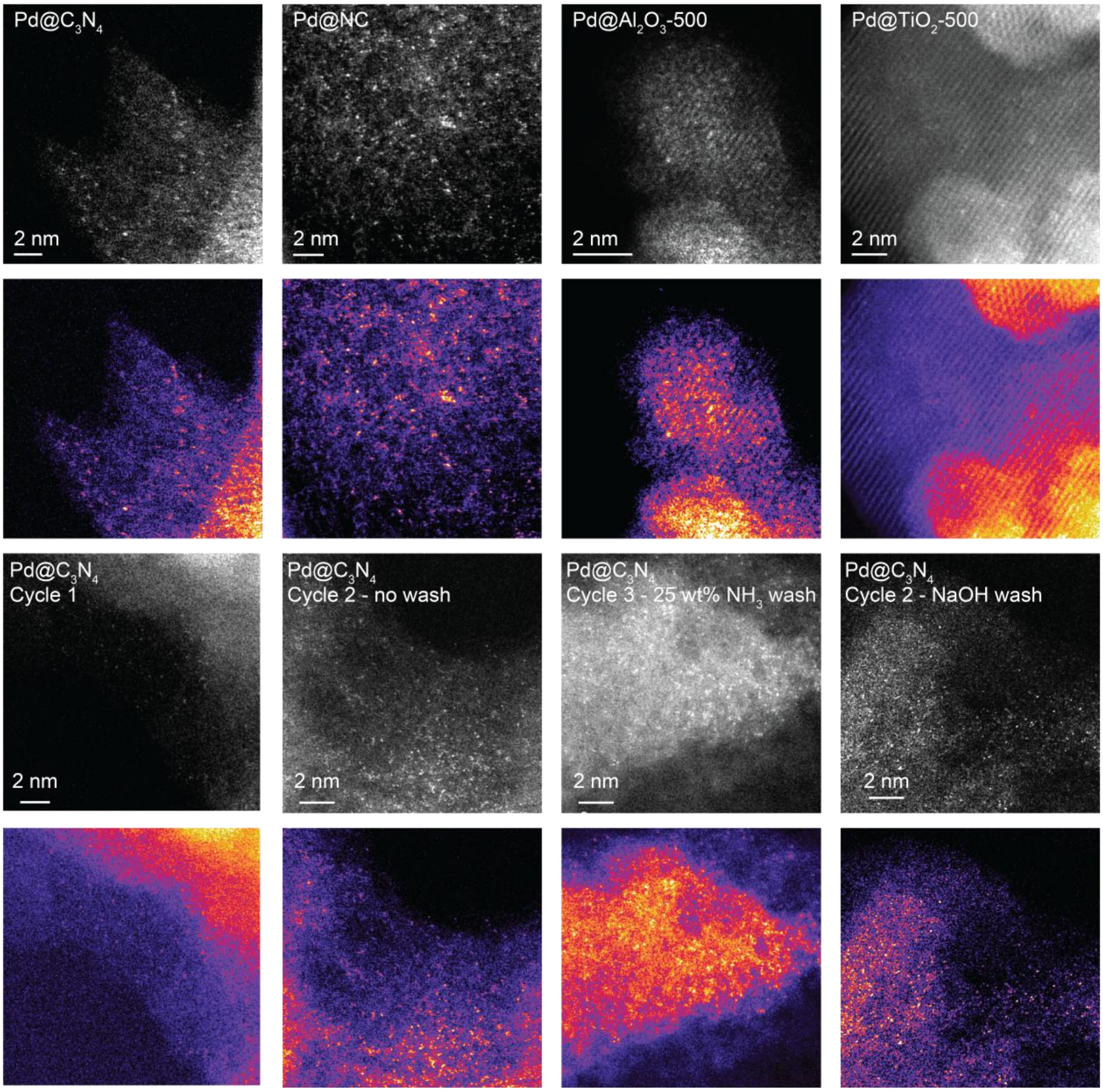

Figure S1. HAADF-STEM images of as-prepared and used $Pd@C_3N_4$ catalysts.

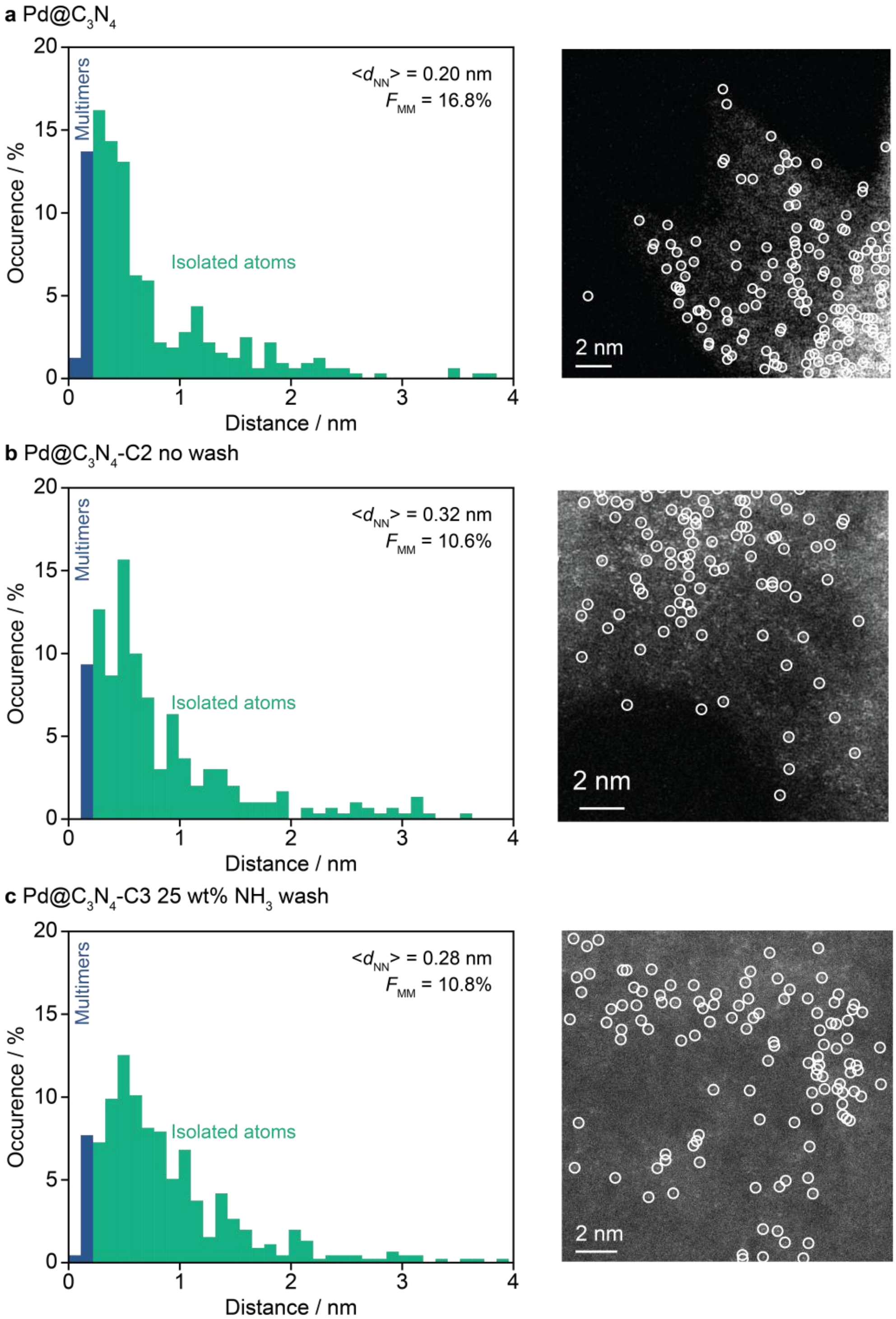

Figure S2. Nearest-neighbor analysis of Pd@C_3N_4 micrographs using an automated atom-detection tool. **(a)** Before reaction; **(b)** after two reaction cycles without washing; **(c)** after three reaction cycles with a 25 wt% NH_3 wash. The confidence threshold for atom detection was set to 0.8. A nearest-neighbor distance of 0.22 nm was used as the threshold for multimer formation in the *xy* plane. The multimer fraction, F_{MM}, therefore represents an upper-bound estimate

owing to projection effects and the statistical occurrence of close neighbors in a random distribution. All micrographs used for the analysis are available on Zenodo (record 20580576; DOI: 10.5281/zenodo.20580576).

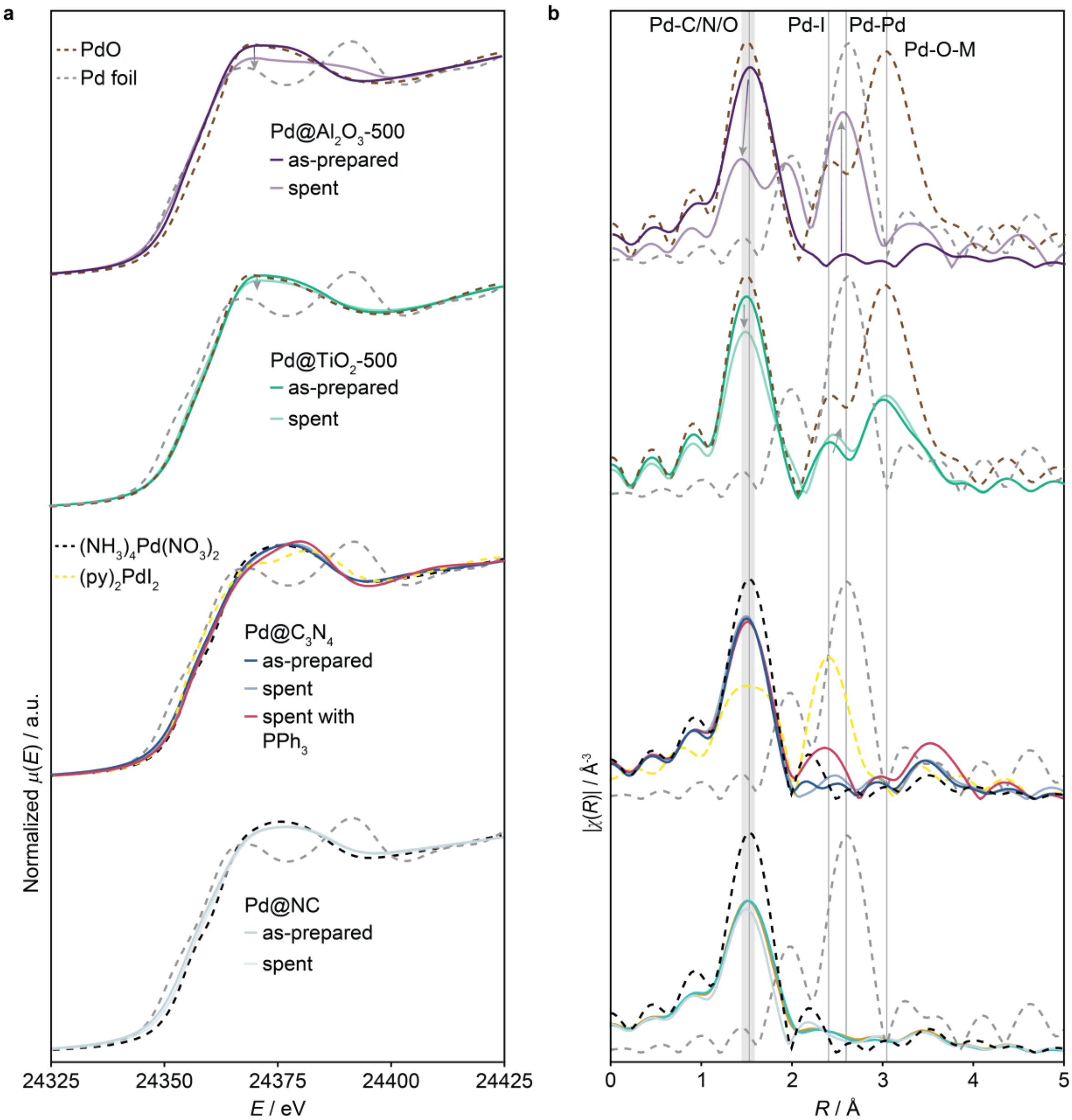

Figure S3. XANES (**a**) and EXAFS (**b**) of as-prepared and used catalysts tested in DCE with and without PPh_3 phosphine.

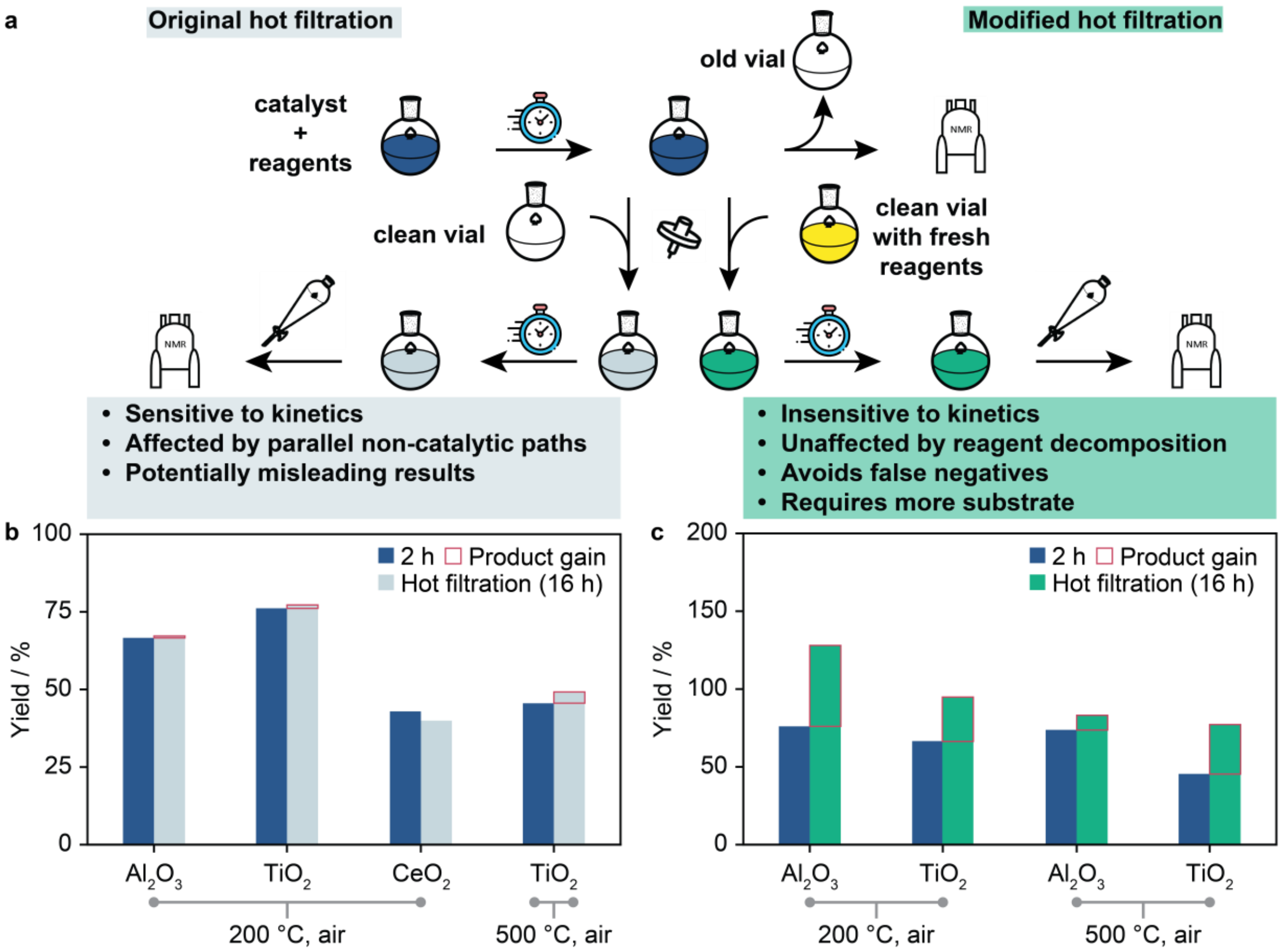

Figure S4. Schematic representation of the hot filtration procedure commonly used in literature (left) and the modified version introduced herein (right). Hot filtration results of representative catalysts using the old (**b**) and modified methodology (**c**).

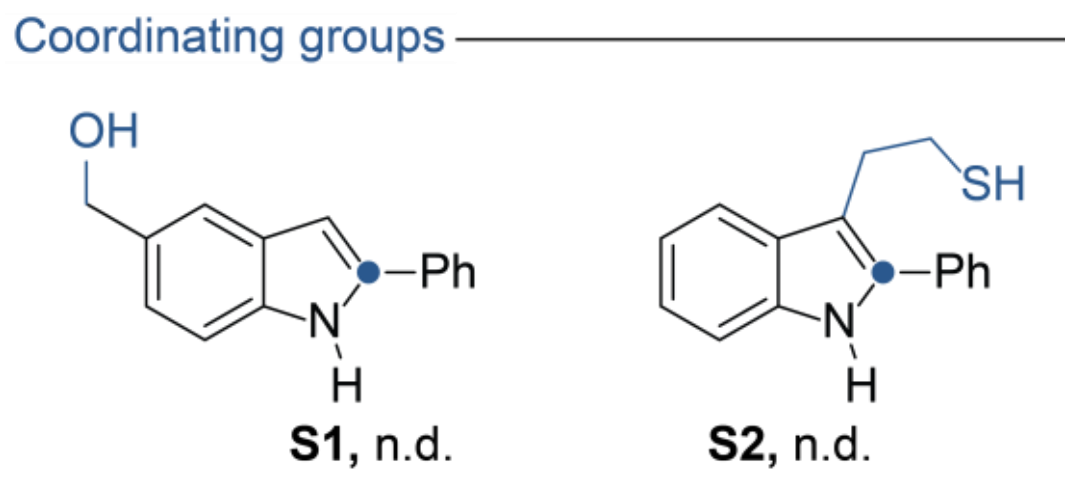

Figure S5. Scope limitations of the developed Pd single-atom-catalyzed C-H arylation of indoles.

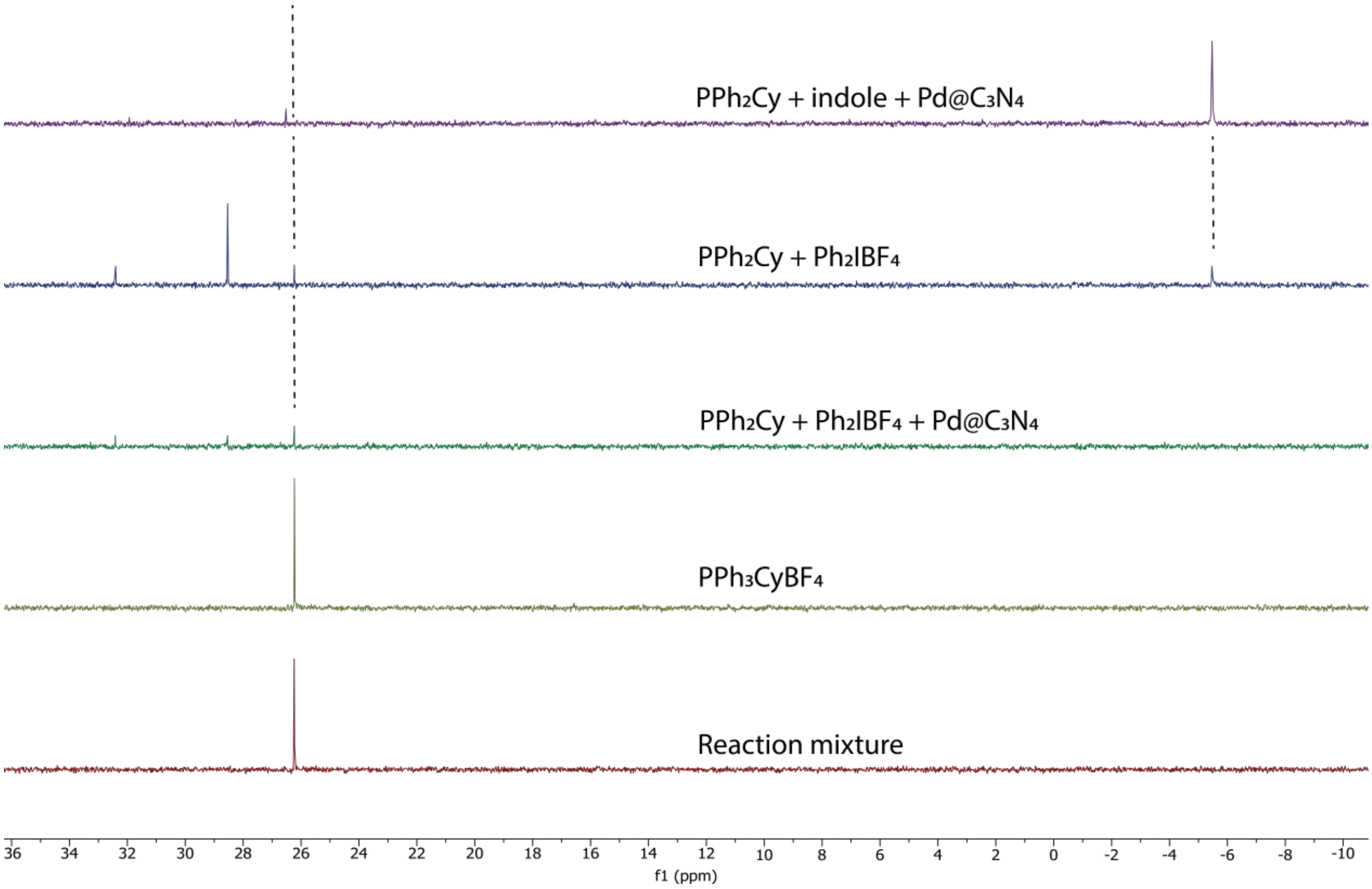

Figure S6. $^{31}P\{^{1}H\}$ NMR of various mixtures resulting from stirring reagents in DCE at 80 °C for 1 h. The spectra were recorded in d_6-DMSO. Data were obtained following **GP-S6** for the reaction mixture with filtration through a filter instead of a silica plug. Scale: 0.1 mmol.

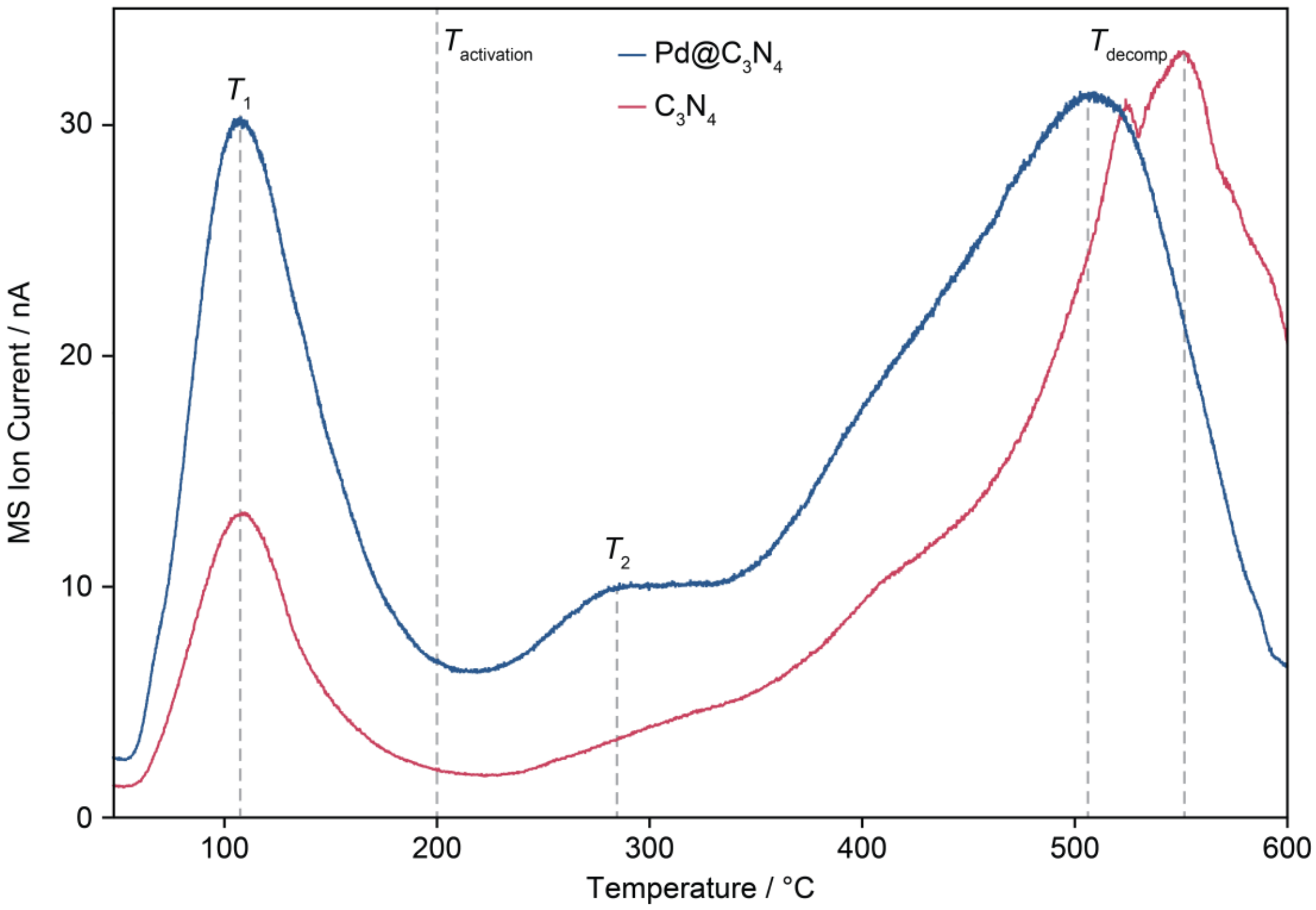

Figure S7. NH_3 temperature-programmed desorption profiles of as-prepared C_3N_4 and Pd@C_3N_4, monitored by electron-impact mass spectrometry at $m/z = 17$. The desorption temperatures T_1 and T_2 are indicated together with the thermal activation temperature ($T_{activation}$) used during synthesis and the decomposition temperature of carbon nitride (T_{decomp}). T_1 is tentatively assigned to NH_3 adsorbed on the carbon nitride surface, whereas T_2 is tentatively attributed to Pd-bound NH_3 species.

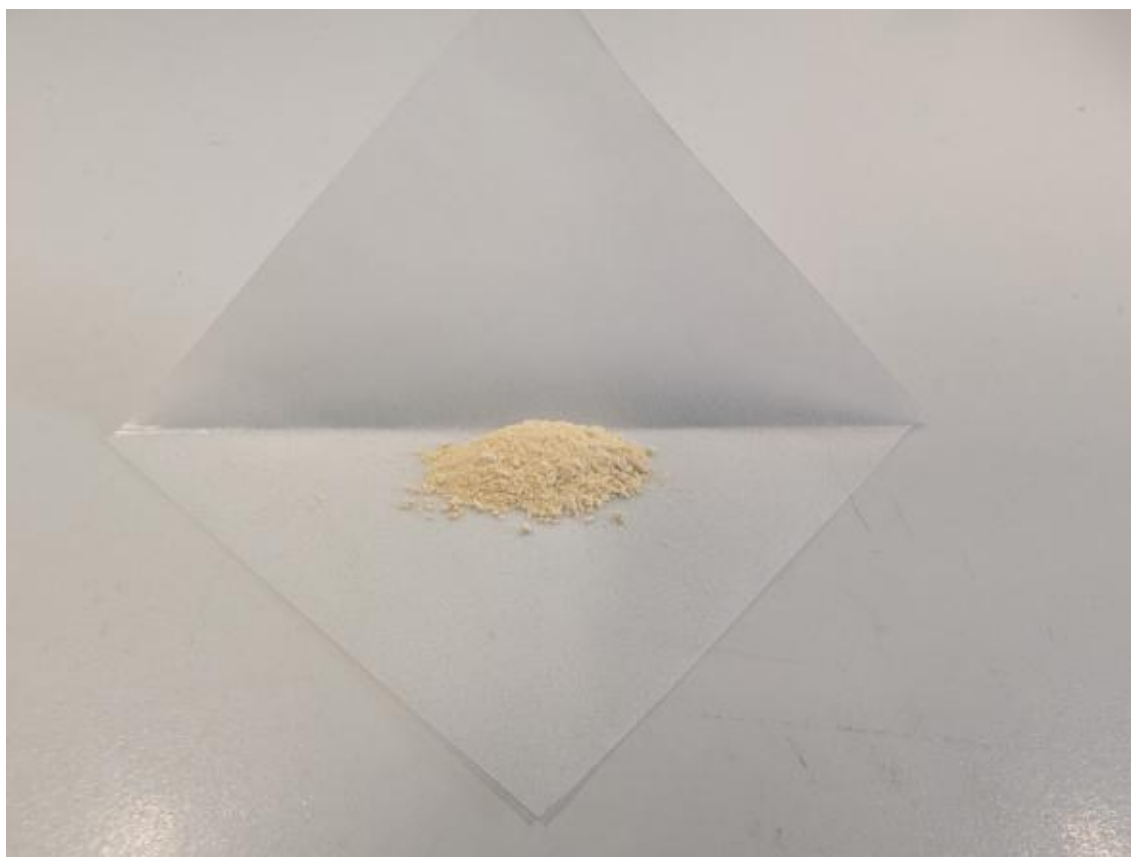

as-prepared Pd@C_3N_4 (light yellow)

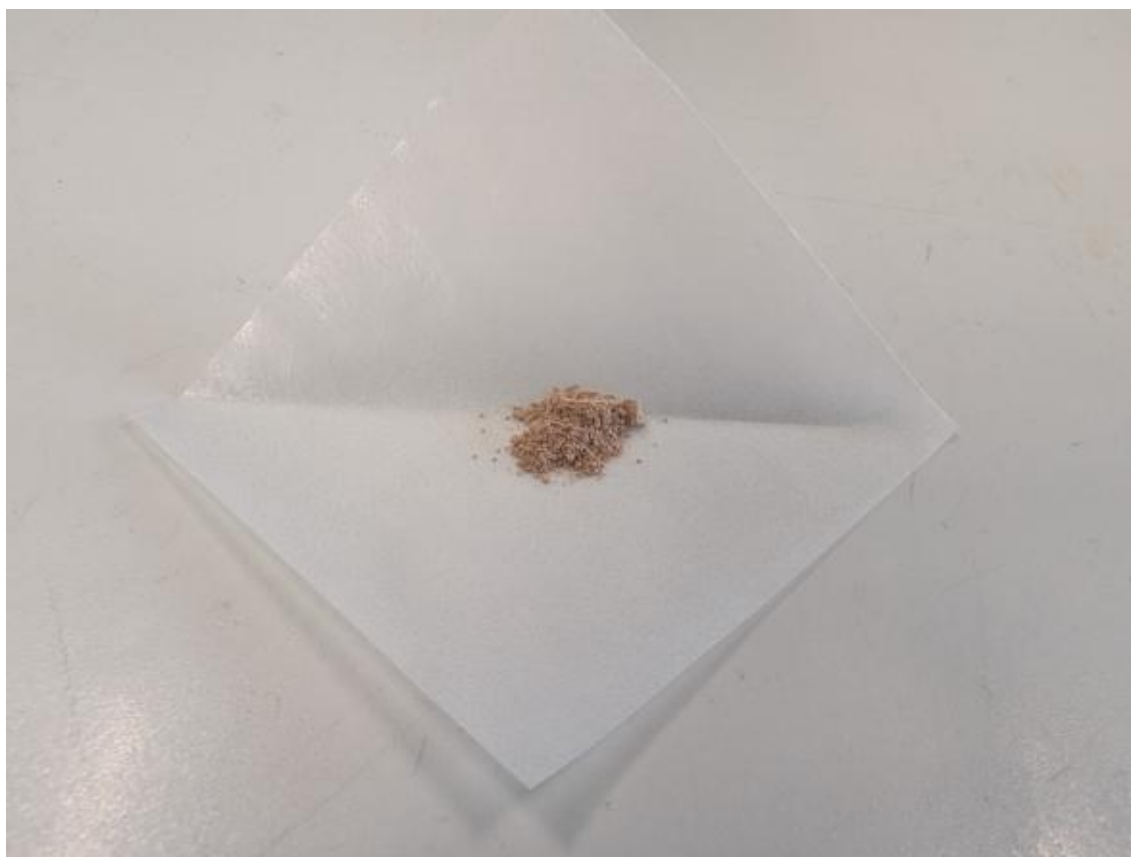

Pd@C_3N_4 after the first cycle and DCM washes to remove organics (pinkish brown)

Figure S8. Pd@C_3N_4 appearance before and after the first cycle.

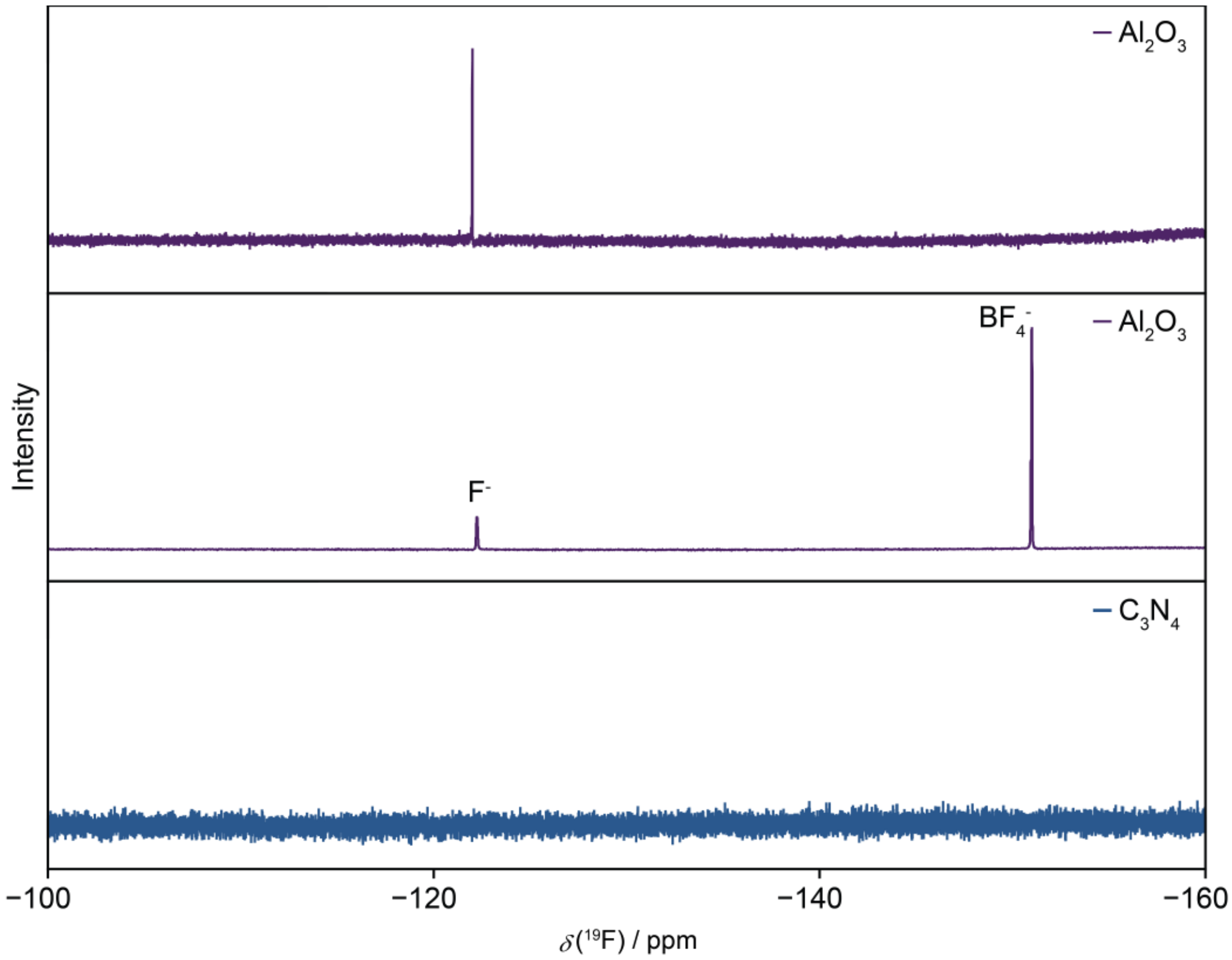

Figure S9. ^{19}F NMR spectra in D_2O of digested Pd@Al_2O_3 (top), digested Pd@Al_2O_3 with $NaBF_4$ as a standard (middle; spectra recorded with comp pulse decoupling), and digested Pd@C_3N_4 (bottom).

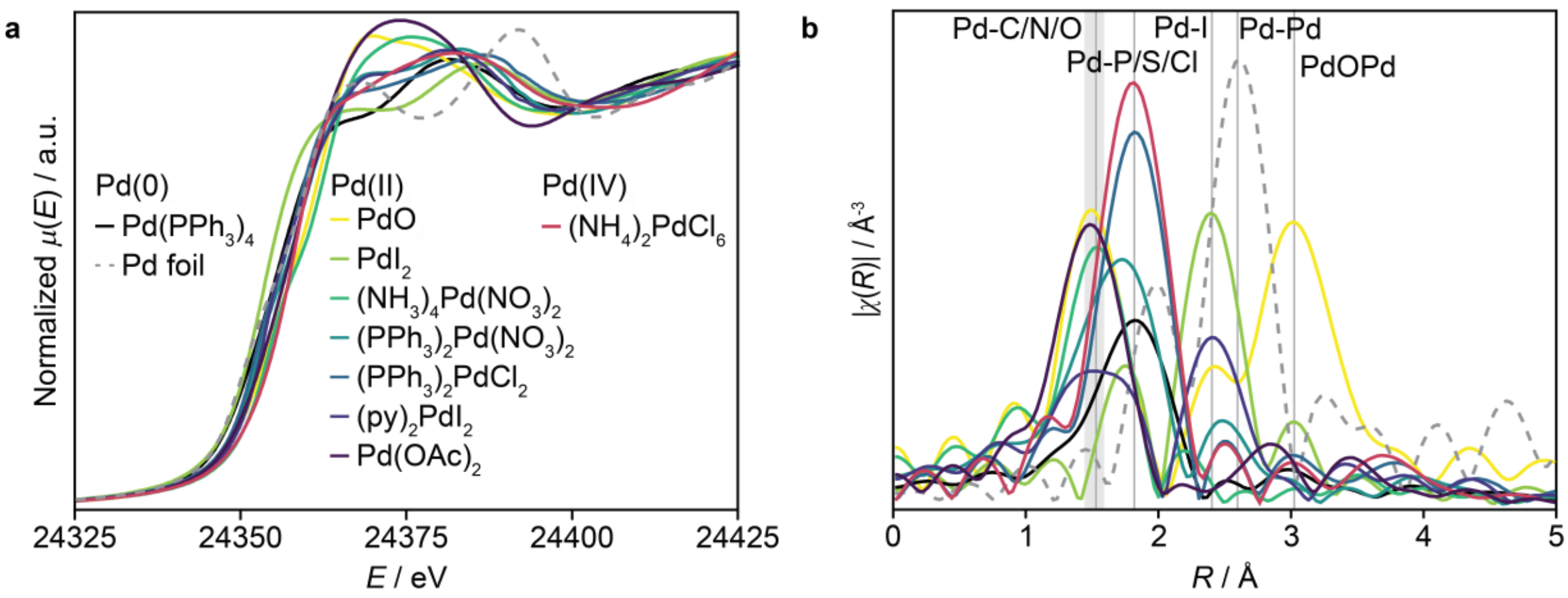

Figure S10. XANES (**a**) and EXAFS (**b**) spectra of Pd(0), Pd(II), and Pd(IV) reference compounds.

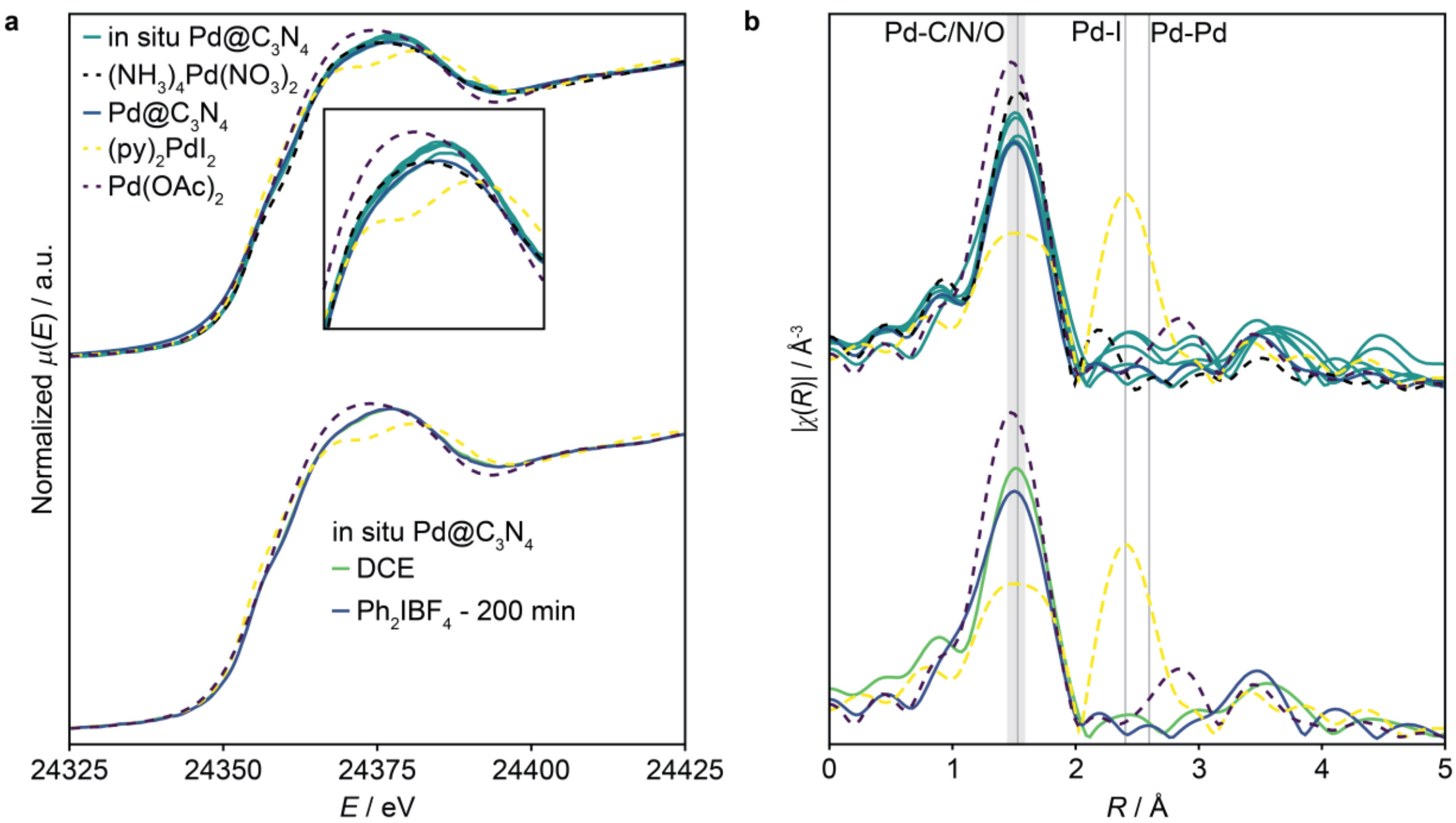

Figure S11. Comparison of in situ XANES (**a**) and EXAFS (**b**) of $Pd@C_3N_4$ with ex situ $Pd@C_3N_4$ and $(NH_3)_4Pd(NO_3)_2$ (top) and in situ spectra of $Pd@C_3N_4$ in DCE and with Ph_2IBF_4.

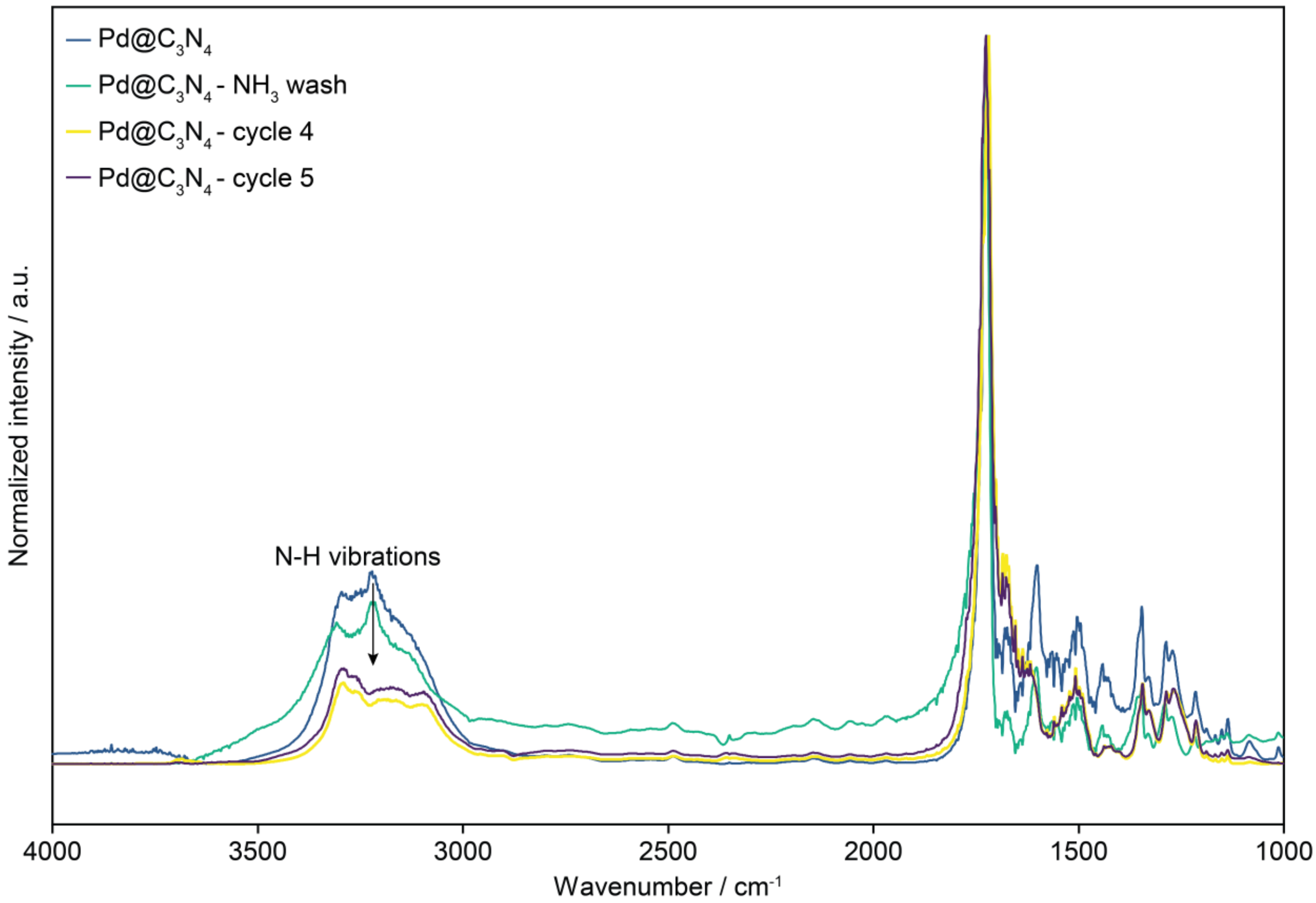

Figure S12. DRIFTS spectra of as-prepared and washed Pd@C_3N_4 as well as Pd@C_3N_4 after the fourth and fifth reaction cycles, indicating decrease in surface N-H bonds relative to the main N=C-N vibrations at ca. 1720 cm^{-1}.

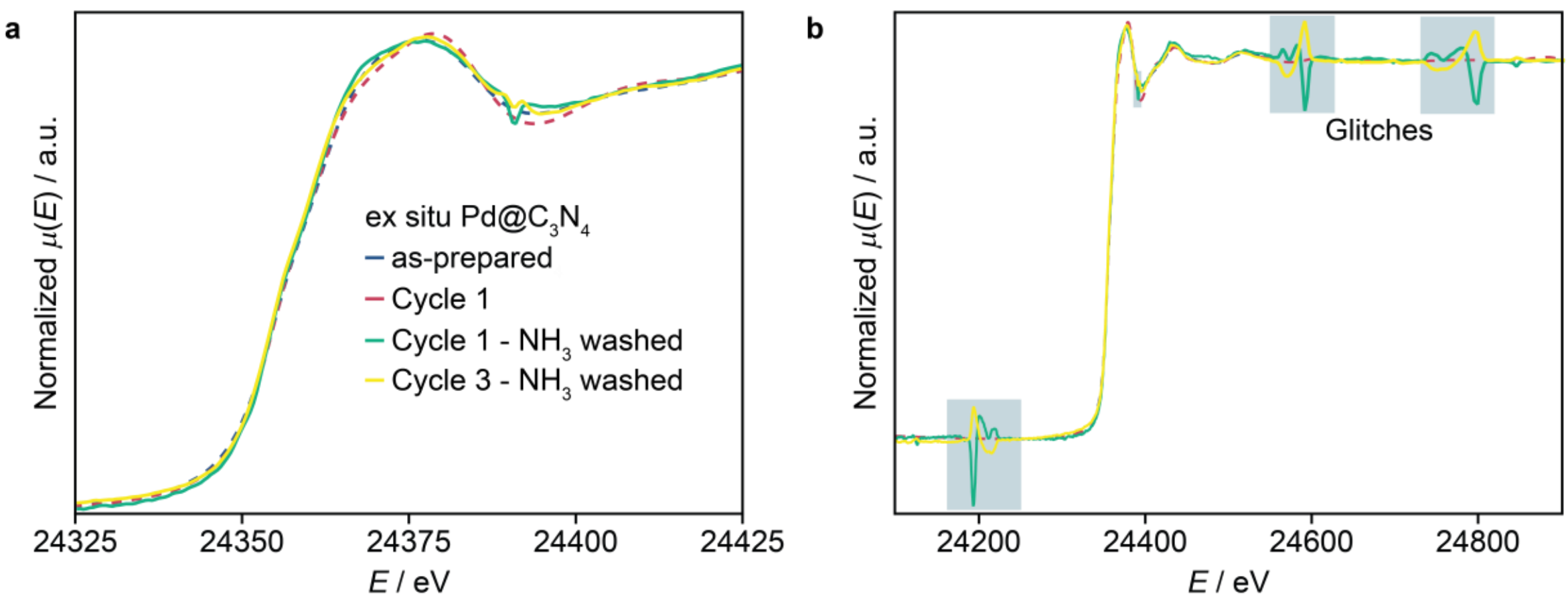

Figure S13. (**a**) Ex situ XANES spectra of Pd@C_3N_4 after the first cycle, first cycle with ammonia wash, and the third cycle with ammonia wash. (**b**) Corresponding XAS spectra with measurement glitches highlighted with grey boxes. The glitches prevented Fourier transformation and EXAFS analysis due to significant signal loss.

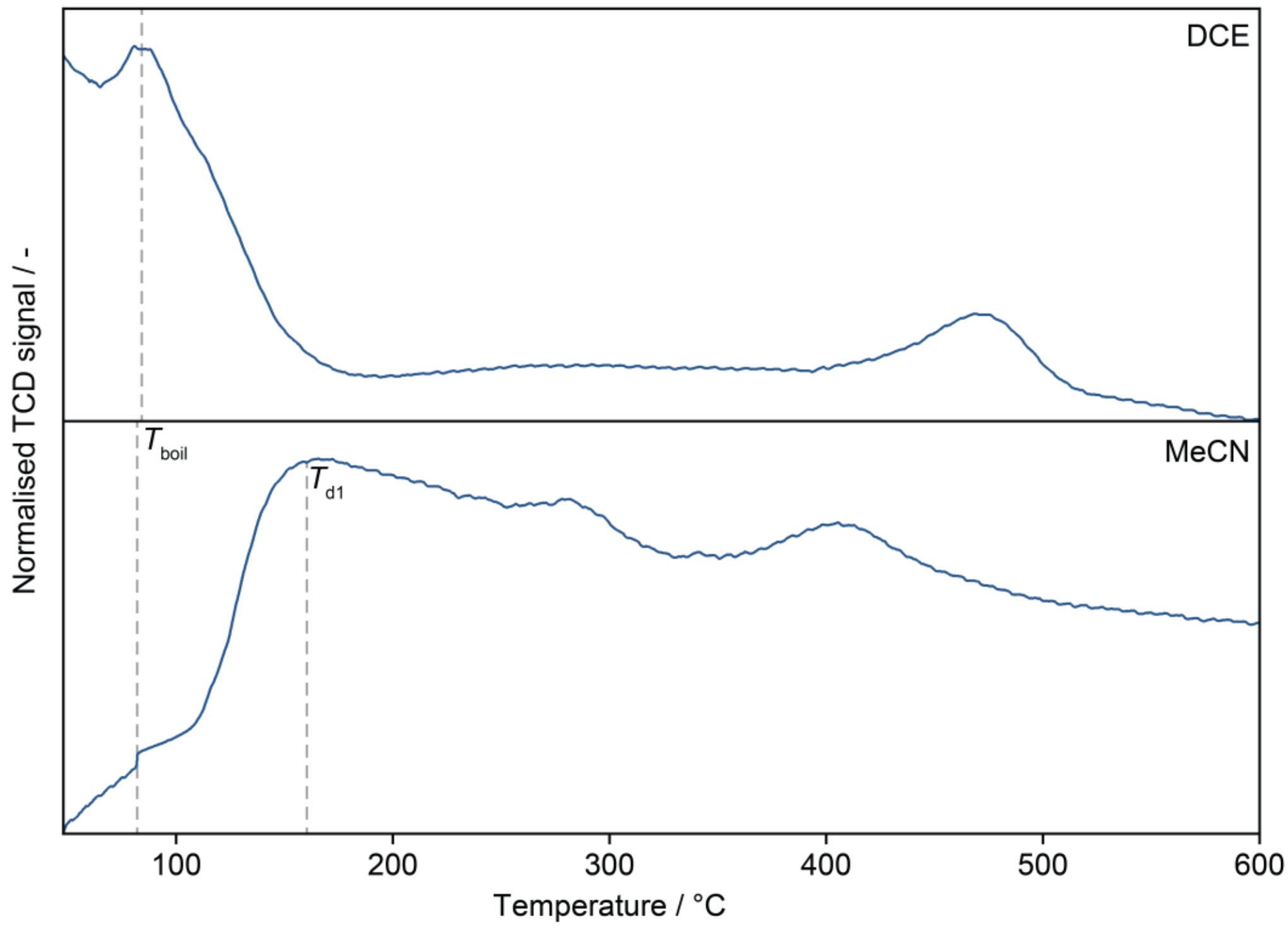

Figure S14. Temperature-dependent desorption curves of DCE (top) and MeCN (bottom) over Pd@Al_2O_3-500. The respective boiling temperature T_{boil} and the first desorption temperature T_{d1} are indicated by gray dashed lines.

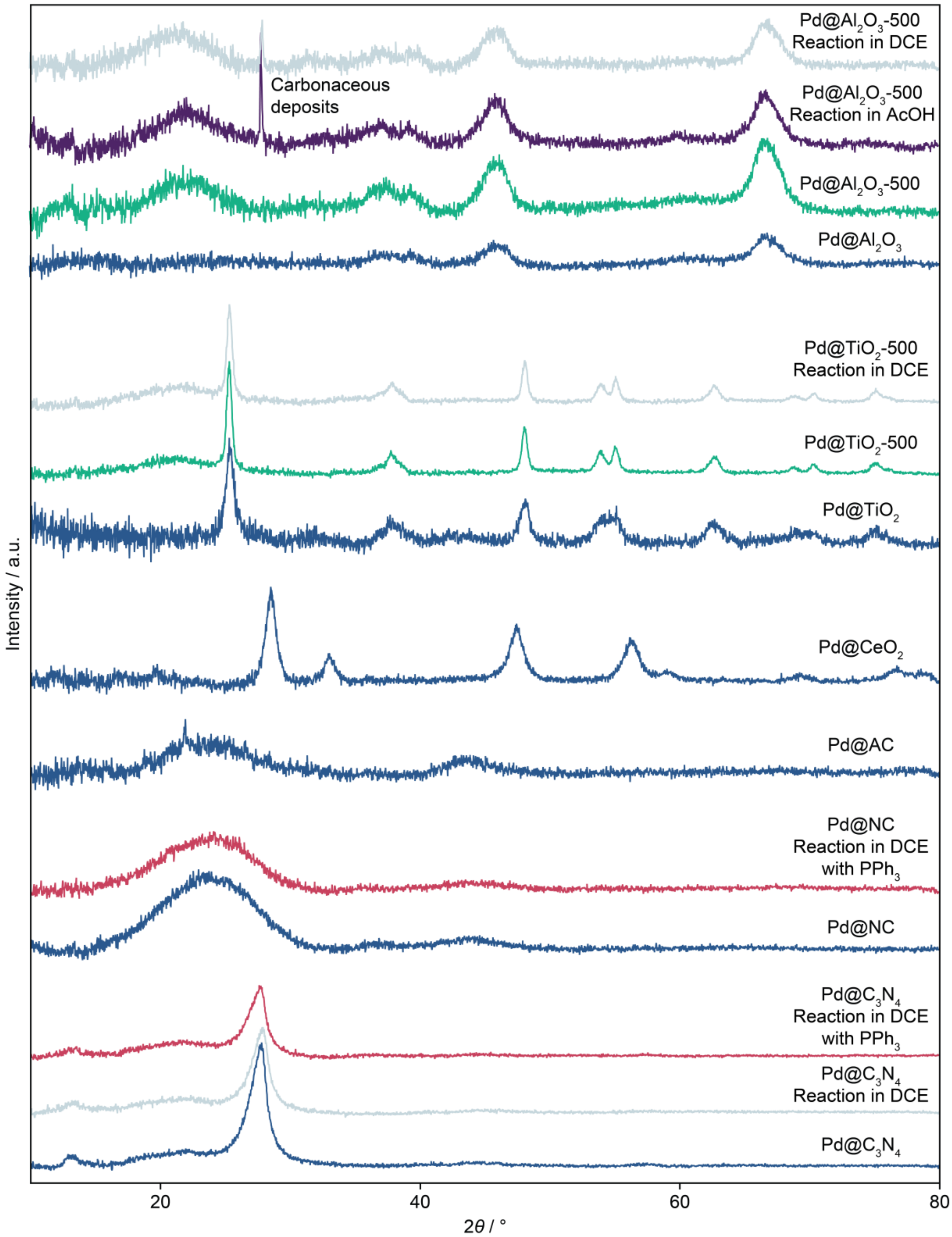

Figure S15. XRD diffractograms of as-prepared and selected used catalysts, prepared in the absence of Pd and PdO peaks. Changes due to the reaction are only observed for alumina-based catalysts by a new peak at 27°, likely corresponding to carbonaceous deposits.

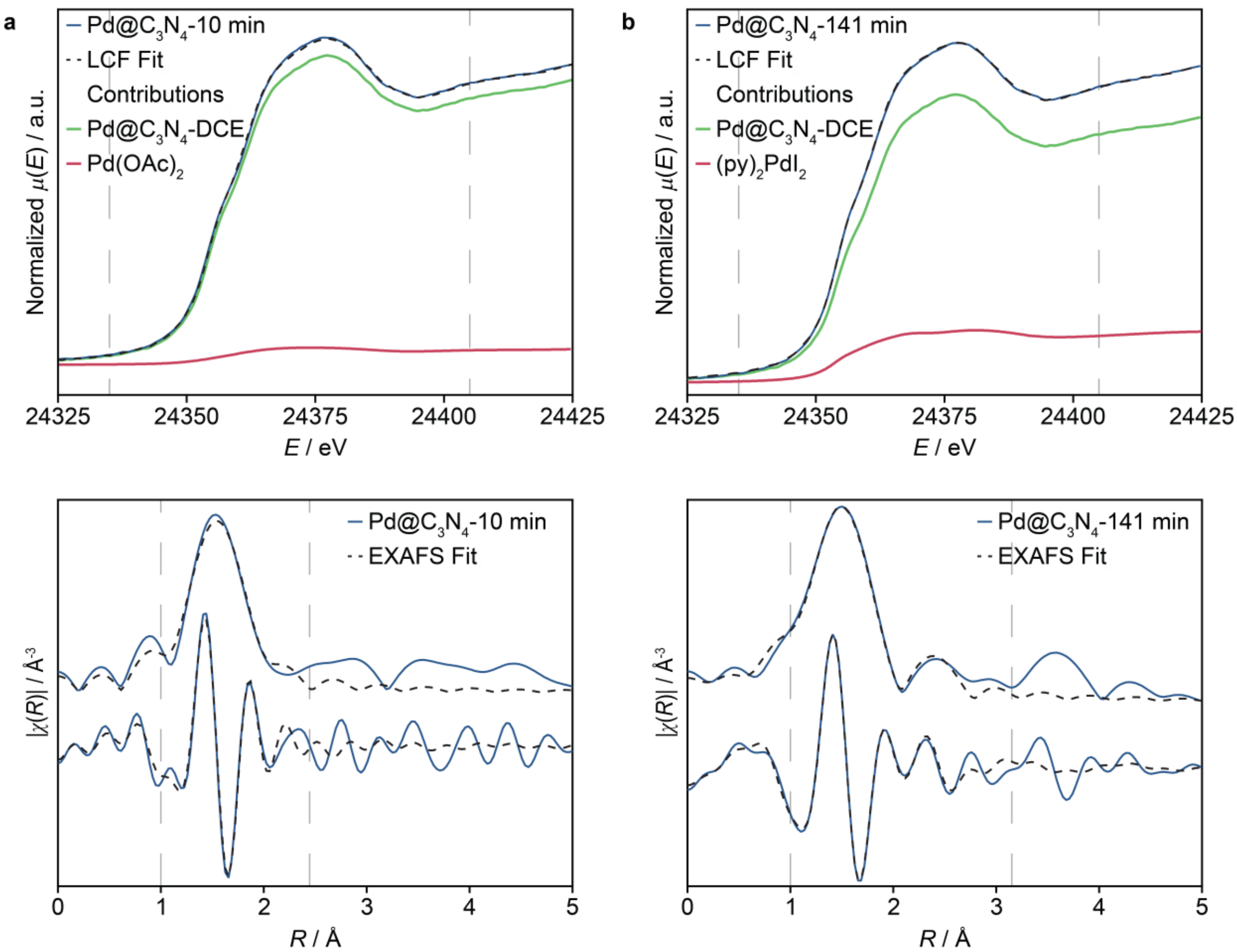

Figure S16. Example XANES (top) and EXAFS (bottom) fits of the in situ measurement of Pd@C_3N_4 under reaction conditions after (**a**) 10 min and (**b**) 142 min following the **GP-S6**.

Supplementary Synthetic Methods

5-fluoro-1-methyl-2-phenyl-1H-indole (2)

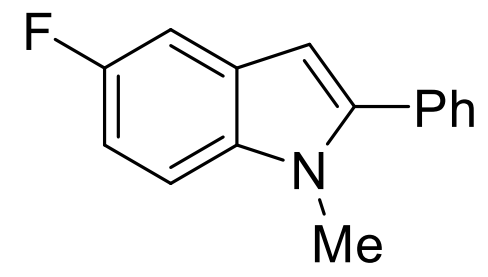

2

Following **GP-S6** with 5-fluoro-1-methylindole (29.8 mg, 200 µmol, 1.00 equiv) and Ph_2IBF_4 **S21** (147 mg, 400 µmol, 2.00 equiv) to give the title compound as a yellow oil which solidified upon cooling (28.5 mg, 127 µmol, 63% yield). 8:1 C2/C3 in the crude mixture.

FCC: SiO_2, 2-4% EtOAc/hexane, $\mathbf{R_f}$ = 0.53 (10% EtOAc/hexane).

^{1}H NMR (400 MHz, $CDCl_3$) δ 7.44-7.36 (m, 4H, Ar*H*), 7.36-7.30 (m, 1H, Ar*H*), 7.23-7.15 (m, 2H, Ar*H*), 6.91 (td, *J* = 9.1, 2.5 Hz, 1H, Ar*H*), 6.44 (d, *J* = 0.8 Hz, 1H, Ar*H*), 3.65 (s, 3H, C*H*$_3$).

^{13}C NMR (100 MHz, $CDCl_3$) δ 158.3 (d, *J* = 234.2 Hz), 143.3, 135.1, 132.7, 129.5, 128.7, 128.2, 128.2 (d, *J* = 10.3 Hz), 110.3 (d, *J* = 9.7 Hz), 110.0 (d, *J* = 26.3 Hz), 105.3 (d, *J* = 23.5 Hz), 101.7 (d, *J* = 4.7 Hz), 31.5.

^{19}F NMR (376 MHz, $CDCl_3$) δ -124.84.

The spectral data was consistent with the literature.[21]

5-fluoro-2-phenyl-1H-indole (3)

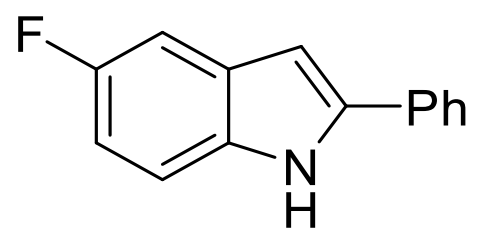

3

Following **GP-S6** with 5-fluoro-1H-indole (27.0 mg, 200 µmol, 1.00 equiv) and Ph_2IBF_4 **S21** (147 mg, 400 µmol, 2.00 equiv) to give the title compound as a white solid (28.8 mg, 136 µmol, 68% yield). 7:1 C2/C3 in the crude mixture.

FCC: SiO_2, 2-10% EtOAc/hexane, **R$_f$** = 0.22 (10% EtOAc/hexane).

^{1}H NMR (400 MHz, $CDCl_3$) δ 8.31 (s, 1H, N*H*), 7.69-7.63 (m, 2H, Ar*H*), 7.49-7.42 (m, 2H, Ar*H*), 7.39-7.32 (m, 1H, Ar*H*), 7.32-7.25 (m, 2H, Ar*H*), 6.94 (ddd, *J* = 9.3, 8.8, 2.5 Hz, 1H, Ar*H*), 6.79 (dd, *J* = 2.1, 0.9 Hz, 1H, Ar*H*).

^{13}C NMR (100 MHz, $CDCl_3$) δ 158.4 (d, *J* = 234.7 Hz), 139.8, 133.5, 132.2, 129.8 (d, *J* = 10.5 Hz), 129.2, 128.2, 125.4, 111.6 (d, *J* = 9.8 Hz), 110.8 (d, *J* = 26.4 Hz), 105.6 (d, *J* = 23.6 Hz), 100.2 (d, *J* = 4.8 Hz).

^{19}F NMR (376 MHz, $CDCl_3$) δ -124.17.

The spectral data was consistent with the literature.[22]

3-methyl-2-phenyl-1H-indole (6)

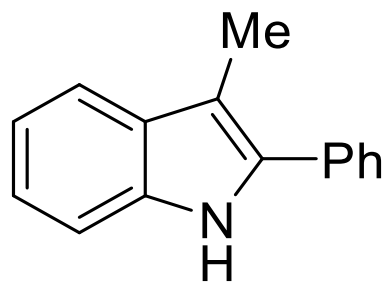

6

Following **GP-S6** with 3-methyl-1H-indole (26.2 mg, 200 μmol, 1.00 equiv) and Ph_2IBF_4 **S21** (147 mg, 400 μmol, 2.00 equiv) to give the title compound as a light brown solid (19.4 mg, 93.6 μmol, 47% yield).

FCC: SiO_2, 2-10% EtOAc/hexane, **R_f** = 0.31 (10% EtOAc/hexane).

^{1}H NMR (400 MHz, $CDCl_3$) δ 8.01 (s, 1H), 7.64-7.57 (m, 3H, Ar*H*), 7.52-7.45 (m, 2H, Ar*H*), 7.40-7.32 (m, 2H, Ar*H*), 7.22 (ddd, *J* = 8.1, 7.0, 1.3 Hz, 1H, Ar*H*), 7.16 (ddd, *J* = 8.1, 7.1, 1.1 Hz, 1H, Ar*H*), 2.48 (s, 3H, CH_3).

^{13}C NMR (100 MHz, $CDCl_3$) δ 136.0, 134.2, 133.5, 130.2, 129.0, 127.9, 127.5, 122.5, 119.7, 119.1, 110.8, 108.9, 9.8.

The spectral data was consistent with the literature.[23]

2-phenyl-1H-indole (7)

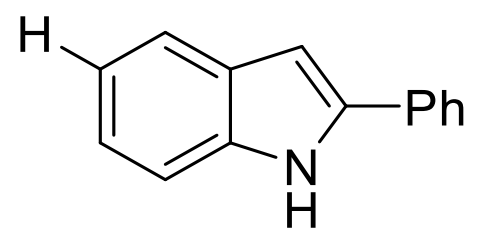

7

Following **GP-S6** with indole (23.4 mg, 200 μmol, 1.00 equiv) and Ph_2IBF_4 **S21** (147 mg, 400 μmol, 2.00 equiv) to give the title compound as a white solid (18.8 mg, 97.3 μmol, 49% yield). No other isomers were detected in the crude ^{1}H NMR or during purification.

FCC: SiO_2, 2-10% EtOAc/hexane, **R_f** = 0.32 (10% EtOAc/hexane).

^{1}H NMR (400 MHz, $CDCl_3$) δ 8.33 (s, 1H, N*H*), 7.70-7.66 (m, 2H), 7.63 (d, *J* = 7.8 Hz, 1H, Ar*H*), 7.45 (dd, *J* = 8.4, 7.1 Hz, 2H, Ar*H*), 7.41 (d, *J* = 8.2 Hz, 1H, Ar*H*), 7.36-7.30 (m, 1H, Ar*H*), 7.20 (ddd, *J* = 8.2, 6.9, 1.3 Hz, 1H, Ar*H*), 7.13 (ddd, *J* = 8.1, 7.1, 1.1 Hz, 1H, Ar*H*), 6.84 (dd, *J* = 2.2, 0.9 Hz, 1H, Ar*H*).

^{13}C NMR (100 MHz, $CDCl_3$) δ 138.0, 137.0, 132.5, 129.4, 129.2, 127.9, 125.3, 122.5, 120.8, 120.4, 111.0, 100.2.

The spectral data was consistent with the literature.[24]

5-chloro-2-phenyl-1H-indole (9)

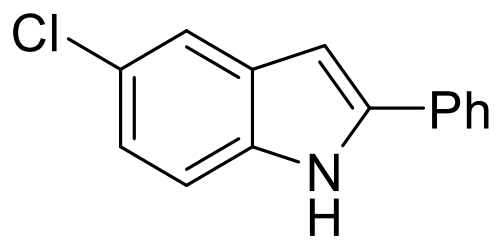

9

Following **GP-S6** with 5-chloro-1H-indole (30.3 mg, 200 µmol, 1.00 equiv) and Ph_2IBF_4 **S21** (147 mg, 400 µmol, 2.00 equiv) to give the title compound as a white solid (30.0 mg, 132 µmol, 66% yield). 10:1 C2/C3 in the crude mixture.

FCC: SiO_2, 2-10% EtOAc/hexane, $\mathbf{R_f}$ = 0.21 (10% EtOAc/hexane).

^{1}H NMR (400 MHz, $CDCl_3$) δ 8.35 (s, 1H, N*H*), 7.67-7.63 (m, 2H, Ar*H*), 7.59 (d, *J* = 2.0 Hz, 1H, Ar*H*), 7.49-7.43 (m, 2H, Ar*H*), 7.38-7.33 (m, 1H, Ar*H*), 7.31 (dt, *J* = 8.6, 0.7 Hz, 1H, Ar*H*), 7.14 (dd, *J* = 8.6, 2.0 Hz, 1H, Ar*H*), 6.76 (dd, *J* = 2.2, 0.9 Hz, 1H, Ar*H*).

^{13}C NMR (100 MHz, $CDCl_3$) δ 139.4, 135.3, 132.0, 130.5, 129.3, 128.3, 126.0, 125.39, 122.7, 120.1, 112.0, 99.7.

The spectral data was consistent with the literature.[24]

5-bromo-2-phenyl-1H-indole (10)

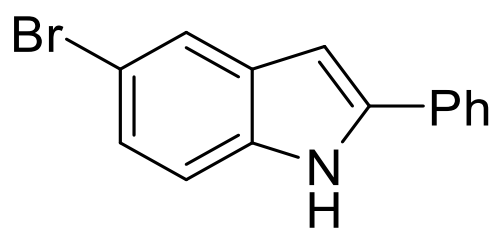

10

Following **GP-S6** with 5-bromo-1H-indole (39.2 mg, 200 µmol, 1.00 equiv) and Ph_2IBF_4 **S21** (147 mg, 400 µmol, 2.00 equiv) to give the title compound as a white solid (40.0 mg, 147 µmol, 73% yield). 9:1 C2/C3 in the crude mixture.

FCC: SiO_2, 2-10% EtOAc/hexane, $\mathbf{R_f}$ = 0.18 (10% EtOAc/hexane).

^{1}**H NMR** (400 MHz, $CDCl_3$) δ 8.35 (s, 1H, N*H*), 7.77-7.73 (m, 1H, Ar*H*), 7.67-7.63 (m, 2H, Ar*H*), 7.49-7.43 (m, 2H, Ar*H*), 7.38-7.33 (m, 1H, Ar*H*), 7.27 (d, *J* = 1.2 Hz, 2H, Ar*H*), 6.76 (d, *J* = 2.1 Hz, 1H, Ar*H*).

^{13}**C NMR** (100 MHz, $CDCl_3$) δ 139.3, 135.5, 132.0, 131.2, 129.3, 128.3, 125.4, 125.3, 123.2, 113.6, 112.4, 99.6.

The spectral data was consistent with the literature.[25]

5-iodo-2-phenyl-1H-indole (11)

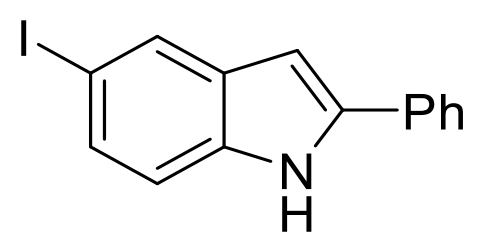

11

Following **GP-S6** with 5-iodo-1H-indole (48.6 mg, 200 μmol, 1.00 equiv) and Ph_2IBF_4 **S21** (147 mg, 400 μmol, 2.00 equiv) to give the title compound as a white solid (17.5 mg, 54.8 μmol, 27% yield). No other isomers were detected in the crude ^{1}H NMR or during purification.

FCC: SiO_2, 2-10% EtOAc/hexane, $\mathbf{R_f}$ = 0.24 (10% EtOAc/hexane).

^{1}H NMR (400 MHz, $CDCl_3$) δ 8.35 (s, 1H, N*H*), 7.96 (dt, *J* = 1.5, 0.6 Hz, 1H, Ar*H*), 7.67-7.62 (m, 2H, Ar*H*), 7.49-7.41 (m, 3H, Ar*H*), 7.39-7.31 (m, 1H, Ar*H*), 7.19 (dt, *J* = 8.5, 0.8 Hz, 1H, Ar*H*), 6.74 (dd, *J* = 2.2, 0.9 Hz, 1H, Ar*H*).

^{13}C NMR (100 MHz, $CDCl_3$) δ 138.9, 136.0, 132.0, 131.9, 130.8, 129.6, 129.3, 128.3, 125.4, 112.9, 99.3, 83.8.

IR (ν_{max}, cm^{-1}) 3289 (m), 2935 (w), 1685 (s), 1475 (m), 1458 (m), 1311 (m), 898 (m), 763 (s).

HRMS (ESI/QTOF) m/z: $[M + H]^+$ Calcd for $C_{14}H_{11}IN^+$ 319.9931; Found 319.9934.

Methyl 2-phenyl-1H-indole-5-carboxylate (12)

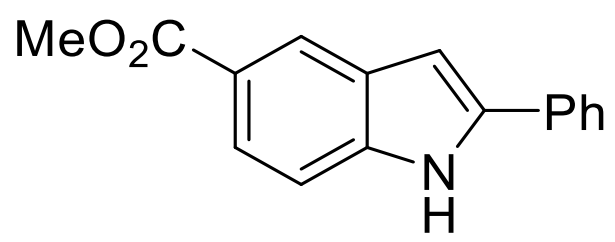

12

Following **GP-S6** with methyl 1H-indole-5-carboxylate (35.0 mg, 200 µmol, 1.00 equiv) and Ph_2IBF_4 **S21** (147 mg, 400 µmol, 2.00 equiv) to give the title compound as a white solid (8.30 mg, 33.0 µmol, 17% yield). 7:1 C2/C3 in the crude mixture.

FCC: SiO_2, 2-10% EtOAc/hexane, $\mathbf{R_f}$ = 0.15 (10% EtOAc/hexane).

^{1}H NMR (400 MHz, $CDCl_3$) δ 8.61 (s, 1H, N*H*), 8.40 (dt, *J* = 1.5, 0.7 Hz, 1H, Ar*H*), 7.91 (dd, *J* = 8.6, 1.6 Hz, 1H, Ar*H*), 7.72-7.64 (m, 2H, Ar*H*), 7.46 (tq, *J* = 6.9, 0.7 Hz, 2H, Ar*H*), 7.41 (dt, *J* = 8.6, 0.8 Hz, 1H, Ar*H*), 7.39-7.32 (m, 1H, Ar*H*), 6.90 (dd, *J* = 2.2, 0.9 Hz, 1H, Ar*H*), 3.94 (s, 3H, CH_3).

^{13}C NMR (100 MHz, $CDCl_3$) δ 168.3, 139.5, 139.3, 131.9, 129.3, 129.0, 128.3, 125.4, 123.9, 123.7, 122.5, 110.7, 101.1, 52.0.

The spectral data was consistent with the literature.[26]

5-methyl-2-phenyl-1H-indole (13)

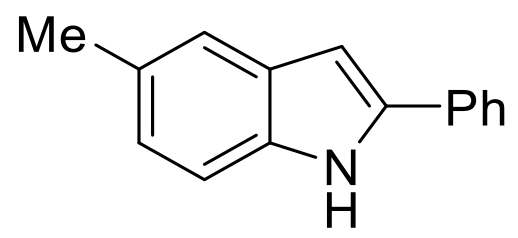

13

Following **GP-S6** with 5-methyl-1H-indole (26.2 mg, 200 µmol, 1.00 equiv) and Ph_2IBF_4 **S21** (147 mg, 400 µmol, 2.00 equiv) to give the title compound as a white solid (12.5 mg, 60.3 µmol, 30% yield). 7:1 C2/C3 in the crude mixture.

FCC: SiO_2, 2-10% EtOAc/hexane, $\mathbf{R_f}$ = 0.33 (10% EtOAc/hexane).

^{1}H NMR (400 MHz, $CDCl_3$) δ 8.16 (s, 1H, N*H*), 7.60-7.56 (m, 2H, Ar*H*), 7.41-7.33 (m, 3H, Ar*H*), 7.29-7.20 (m, 2H, Ar*H*), 6.95 (dd, *J* = 8.2, 1.6 Hz, 1H, Ar*H*), 6.68 (dd, *J* = 2.2, 0.9 Hz, 1H, Ar*H*), 2.38 (s, 3H, C*H₃*).

^{13}C NMR (100 MHz, $CDCl_3$) δ 138.1, 135.3, 132.7, 129.7, 129.6, 129.1, 127.7, 125.2, 124.1, 120.5, 110.7, 99.7, 21.6.

The spectral data was consistent with the literature.[27]

5-methoxy-2-phenyl-1H-indole (14)

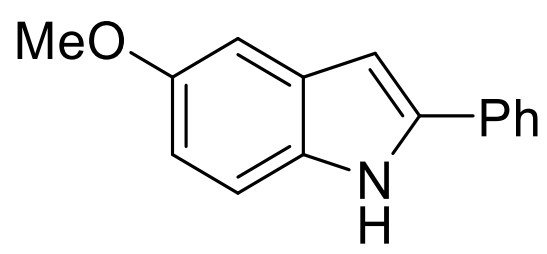

14

Following **GP-S6** (reaction temperature 60 °C instead of 80 °C) with 5-methoxy-1H-indole (29.4 mg, 200 μmol, 1.00 equiv) and Ph_2IBF_4 **S21** (147 mg, 400 μmol, 2.00 equiv) to give the title compound as a light-yellow solid (11.8 mg, 52.9 μmol, 26% yield). 4:1 C2/C3 in the crude mixture.

FCC: SiO_2, 5-60% DCM/hexane, $\mathbf{R_f}$ = 0.31 (60% DCM/hexane).

^{1}H NMR (400 MHz, $CDCl_3$) δ 8.22 (s, 1H, N*H*), 7.68-7.63 (m, 2H, Ar*H*), 7.48-7.39 (m, 2H, Ar*H*), 7.35-7.27 (m, 2H, Ar*H*), 7.09 (d, *J* = 2.4 Hz, 1H, Ar*H*), 6.86 (dd, *J* = 8.8, 2.4 Hz, 1H, Ar*H*), 6.76 (dd, *J* = 2.1, 0.9 Hz, 1H, Ar*H*), 3.87 (s, 3H, OCH_3).

^{13}C NMR (100 MHz, $CDCl_3$) δ 154.7, 138.8, 132.6, 132.2, 129.9, 129.2, 127.8, 125.2, 112.8, 111.8, 102.4, 100.0, 56.0.

The spectral data was consistent with the literature.[25]

Note. While the ^{1}H NMR yield of 39% was obtained (CH_2Br_2 internal standard), the isolated yield was reduced due to a challenging separation from an unknown impurity.

2-phenylbenzofuran (15)

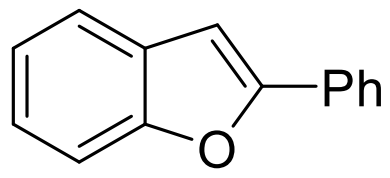

15

Following **GP-S6** with benzofuran (23.6 mg, 200 µmol, 1.00 equiv) and Ph_2IBF_4 **S21** (147 mg, 400 µmol, 2.00 equiv) to give the title compound as a white solid (10.5 mg, 54.1 µmol, 27% yield). No other isomers were detected in the crude ^{1}H NMR or during purification.

FCC: SiO_2, 2-4% EtOAc/hexane, $\mathbf{R_f}$ = 0.36 (10% EtOAc/hexane).

^{1}H NMR (400 MHz, $CDCl_3$) δ 7.90-7.85 (m, 2H, Ar*H*), 7.59 (ddd, *J* = 7.5, 1.5, 0.7 Hz, 1H, Ar*H*), 7.53 (dq, *J* = 8.1, 0.9 Hz, 1H, Ar*H*), 7.48-7.42 (m, 2H, Ar*H*), 7.39-7.33 (m, 1H, Ar*H*), 7.29 (ddd, *J* = 8.1, 7.3, 1.5 Hz, 1H, Ar*H*), 7.23 (td, *J* = 7.4, 1.2 Hz, 1H, Ar*H*), 7.03 (s, 1H, Ar*H*).

^{13}C NMR (100 MHz, $CDCl_3$) δ 156.1, 155.0, 130.6, 129.4, 128.9, 128.7, 125.1, 124.4, 123.1, 121.1, 111.3, 101.5.

The spectral data was consistent with the literature.[28]

5-fluoro-2-(4-fluorophenyl)-1H-indole (16)

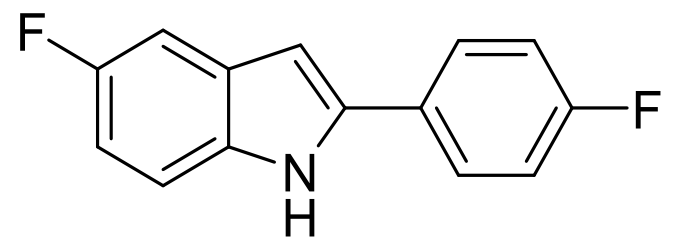

16

Following **GP-S6** with 5-fluoro-1H-indole (27.0 mg, 200 μmol, 1.00 equiv) and **S23** (162 mg, 400 μmol, 2.00 equiv) to give the title compound as a white solid (38.1 mg, 166 μmol, 83% yield). 9:1 C2/C3 in the crude mixture.

FCC: SiO_2, 2-10% EtOAc/hexane, $\mathbf{R_f}$ = 0.24 (10% EtOAc/hexane).

^{1}H NMR (400 MHz, $CDCl_3$) δ 8.23 (s, 1H, N*H*), 7.64-7.57 (m, 2H, Ar*H*), 7.30 (dd, *J* = 8.9, 4.4 Hz, 1H, Ar*H*), 7.28-7.24 (m, 1H, Ar*H*), 7.18-7.11 (m, 2H, Ar*H*), 6.94 (td, *J* = 9.1, 2.5 Hz, 1H, Ar*H*), 6.71 (dd, *J* = 2.3, 0.8 Hz, 1H, Ar*H*).

^{13}C NMR (100 MHz, $CDCl_3$) δ 162.7 (d, *J* = 248.2 Hz), 158.4 (d, *J* = 234.9 Hz), 138.9, 133.5, 129.8 (d, *J* = 10.4 Hz), 128.6 (d, *J* = 3.3 Hz), 127.1 (d, *J* = 8.2 Hz), 116.3 (d, *J* = 21.8 Hz), 111.6 (d, *J* = 9.6 Hz), 110.9 (d, *J* = 26.5 Hz), 105.6 (d, *J* = 23.6 Hz), 100.2 (d, *J* = 4.6 Hz).

^{19}F NMR (376 MHz, $CDCl_3$) δ -113.40 (1F), -123.99 (1F).

IR (ν_{max}, cm^{-1}) 3433 (m), 2928 (m), 2853 (w), 1608 (m), 1547 (m), 1483 (s), 1451 (m), 1430 (m), 1249 (s), 836 (s), 784 (s)

HRMS (ESI/QTOF) m/z: $[M + H]^+$ Calcd for $C_{14}H_{10}F_2N^+$ 230.0776; Found 230.0765.

5-fluoro-2-(4-(trifluoromethyl)phenyl)-1H-indole (17)

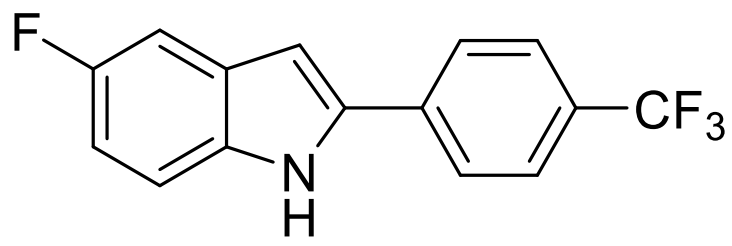

17

Following **GP-S6** with 5-fluoro-1H-indole (27.0 mg, 200 µmol, 1.00 equiv) and **S24** (202 mg, 400 µmol, 2.00 equiv) to give the title compound as a white solid (17.0 mg, 60.9 µmol, 30% yield). 11:1 C2/C3 in the crude mixture.

FCC: SiO_2, 2-10% EtOAc/hexane, $\mathbf{R_f}$ = 0.24 (10% EtOAc/hexane).

^{1}H NMR (600 MHz, $CDCl_3$) δ 8.36 (s, 1H), 7.77-7.67 (m, 4H), 7.34 (dd, *J* = 8.8, 4.3 Hz, 1H), 7.29 (dd, *J* = 9.4, 2.5 Hz, 1H), 6.99 (td, *J* = 9.0, 2.5 Hz, 1H), 6.88 (d, *J* = 2.2 Hz, 1H).

^{13}C NMR (151 MHz, $CDCl_3$) δ 158.5 (d, *J* = 235.6 Hz), 138.0, 135.5, 133.8, 129.9 (q, *J* = 32.6 Hz), 129.5 (d, *J* = 10.3 Hz), 126.3 (q, *J* = 3.8 Hz), 125.4, 124.2 (q, *J* = 272.0 Hz), 111.9, 111.8 (d, *J* = 36.8 Hz), 105.9 (d, *J* = 23.7 Hz), 101.9 (d, *J* = 4.7 Hz).

^{19}F NMR (376 MHz, $CDCl_3$) δ -62.60 (3F), -123.49 (1F).

The spectral data was consistent with the literature.[29]

5-fluoro-2-(p-tolyl)-1H-indole (18)

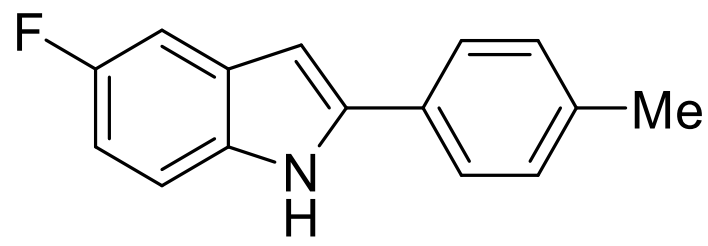

18

Following **GP-S6** with 5-fluoro-1H-indole (27.0 mg, 200 µmol, 1.00 equiv) and **S22** (158 mg, 400 µmol, 2.00 equiv) to give the title compound as a white solid (21.0 mg, 93.2 µmol, 47% yield). 3:1 C2/C3 in the crude mixture.

FCC: SiO_2, 2-10% EtOAc/hexane, $\mathbf{R_f}$ = 0.23 (10% EtOAc/hexane).

^{1}H NMR (400 MHz, $CDCl_3$) δ 8.27 (s, 1H, N*H*), 7.55 (d, *J* = 8.1 Hz, 2H, Ar*H*), 7.32-7.28 (m, 1H, Ar*H*), 7.28-7.22 (m, 3H, Ar*H*), 6.92 (td, *J* = 9.1, 2.5 Hz, 1H, Ar*H*), 6.74 (d, *J* = 2.2 Hz, 1H, Ar*H*), 2.40 (s, 3H, CH_3).

^{13}C NMR (100 MHz, $CDCl_3$) δ 158.3 (d, *J* = 234.5 Hz), 140.0, 138.2, 133.3, 129.9, 129.8, 129.4, 125.3, 111.5 (d, *J* = 9.7 Hz), 110.5 (d, *J* = 26.4 Hz), 105.4 (d, *J* = 23.7 Hz), 99.7 (d, *J* = 4.7 Hz), 21.4.

^{19}F NMR (376 MHz, $CDCl_3$) δ -124.33.

The spectral data was consistent with the literature,[30] apart from ^{13}C NMR signal at 21.4 ppm.

Scale up experiment

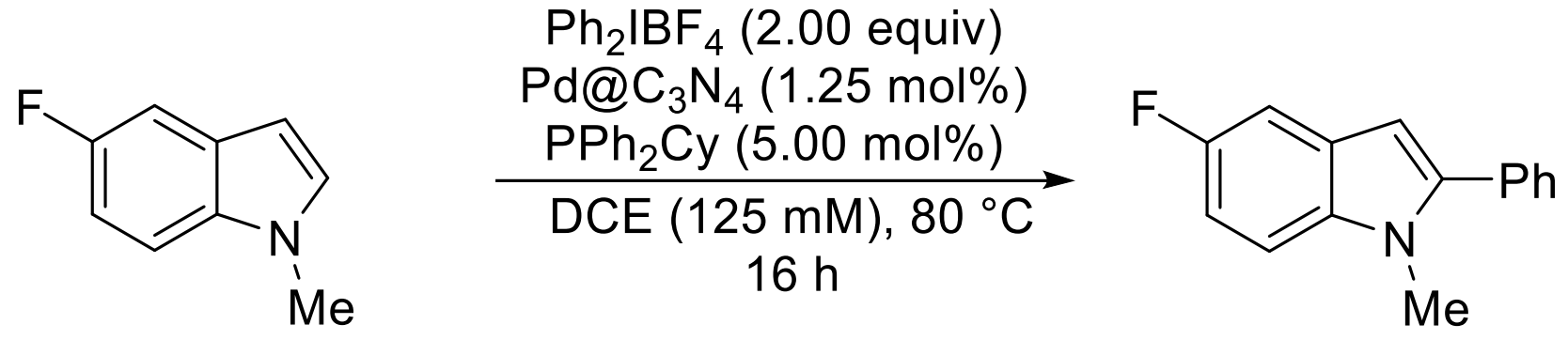

1
1.00 mmol

2

An oven dried vial was charged with 5-fluoro-1-methylindole **1** (149 mg, 1.00 mmol, 1.00 equiv), Ph_2IBF_4 **S19** (736 mg, 2.00 mmol, 2.00 equiv), Pd@C_3N_4 (267 mg, 12.5 μmol, 1.25 mol%), PPh_2Cy (13.4 mg, 50.0 μmol, 5.00 mol%), and DCE (8.00 mL) under air. The vial was then sealed and stirred (1000 rpm) on a pre-heated hotplate at 80 °C for 16 h. The resulting mixture was then diluted with DCM (17 mL) and centrifuged to remove the catalyst. The catalyst was then washed with DCM (3 x 25 mL). The combined washes were concentrated under reduced pressure, and the obtained crude residue was purified by FCC (2-4% EtOAc/hexane) to give **2** as a light-yellow solid (158 mg, 699 μmol, 70% yield).

Glassware used: Biotage Microwave Reaction Kit, 10-20 mL (cat. no. 354833).

Synthesis of metal complexes

***Trans*-bis(pyridine)palladium dichloride (S4)**

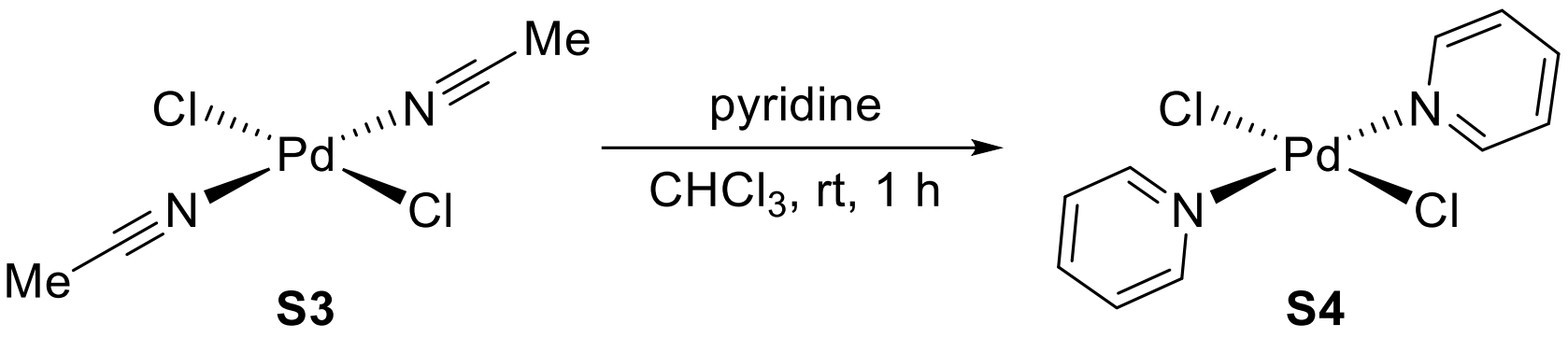

Following a reported procedure,[31] $Pd(MeCN)_2Cl_2$ (110 mg, 424 μmol, 1.00 equiv) was dissolved in dry $CHCl_3$ (2.50 mL), followed by the addition of pyridine (68.8 mg, 70.0 μL, 869 μmol, 2.05 equiv). The resulting mixture was stirred at rt for 1 h. Then, it was concentrated under reduced pressure and resuspended in Et_2O (5 mL). The mixture was filtered to collect the title compound as a yellow solid (116 mg, 343 μmol, 81% yield).

^{1}H NMR (400 MHz, $CDCl_3$) δ 8.84 (d, *J* = 5.8 Hz, 4H, Ar*H*), 7.79 (t, *J* = 7.6 Hz, 2H, Ar*H*), 7.34 (t, *J* = 6.9 Hz, 4H, Ar*H*).

^{13}C NMR (100 MHz, $CDCl_3$) δ 153.5, 138.7, 125.1.

The spectral data was consistent with the literature.[32]

***Trans*-bis(pyridine)palladium diiodide (S5)**

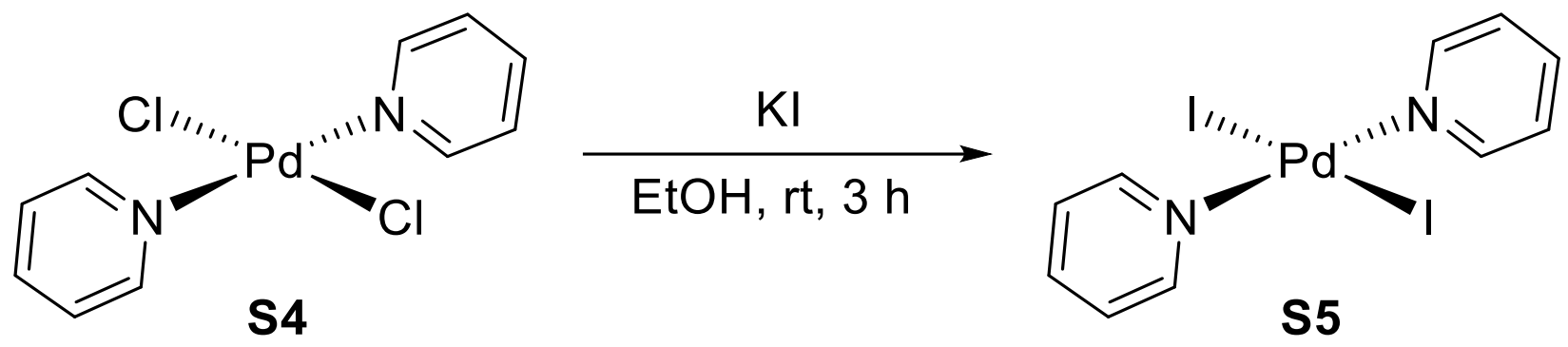

Following a modified reported procedure,[31] $Pd(py)_2Cl_2$ (116 mg, 343 µmol, 1.00 equiv) and KI (359 mg, 2.16 mmol, 6.31 equiv) were stirred in EtOH (5.89 mL) at rt for 16 h. The mixture was then concentrated under reduced pressure. The crude residue was suspended in water and filtered to collect the solid. The solid was then washed with water (3 x 5 mL) and Et_2O (3 x 5 mL) to give the title compound as a dark orange solid (91.8 mg, 176 µmol, 51% yield).

^{1}H NMR (400 MHz, $CDCl_3$) δ 8.94 (t, *J* = 5.5 Hz, 4H, Ar*H*), 7.90-7.71 (m, 2H, Ar*H*), 7.42-7.31 (m, 4H, Ar*H*).

^{13}C NMR (100 MHz, $CDCl_3$) δ 155.6, 154.9 (minor), 138.5 (minor), 138.3, 125.2 (minor), 125.0.

HRMS (ESI/QTOF) m/z: $[M]^+$ Calcd for $C_{10}H_{10}IN_2Pd^+$ 390.8918; Found 390.8923.

Note: we propose a mixture of cis/trans isomers the chemical shifts in ^{13}C NMR do not overlap with py_2PdCl_2 complex **S4** and no chloro-derivatives, for example arising from a mixed counter anion complex, were detected by HRMS. However, other impurities cannot be ruled out.

***Cis*-bis(triphenylphosphine)palladium dinitrate (S6)**

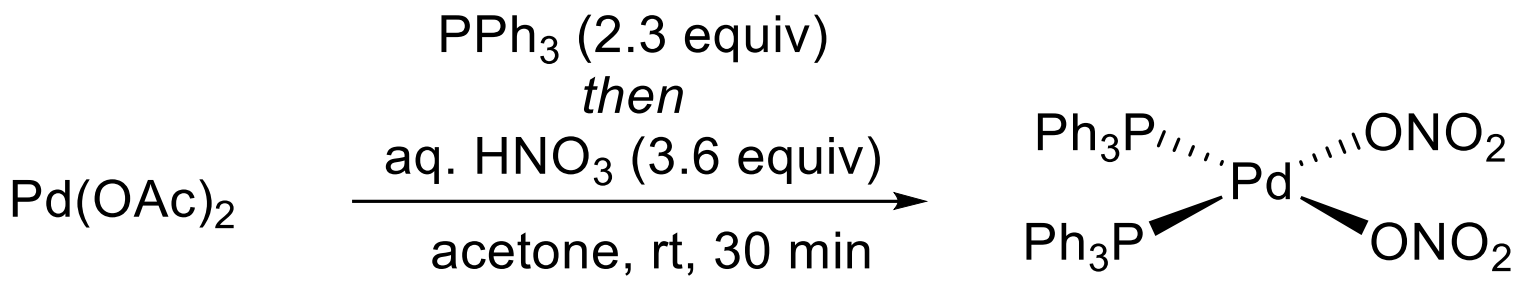

S6

Following a reported procedure,[33] to a solution of $Pd(OAc)_2$ (89.8 mg, 400 µmol, 1.00 equiv) in acetone (4.0 mL), PPh_3 (239 mg, 910 µmol, 2.27 equiv) was added at rt and allowed to stir for 5 min. Then, 60% aq. HNO_3 (140 mg, 100 µL, 1.44 mmol, 3.61 equiv) was added dropwise at rt and the mixture was allowed to stir for 30 min at rt, after which the yellow precipitate was filtered off and washed with Et_2O (3 x 5 mL) to give the title compound as a yellow solid (227 mg, 341 µmol, 85% yield).

^{1}H NMR (400 MHz, CD_2Cl_2) δ 7.56-7.46 (m, 18H), 7.30 (td, *J* = 7.9, 2.5 Hz, 12H).

^{13}C NMR (100 MHz, CD_2Cl_2) δ 134.8-134.4 (m), 132.4, 129.4-129.0 (m), 126.8 (d, *J* = 58.5 Hz).

^{31}P NMR (162 MHz, CD_2Cl_2) δ 32.31.

Note: previously reported ^{1}H NMR signals gradually disappeared upon complex dissolution in CD_2Cl_2 and appear as reported above after ca. 3 h after NMR sample preparation. ^{31}P NMR follows the ^{1}H NMR trend, but the previously reported signal appears over time while the other signal at +13.41 ppm diminishes. We propose the structure is in a dynamic equilibrium with an unidentified species (potentially cis/trans isomers or partial ligand dissociation) upon dissolution which settles after ca. 3 h to a single species, which spectral data is reported above.

^{1}H NMR evolution

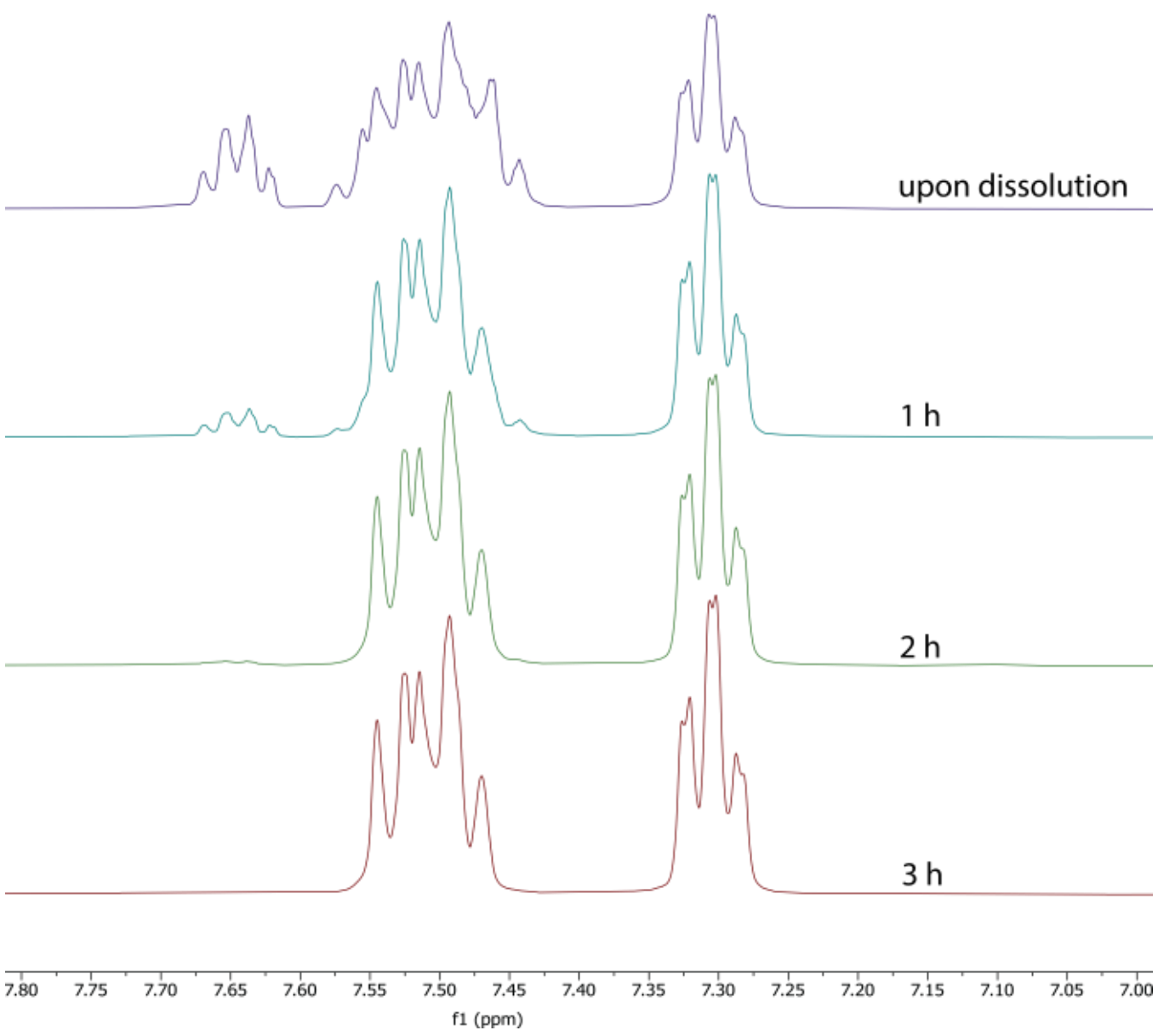

^{31}P NMR evolution

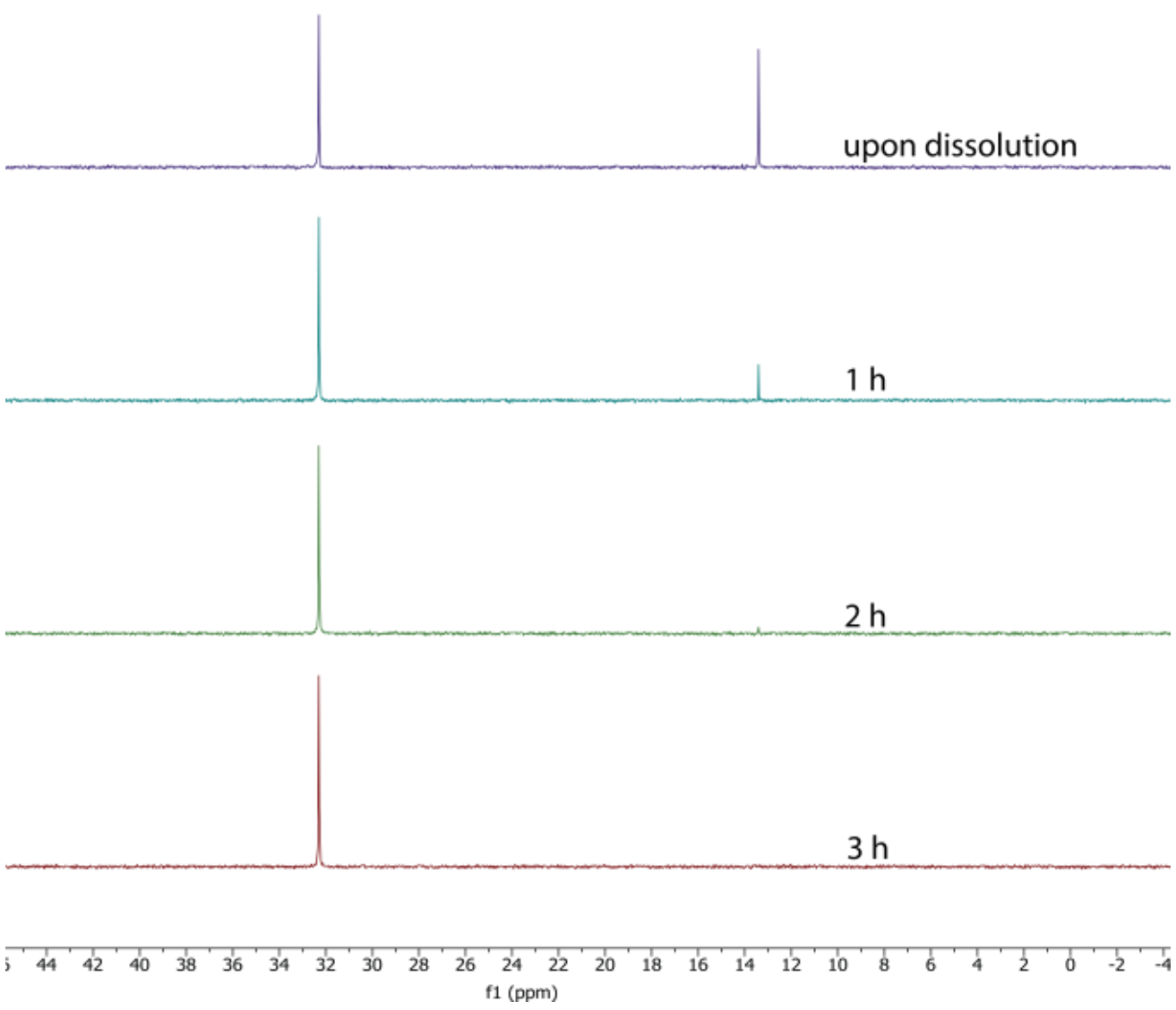

Synthesis of cyclohexyl(triphenyl)phosphonium tetrafluoroborate (P3)

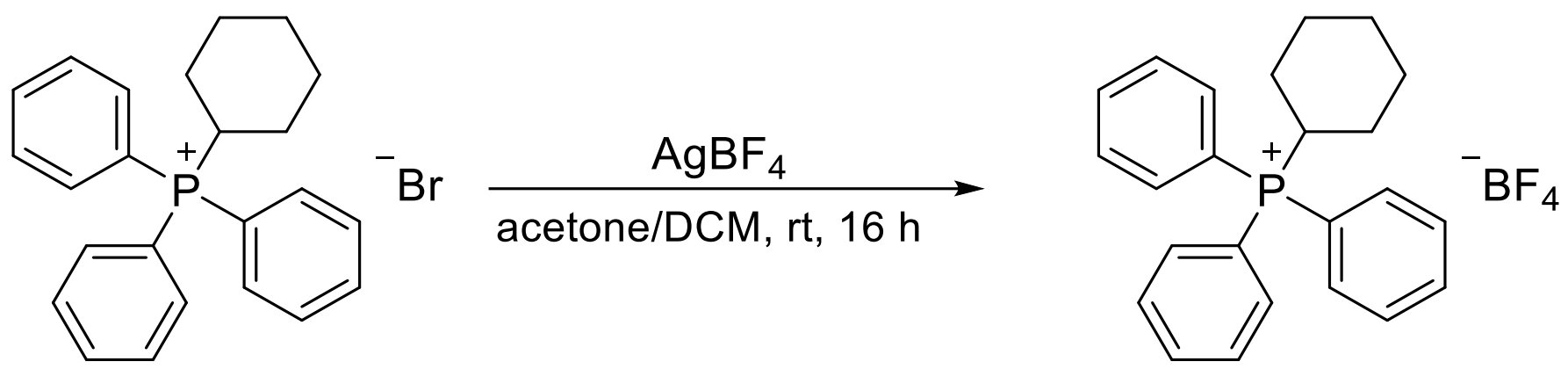

To a solution of cyclohexyl(triphenyl)phosphonium bromide (425 mg, 1.00 mmol, 1.00 equiv) in DCM (10.0 mL), a solution of $AgBF_4$ (253 mg, 1.30 mmol, 1.30 equiv) in acetone (5.00 mL) was added under N_2 atmosphere. The resulting mixture was stirred for 16 h, after which time it was filtered, concentrated, and purified by FCC (SiO_2, 0 - 10% MeOH/DCM) to give the title compound as a white solid (419 mg, 969 μmol, 97% yield).

^{1}H NMR (600 MHz, DMSO) δ 7.92-7.87 (m, 9H, Ar*H*), 7.80-7.75 (m, 6H, Ar*H*), 4.19 (q, *J* = 12.0 Hz, 1H, PC*H*), 2.08-1.98 (m, 2H, Cy), 1.79 (d, *J* = 12.6 Hz, 2H, Cy), 1.70 (d, *J* = 13.4 Hz, 1H, Cy), 1.58 (q, *J* = 13.0 Hz, 2H, Cy), 1.11-1.00 (m, 3H, Cy).

^{13}C NMR (151 MHz, DMSO) δ 135.3 (d, *J* = 3.1 Hz), 134.2 (d, *J* = 9.4 Hz), 130.8 (d, *J* = 12.2 Hz), 117.8 (d, *J* = 83.1 Hz), 29.2 (d, *J* = 46.7 Hz), 26.4 (d, *J* = 2.7 Hz), 25.7 (d, *J* = 14.0 Hz), 25.4.

^{19}F NMR (376 MHz, DMSO) δ -148.20--148.34 (m).

^{31}P NMR (162 MHz, DMSO) δ 26.23.

HRMS (ESI/QTOF) m/z: $[M]^+$ Calcd for $C_{24}H_{26}P^+$ 345.1767; Found 345.1761.

R_f = 0.52 (10% MeOH/DCM)

Starting material synthesis

1H-indol-5-ylmethanol (S9)

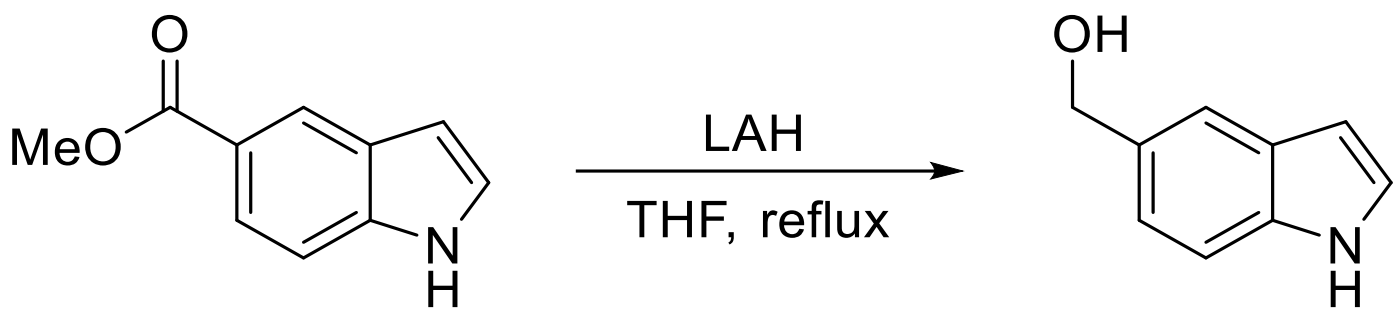

S8 **S9**

Following a reported procedure,[34] methyl 1H-indole-5-carboxylate (298 mg, 1.70 mmol, 1.00 equiv) was dissolved in dry THF (1.18 mL) at 0 °C under N_2 atmosphere. Lithium aluminum hydride (1.0 M) in THF (7.14 mL, 7.14 mmol) was added dropwise and the resulting mixture was brought to reflux for 2 h. The solution was cooled, quenched with EtOAc, water (100 mL), and DCM (100 mL). Then, 10% aqueous Rochelle's salt (100 mL) was added and filtered after stirring at rt for 1 h. The aqueous residue was extracted with ethyl acetate, washed with brine, dried with magnesium sulfate, filtered, and concentrated under reduced pressure. The crude residue was purified by FCC (30-60% EtOAc/hexane), to give 1H-indol-5-ylmethanol (175 mg, 1.19 mmol, 70% yield) as a clear oil.

^{1}H NMR (400 MHz, $CDCl_3$) δ 8.20 (s, 1H, N*H*), 7.67-7.60 (m, 1H, Ar*H*), 7.39 (d, *J* = 8.3 Hz, 1H, Ar*H*), 7.25-7.20 (m, 2H, Ar*H*), 6.56 (ddd, *J* = 3.1, 2.0, 0.9 Hz, 1H, Ar*H*), 4.77 (d, *J* = 5.1 Hz, 2H, CH_2OH), 1.58 (s, 1H, O*H*).

^{13}C NMR (100 MHz, $CDCl_3$) δ 135.5, 132.6, 128.0, 124.7, 122.0, 119.7, 111.2, 102.8, 66.4.

R_f = 0.53 (60% EtOAc/hexane)

The spectra data was consistent with the literature.[34]

2-(1H-indol-3-yl)ethane-1-thiol (S11)

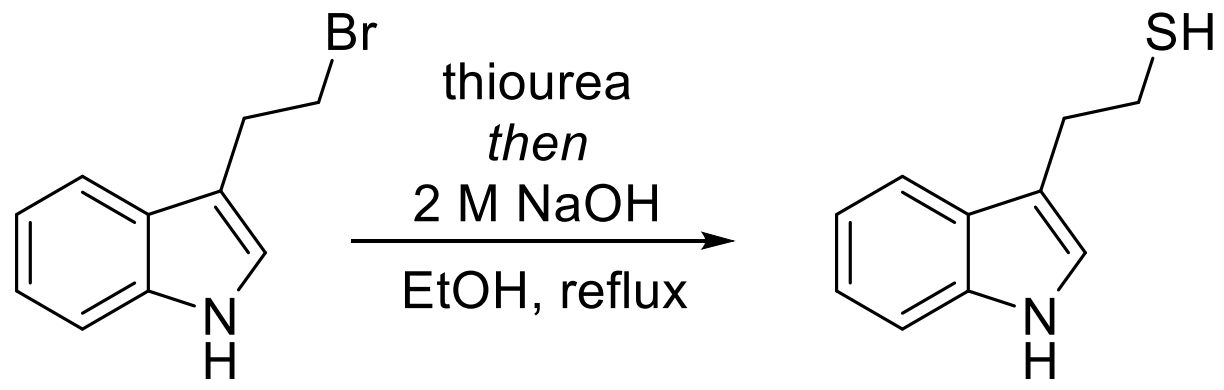

Following a reported procedure,[35] thiourea (2 mmol, 2 equiv.) was added to a solution of 3-(2-bromoethyl)-1H-indole (224 mg, 1.00 mmol, 1.00 equiv) in absolute EtOH (16 mL) and the reaction was refluxed for 16 h. The solution was then cooled to rt and a solution of 2 M NaOH (4 mmol, 4 equiv.) was added. The reaction was then refluxed for another 4 h, cooled to rt, and neutralized with 1 M HCl. The volatiles were removed under reduced pressure and then the aqueous phase was diluted with DCM (50 mL). The phases were separated and the aqueous phase was extracted with DCM (10 mL). The combined organic fractions were washed with brine (10 mL), dried over anhydrous Na_2SO_4, filtered, and concentrated under reduced pressure. The crude product was purified by FCC (2-30% EtOAc/hexane) to give the title compound (115 mg, 646 μmol, 65% yield) as an off-white solid.

^{1}H NMR (400 MHz, $CDCl_3$) δ 7.98 (s, 1H, N*H*), 7.63-7.56 (m, 1H, Ar*H*), 7.38 (dt, *J* = 8.1, 0.9 Hz, 1H, Ar*H*), 7.21 (ddd, *J* = 8.2, 7.0, 1.2 Hz, 1H, Ar*H*), 7.13 (ddd, *J* = 8.0, 7.0, 1.0 Hz, 1H, Ar*H*), 7.09-7.04 (m, 1H, Ar*H*), 3.10 (t, *J* = 7.2, 2H, CH_2CH_2SH), 2.88 (dt, *J* = 7.4, 7.4 Hz, 2H), 1.46 (t, *J* = 7.9 Hz, 1H, S*H*).

^{13}C NMR (100 MHz, $CDCl_3$) δ 136.5, 127.3, 122.3, 122.2, 119.6, 118.9, 114.4, 111.3, 30.2, 25.5.

R_f = 0.26 (10% EtOAc/hexane)

The spectral data was consistent with the literature.[35]

5-Fluoro-1-methylindole (1)

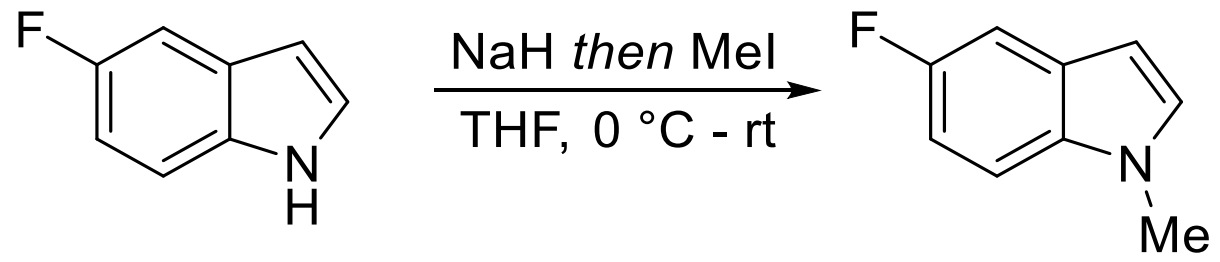

S12 **1**

Following a modified reported procedure,[36] to a solution of 5-fluoro-1H-indole (2.00 g, 14.8 mmol, 1.00 equiv) in dry THF (15.0 mL) in an oven-dried flask, sodium hydride (710 mg, 17.8 mmol, 1.20 equiv) was added in portions at 0 °C. The mixture was allowed to warm to rt and stirred for 30 min. The resulting solution was cooled again to 0 °C and iodomethane (2.73 g, 1.20 mL, 19.2 mmol, 1.30 equiv) was added. The mixture was stirred for 2 h at rt until completion (TLC, 10% Et_2O/hexane, R_f = 0.50). The reaction mixture was then quenched with sat. aq. NH_4Cl at 0 °C and extracted with Et_2O (3x). The organic layers were washed with brine, dried over $MgSO_4$, and concentrated under reduced pressure. The crude residue was purified by FCC (25 g SiO_2, 0 - 20% Et_2O/hexane) to give the titled product (1.99 g, 13.3 mmol, 90% yield) as a transparent oil which solidified to a white crystalline solid upon cooling to 4 °C.

^{1}H NMR (400 MHz, $CDCl_3$) δ 7.31 (dd, *J* = 9.7, 2.5 Hz, 1H, Ar*H*), 7.26 (dd, *J* = 8.9, 4.3 Hz, 1H, Ar*H*), 7.12 (d, *J* = 3.1 Hz, 1H, Ar*H*), 7.01 (td, *J* = 9.1, 2.5 Hz, 1H, Ar*H*), 6.48 (dd, *J* = 3.1, 0.9 Hz, 1H, Ar*H*), 3.81 (s, 3H, CH_3).

^{13}C NMR (100 MHz, $CDCl_3$) δ 158.0 (d, *J* = 233.7 Hz), 133.5, 130.5, 128.8 (d, *J* = 10.3 Hz), 110.0 (d, *J* = 18.2 Hz), 109.9 (d, *J* = 1.8 Hz), 105.6 (d, *J* = 23.3 Hz), 101.0 (d, *J* = 4.7 Hz), 33.2.

^{19}F NMR (376 MHz, $CDCl_3$) δ -125.77.

R_f = 0.50 (10% Et_2O/hexane).

The spectral data was consistent with the literature.[36]

1-butyl-5-fluoro-1H-indole (S13)

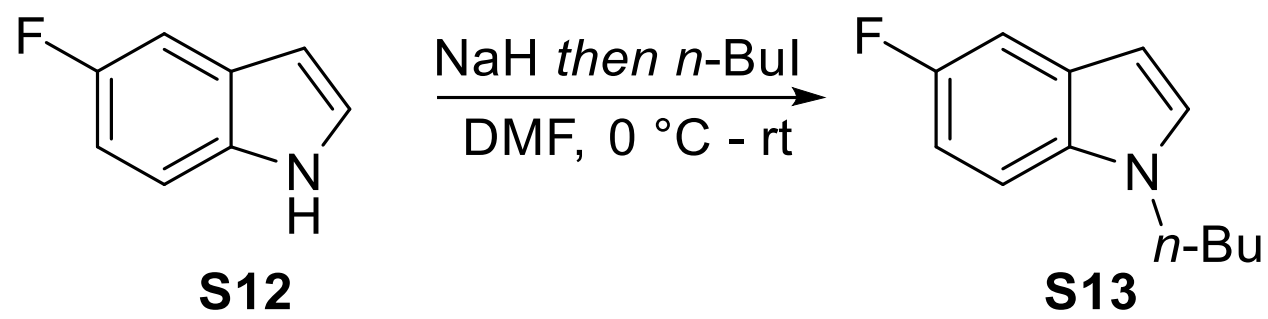

S12 **S13**

To a solution of 5-fluoro-1H-indole (1.54 g, 11.4 mmol, 1.00 equiv) in dry DMF (15.0 mL) under N_2, sodium hydride (547 mg, 13.7 mmol, 1.20 equiv) was added portion wise at 0 °C and the resulting mixture was stirred at 0 °C for 30 min. Then, 1-iodobutane (3.15 g, 1.94 mL, 17.1 mmol, 1.50 equiv) was added at 0 °C and the mixture was allowed to stir at rt for 16 h. The reaction was then diluted with water (50 mL) and extracted with EtOAc (3 x 50 mL). The combined organic layers were washed with water (2 x 50 mL), 1 M HCl (50 mL), and brine (50 mL), dried over $MgSO_4$, filtered, and concentrated under reduced pressure. The crude residue was purified by FCC (1-10% EtOAc/hexane) to give the title compound as a light-yellow oil (2.07 g, 10.8 mmol, 95% yield).

^{1}H NMR (400 MHz, $CDCl_3$) δ 7.33-7.20 (m, 2H, Ar*H*), 7.13 (dd, *J* = 3.1, 1.2 Hz, 1H, Ar*H*), 6.96 (td, *J* = 8.9, 2.1 Hz, 1H, Ar*H*), 6.44 (dq, *J* = 3.1, 0.9 Hz, 1H, Ar*H*), 4.10 (t, *J* = 7.1 Hz, 2H, NCH_2), 1.88-1.75 (m, 2H, NCH_2CH_2), 1.34 (hd, *J* = 7.4, 1.4 Hz, 2H, $NCH_2CH_2CH_2$), 0.95 (ddd, *J* = 8.4, 7.0, 1.6 Hz, 3H, CH_3).

^{13}C NMR (100 MHz, $CDCl_3$) δ 157.9 (d, *J* = 233.6 Hz), 132.8, 129.5, 128.8 (d, *J* = 10.2 Hz), 110.1 (d, *J* = 9.8 Hz), 109.8 (d, *J* = 26.3 Hz), 105.7 (d, *J* = 23.2 Hz), 100.9 (d, *J* = 4.7 Hz), 46.5, 32.5, 20.3, 13.8.

^{19}F NMR (376 MHz, $CDCl_3$) δ -125.90.

HRMS (Sicrit plasma/LTQ-Orbitrap) m/z: $[M + H]^+$ Calcd for $C_{12}H_{15}FN^+$ 192.1183; Found 192.1181.

IR (ν_{max}, cm^{-1}) 2959 (m), 2874 (w), 1488 (s), 1450 (m), 1342 (w), 1227 (m), 1131 (m), 949 (m), 795 (m), 717 (m).

R_f = 0.60 (10% EtOAc/hexane).

1-(5-fluoro-1H-indol-1-yl)ethan-1-one (S14)

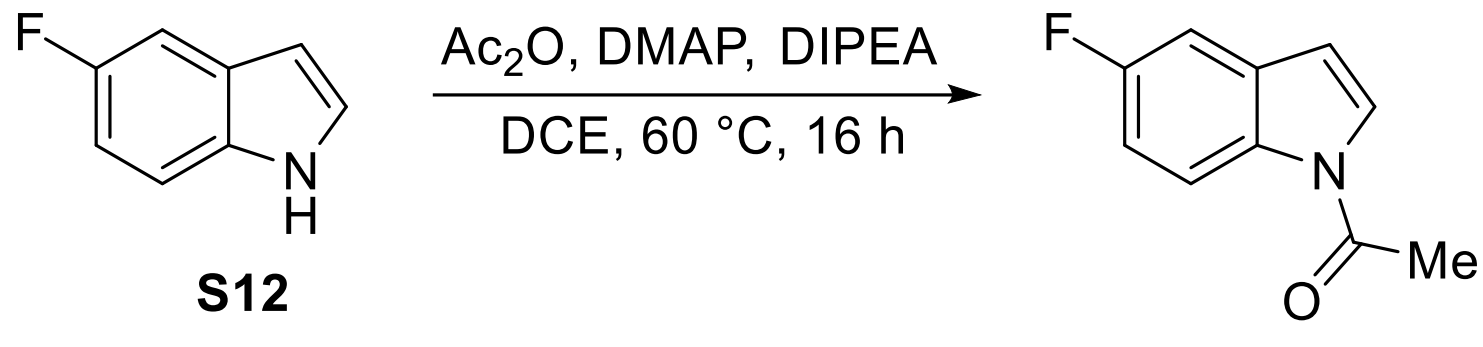

DIPEA (1.31 g, 1.80 mL, 12.9 mmol, 1.48 equiv), acetic anhydride (3.45 g, 3.19 mL, 33.8 mmol, 3.89 equiv) *N,N*-dimethylpyridin-4-amine (203 mg, 1.66 mmol, 0.191 equiv) were added to a solution of 5-fluoro-1H-indole (1.18 g, 8.70 mmol, 1.00 equiv) in 1,2-dichloroethane (20.0 mL), The mixture was stirred at 60 °C for 16 h. Upon cooling to rt, the reaction was diluted with sat. aq. NH_4Cl (40 mL) and extracted with EtOAc (3 x 40 mL). The combined organic phase was washed with brine, dried over $MgSO_4$, filtered, and concentrated under reduced pressure. The crude residue was purified by FCC (10 - 30% Et_2O/hexane) to give the title compound as a white solid (1.03 g, 5.79 mmol, 67% yield).

^{1}H NMR (400 MHz, $CDCl_3$) δ 8.41 (dd, *J* = 9.1, 4.7 Hz, 1H, Ar*H*), 7.45 (d, *J* = 3.8 Hz, 1H, Ar*H*), 7.21 (dd, *J* = 8.7, 2.6, 0.6 Hz, 1H, Ar*H*), 7.07 (tdd, *J* = 9.1, 2.6, 0.5 Hz, 1H, Ar*H*), 6.60 (dd, *J* = 3.8, 0.8 Hz, 1H, Ar*H*), 2.63 (s, 3H, CH_3).

^{13}C NMR (100 MHz, $CDCl_3$) δ 168.5, 159.8 (d, *J* = 240.0 Hz), 132.1, 131.5 (d, *J* = 10.2 Hz), 126.8, 117.8 (d, *J* = 9.1 Hz), 113.0 (d, *J* = 24.7 Hz), 109.0 (d, *J* = 3.9 Hz), 106.5 (d, *J* = 23.9 Hz), 23.9.

^{19}F NMR (376 MHz, $CDCl_3$) δ -119.39.

HRMS (nanochip-ESI/LTQ-Orbitrap) m/z: $[M + H]^+$ Calcd for $C_{10}H_9FNO^+$ 178.0663; Found 178.0665.

IR (ν_{max}, cm^{-1}) 3132 (w), 1711 (s), 1466 (s), 1446 (m), 1384 (s), 1333 (s), 1258 (s), 1207 (s), 1138 (m), 953 (m), 810 (m).

R_f = 0.22 (20% Et_2O/hexane).

Benzenediazonium triflate (S16)

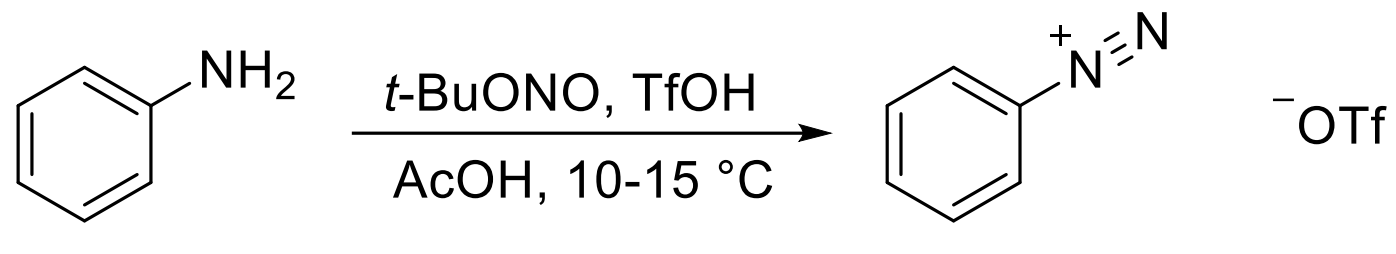

Following a reported procedure,[37] a precooled (10-15 °C) solution of *t*-BuONO (247 mg, 285 μL, 2.40 mmol, 1.20 equiv) and aniline (186 mg, 183 μL, 2.00 mmol, 1.00 equiv) in glacial AcOH (8.00 mL) was added dropwise to a precooled solution (10-15 °C) of TfOH (360 mg, 212 μL, 2.40 mmol, 1.20 equiv) in glacial AcOH (12.0 mL). The reaction mixture was stirred for 30 min at 10-15 °C. Then, Et_2O (200 mL) was added. The precipitated diazonium salt was collected by filtration and dried under vacuum to give the title compound as a beige solid (458 mg, 1.80 mmol, 90% yield).

^{1}H NMR (600 MHz, DMSO) δ 8.73-8.62 (m, 2H), 8.30-8.23 (m, 1H), 8.02-7.91 (m, 2H).

^{13}C NMR (151 MHz, DMSO) δ 140.8, 132.7, 131.2, 120.7 (q, J = 322.3 Hz), 116.1.

Note: The compound does not appear fully stable at room temperature in d_6-DMSO.

The spectral data were consistent with the literature.[37]

Mesityl-λ^3-iodanediyl diacetate (S18)

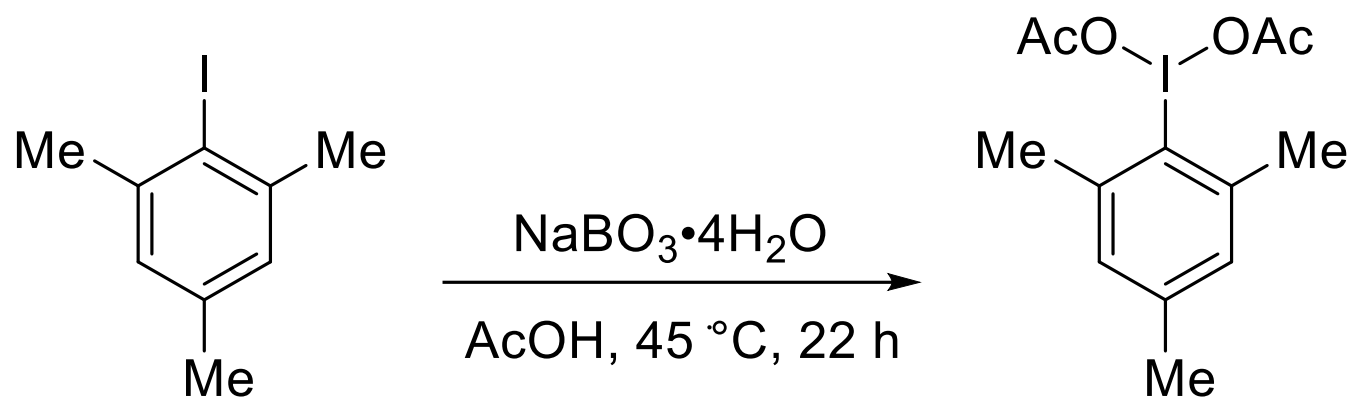

S17 **S18**

Following a reported procedure,[38] mesityl iodide (6.15 g, 25.0 mmol, 1.00 equiv) and glacial AcOH (227 mL) were added to a flask, followed by $NaBO_3$•$4H_2O$ (38.5 g, 250 mmol, 10.0 equiv). The resulting mixture was heated at 45 °C for 22 h. The mixture was then concentrated under reduced pressure (50 °C, 45 mbar) to approximately half its volume. H_2O (250 mL) was added, and the mixture was stirred vigorously for 10 min. Then, it filtered to collect the solid. The white solid was rinsed with H_2O (3 × 25 mL) and left to dry in air for ca. 10 min (with vacuum applied). The damp solid was then dissolved in 1:1 AcOH/Ac_2O (16 mL) at 80 °C. The solvent was then removed under reduced pressure (50 °C, 10 mbar) to give a white solid, which was washed with Et_2O (3 × 5 mL) and dried under vacuum to give the title compound as a white, crystalline solid (5.66 g, 15.5 mmol, 62% yield).

^{1}H NMR (400 MHz, $CDCl_3$) δ 7.11-7.07 (m, 2H, Ar*H*), 2.71 (s, 6H, ArC*H*$_3$), 2.35 (s, 3H, ArC*H*$_3$), 1.97 (s, 6H, COC*H*$_3$).

^{13}C NMR (100 MHz, $CDCl_3$) δ 176.6, 143.3, 141.5, 129.8, 129.1, 26.9, 21.4, 20.5.

The spectral data was in accordance with the literature.[38]

Phenyl-(2,4,6-trimethylphenyl)iodonium tetrafluoroborate (S19)

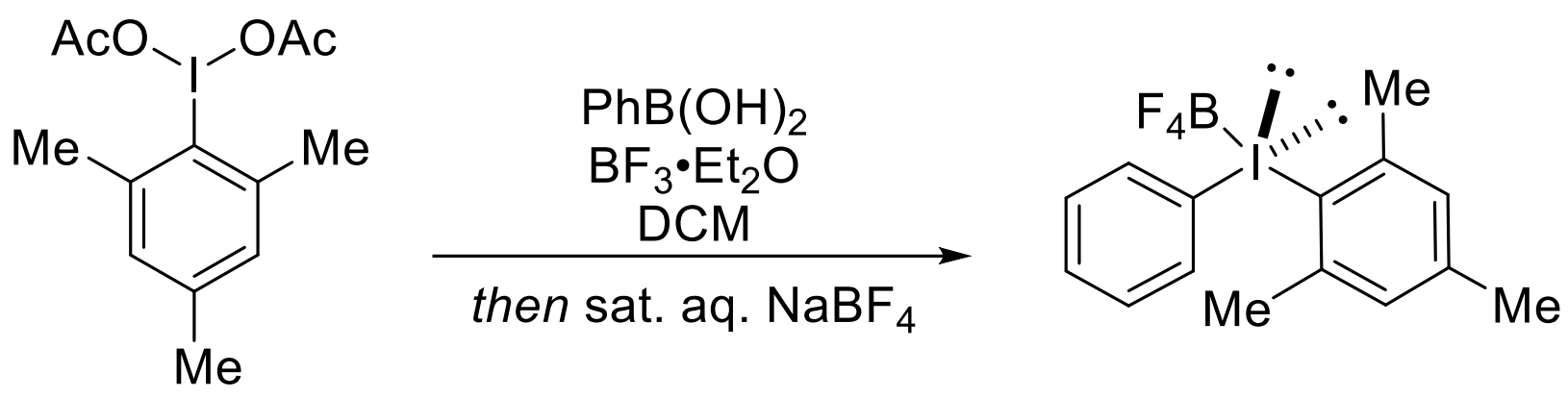

S18 **S19**

Following a reported procedure,[39] to a stirred mixture of phenylboronic acid (703 mg, 5.77 mmol, 1.05 equiv) and BF_3•Et_2O (857 mg, 746 μL, 6.04 mmol, 1.10 equiv) in DCM (27.5 mL), a solution of [acetyloxy(phenyl)-lambda3-iodanyl] acetate (2.00 g, 5.49 mmol, 1.00 equiv) in DCM (100 mL) was added at 0 °C under N_2 atmosphere, and the resulting mixture was stirred for 1.5 h at 0 °C. After the addition of sat. aq. $NaBF_4$ (24.1 g, 17.0 mL, 110 mmol, 20.0 equiv), the mixture was stirred for 30 min at rt. The reaction mixture was then poured into water (30 mL) and extracted with DCM (3 x 30 mL). The organic layer was concentrated under reduced pressure to give a solid residue, which was washed with Et_2O (3 x 20 mL) at -78 °C. Recrystallisation from DCM/hexane gave the title compound as a white crystalline solid (1.21 g, 2.95 mmol, 54% yield).

^{1}H NMR (400 MHz, DMSO) δ 8.01-7.94 (m, 2H, Ar*H*), 7.68-7.59 (m, 1H, Ar*H*), 7.50 (t, *J* = 7.8 Hz, 2H, Ar*H*), 7.22 (s, 2H, Ar*H*), 2.60 (s, 6H, ArCH_3), 2.30 (s, 3H, ArCH_3).

^{13}C NMR (100 MHz, DMSO) δ 143.1, 141.6, 134.5, 131.9, 131.8, 129.8, 122.6, 114.5, 26.3, 20.5.

^{19}F NMR (376 MHz, DMSO) δ -148.16--148.32 (m).

The spectral data was consistent with the literature.[39]

Diphenyliodonium tetrafluoroborate (S21)

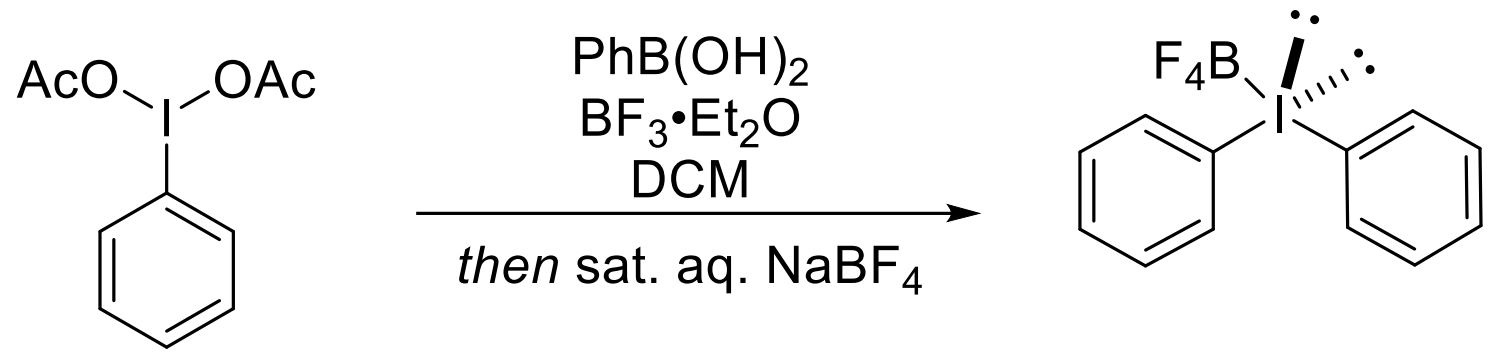

S20 S21

Following a reported procedure, to a stirred mixture of phenylboronic acid (2.56 g, 21.0 mmol, 1.1 equiv) and BF_3•OEt_2 (3.12 g, 2.71 mL, 22.0 mmol, 1.1 equiv) in DCM (100 mL), a solution of PIDA (6.44 g, 20.0 mmol, 1.0 equiv) in DCM (100 mL) was added at 0 °C under N_2 atmosphere, and the mixture was stirred for 1.5 h at 0 °C. After the addition of saturated aqueous $NaBF_4$ solution (87.8 g, 61.9 mL, 400 mmol, 20.0 equiv). The mixture was stirred for 30 min at rt. The reaction mixture was then poured into water and extracted with DCM. The organic layer was concentrated under reduced pressure to give a solid residue, which was washed with Et_2O at -78 °C (4x). Recrystallisation from hexane-DCM (2x) afforded Ph_2IBF_4 (5.86 g, 15.9 mmol, 80% yield) as an off-white crystalline powder.

^{1}H NMR (400 MHz, DMSO) δ 8.28-8.20 (m, 4H, Ar*H*), 7.70-7.61 (m, 2H, Ar*H*), 7.58-7.48 (m, 4H, Ar*H*).

^{13}C NMR (100 MHz, DMSO) δ 135.2, 132.1, 131.8, 116.5.

^{19}F NMR (376 MHz, DMSO) δ -148.15--148.24 (m).

The spectral data was consistent with the literature.[40]

General procedure S7 (GP-S7) for iodonium salt synthesis

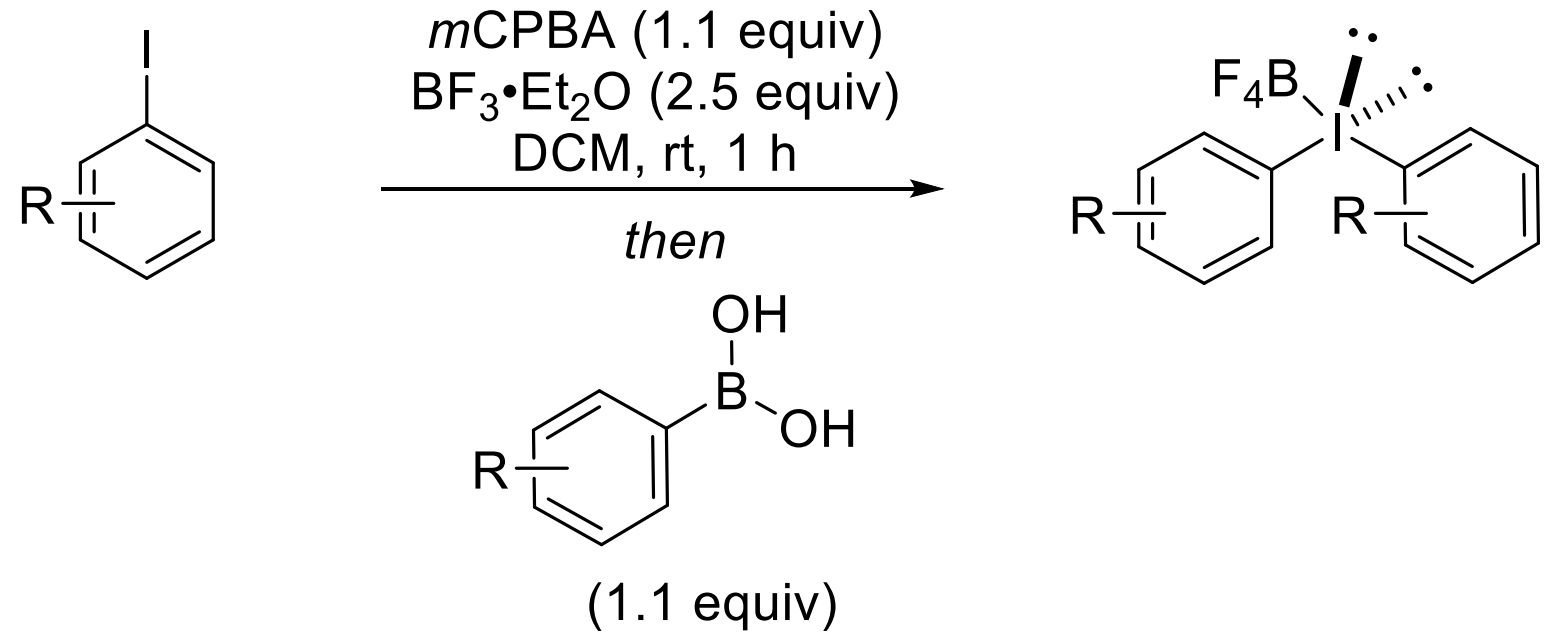

Following a reported procedure,[41] *m*-Chloroperbenzoic acid (77% active oxidant, 1.35 g, 6.00 mmol) was dissolved in DCM (20 mL). To the solution was added iodoarene derivative (5.40 mmol) followed by slow addition of BF_3•OEt_2 (1.70 mL, 13.5 mmol) at room temperature. The resulting yellow solution was stirred at rt for 45 min and then cooled to 0 °C. Arylboronic acid (6.00 mmol) was added portion wise over 10 min. After stirring for 30 min at rt, the crude reaction mixture was applied on a silica plug (ca. 12 g) and eluted with DCM (120 mL) followed by 5% MeOH in DCM (250 mL). The obtained solution was concentrated, and diethyl ether (20 mL) was added to the residue to induce precipitation of the product. The mixture was allowed to stir for 15 min and then the ether phase was decanted. The solid was washed twice with diethyl ether (2 × 20 mL) and then dried under high vacuum to afford the diaryliodonium salt.

Diphenyliodonium tetrafluoroborate (S21)

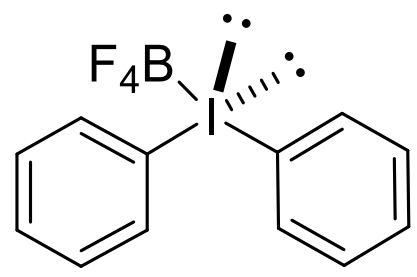

S21

Following **GP-S7** with iodobenzene (1.10 g, 601 µL, 5.39 mmol, 1.00 equiv) and phenylboronic acid (733 mg, 6.01 mmol, 1.12 equiv) to give the title compound (1.57 g, 4.28 mmol, 79% yield) as a white solid.

^{1}H NMR (400 MHz, DMSO) δ 8.28-8.20 (m, 4H, Ar*H*), 7.70-7.61 (m, 2H, Ar*H*), 7.58-7.48 (m, 4H, Ar*H*).

^{13}C NMR (100 MHz, DMSO) δ 135.2, 132.1, 131.8, 116.5.

^{19}F NMR (376 MHz, DMSO) δ -148.12--148.31 (m).

The spectral data was consistent with the literature.[41]

Di-p-tolyliodonium tetrafluofroborate (S22)

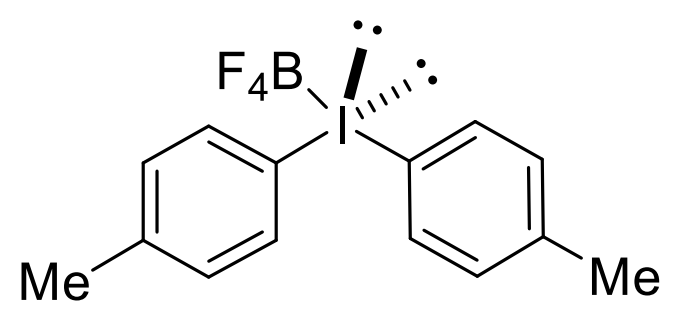

S22

Following **GP-S7** with 1-iodo-4-methylbenzene (1.18 g, 5.40 mmol, 1.00 equiv) and p-tolylboronic acid (819 mg, 6.02 mmol, 1.11 equiv) to give the title compound (1.57 g, 3.12 mmol, 58% yield) as a white solid.

^{1}H NMR (400 MHz, DMSO) δ 8.28-8.22 (m, 4H, Ar*H*), 7.55-7.46 (m, 4H, Ar*H*), 2.50 (s, 6H, C*H₃*).

^{13}C NMR (100 MHz, DMSO) δ 142.5, 135.0, 132.3, 113.0, 20.8.

^{19}F NMR (376 MHz, DMSO) δ -148.03--148.36 (m).

The spectral data were consistent with the literature.[41]

Bis(4-fluorophenyl)iodonium tetrafluoroborate (S23)

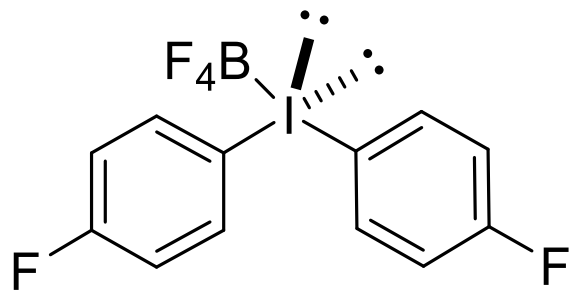

S23

Following **GP-S7** with 1-fluoro-4-iodo-benzene (1.20 g, 623 μL, 5.40 mmol, 1.00 equiv) and (4-fluorophenyl)boronic acid (842 mg, 6.02 mmol, 1.11 equiv) to give the title compound (1.56 g, 3.86 mmol, 72% yield) as a white solid.

^{1}H NMR (400 MHz, DMSO) δ 8.40-8.25 (m, 4H, Ar*H*), 7.47-7.36 (m, 4H, Ar*H*).

^{13}C NMR (100 MHz, DMSO) δ 164.0 (d, *J* = 251.7 Hz), 138.0 (d, *J* = 9.0 Hz), 119.3 (d, *J* = 22.8 Hz), 111.2 (d, *J* = 3.0 Hz).

^{19}F NMR (376 MHz, DMSO) δ -106.59 (2F), -148.01--148.33 (m, 4F).

The spectral data was consistent with the literature.[41]

Bis(4-(trifluoromethyl)phenyl)iodonium tetrafluoroborate (S24)

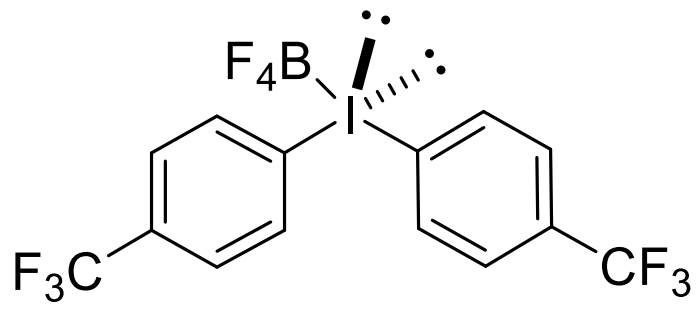

S24

Following **GP-S7** with 1-iodo-4-(trifluoromethyl)benzene (1.47 g, 5.40 mmol, 1.00 equiv) and [4-(trifluoromethyl)phenyl]boronic acid (1.14 g, 6.02 mmol, 1.11 equiv) to give the title compound (1.66 g, 3.29 mmol, 61% yield) as a white solid.

^{1}H NMR (400 MHz, DMSO) δ 8.51 (d, *J* = 8.2 Hz, 4H, Ar*H*), 7.99-7.88 (m, 4H, Ar*H*).

^{13}C NMR (100 MHz, DMSO) δ 136.3, 132.1 (q, *J* = 32.6 Hz), 128.5 (q, *J* = 3.7 Hz), 123.4 (q, *J* = 273.1 Hz), 120.9 (q, *J* = 1.2 Hz).

^{19}F NMR (376 MHz, DMSO) δ -61.72 (6F), -148.09--148.37 (m, 4F).

The spectral data was consistent with the literature.[41]

5-fluoro-1-(phenylsulfonyl)-1H-indole (S25)

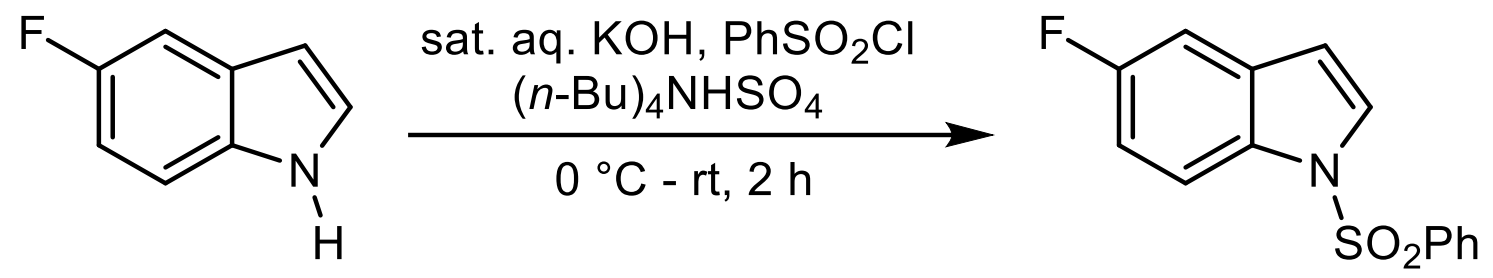

S12 **S25**

Following a reported procedure,[42] to a solution of 5-fluoro-1H indole (3.38 g, 25.0 mmol, 1.00 equiv) and tetrabutylammonium hydrogen sulfate (849 mg, 2.50 mmol, 0.100 equiv) in DCM (62.5 mL), sat. aq. KOH (4.21 g of KOH, 75.0 mmol, 3.00 equiv) and benzenesulfonyl chloride (6.62 g, 4.79 mL, 37.5 mmol, 1.50 equiv) were added at 0 °C and the resulting mixture was stirred for 2 h at rt. After this time, the organic layer was separated and washed with sat. aq. NH_4Cl (3 x 50 mL). The collected organic layer was dried over anhydrous Na_2SO_4, filtered, and concentrated under reduced pressure. The crude residue was purified by FCC (SiO_2, 2-10% EtOAc/hexane) to give the title compound (6.55 g, 23.8 mmol, 95% yield) as an off-white solid.

^{1}H NMR (400 MHz, $CDCl_3$) δ 7.94 (dd, *J* = 9.1, 4.4 Hz, 1H, Ar*H*), 7.88-7.78 (m, 2H, Ar*H*), 7.60 (d, *J* = 3.7 Hz, 1H, Ar*H*), 7.58-7.51 (m, 1H, Ar*H*), 7.44 (dd, *J* = 8.5, 7.1 Hz, 2H, Ar*H*), 7.18 (dd, *J* = 8.7, 2.6 Hz, 1H, Ar*H*), 7.04 (td, *J* = 9.1, 2.6 Hz, 1H, Ar*H*), 6.63 (d, *J* = 3.7 Hz, 1H, Ar*H*).

^{13}C NMR (100 MHz, $CDCl_3$) δ 159.8 (d, *J* = 240.2 Hz), 138.2, 134.1, 131.9 (d, *J* = 10.2 Hz), 131.4, 129.5, 128.1, 126.8, 114.7 (d, *J* = 9.5 Hz), 112.8 (d, *J* = 25.7 Hz), 109.3 (d, *J* = 4.1 Hz), 107.1 (d, *J* = 24.0 Hz).

^{19}F NMR (376 MHz, $CDCl_3$) δ -119.78.

R_f = 0.14 (3% EtOAc/hexane).

The spectral data was consistent with the literature.[36]

2-deuterio-5-fluoro-1H-indole (*d*-S12)

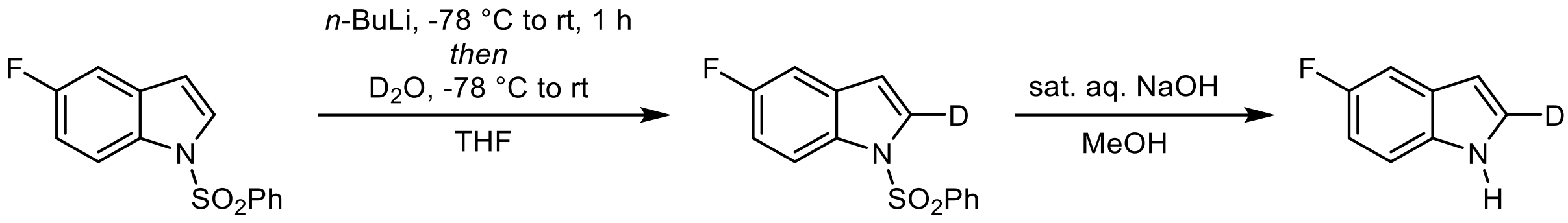

S25 **d-S25** **d-S12**

Following a reported procedure,[42] to a solution of 1-(benzenesulfonyl)-5-fluoro-indole (6.55 g, 23.8 mmol, 1.00 equiv) in THF (68.0 mL), a solution of *n*-BuLi (2.98 g, 18.6 mL, 46.5 mmol, 2.50 M, 2.00 equiv) was added dropwise under N_2 atmosphere at -78 °C. The solution was then allowed to warm to rt and stirred for 1 h. The mixture was cooled to -78 °C followed by the addition of D_2O (1.68 g, 1.68 mL, 93.0 mmol, 4.00 equiv). The mixture was warmed to rt and solid K_2CO_3 (6.43 g, 46.5 mmol, 2.00 equiv) was added. After stirring with dry Et_2O (70 mL) for 30 min, the organic layer was separated (H_2O may be added to improve separation), dried over anhydrous Na_2SO_4, filtered, and concentrated under reduced pressure and used in the next step without further purification.

To a solution of the crude residue in MeOH (78.0 mL), sat. aq. NaOH (9.26 g of NaOH, 232 mmol, 10.0 equiv) was added at rt and the resulting mixture was refluxed for 3 h. After that time, sat. aq. NH_4Cl solution was added. The mixture was extracted with EtOAc (3 x 50 mL), dried over anhydrous Na_2SO_4, filtered, and concentrated under reduced pressure. The crude residue was purified by FCC (SiO_2, 2-10% EtOAc/hexane) to give 2-deuterio-5-fluoro-1H-indole (3.07 g, 22.5 mmol, 95% yield over 2 steps, 87% D) as an off-white solid. The deuterium content was determined by ^1H qNMR and confirmed by HRMS (see KIE measurements for method).

^{1}H NMR (400 MHz, DMSO) δ 11.16 (s, 1H, N*H*), 7.43-7.34 (m, 1.13H, Ar*H*), 7.28 (dd, *J* = 10.1, 2.6 Hz, 1H, Ar*H*), 6.91 (td, *J* = 9.2, 2.5 Hz, 1H, Ar*H*), 6.41 (d, *J* = 1.9 Hz, 1H, Ar*H*).

^{13}C NMR (100 MHz, DMSO) δ 156.9 (d, *J* = 230.7 Hz), 132.5, 127.8 (d, *J* = 10.3 Hz), 127.44-126.75 (m), 127.3 (H-indole), 112.2 (d, *J* = 9.9 Hz), 109.0 (d, *J* = 26.1 Hz), 104.4 (d, *J* = 23.0 Hz), 101.2 (d, *J* = 4.7 Hz), 101.0 (d, *J* = 4.7 Hz, H-indole).

^{19}F NMR (376 MHz, DMSO) δ -125.61.

R_f = 0.19 (3% EtOAc/hexane).

HRMS (nanochip-ESI/LTQ-Orbitrap) m/z: $[M + H]^+$ Calcd for $C_8H_8FN^+$ 137.0635; Found 137.0623.

References

(1) Usteri, M. E.; Giannakakis, G.; Bugaev, A.; Pérez-Ramírez, J.; Mitchell, S. Understanding and Controlling Reactivity Patterns of $Pd_1@C_3N_4$-Catalyzed Suzuki–Miyaura Couplings. *ACS Catal.* **2024**, 12635–12646. doi.org/10.1021/acscatal.4c03531

(2) Abdala, P. M.; Safonova, O. V.; Wiker, G.; Beek, W. van; Emerich, H.; Bokhoven, J. A. van; Sá, J.; Szlachetko, J.; Nachtegaal, M. Scientific Opportunities for Heterogeneous Catalysis Research at the SuperXAS and SNBL Beam Lines. *CHIMIA* **2012**, *66* (9), 699–699. doi.org/10.2533/chimia.2012.699

(3) Clark, A. H.; Imbao, J.; Frahm, R.; Nachtegaal, M. ProQEXAFS: A Highly Optimized Parallelized Rapid Processing Software for QEXAFS Data. *J. Synchrotron Radiat.* **2020**, *27* (2), 551–557. doi.org/10.1107/S1600577519017053

(4) Ravel, B.; Newville, M. ATHENA, ARTEMIS, HEPHAESTUS: Data Analysis for X-Ray Absorption Spectroscopy Using IFEFFIT. *J. Synchrotron Radiat.* **2005**, *12* (4), 537–541. doi.org/10.1107/S0909049505012719

(5) Solvent Effects on the Rates of Homogeneous Chemical Reactions. In *Solvents and Solvent Effects in Organic Chemistry*; John Wiley & Sons, Ltd, 2010; pp 165–357. doi.org/10.1002/9783527632220.ch5

(6) Sherwood, J.; H. Clark, J.; S. Fairlamb, I. J.; M. Slattery, J. Solvent Effects in Palladium Catalysed Cross-Coupling Reactions. *Green Chem.* **2019**, *21* (9), 2164–2213. doi.org/10.1039/C9GC00617F

(7) Bishop, R.; Majumdar, D.; Boons, G.-J. Acetonitrile. In *Encyclopedia of Reagents for Organic Synthesis*; John Wiley & Sons, Ltd, 2004. doi.org/10.1002/047084289X.ra017.pub2

(8) Dreher, E.-L.; Beutel, K. K.; Myers, J. D.; Lübbe, T.; Krieger, S.; Pottenger, L. H. Chloroethanes and Chloroethylenes. In *Ullmann's Encyclopedia of Industrial Chemistry*; John Wiley & Sons, Ltd, 2014; pp 1–81. doi.org/10.1002/14356007.o06_o01.pub2

(9) Chatterjee, R.; Sivananda, C.; Sahoo, M.; Behera, D.; Nag, S.; Pal, P. A Thermo-Gravimetric Approach for Quantification of Carbon Sources from Coal-Char and Coke Mixture of Interests. *ISIJ Int.* **2023**, *63* (6), 1011–1016. doi.org/10.2355/isijinternational.ISIJINT-2022-563

(10) Giannakakis, G.; Usteri, M. E.; Bugaev, A.; Ruiz-Ferrando, A.; Faust Akl, D.; López, N.; Fantasia, S.; Püntener, K.; Pérez-Ramírez, J.; Mitchell, S. Reactivity and Mechanism of Recoverable $Pd_1@C_3N_4$ Single-Atom Catalyst in Buchwald–Hartwig Aminations. *ACS Catal.* **2025**, *15* (1), 284–295. doi.org/10.1021/acscatal.4c05134

(11) Lane, B. S.; Brown, M. A.; Sames, D. Direct Palladium-Catalyzed C-2 and C-3 Arylation of Indoles: A Mechanistic Rationale for Regioselectivity. *J. Am. Chem. Soc.* **2005**, *127* (22), 8050–8057. doi.org/10.1021/ja043273t

(12) Behrens, H.; Luksch, P. A Bibliometric Study in Crystallography. *Acta Crystallogr. B* **2006**, *62* (6), 993–1001. doi.org/10.1107/S0108768106030278

(13) Belsky, A.; Hellenbrandt, M.; Karen, V. L.; Luksch, P. New Developments in the Inorganic Crystal Structure Database (ICSD): Accessibility in Support of Materials Research and Design. *Acta Crystallogr. B* **2002**, *58* (3), 364–369. doi.org/10.1107/S0108768102006948

(14) Zagorac, D.; Müller, H.; Ruehl, S.; Zagorac, J.; Rehme, S. Recent Developments in the Inorganic Crystal Structure Database: Theoretical Crystal Structure Data and Related Features. *J. Appl. Crystallogr.* **2019**, *52* (5), 918–925. doi.org/10.1107/S160057671900997X

(15) Standards, U. S. N. B. of. *Standard X-Ray Diffraction Powder Patterns*; U. S. Department of Commerce, National Bureau of Standards, 1953.
(16) Waser, J.; Levy, H. A.; Peterson, S. W. The Structure of PdO. *Acta Crystallogr.* **1953**, *6* (7), 661–663. doi.org/10.1107/S0365110X53001800
(17) Smolentsev, A. I.; Gubanov, A. I.; Zadesenets, A. V.; Plyusnin, P. E.; Baidina, I. A.; Korenev, S. V. Structures of Tetraammine Salts $[Pt(NH_3)_4](NO_3)_2$, $[Pd(NH_3)_4](NO_3)_2$, and $[Pd(NH_3)_4]F_2$ H_2O. *J. Struct. Chem.* **2010**, *51* (4), 709–713. doi.org/10.1007/s10947-010-0104-1
(18) Thiele, G.; Brodersen, K.; Kruse, E.; Holle, B. Die Polymorphie des Palladium (II)-jodids. *Naturwissenschaften* **1967**, *54* (23), 615–615. doi.org/10.1007/BF00591424
(19) Deprez, N. R.; Kalyani, D.; Krause, A.; Sanford, M. S. Room Temperature Palladium-Catalyzed 2-Arylation of Indoles. *J. Am. Chem. Soc.* **2006**, *128* (15), 4972–4973. doi.org/10.1021/ja060809x
(20) Rossi, K.; Ruiz-Ferrando, A.; Akl, D. F.; Abalos, V. G.; Heras-Domingo, J.; Graux, R.; Hai, X.; Lu, J.; Garcia-Gasulla, D.; López, N.; Pérez-Ramírez, J.; Mitchell, S. Quantitative Description of Metal Center Organization and Interactions in Single-Atom Catalysts. *Adv. Mater.* **2024**, *36* (5), 2307991. doi.org/10.1002/adma.202307991
(21) Xu, Z.; Xu, Y.; Lu, H.; Yang, T.; Lin, X.; Shao, L.; Ren, F. Efficient and C2-Selective Arylation of Indoles, Benzofurans, and Benzothiophenes with Iodobenzenes in Water at Room Temperature. *Tetrahedron* **2015**, *71* (18), 2616–2621. doi.org/10.1016/j.tet.2015.03.051
(22) Song, H.; Yang, Z.; Tung, C.-H.; Wang, W. Iron-Catalyzed Reductive Coupling of Nitroarenes with Olefins: Intermediate of Iron–Nitroso Complex. *ACS Catal.* **2020**, *10* (1), 276–281. doi.org/10.1021/acscatal.9b03604
(23) Okamoto, K.; Shimbayashi, T.; Yoshida, M.; Nanya, A.; Ohe, K. Synthesis of 2H-Azirines by Iridium-Catalyzed Decarboxylative Ring Contraction of Isoxazol-5(4H)-Ones. *Angew. Chem. Int. Ed.* **2016**, *55* (25), 7199–7202. doi.org/10.1002/anie.201602241
(24) Perea-Buceta, J. E.; Wirtanen, T.; Laukkanen, O.-V.; Mäkelä, M. K.; Nieger, M.; Melchionna, M.; Huittinen, N.; Lopez-Sanchez, J. A.; Helaja, J. Cycloisomerization of 2-Alkynylanilines to Indoles Catalyzed by Carbon-Supported Gold Nanoparticles and Subsequent Homocoupling to 3,3′-Biindoles. *Angew. Chem. Int. Ed.* **2013**, *52* (45), 11835–11839. doi.org/10.1002/anie.201305579
(25) Malmgren, J.; Nagendiran, A.; Tai, C.-W.; Bäckvall, J.-E.; Olofsson, B. C-2 Selective Arylation of Indoles with Heterogeneous Nanopalladium and Diaryliodonium Salts. *Chem.-Eur. J.* **2014**, *20* (42), 13531–13535. doi.org/10.1002/chem.201404017
(26) Wang, X.; Lane, B. S.; Sames, D. Direct C-Arylation of Free (NH)-Indoles and Pyrroles Catalyzed by Ar−Rh(III) Complexes Assembled In Situ. *J. Am. Chem. Soc.* **2005**, *127* (14), 4996–4997. doi.org/10.1021/ja050279p
(27) Lu, M.-Z.; Lu, P.; Xu, Y.-H.; Loh, T.-P. Mild Rh(III)-Catalyzed Direct C–H Bond Arylation of (Hetero)Arenes with Arylsilanes in Aqueous Media. *Org. Lett.* **2014**, *16* (10), 2614–2617. doi.org/10.1021/ol500754h
(28) Ruan, L.; Shi, M.; Mao, S.; Yu, L.; Yang, F.; Tang, J. An Efficient Approach to Construct 2-Arylbenzo[*b*]Furans from 2-Methoxychalcone Epoxides. *Tetrahedron* **2014**, *70* (5), 1065–1070. doi.org/10.1016/j.tet.2013.12.050
(29) Shen, M.; Leslie, B. E.; Driver, T. G. Dirhodium(II)-Catalyzed Intramolecular C□H Amination of Aryl Azides. *Angew. Chem. Int. Ed.* **2008**, *47* (27), 5056–5059. doi.org/10.1002/anie.200800689

(30) Bzeih, T.; Zhang, K.; Khalaf, A.; Hachem, A.; Alami, M.; Hamze, A. One-Pot Reaction between N-Tosylhydrazones and 2-Nitrobenzyl Bromide: Route to NH-Free C2-Arylindoles. *J. Org. Chem.* **2019**, *84* (1), 228–238. doi.org/10.1021/acs.joc.8b02623

(31) Giorgi, E.; Binacchi, F.; Mannelli, M.; Cirri, D.; Cesari, A.; Boldrini, M.; Marotta, C.; Ghelarducci, C.; Corvaglia, D.; Bellina, F.; Gamberi, T.; Pratesi, A.; Gabbiani, C. *Trans*-Pd(II) Complexes: A Different Approach for the Development of Promising Anticancer Drugs. *Inorganica Chim. Acta* **2025**, *581*, 122645. doi.org/10.1016/j.ica.2025.122645

(32) Rajput, J.; Moss, J. R.; Hutton, A. T.; Hendricks, D. T.; Arendse, C. E.; Imrie, C. Synthesis, Characterization and Cytotoxicity of Some Palladium(II), Platinum(II), Rhodium(I) and Iridium(I) Complexes of Ferrocenylpyridine and Related Ligands. Crystal and Molecular Structure of *Trans*-Dichlorobis(3-Ferrocenylpyridine)Palladium(II). *J. Organomet. Chem.* **2004**, *689* (9), 1553–1568. doi.org/10.1016/j.jorganchem.2004.01.034

(33) Amadio, E.; Cavinato, G.; Dolmella, A.; Toniolo, L. Catalytic Properties of [Pd(COOMe)nX2−n(PPh3)2] (n = 0, 1, 2; X = Cl, NO2, ONO2, OAc and OTs) in the Oxidative Carbonylation of MeOH. *Inorg. Chem.* **2010**, *49* (8), 3721–3729. doi.org/10.1021/ic901569w

(34) Cody, W. L.; Holsworth, D. D.; Powell, N. A.; Jalaie, M.; Zhang, E.; Wang, W.; Samas, B.; Bryant, J.; Ostroski, R.; Ryan, M. J.; Edmunds, J. J. The Discovery and Preparation of Disubstituted Novel Amino-Aryl-Piperidine-Based Renin Inhibitors. *Bioorg. Med. Chem.* **2005**, *13* (1), 59–68. doi.org/10.1016/j.bmc.2004.09.056

(35) Kavanagh, M. E.; Gray, J. L.; Gilbert, S. H.; Coyne, A. G.; McLean, K. J.; Davis, H. J.; Munro, A. W.; Abell, C. Substrate Fragmentation for the Design of M. Tuberculosis CYP121 Inhibitors. *ChemMedChem* **2016**, *11* (17), 1924–1935. doi.org/10.1002/cmdc.201600248

(36) Jayaraman, A.; Misal Castro, L. C.; Fontaine, F.-G. Practical and Scalable Synthesis of Borylated Heterocycles Using Bench-Stable Precursors of Metal-Free Lewis Pair Catalysts. *Org. Process Res. Dev.* **2018**, *22* (11), 1489–1499. doi.org/10.1021/acs.oprd.8b00248

(37) Filimonov, V. D.; Krasnokutskaya, E. A.; Kassanova, A. Zh.; Fedorova, V. A.; Stankevich, K. S.; Naumov, N. G.; Bondarev, A. A.; Kataeva, V. A. Synthesis, Structure, and Synthetic Potential of Arenediazonium Trifluoromethanesulfonates as Stable and Safe Diazonium Salts. *Eur. J. Org. Chem.* **2019**, *2019* (4), 665–674. doi.org/10.1002/ejoc.201800887

(38) Cresswell, A. J.; Lloyd-Jones, G. C. Room-Temperature Gold-Catalysed Arylation of Heteroarenes: Complementarity to Palladium Catalysis. *Chem.-Eur. J.* **2016**, *22* (36), 12641–12645. doi.org/10.1002/chem.201602893

(39) Chen, D.-W.; Ochiai, M. Chromium(II)-Mediated Reactions of Iodonium Tetrafluoroborates with Aldehydes: Umpolung of Reactivity of Diaryl-, Alkenyl(Aryl)-, and Alkynyl(Aryl)Iodonium Tetrafluoroborates. *J. Org. Chem.* **1999**, *64* (18), 6804–6814. doi.org/10.1021/jo990809y

(40) Bielawski, M.; Aili, D.; Olofsson, B. Regiospecific One-Pot Synthesis of Diaryliodonium Tetrafluoroborates from Arylboronic Acids and Aryl Iodides. *J. Org. Chem.* **2008**, *73* (12), 4602–4607. doi.org/10.1021/jo8004974

(41) Beaud, R.; Phipps, R. J.; Gaunt, M. J. Enantioselective Cu-Catalyzed Arylation of Secondary Phosphine Oxides with Diaryliodonium Salts toward the Synthesis of P-Chiral Phosphines. *J. Am. Chem. Soc.* **2016**, *138* (40), 13183–13186. doi.org/10.1021/jacs.6b09334

(42) Gao, W.-Y.; Yu, G.-Y.; Li, Y.-Y.; Liang, R.-X.; Hong, X.; Jia, Y.-X. Palladium-Catalyzed Dearomative Heck/Suzuki Coupling of C2-Deuterated or C2-Nonsubstituted Indoles. *Org. Lett.* **2024**, *26* (12), 2359–2364. doi.org/10.1021/acs.orglett.4c00166

Spectra

^{1}H NMR (400 MHz, $CDCl_3$) of **1**

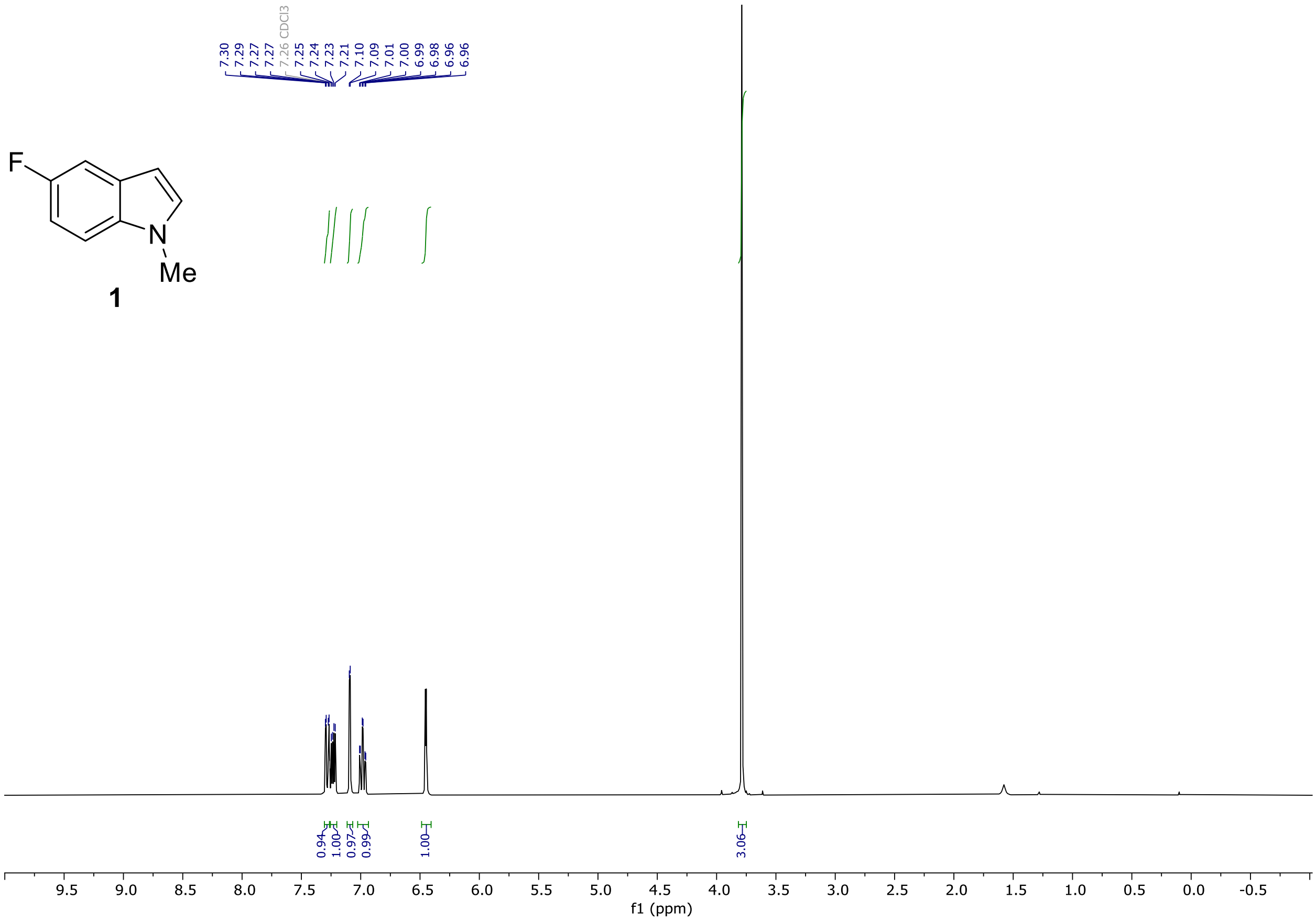

$^{13}C\{^{1}H\}$ NMR (100 MHz, $CDCl_3$) of **1**

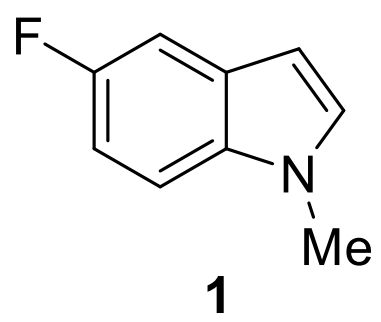

1

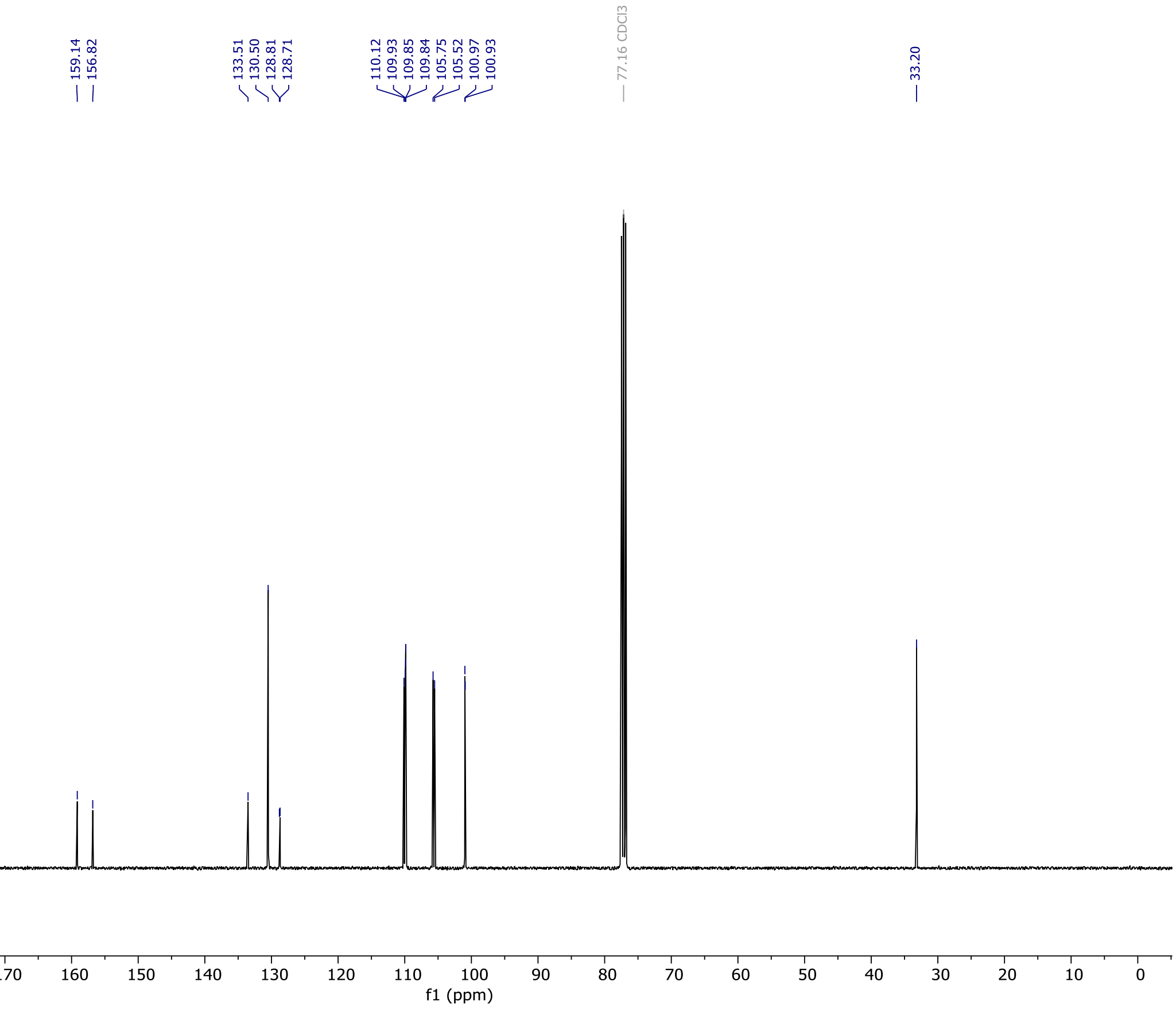

$^{19}F\{^{1}H\}$ NMR (376 MHz, $CDCl_3$) of **1**

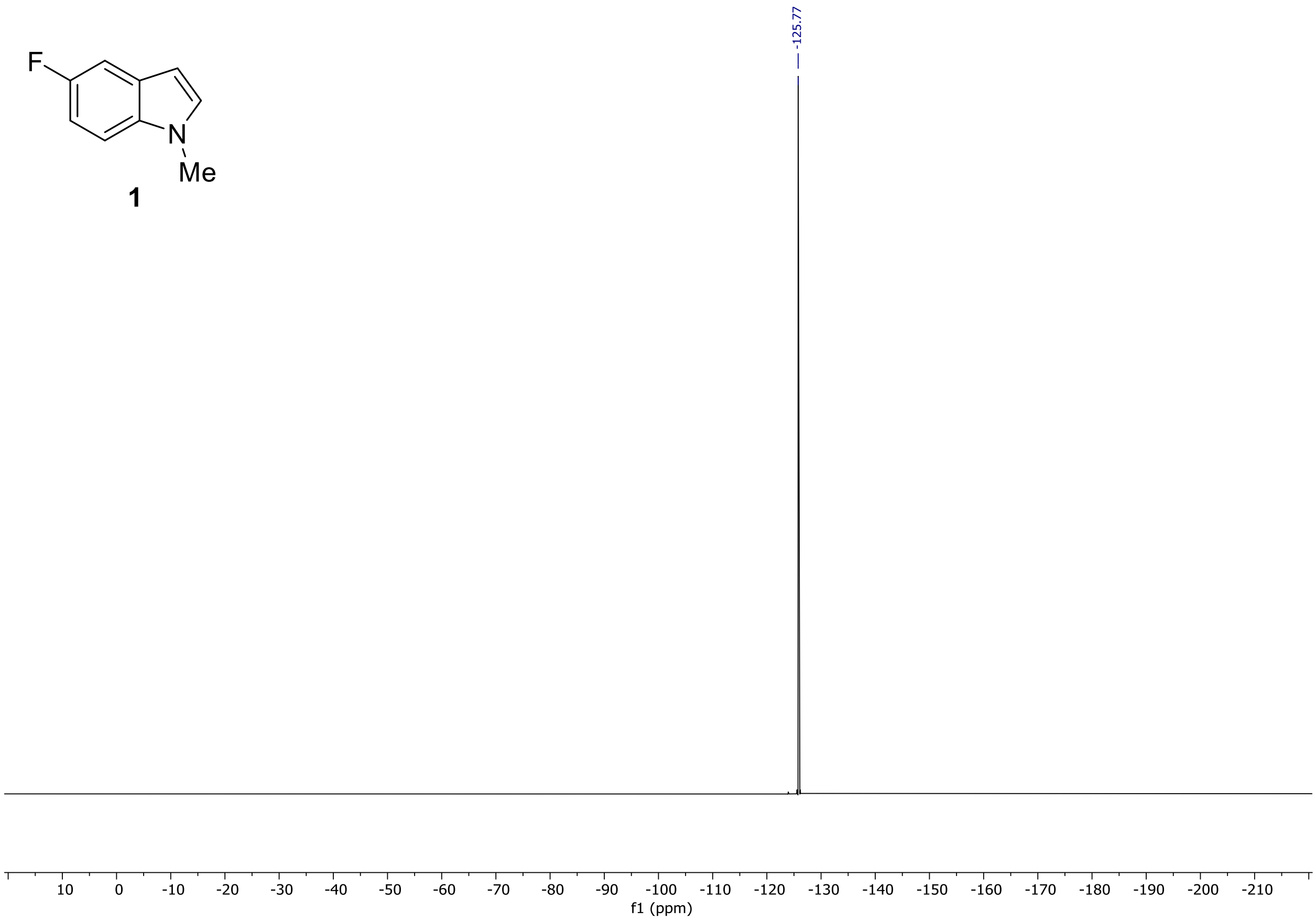

^{1}H NMR (400 MHz, $CDCl_3$) of **2**

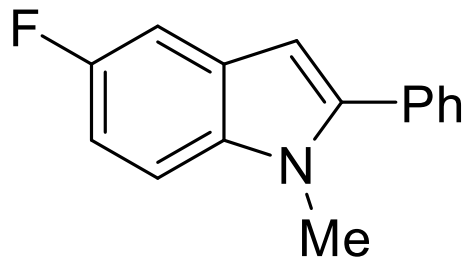

2

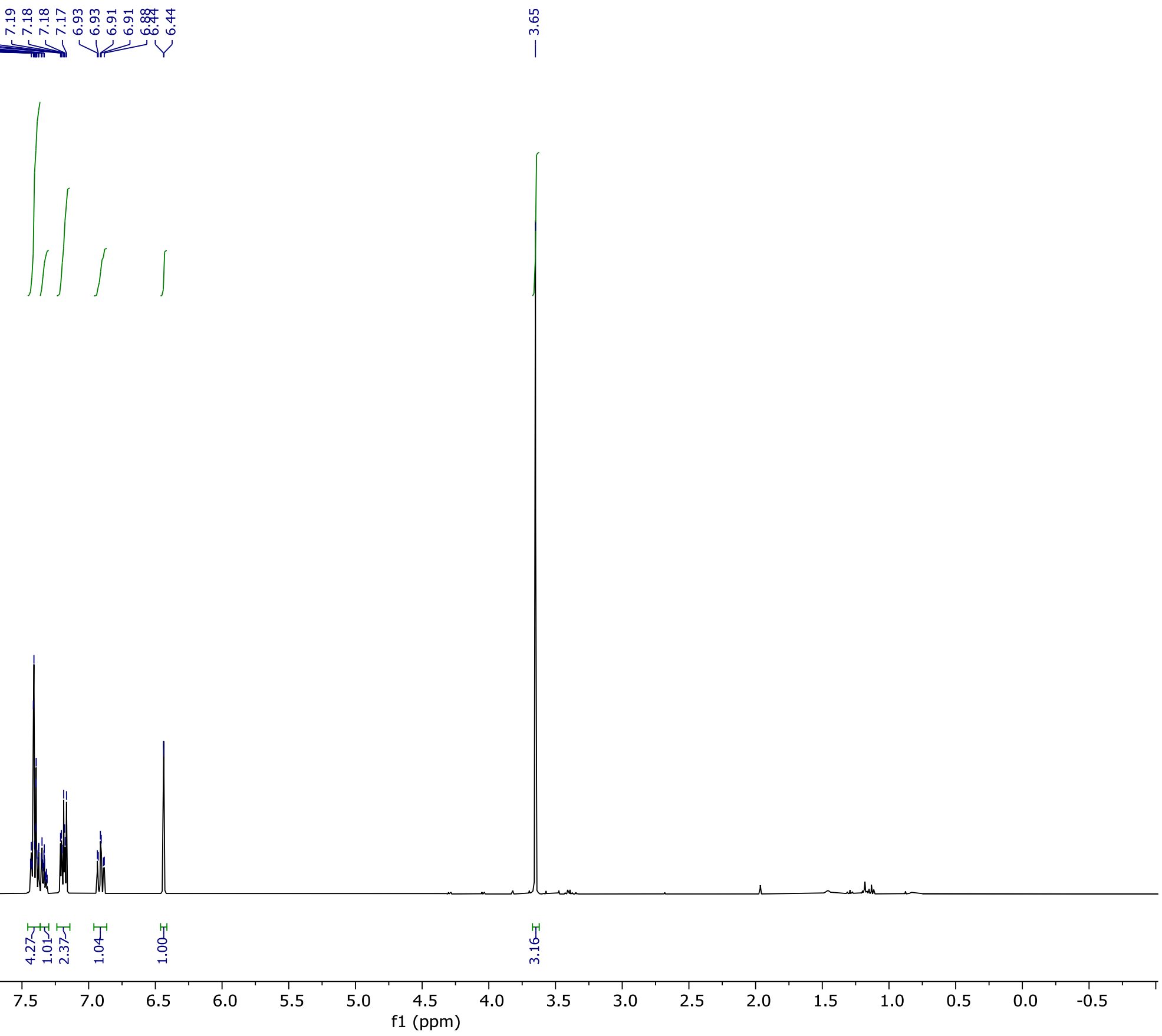

$^{13}C\{^{1}H\}$ NMR (100 MHz, $CDCl_3$) of **2**

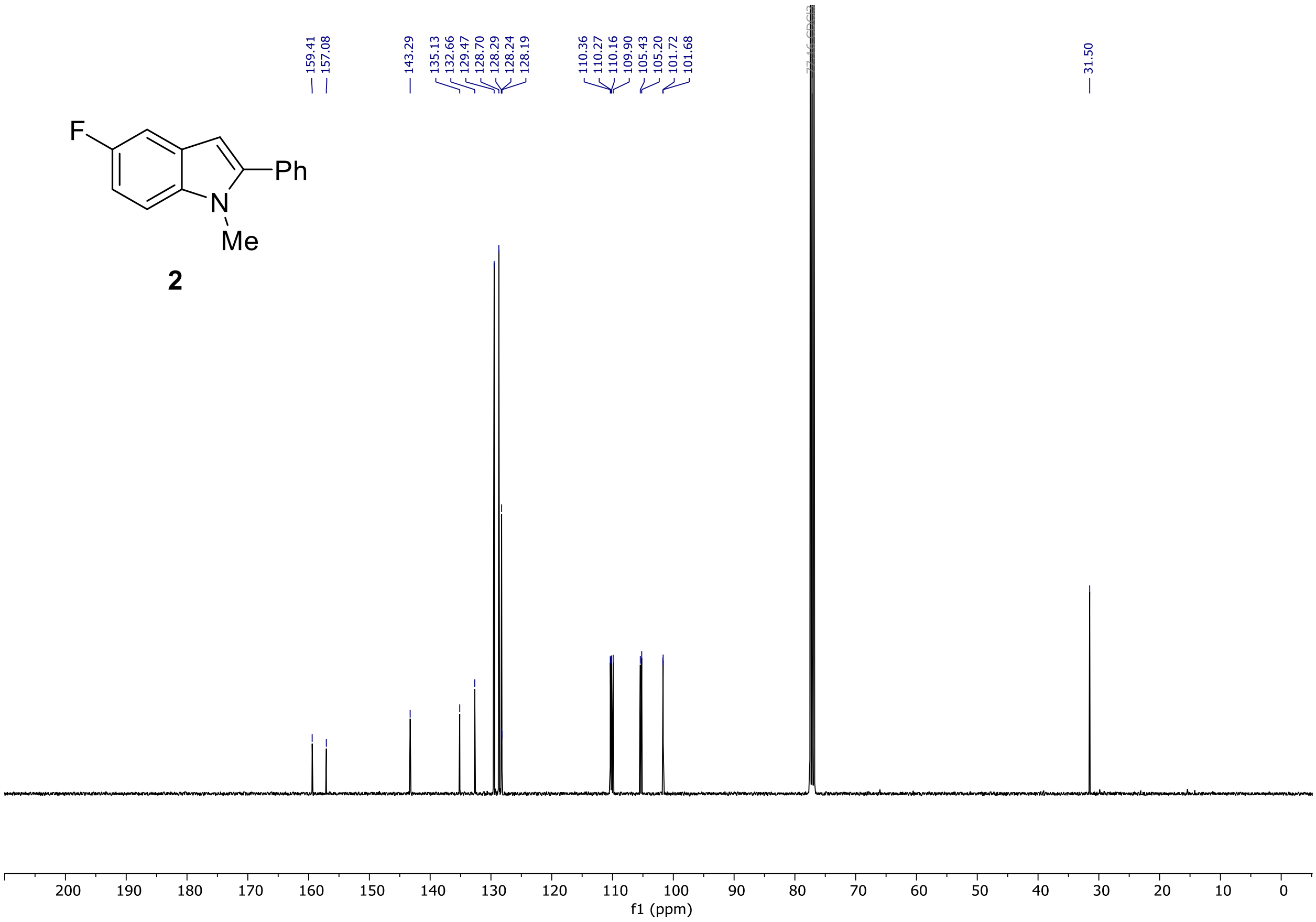

2

$^{19}F\{^{1}H\}$ NMR (376 MHz, $CDCl_3$) of **2**

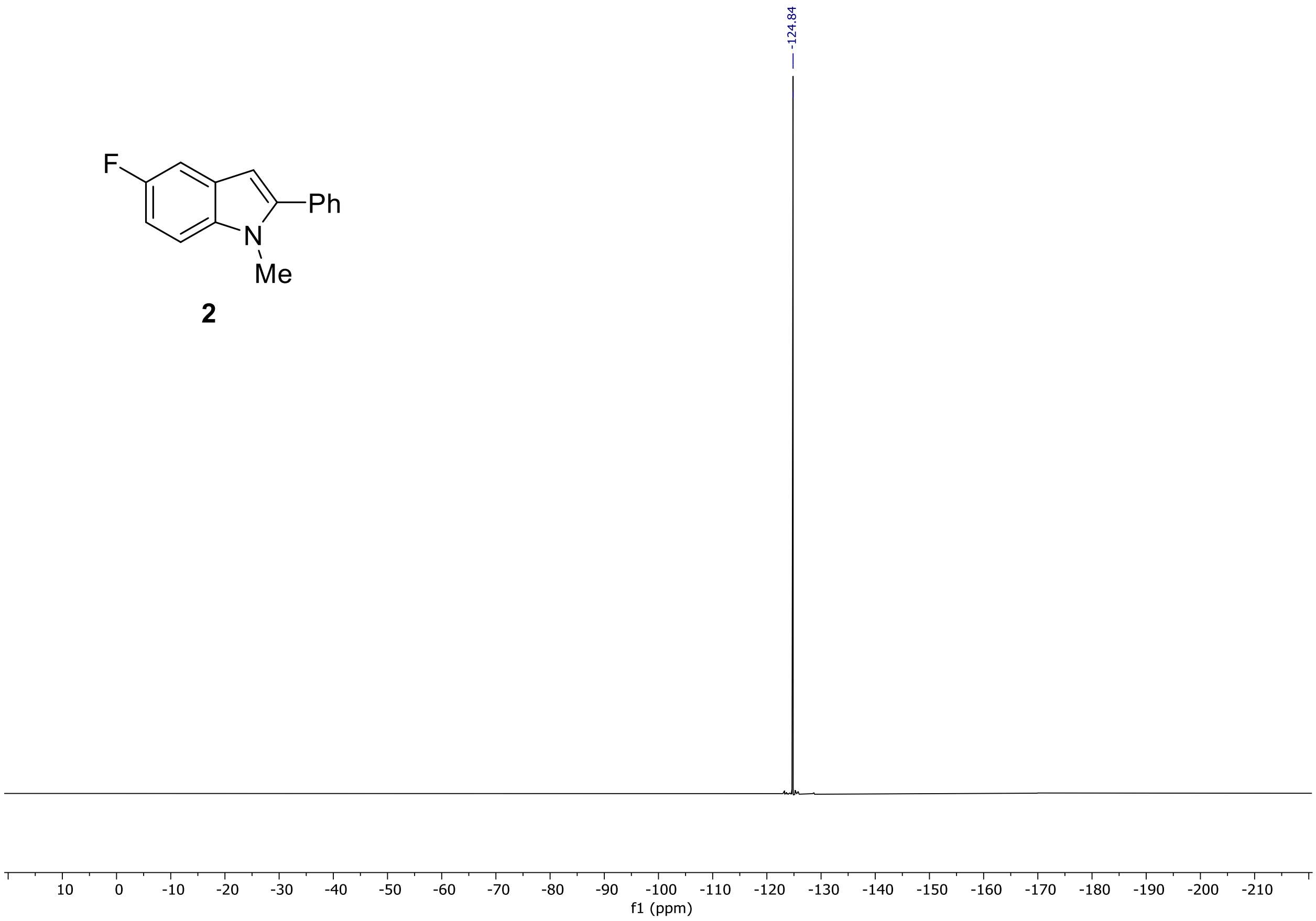

^{1}H NMR (400 MHz, $CDCl_3$) of **3**

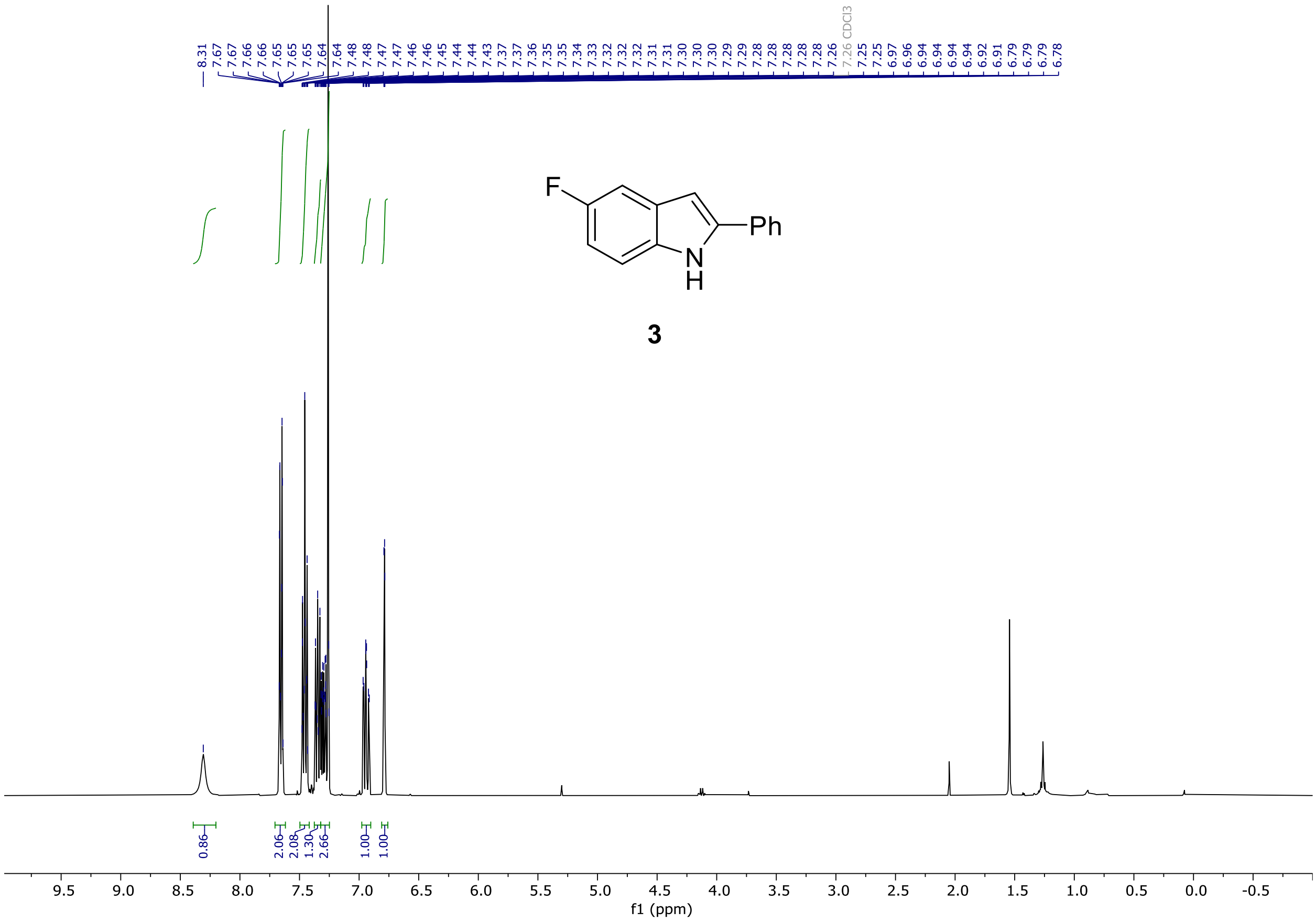

$^{13}C\{^{1}H\}$ NMR (100 MHz, $CDCl_3$) of **3**

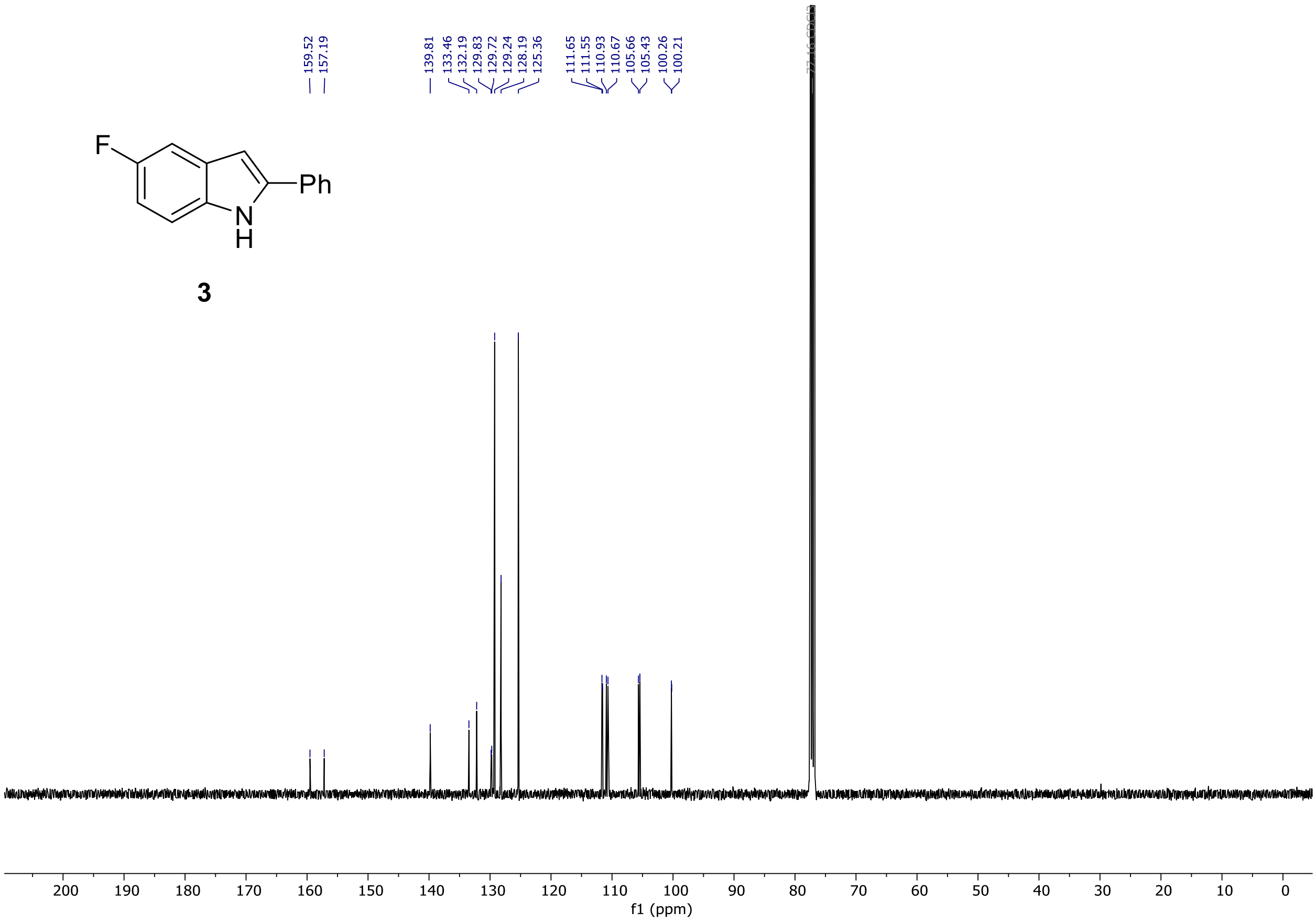

$^{19}F\{^1H\}$ NMR (376 MHz, $CDCl_3$) of **3**

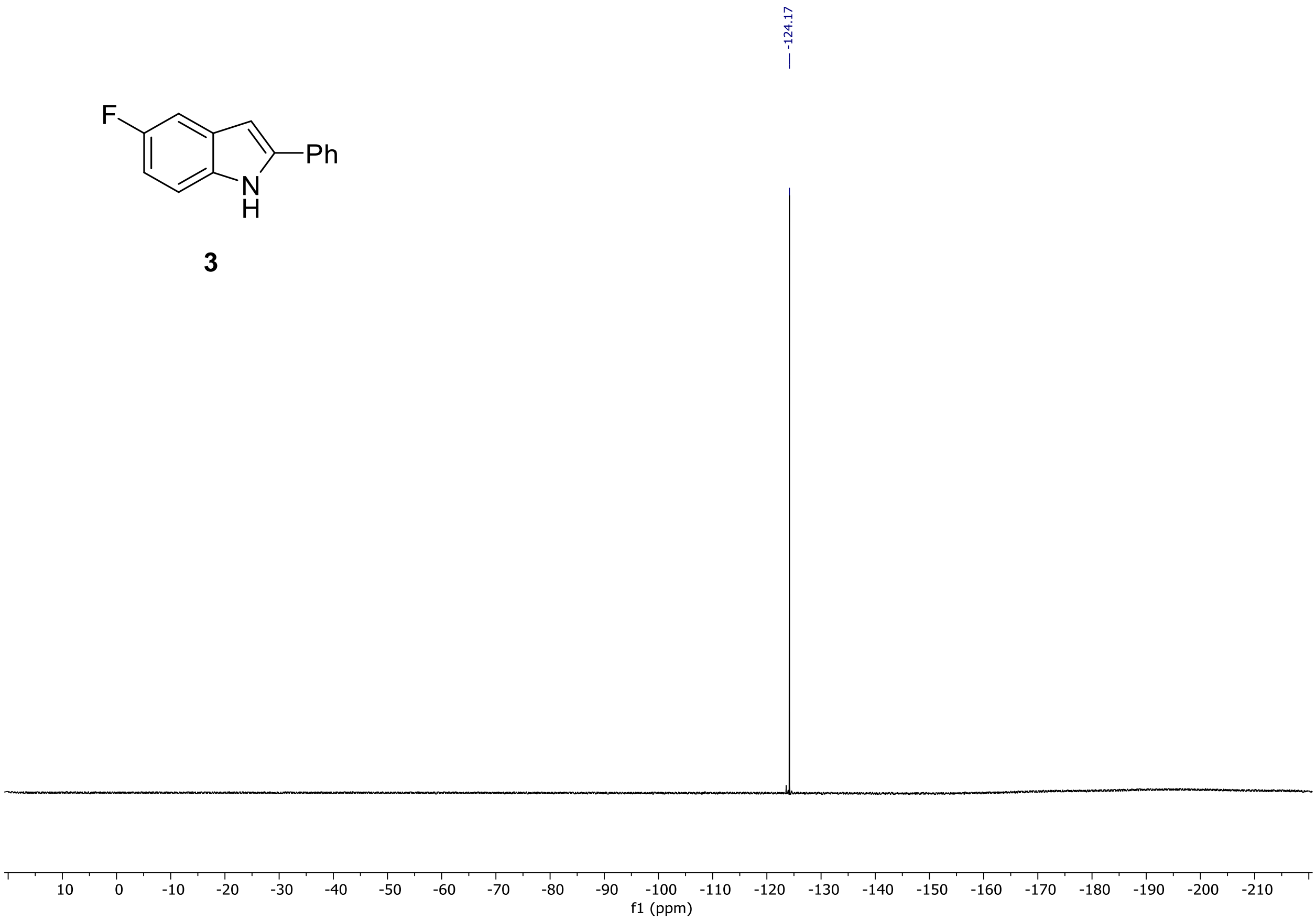

^{1}H NMR (400 MHz, $CDCl_3$) of **6**

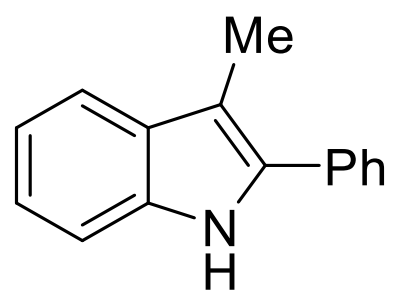

6

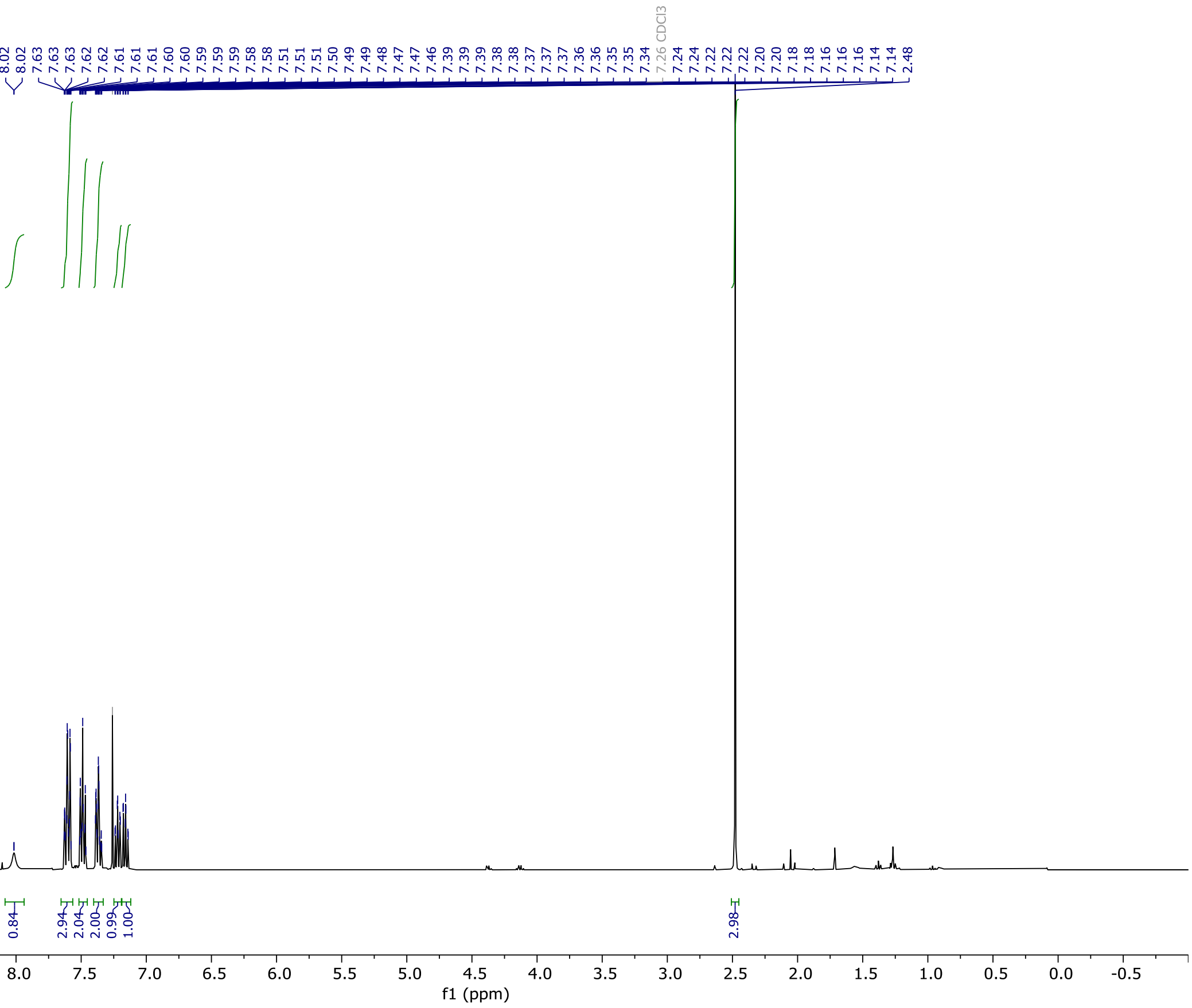

$^{13}C\{^{1}H\}$ NMR (100 MHz, $CDCl_3$) of **6**

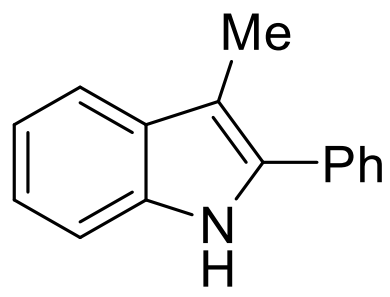

6

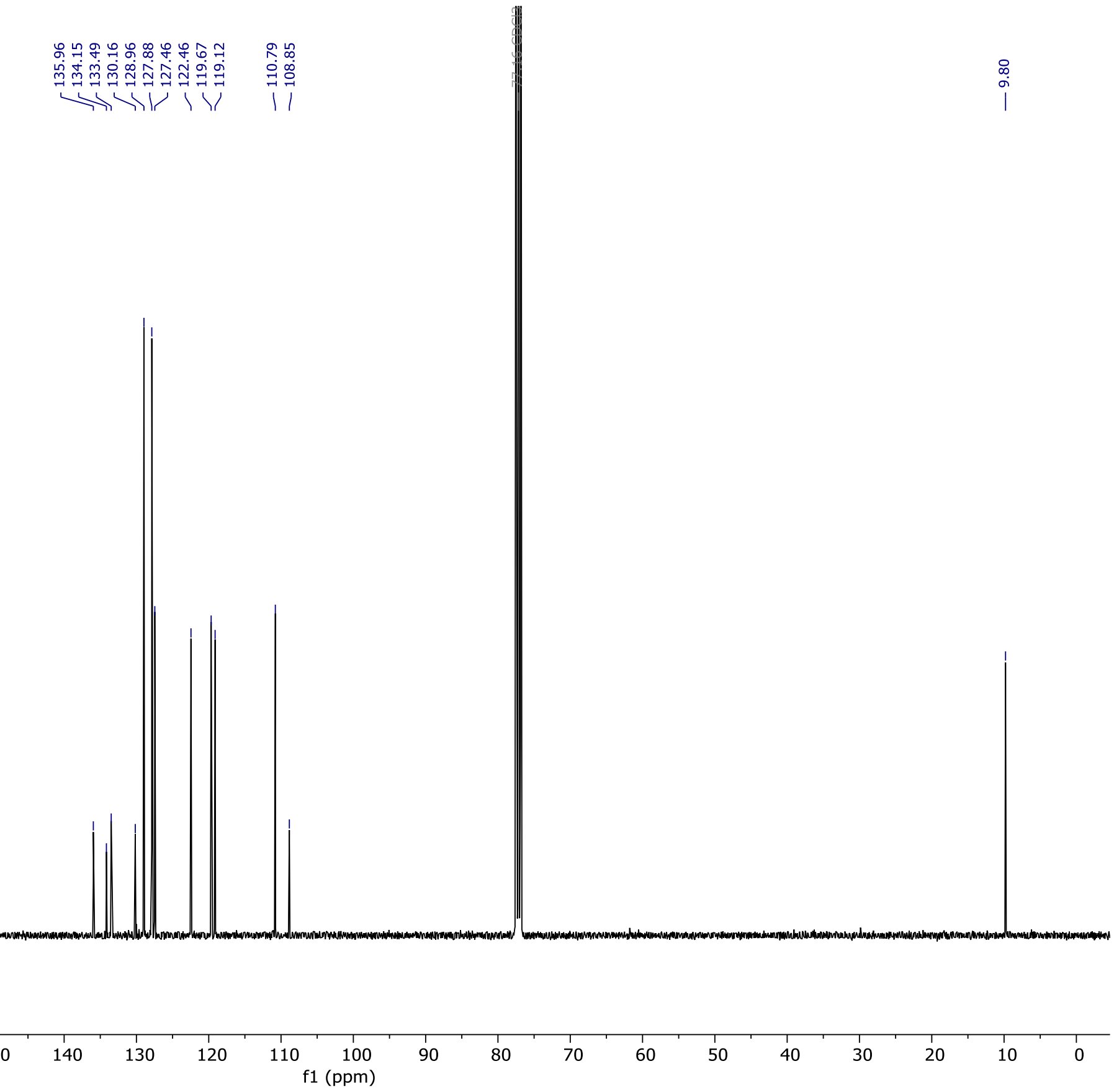

^{1}H NMR (400 MHz, $CDCl_3$) of **7**

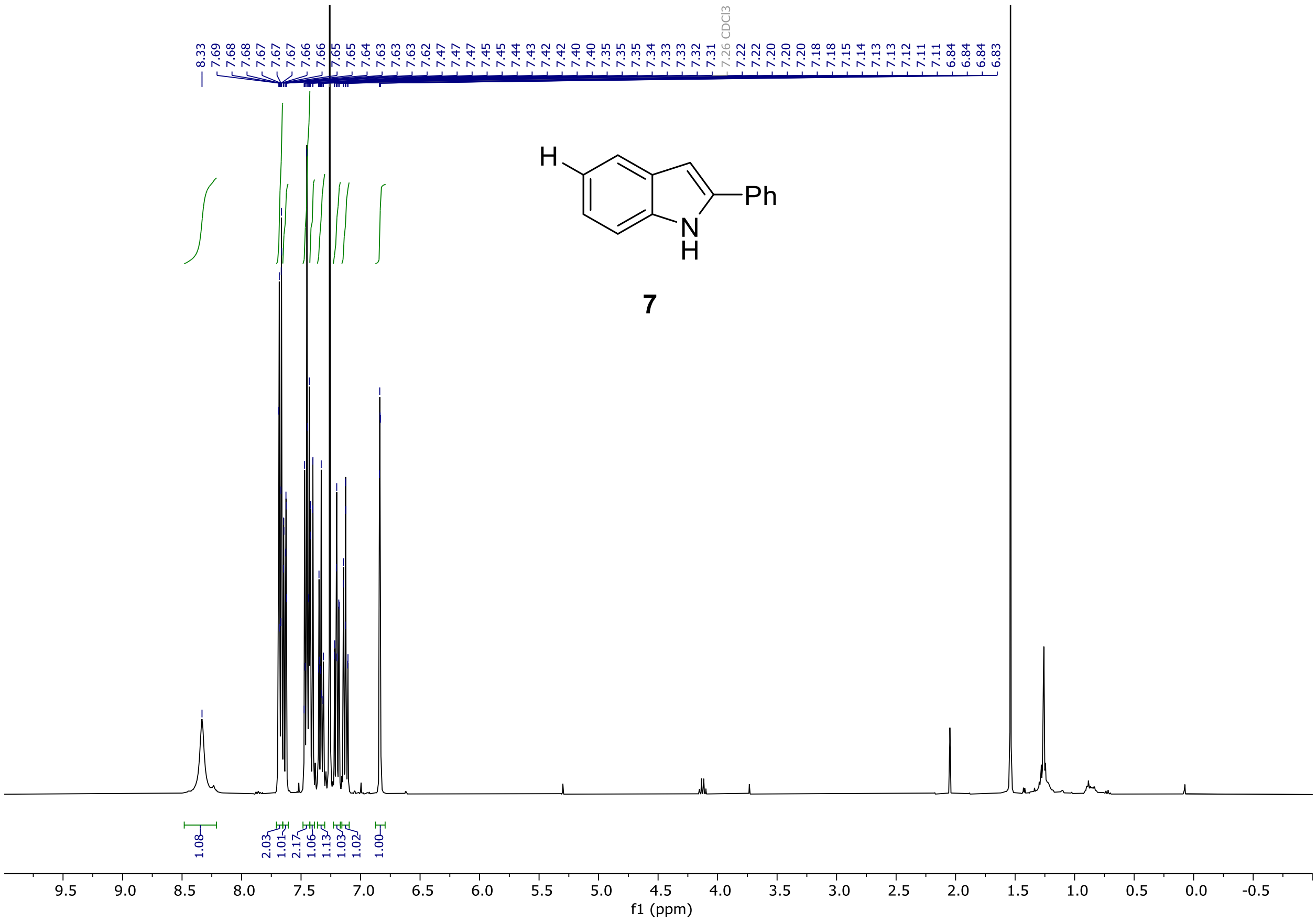

$^{13}C\{^{1}H\}$ NMR (100 MHz, $CDCl_3$) of **7**

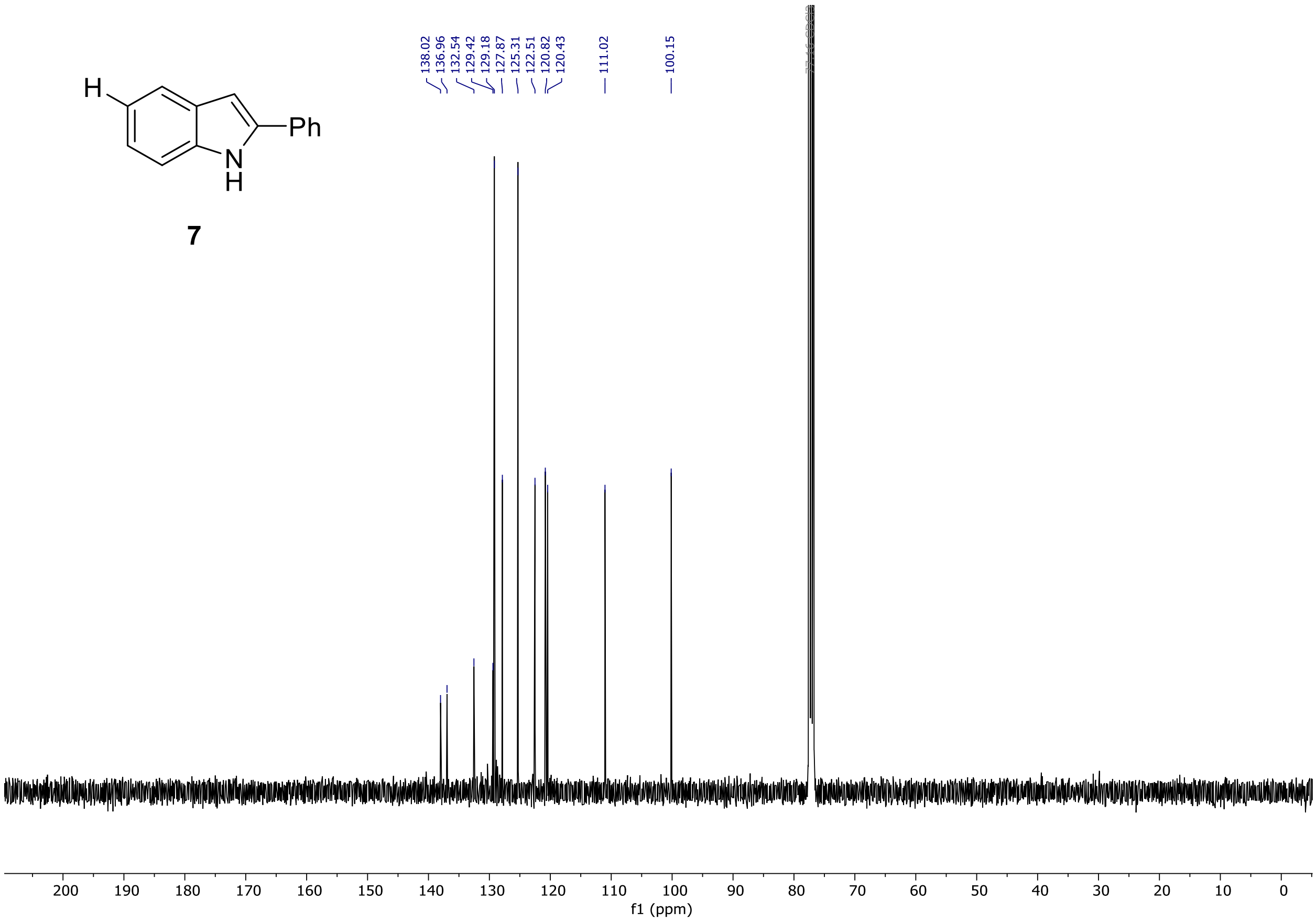

^{1}H NMR (400 MHz, $CDCl_3$) of **9**

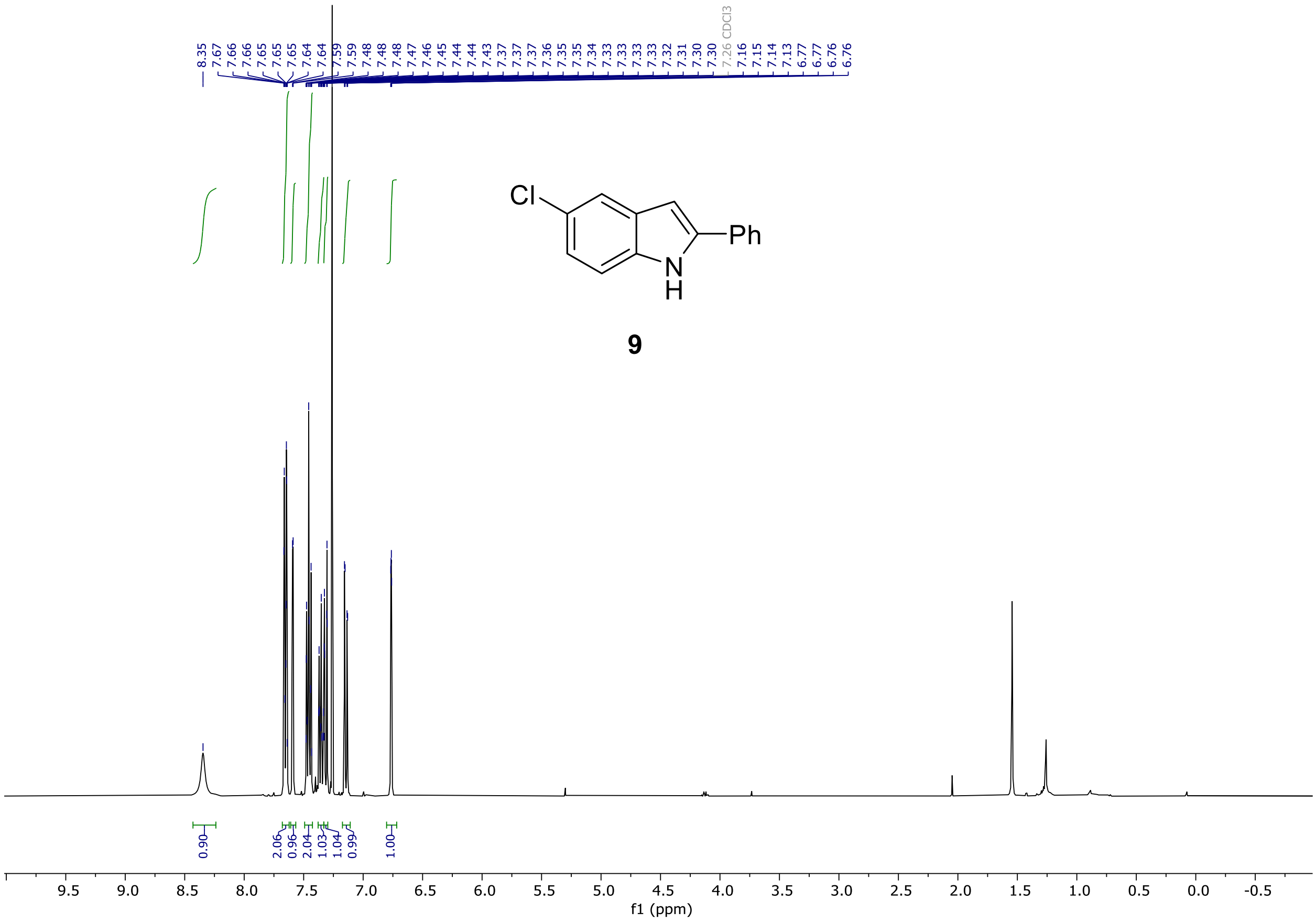

$^{13}C\{^{1}H\}$ NMR (100 MHz, $CDCl_3$) of **9**

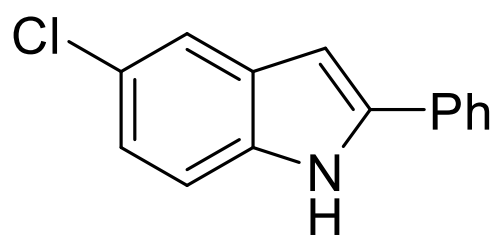

9

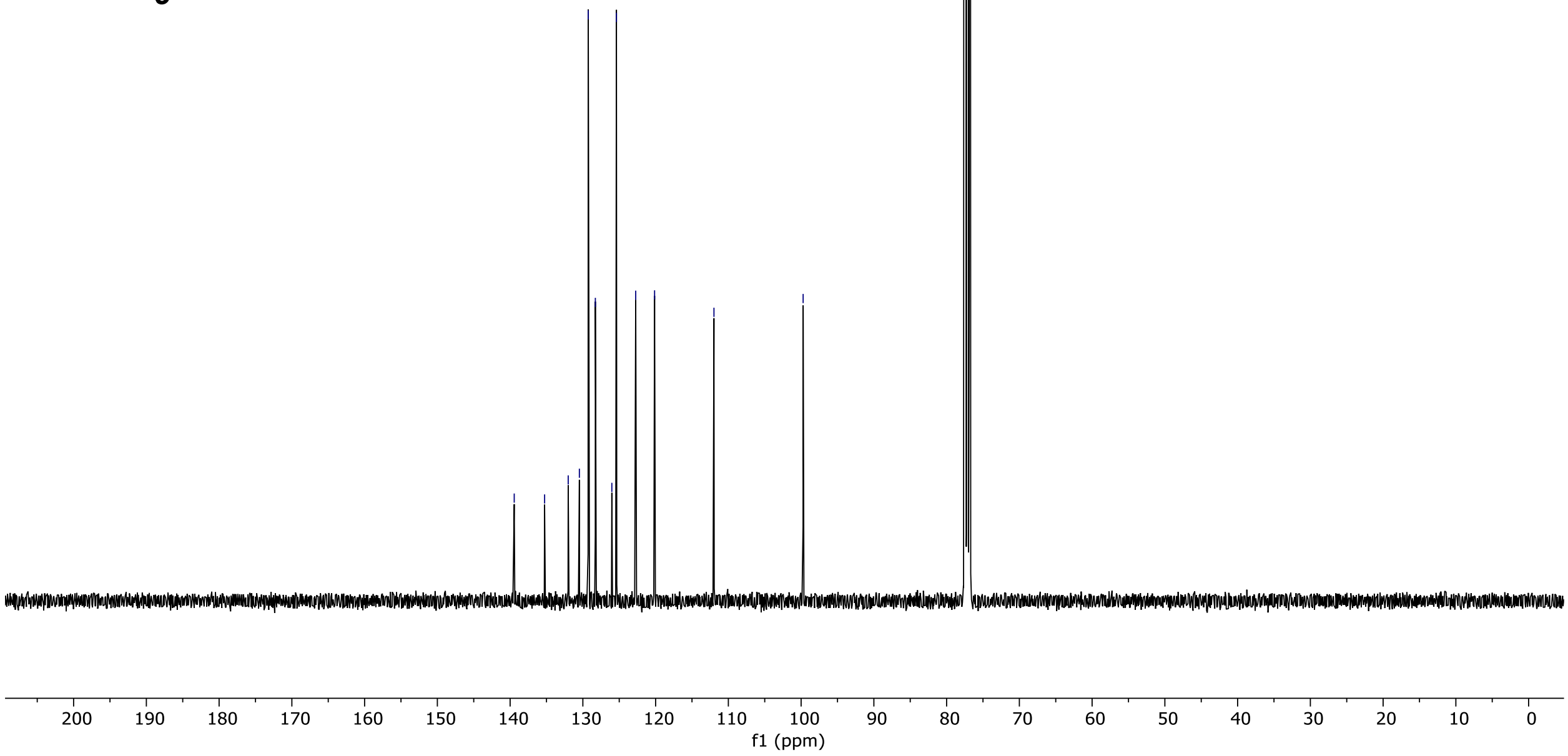

^1H NMR (400 MHz, $CDCl_3$) of **10**

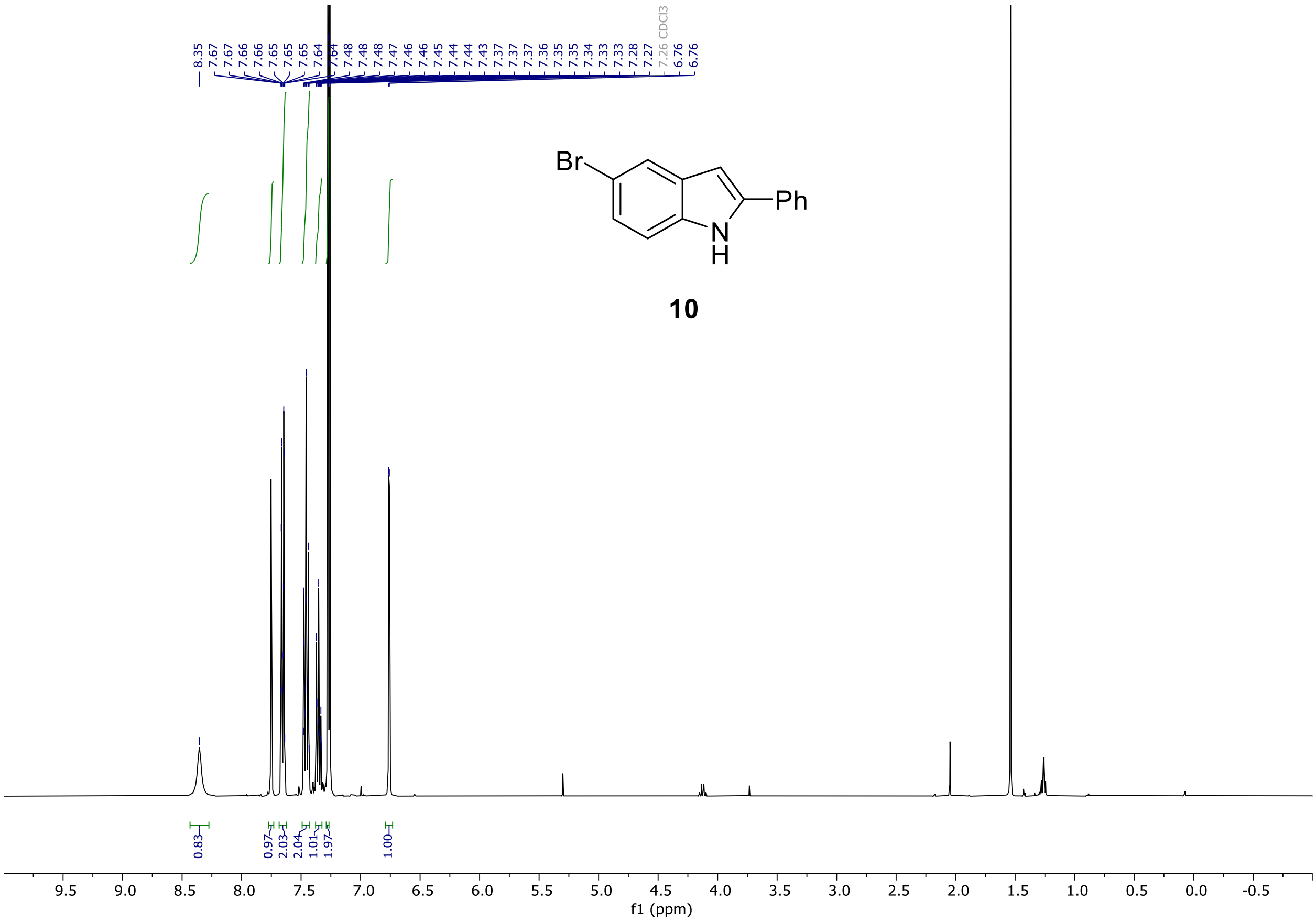

$^{13}C\{^{1}H\}$ NMR (100 MHz, $CDCl_3$) of **10**

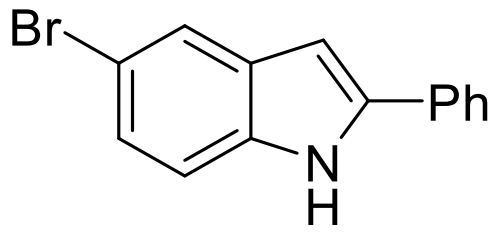

10

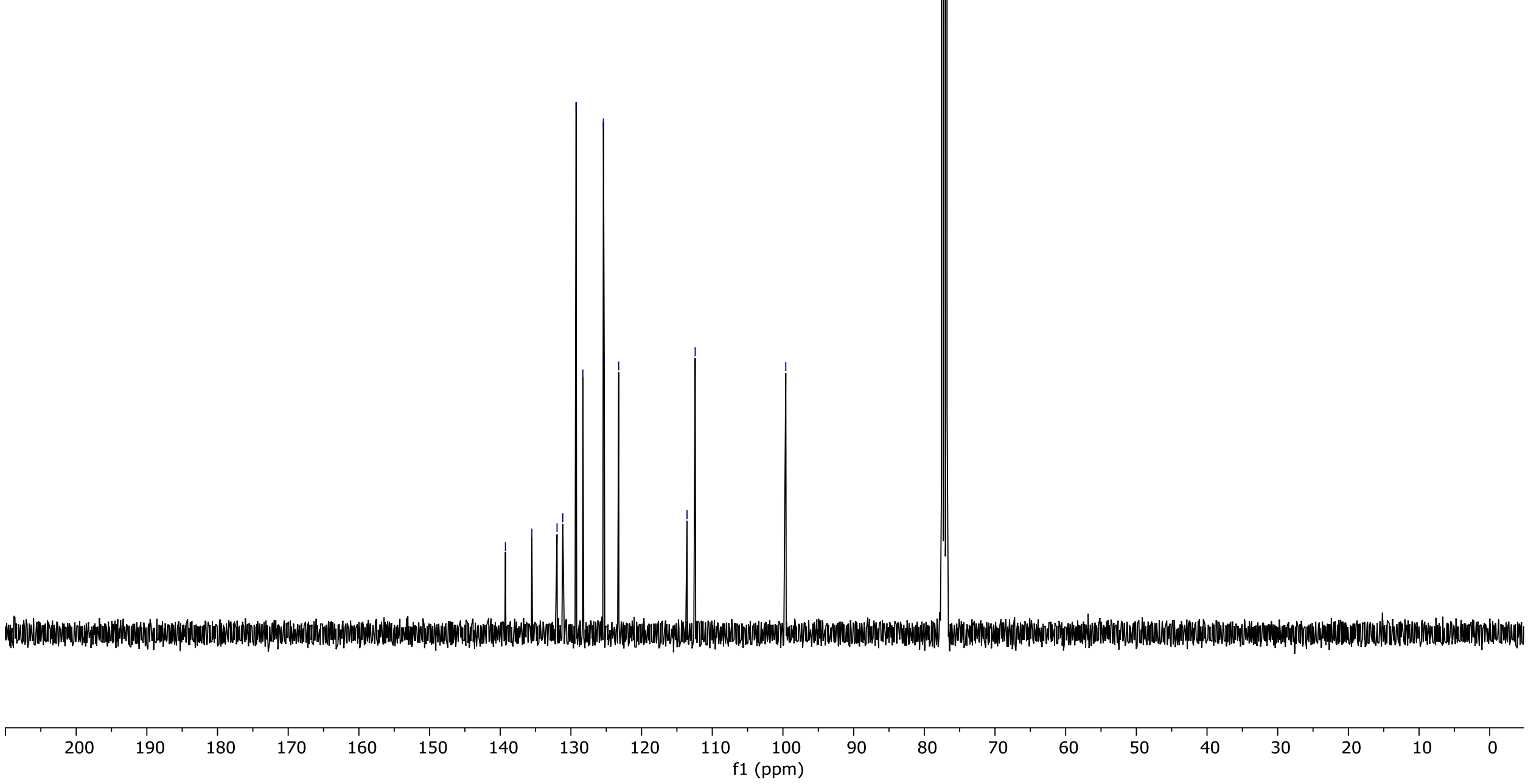

^{1}H NMR (400 MHz, $CDCl_3$) of **11**

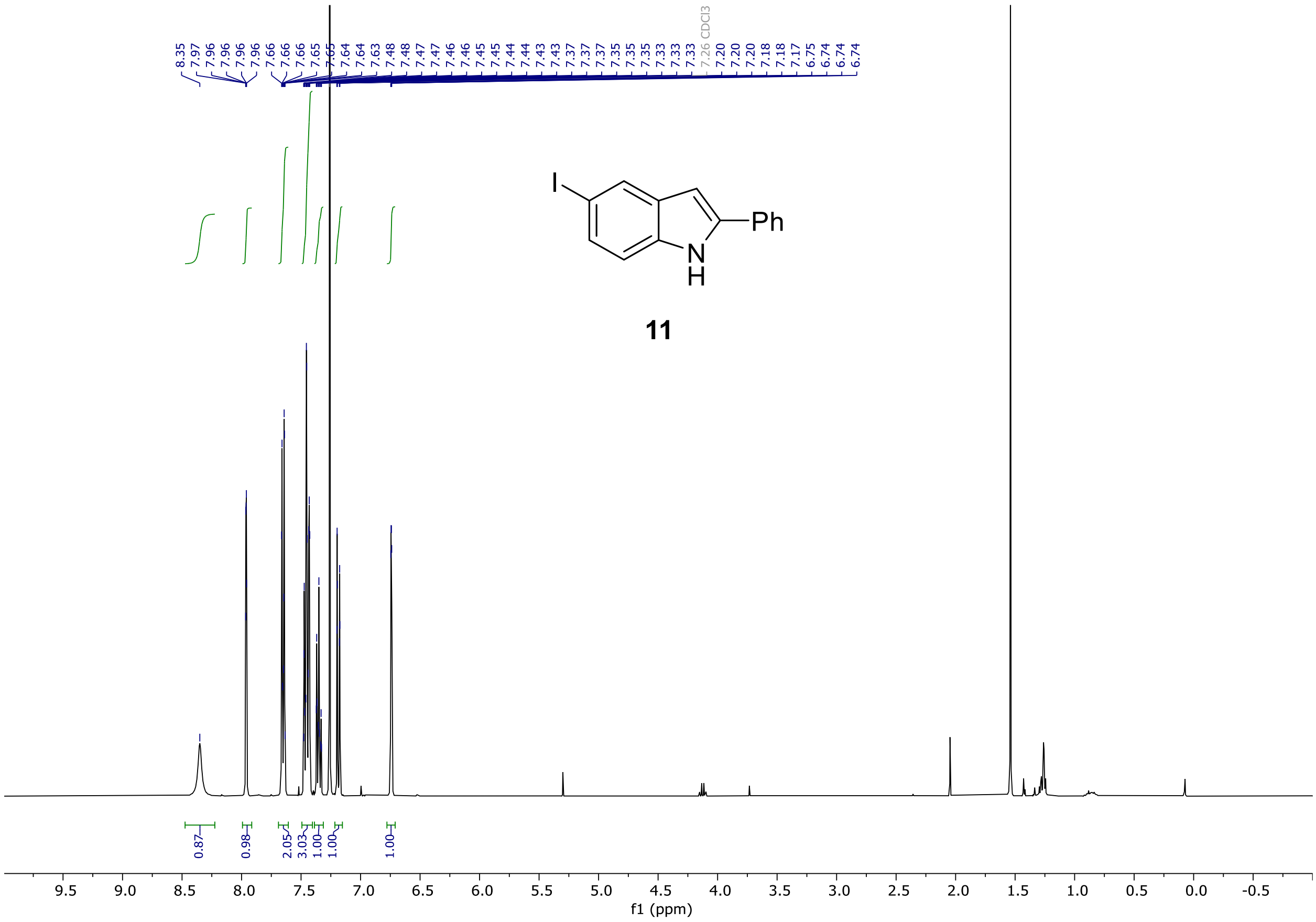

$^{13}C\{^{1}H\}$ NMR (100 MHz, $CDCl_3$) of **11**

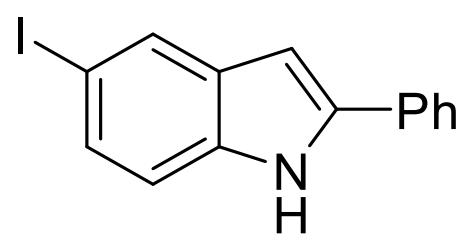

11

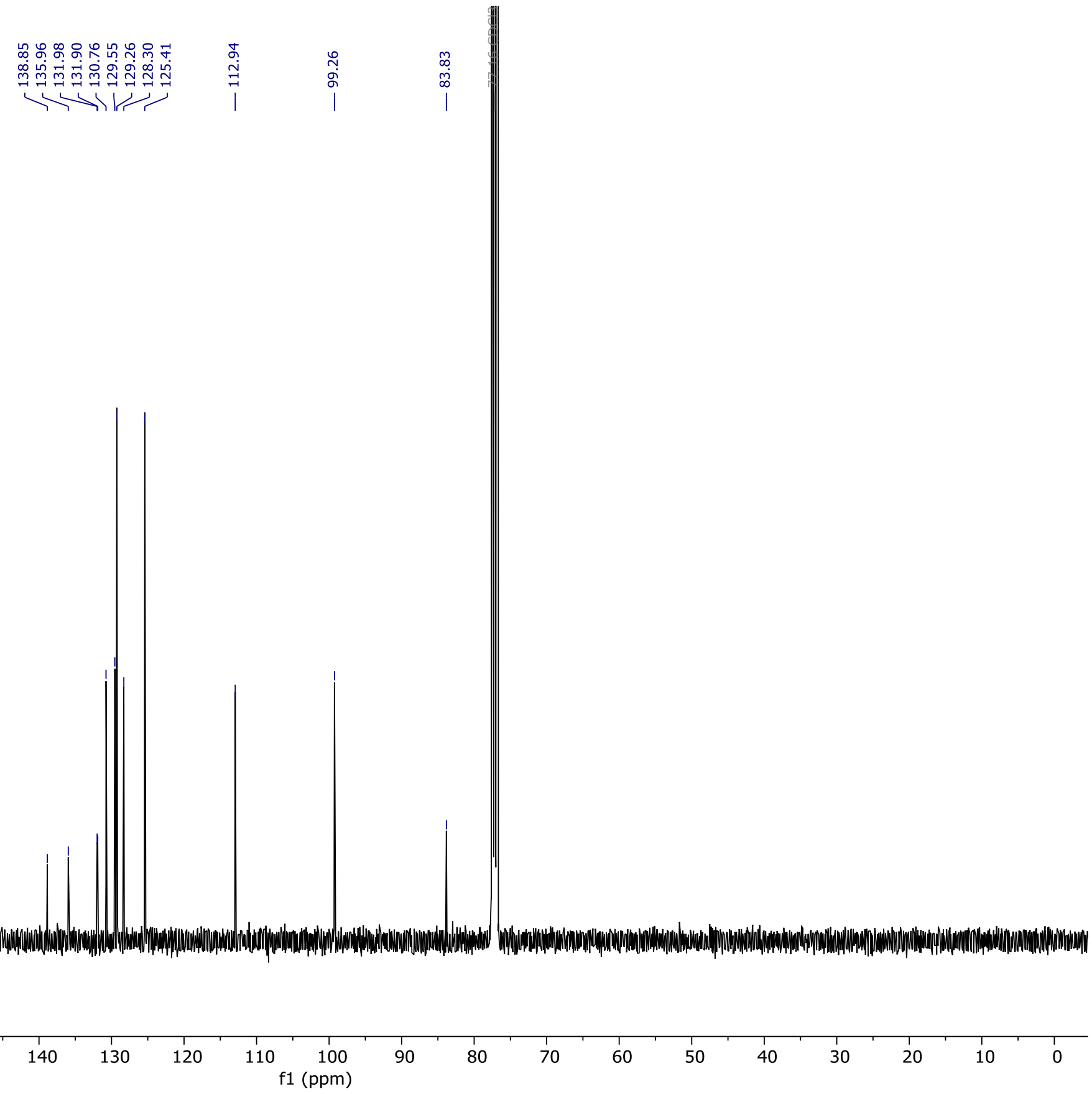

^{1}H NMR (400 MHz, $CDCl_3$) of **12**

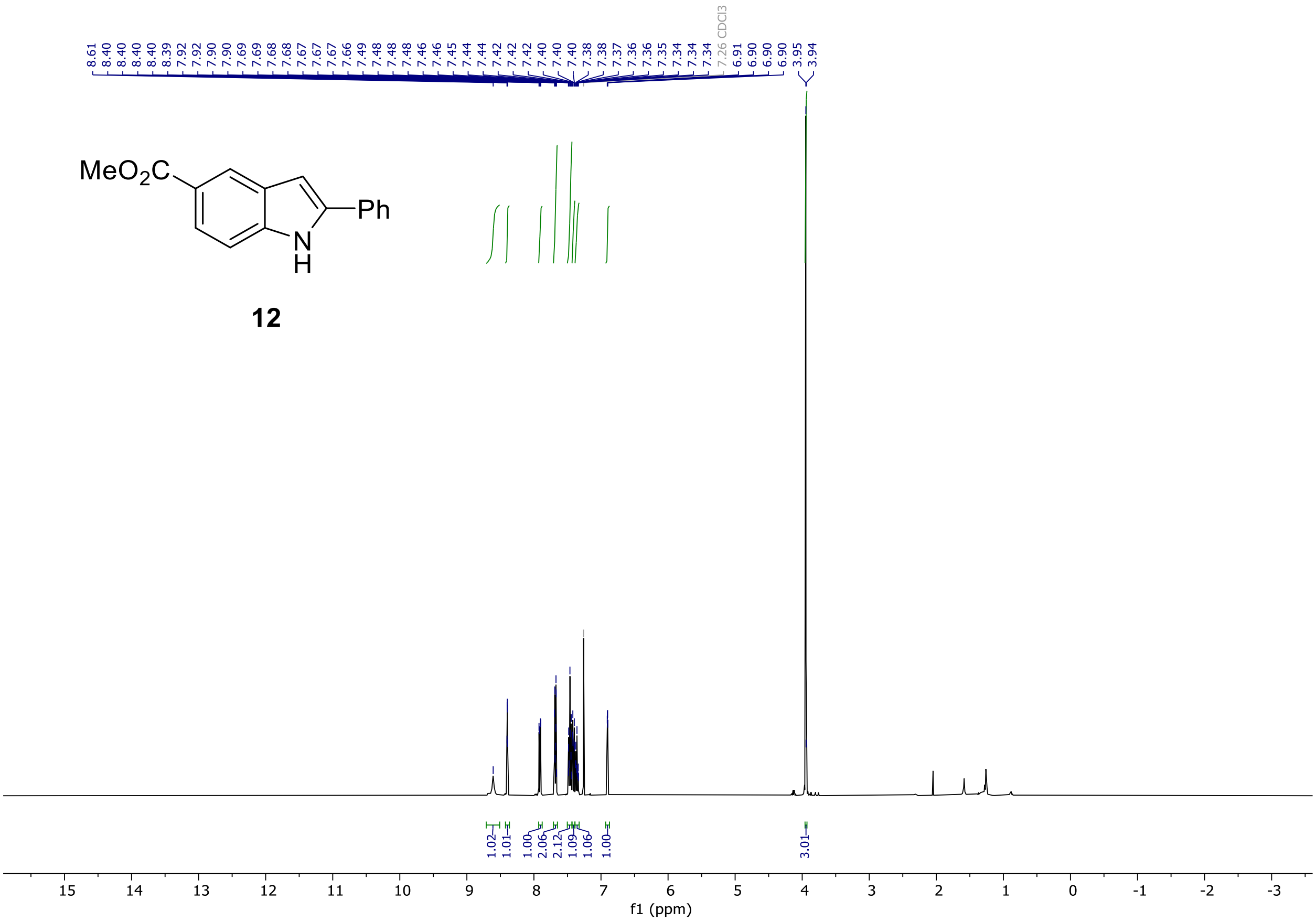

12

$^{13}C\{^{1}H\}$ NMR (100 MHz, $CDCl_3$) of **12**

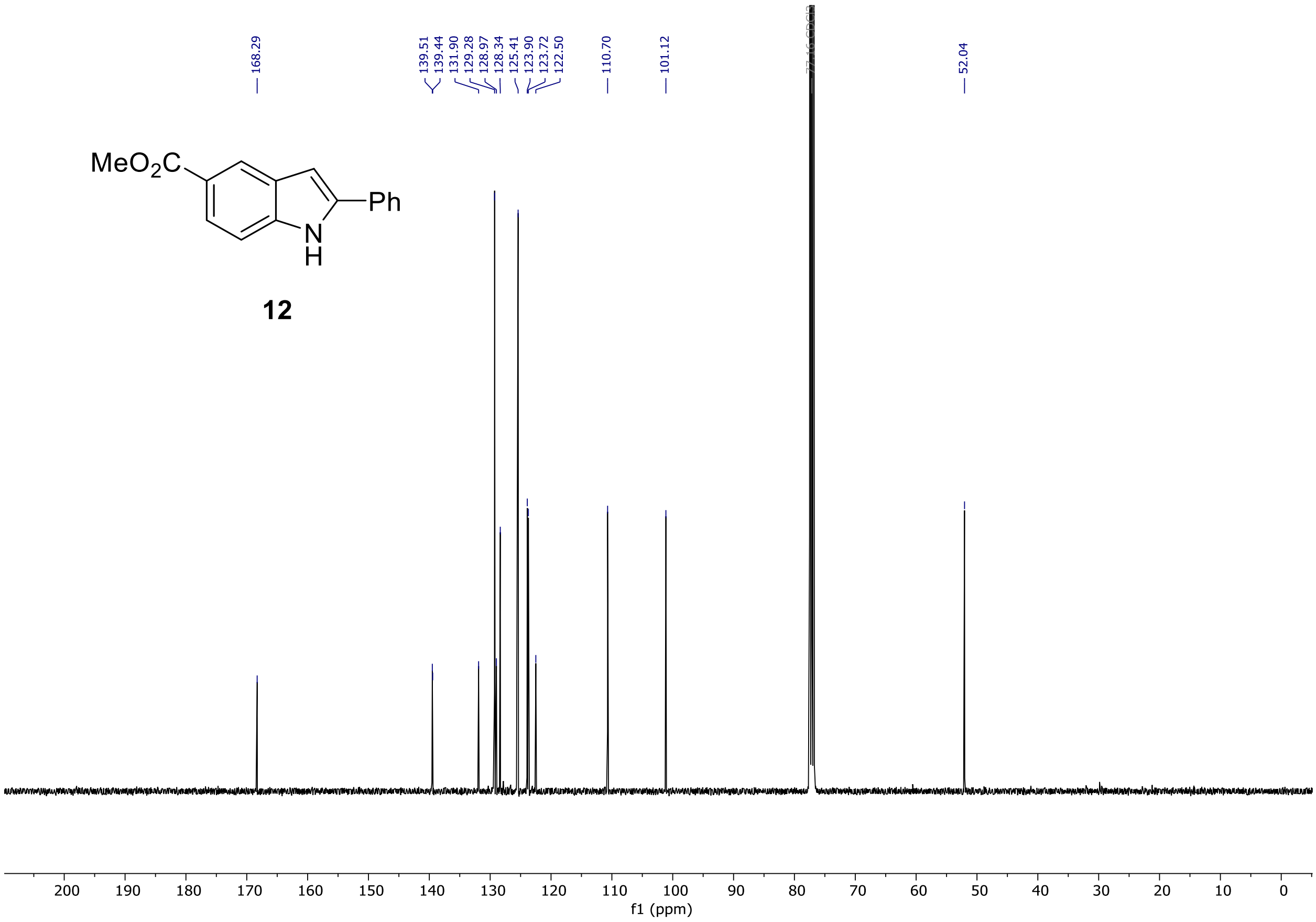

^{1}H NMR (400 MHz, $CDCl_3$) of **13**

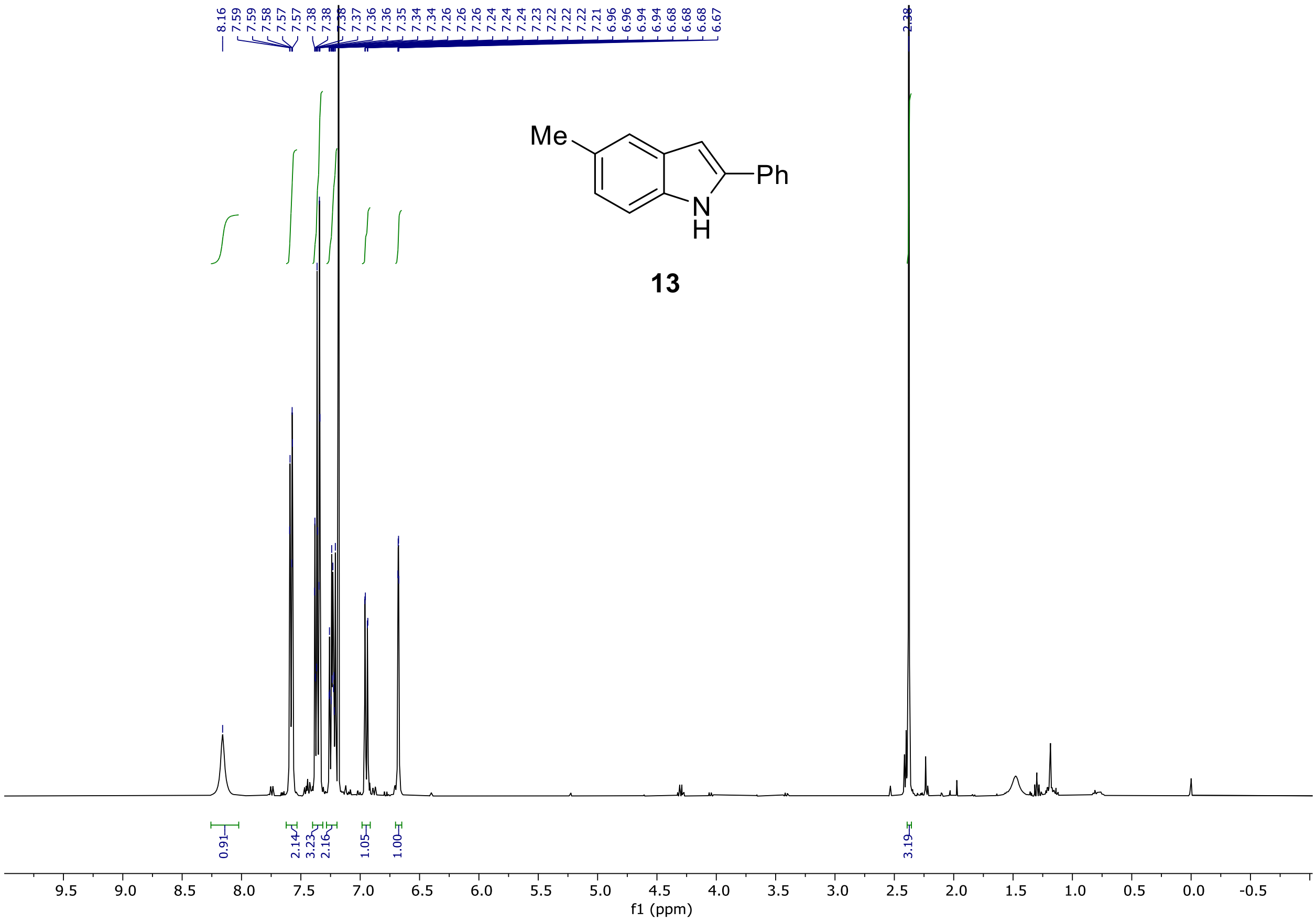

$^{13}C\{^1H\}$ NMR (100 MHz, $CDCl_3$) of **13**

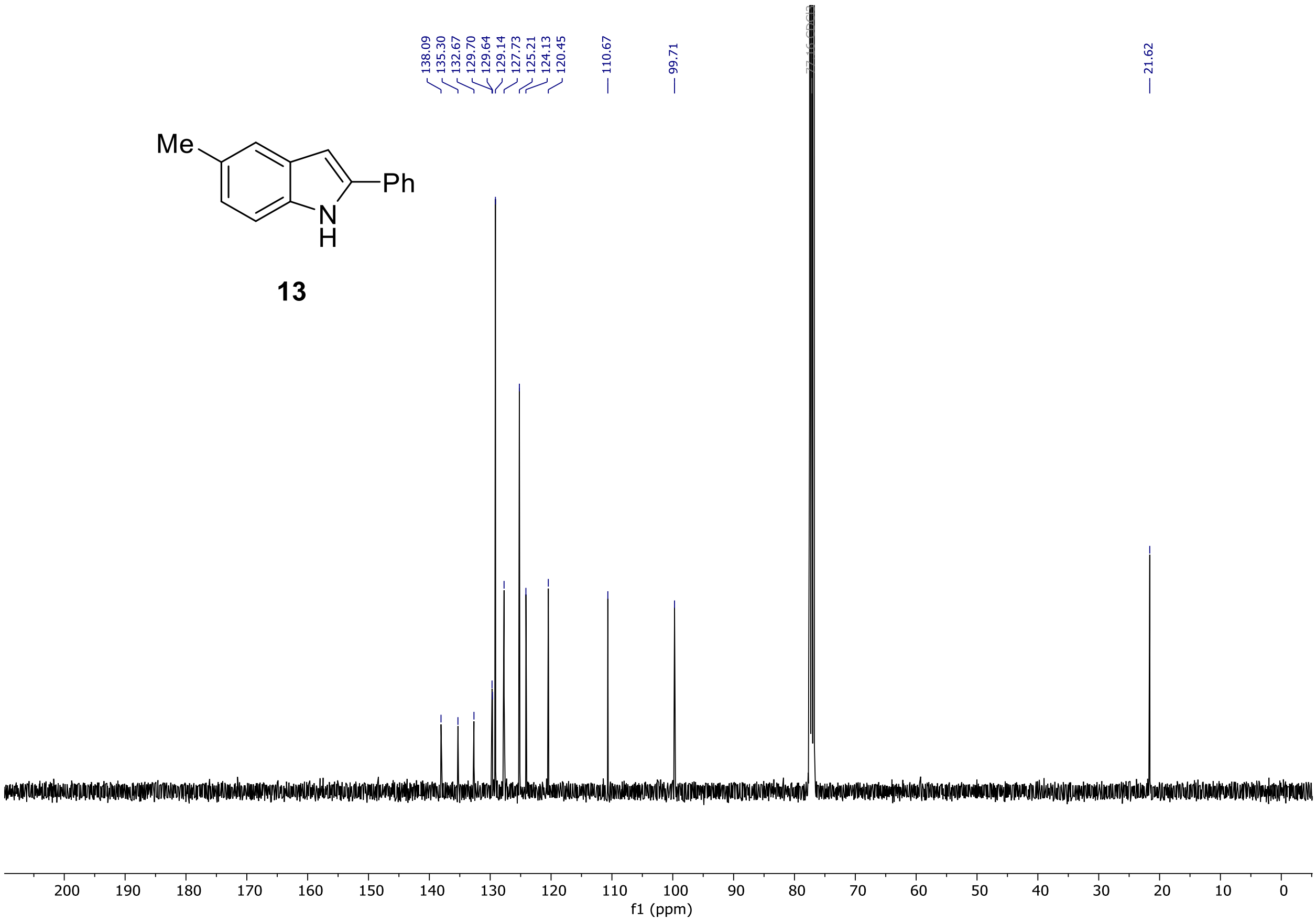

^{1}H NMR (400 MHz, $CDCl_3$) of **14**

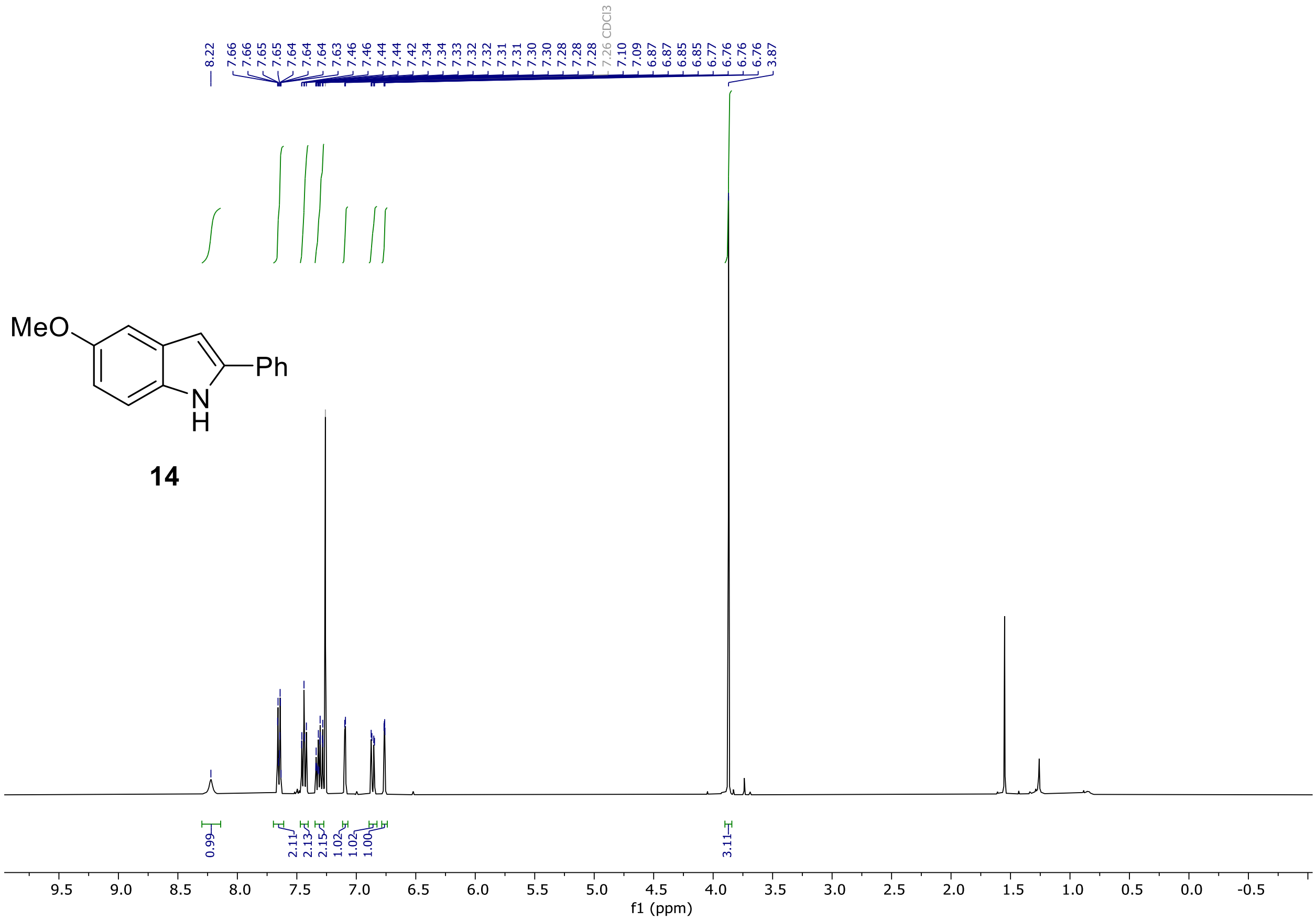

$^{13}C\{^{1}H\}$ NMR (100 MHz, $CDCl_3$) of **14**

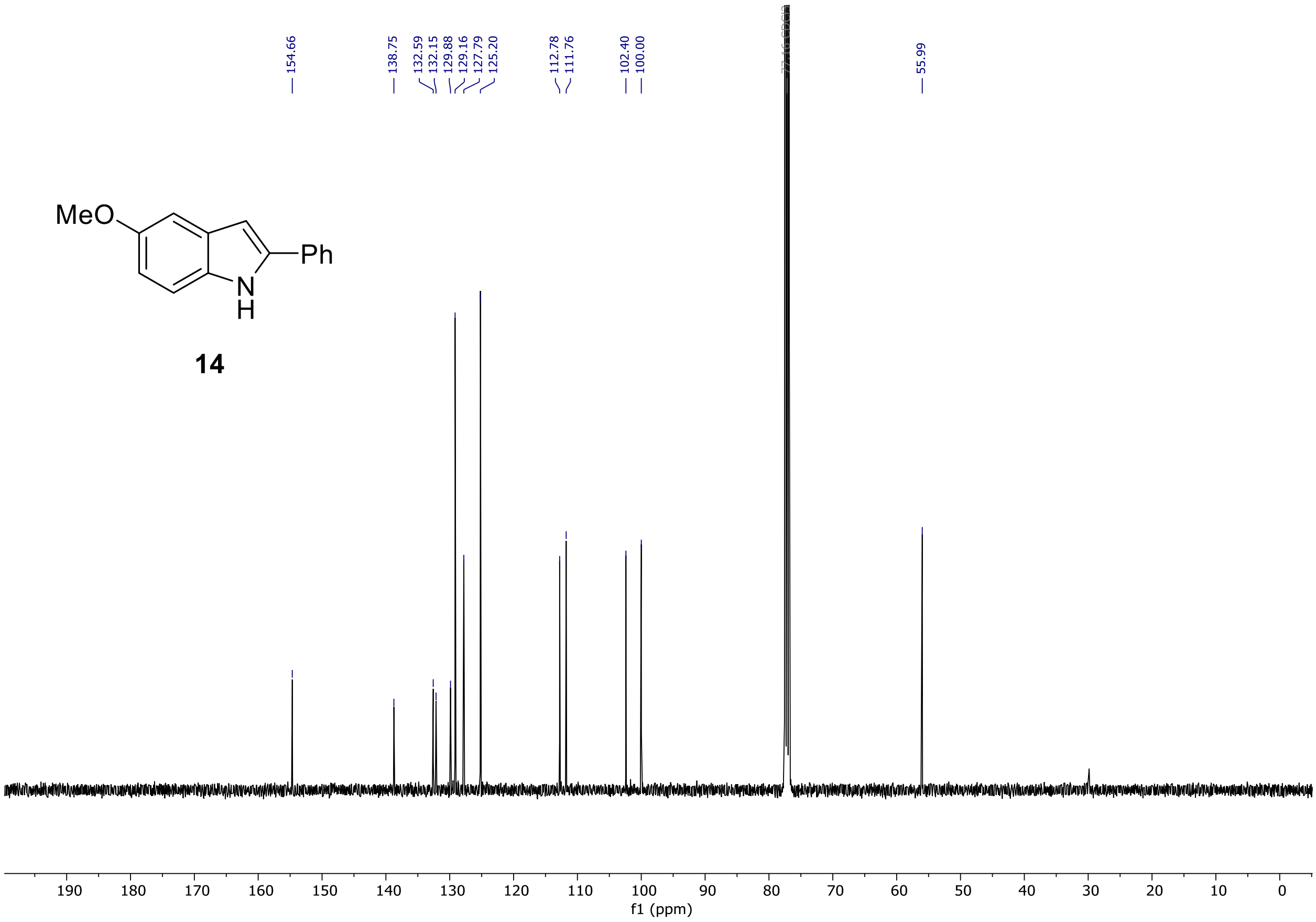

14

^1H NMR (400 MHz, $CDCl_3$) of **15**

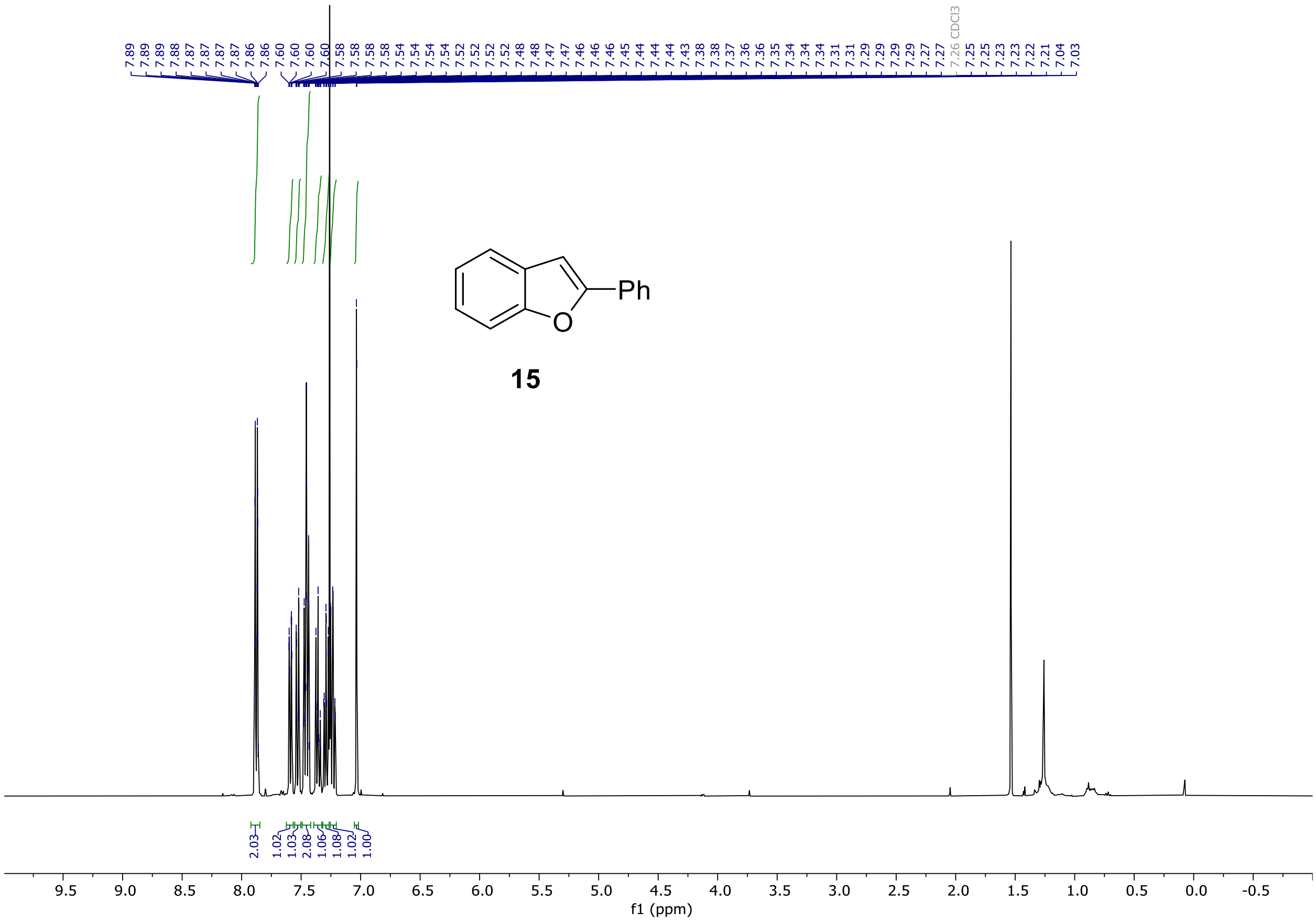

$^{13}C\{^{1}H\}$ NMR (100 MHz, $CDCl_3$) of **15**

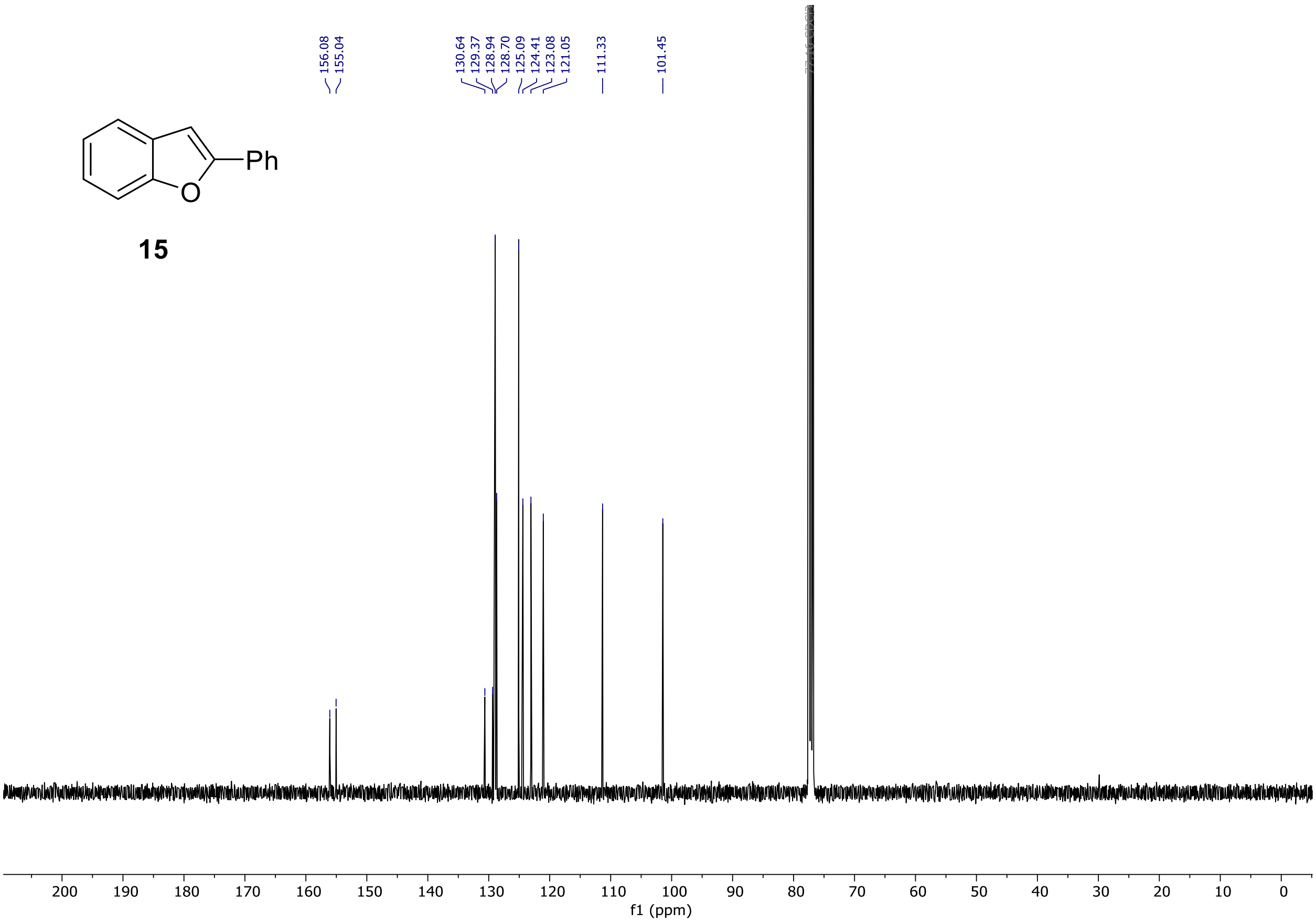

^{1}H NMR (400 MHz, $CDCl_3$) of **16**

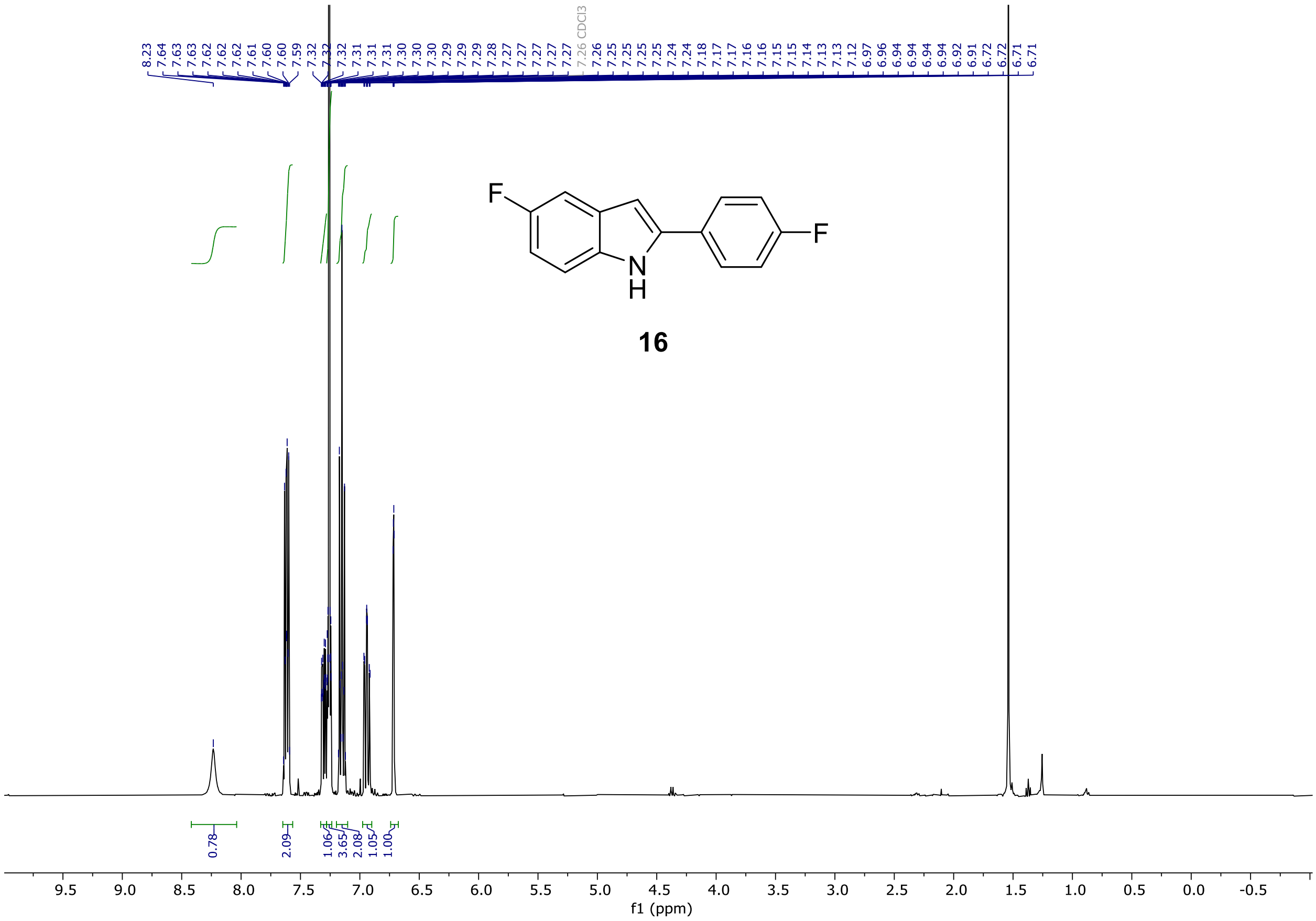

$^{13}C\{^{1}H\}$ NMR (100 MHz, $CDCl_3$) of **16**

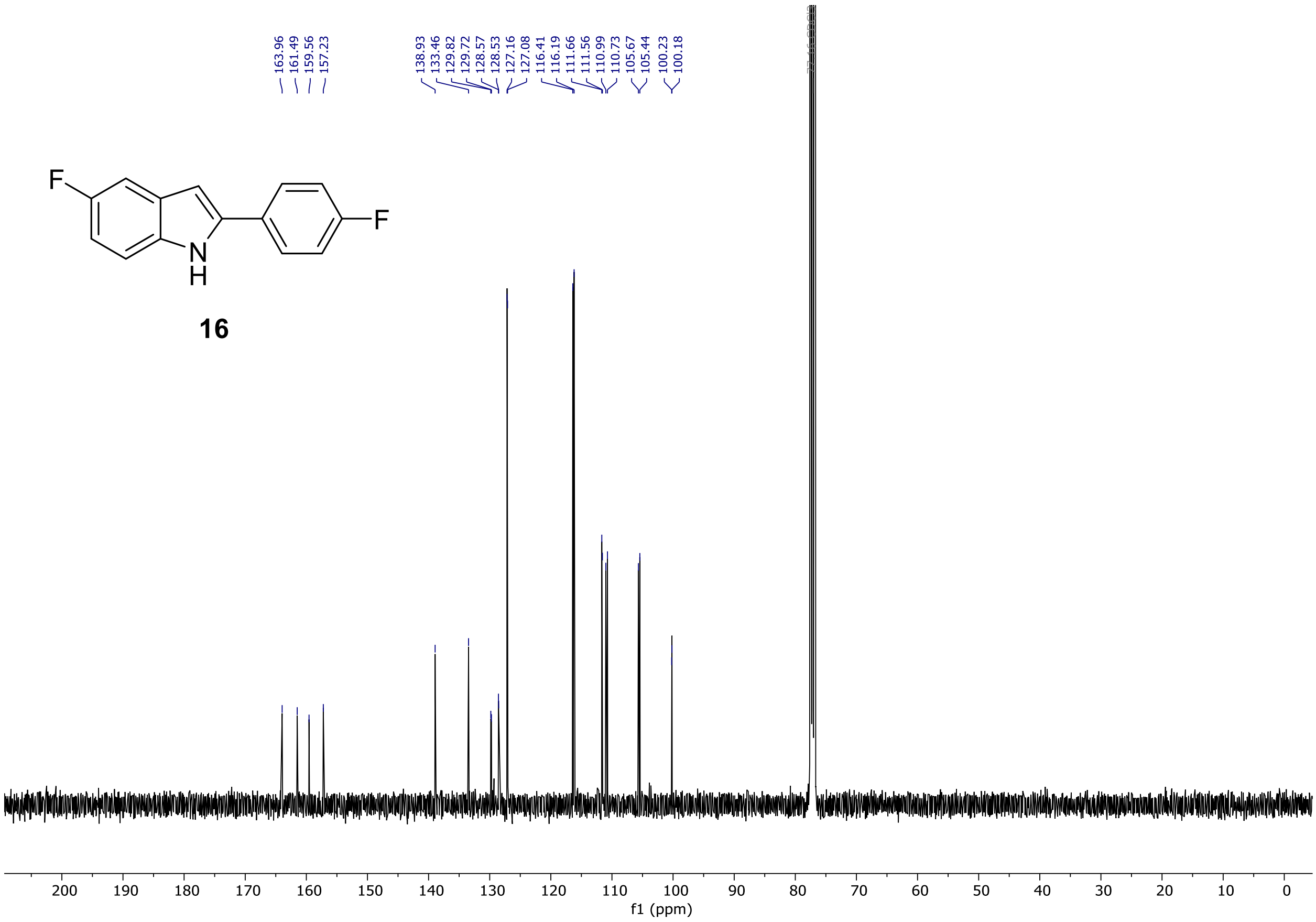

$^{19}F\{^1H\}$ NMR (376 MHz, $CDCl_3$) of **16**

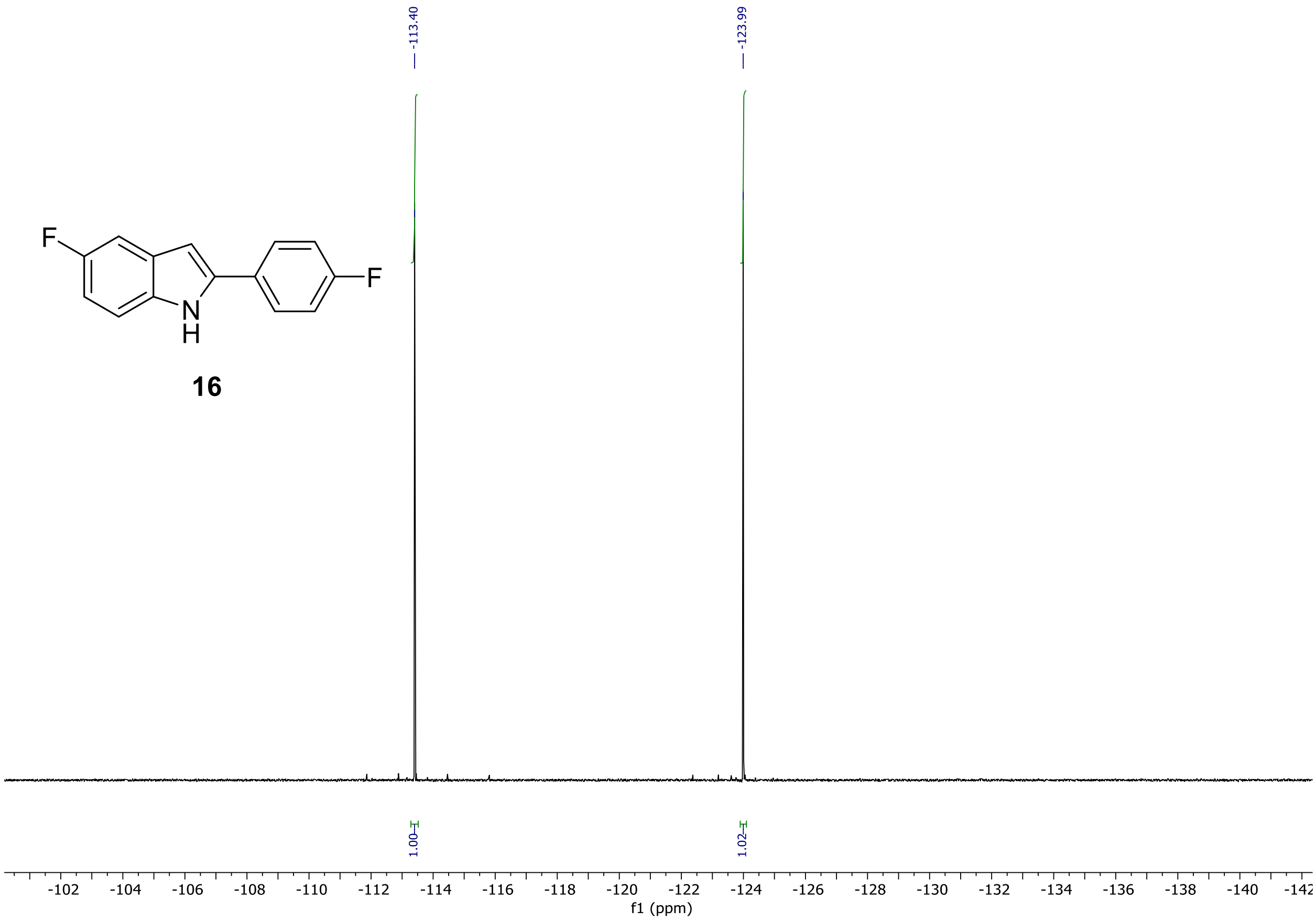

^{1}H NMR (600 MHz, $CDCl_3$) of **17**

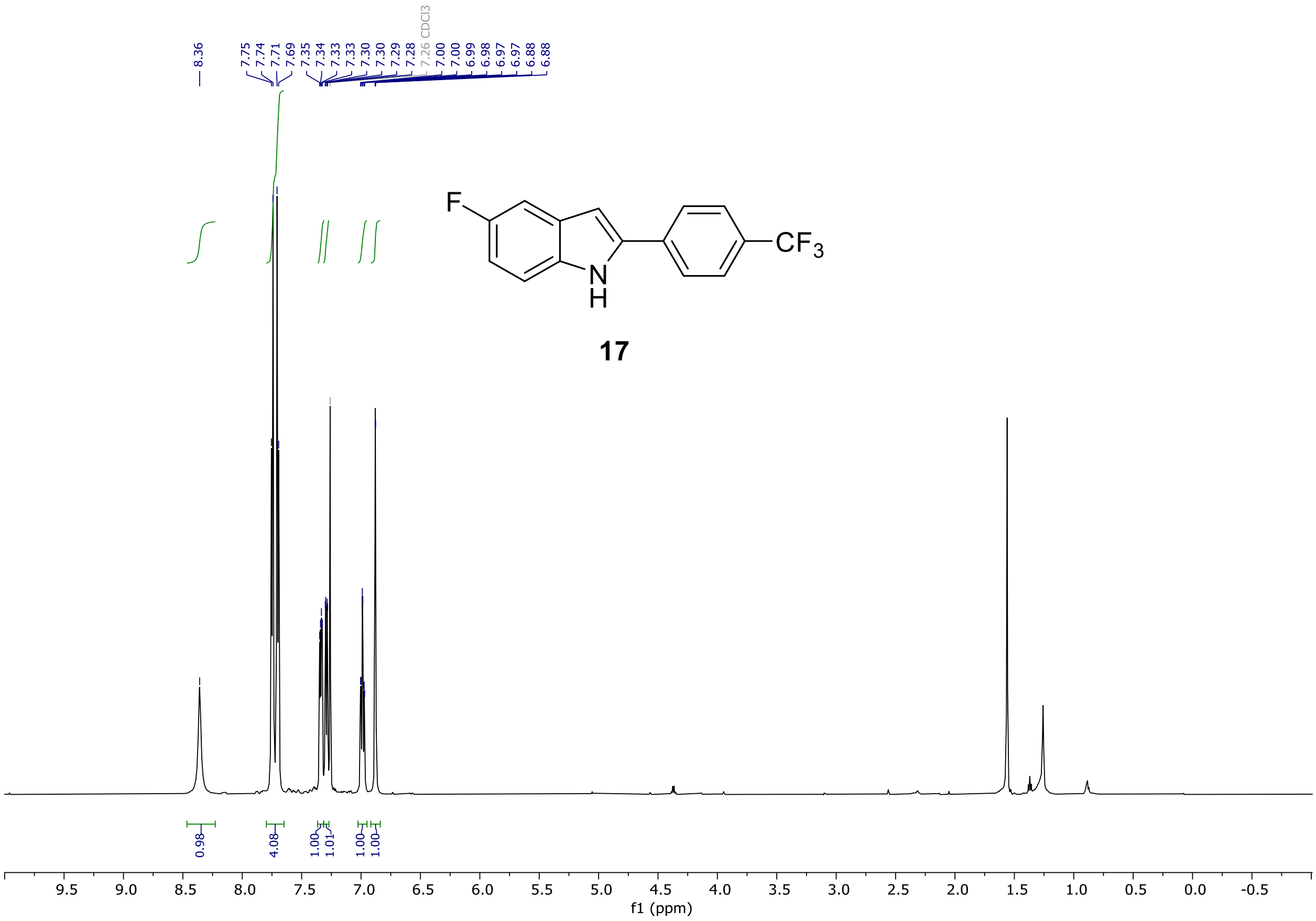

$^{13}C\{^{1}H\}$ NMR (151 MHz, $CDCl_3$) of **17**

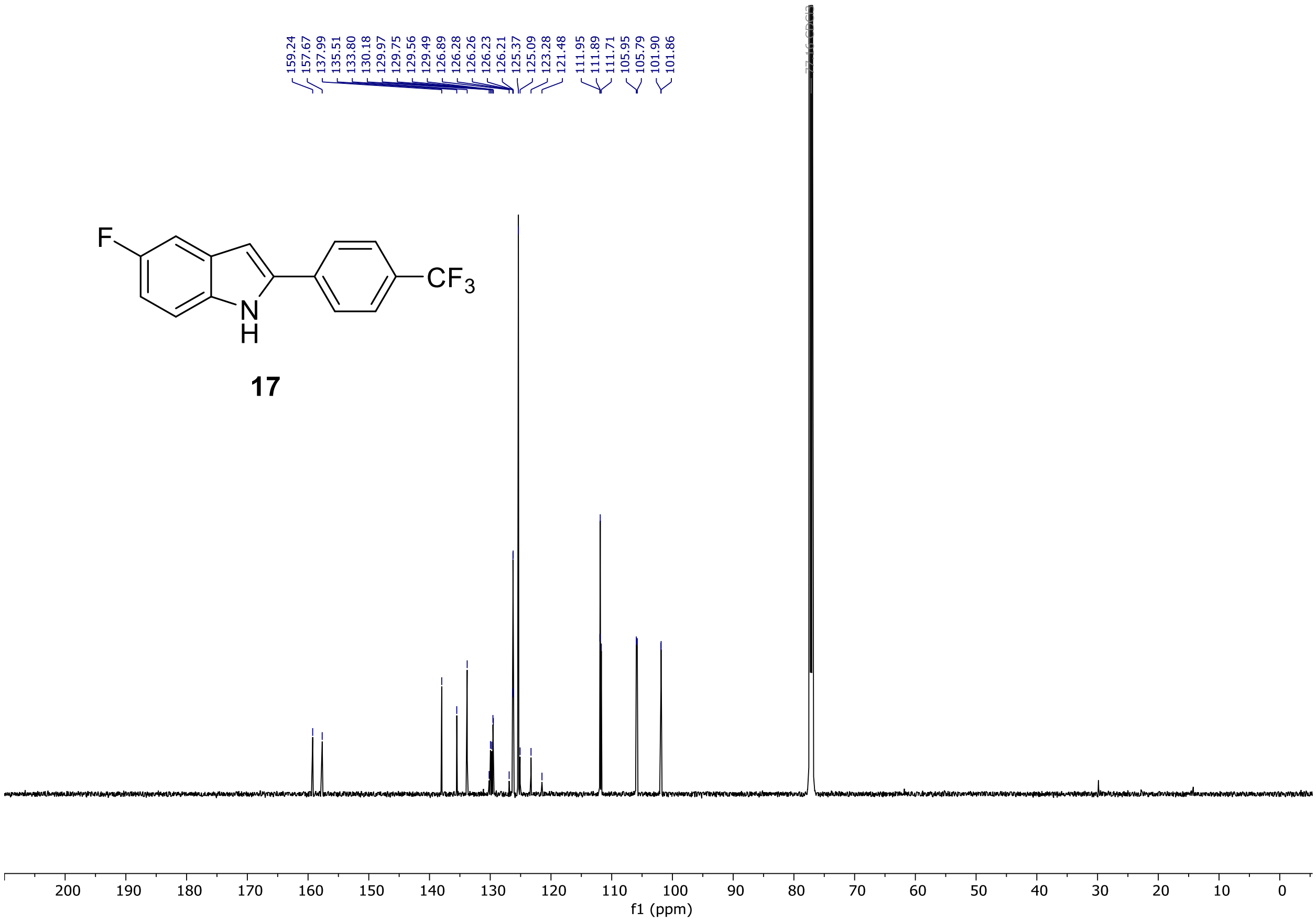

$^{19}F\{^{1}H\}$ NMR (376 MHz, $CDCl_3$) of **17**

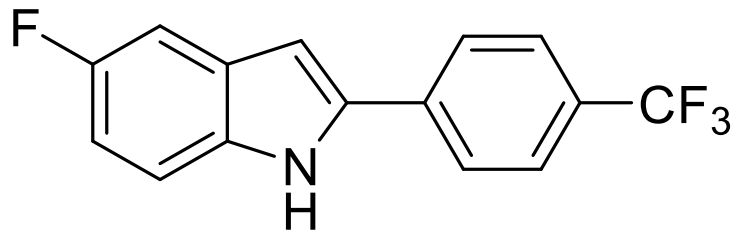

17

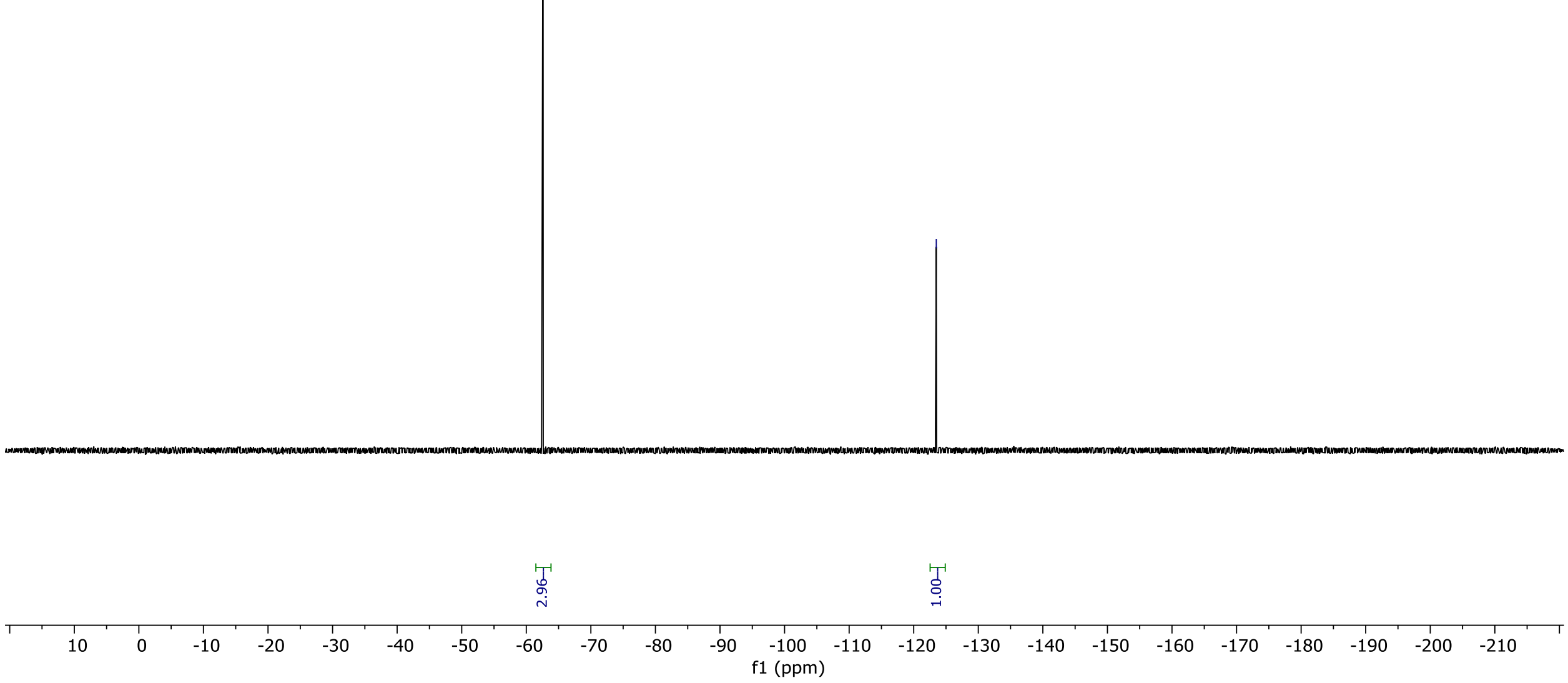

^{1}H NMR (400 MHz, $CDCl_3$) of **18**

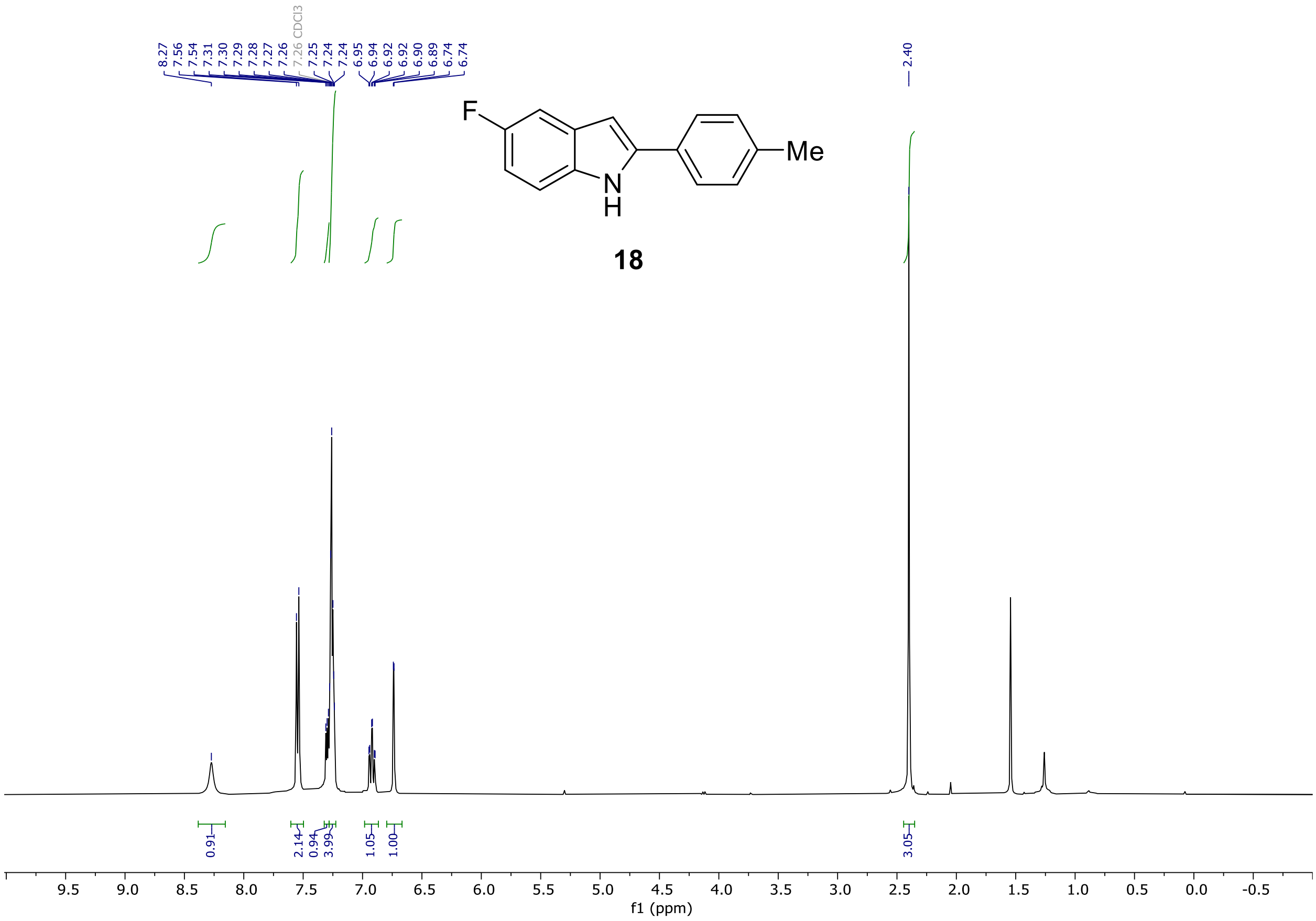

$^{13}C\{^{1}H\}$ NMR (100 MHz, $CDCl_3$) of **18**

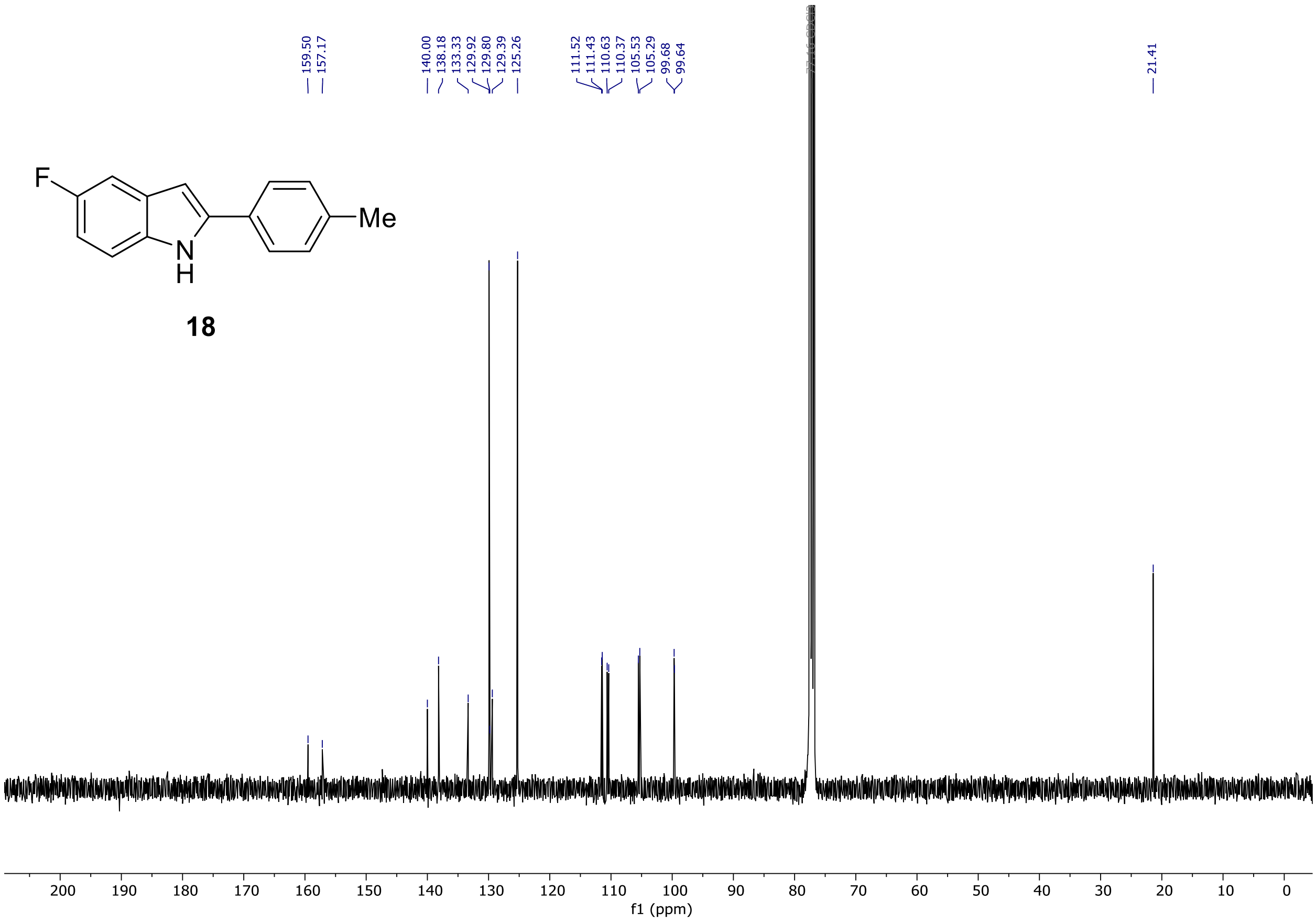

$^{19}F\{^1H\}$ NMR (376 MHz, $CDCl_3$) of **18**

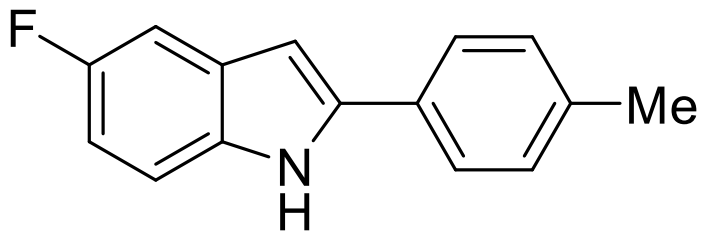

18

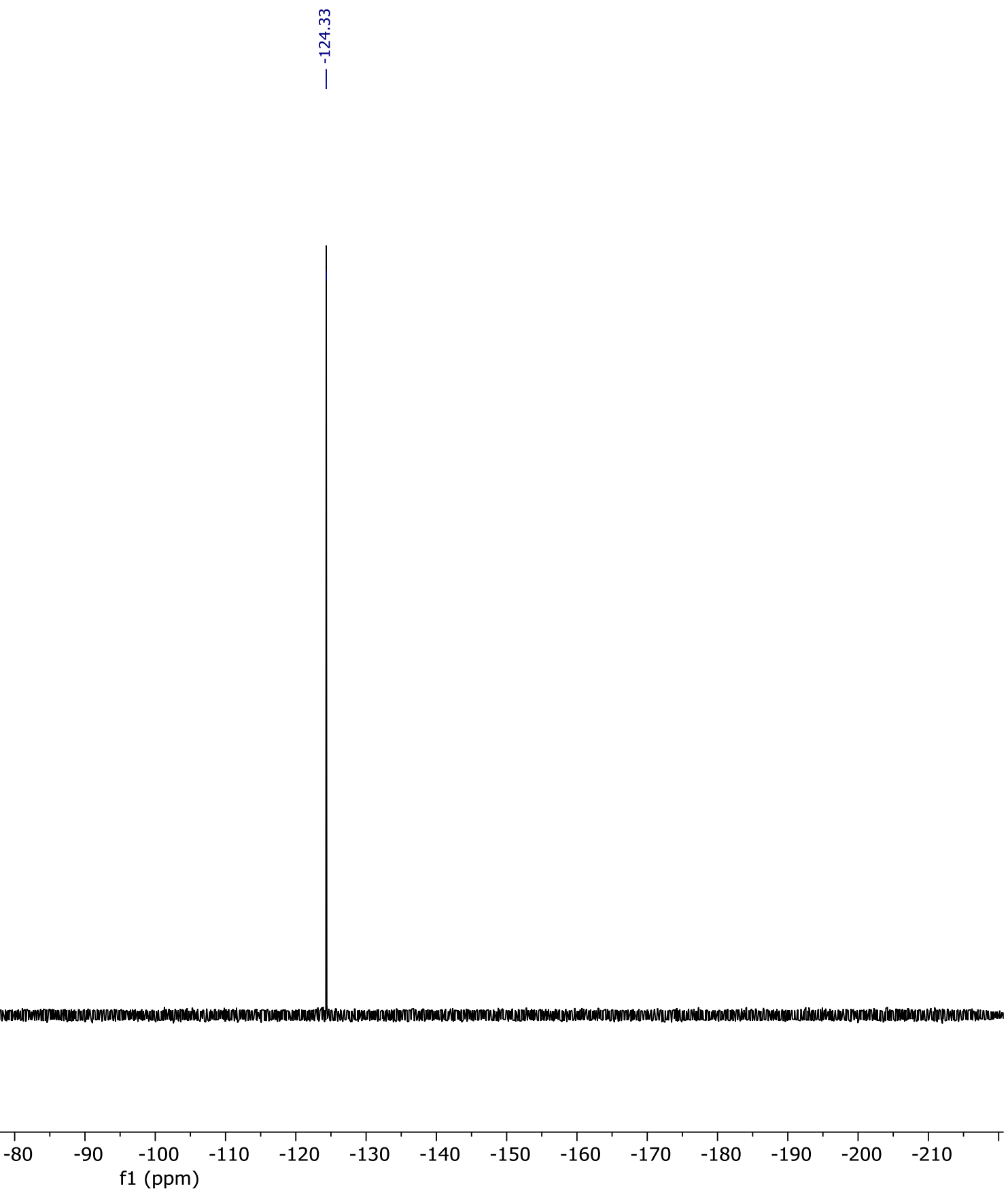

^{1}H NMR (400 MHz, $CDCl_3$) of **S4**

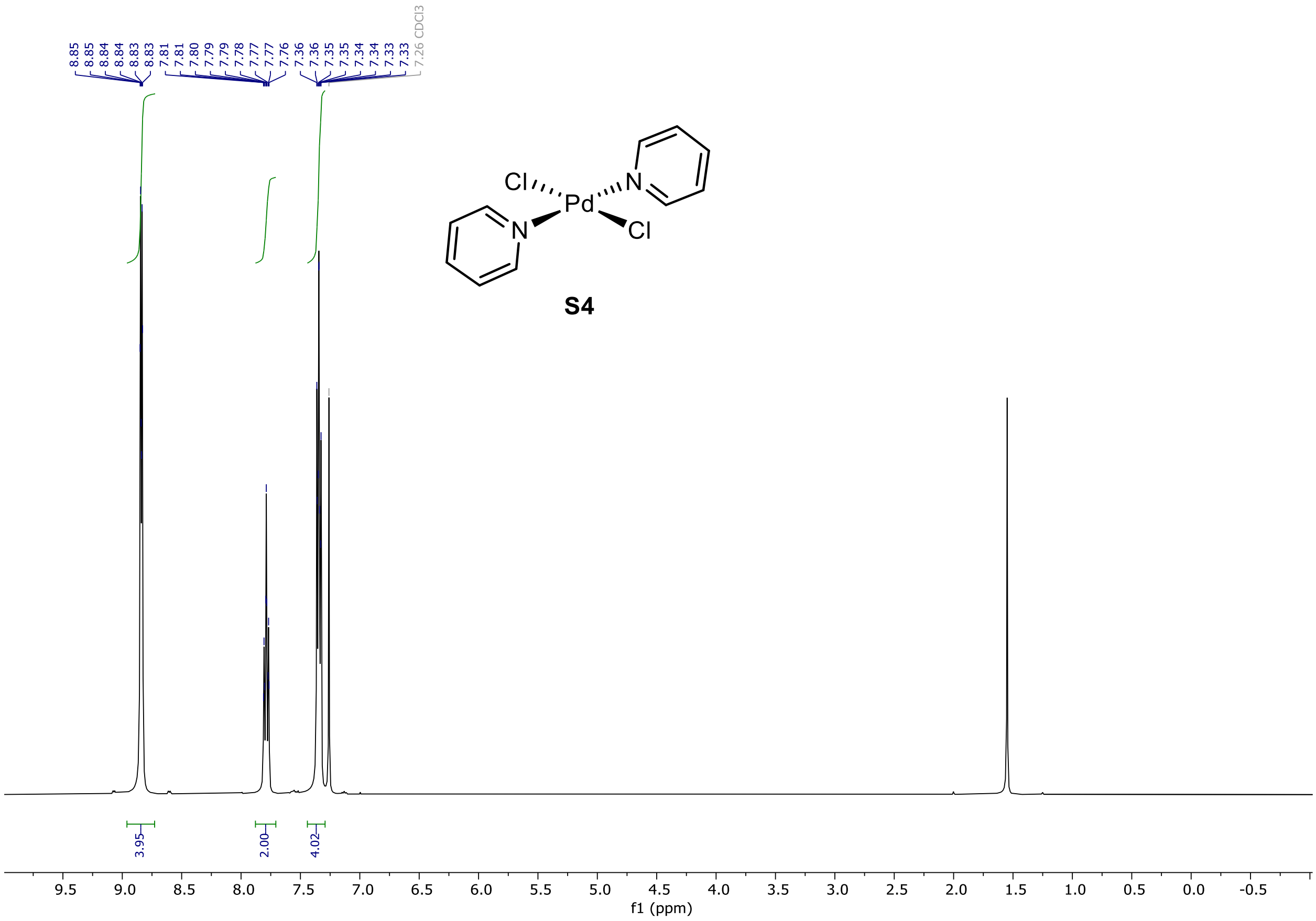

$^{13}C\{^{1}H\}$ NMR (100 MHz, $CDCl_3$) of **S4**

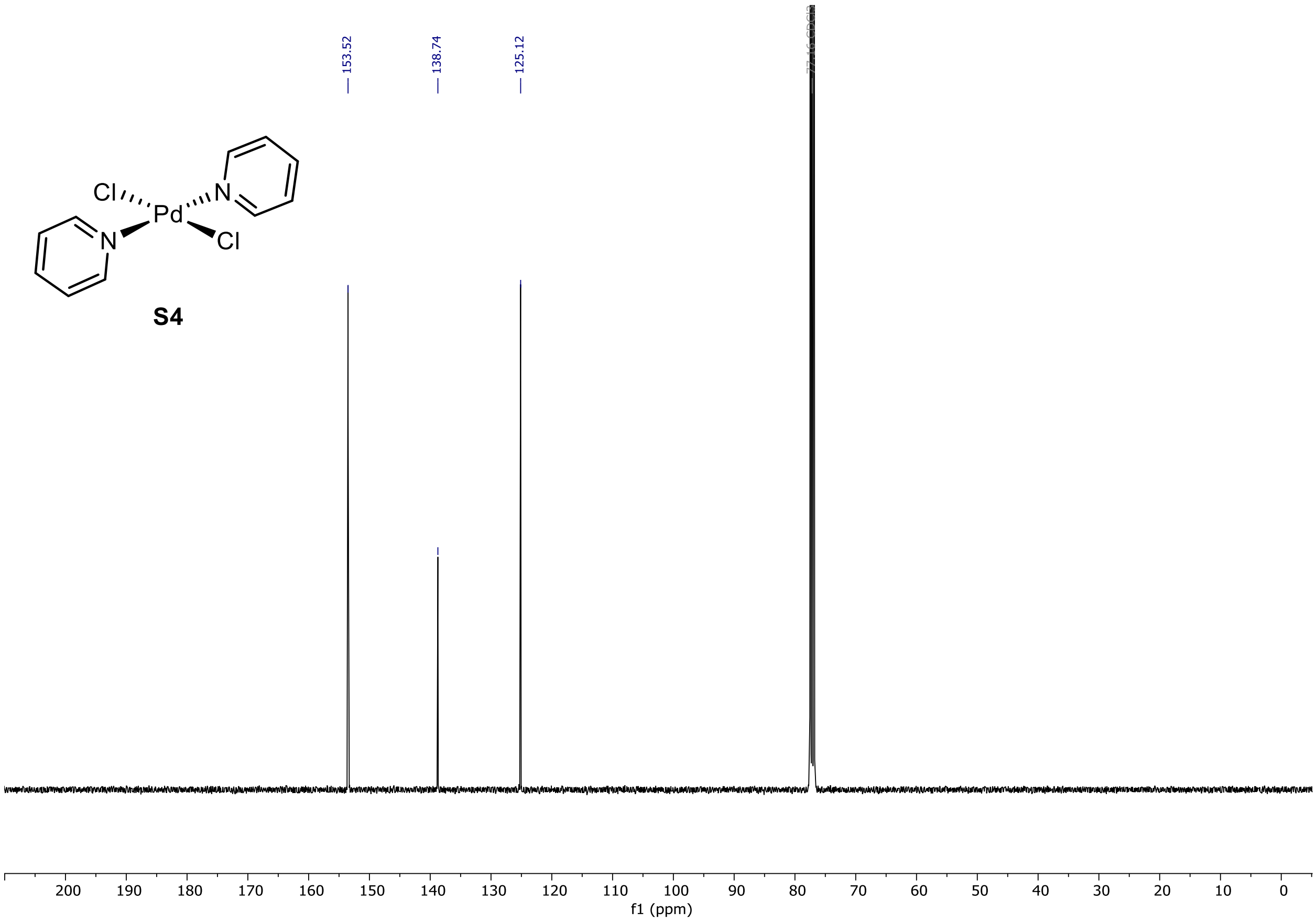

^{1}H NMR (400 MHz, $CDCl_3$) of **S5**

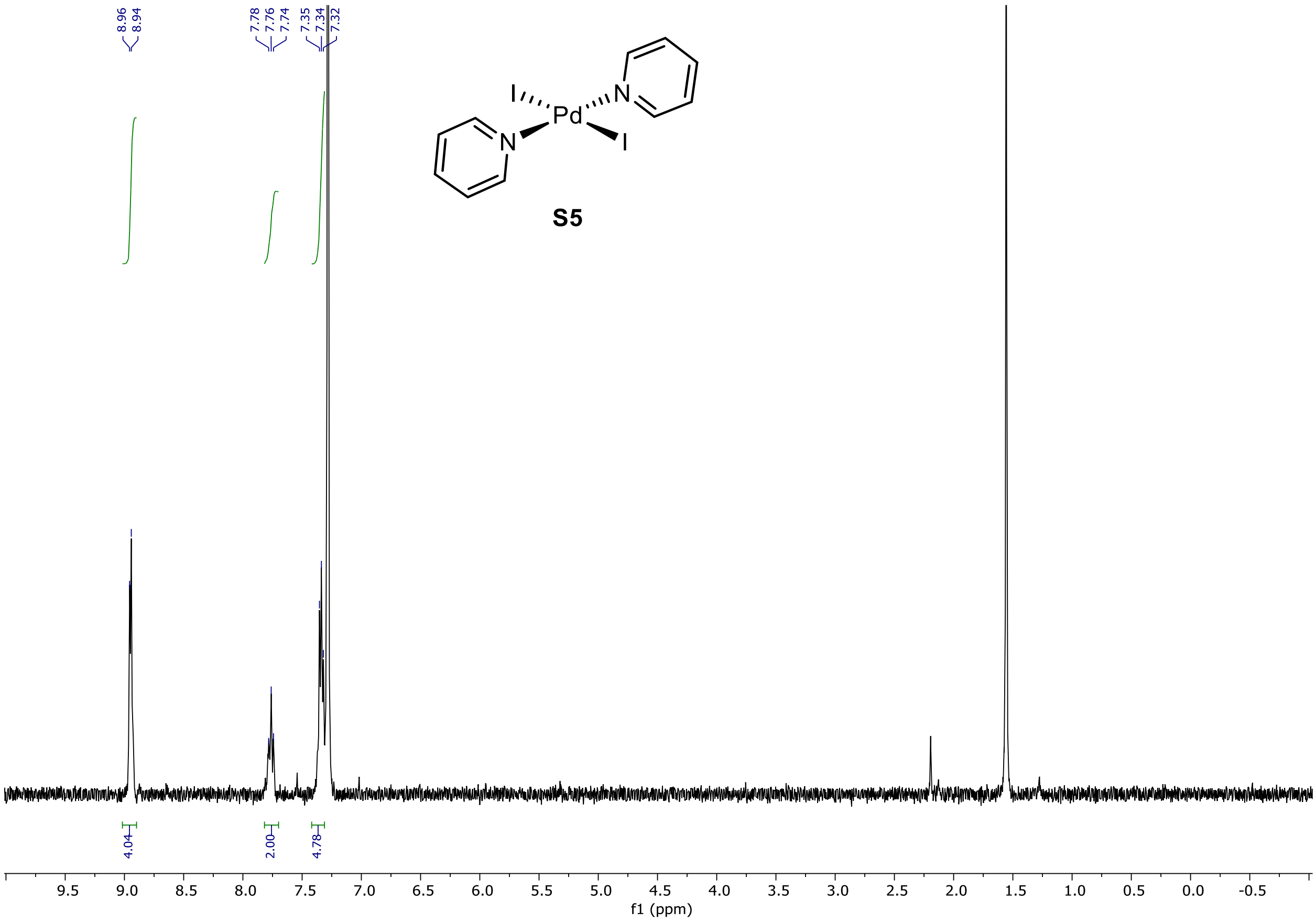

$^{13}C\{^{1}H\}$ NMR (100 MHz, $CDCl_3$) of **S5**

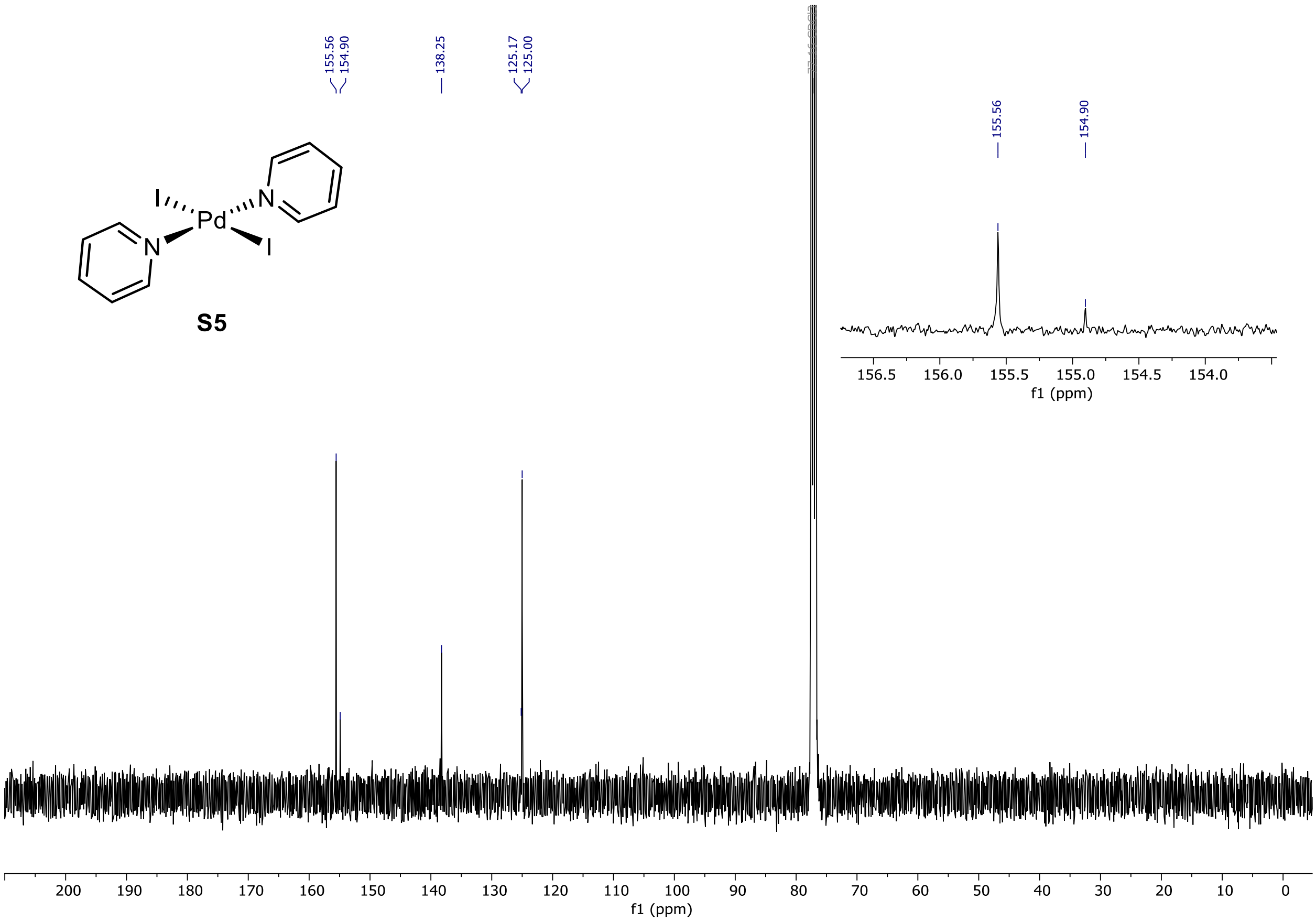

^{1}H NMR (400 MHz, CD_2Cl_2) of **S6**

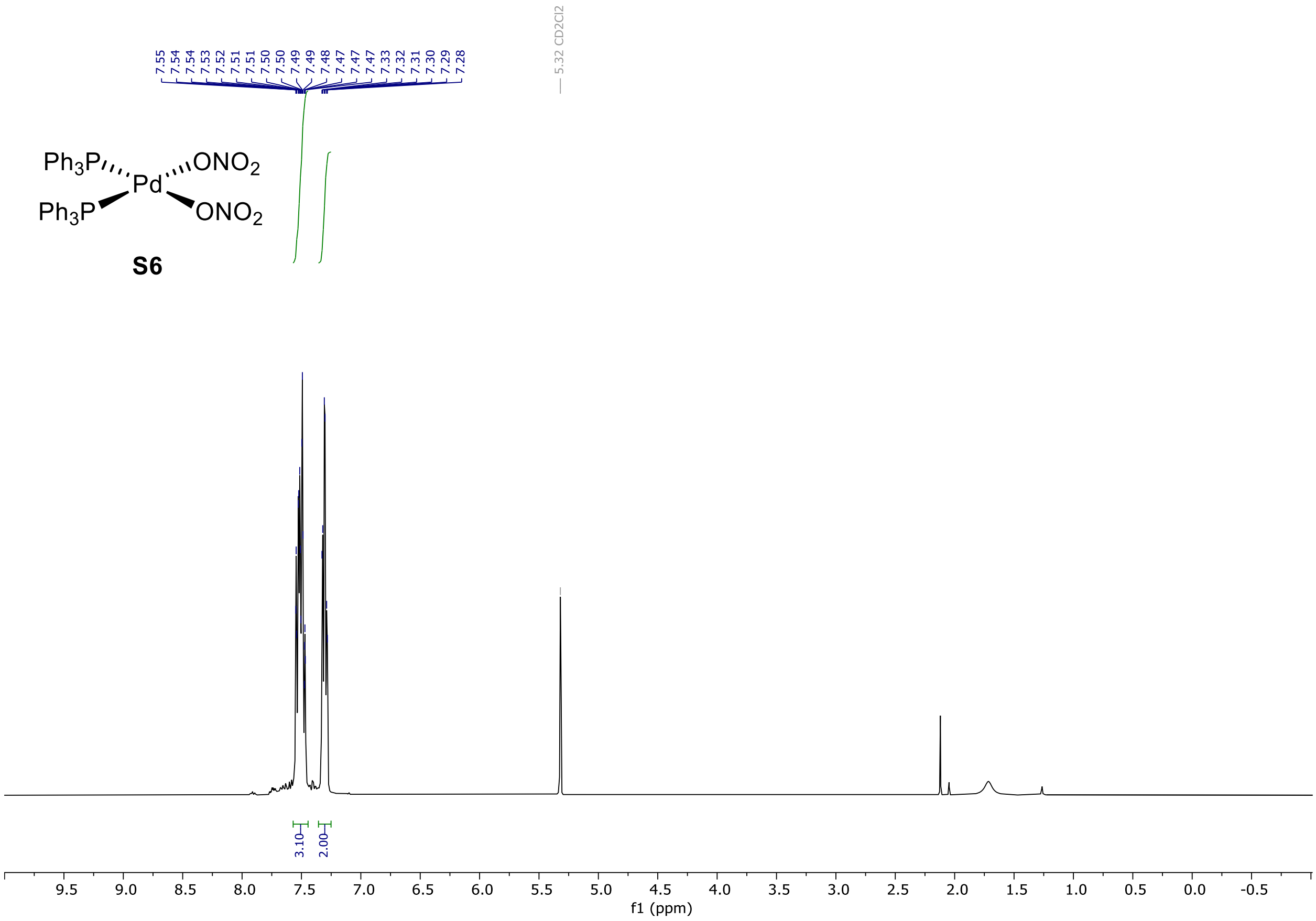

$^{13}C\{^{1}H\}$ NMR (100 MHz, CD_2Cl_2) of **S6**

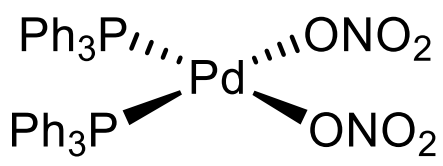

S6

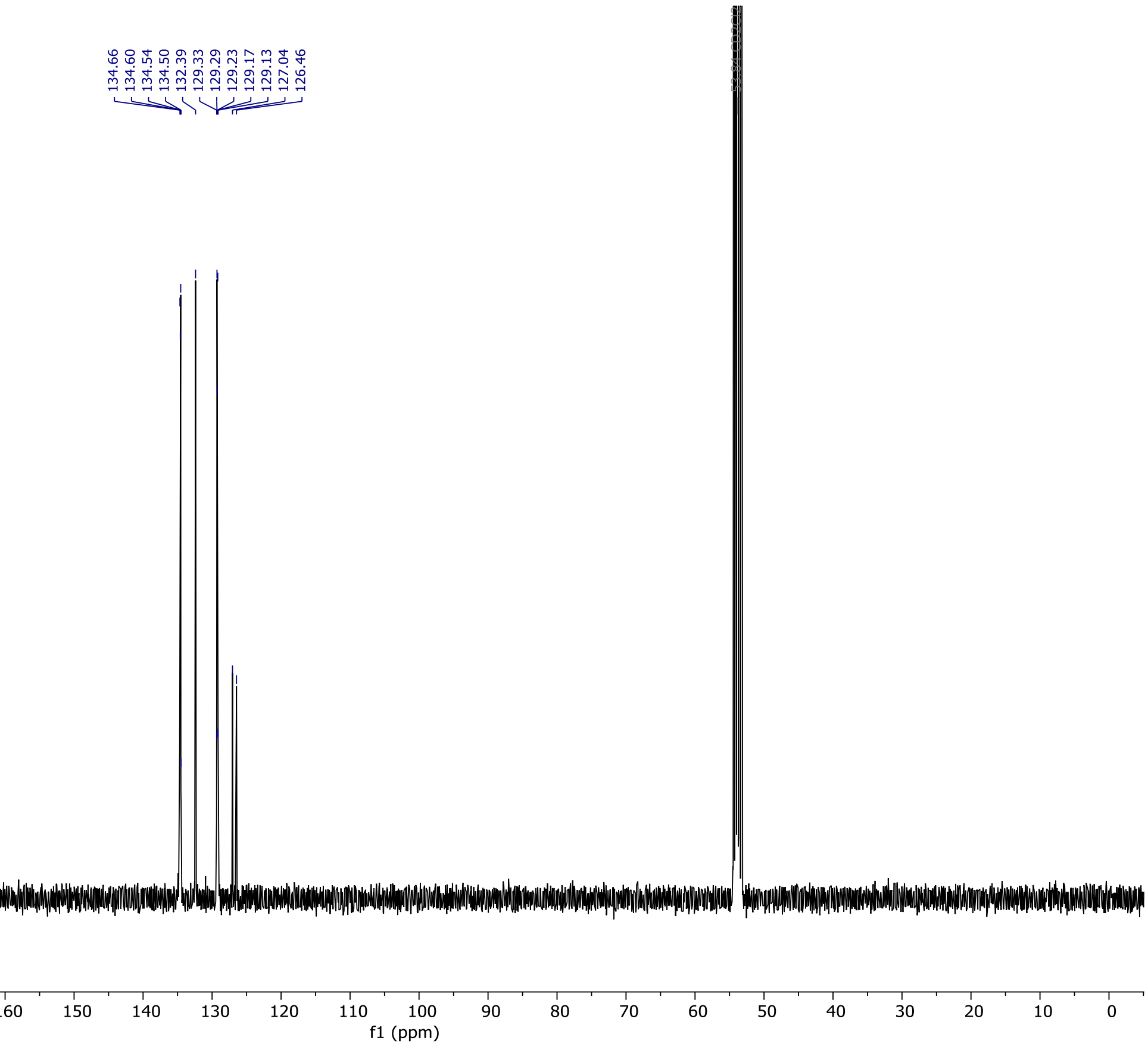

$^{31}P\{^1H\}$ NMR (162 MHz, CD_2Cl_2) of **S6**

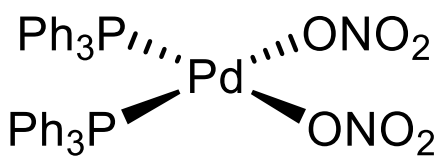

S6

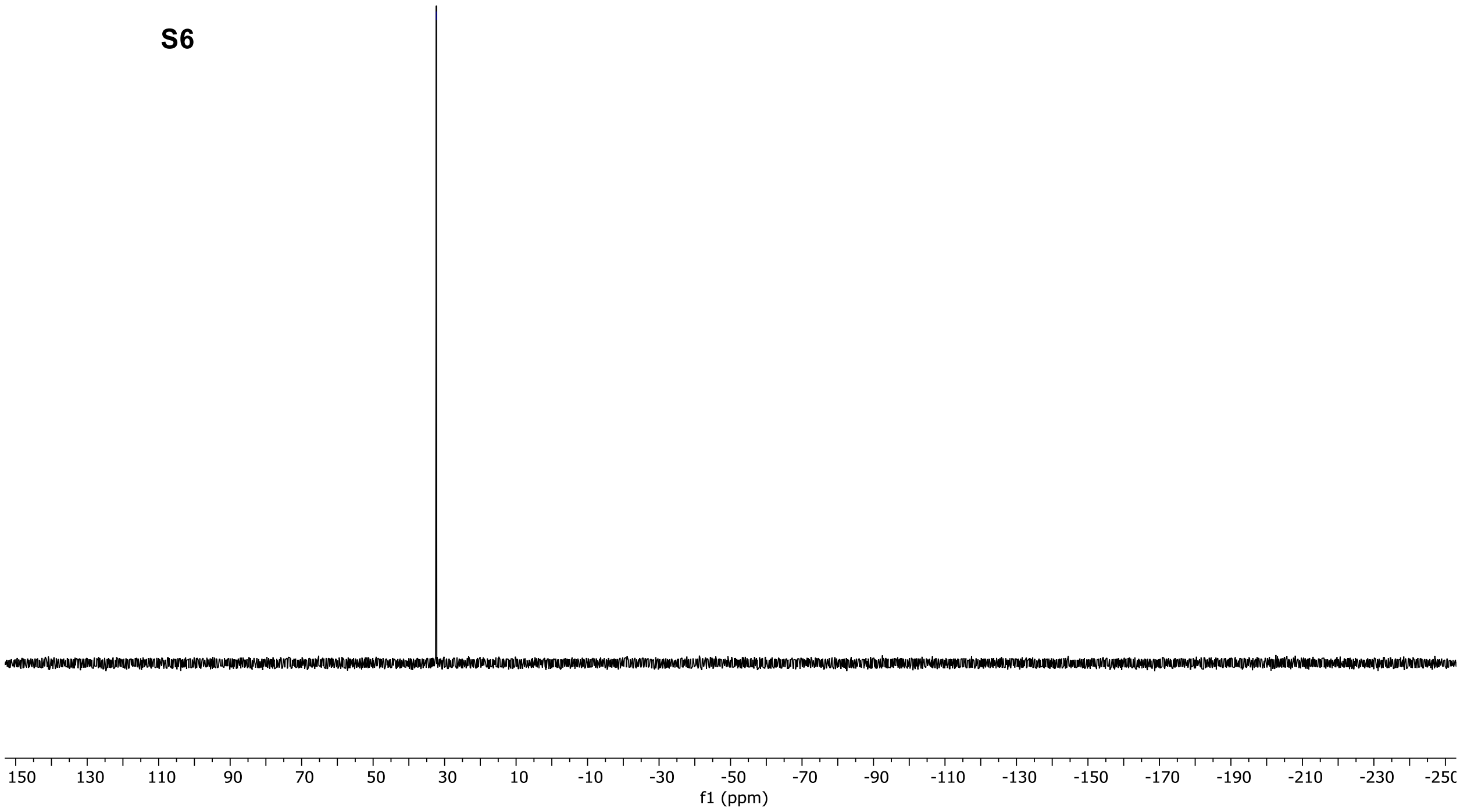

^{1}H NMR (600 MHz, DMSO) of **P3**

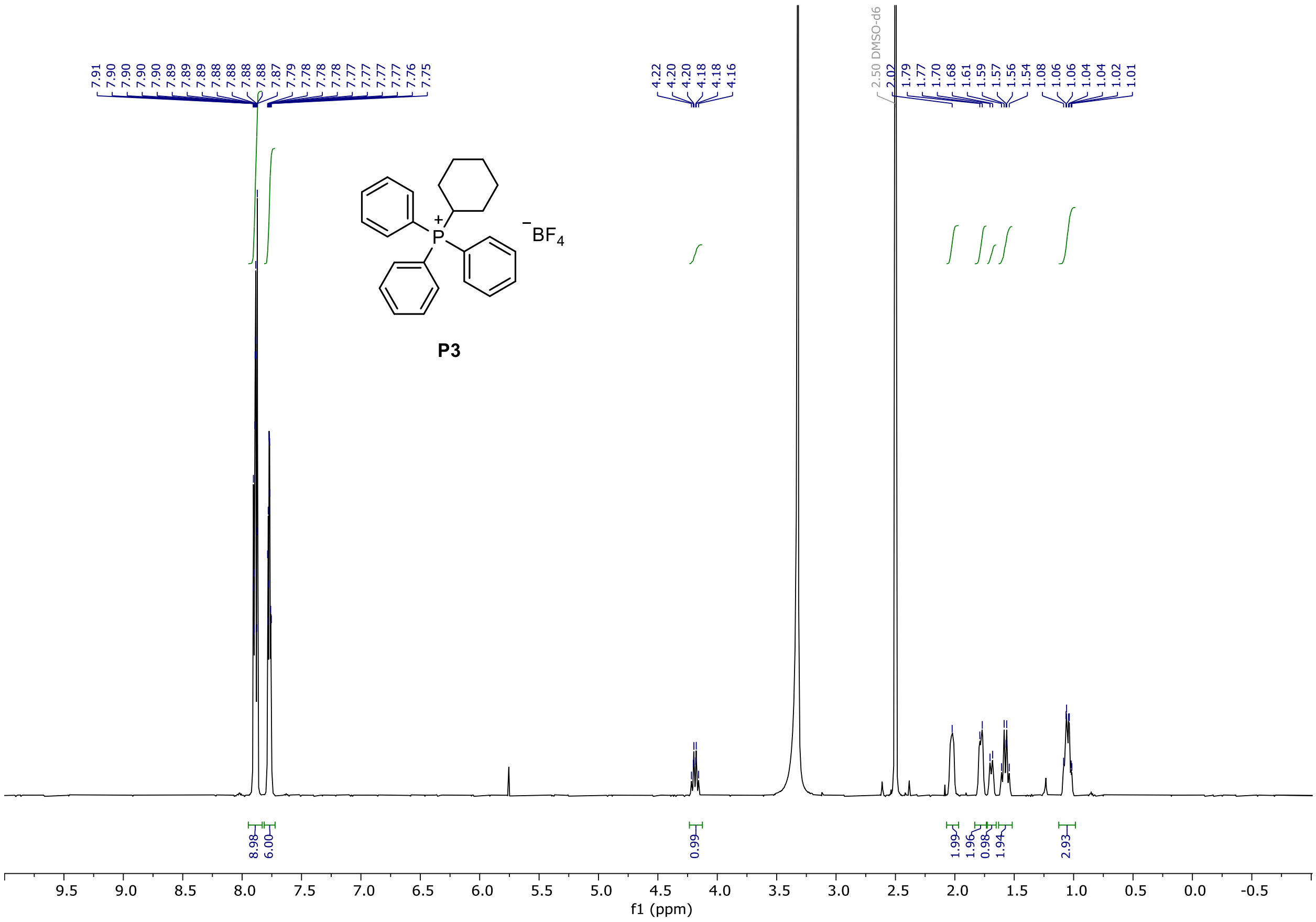

$^{13}C\{^{1}H\}$ NMR (151 MHz, DMSO) of **P3**

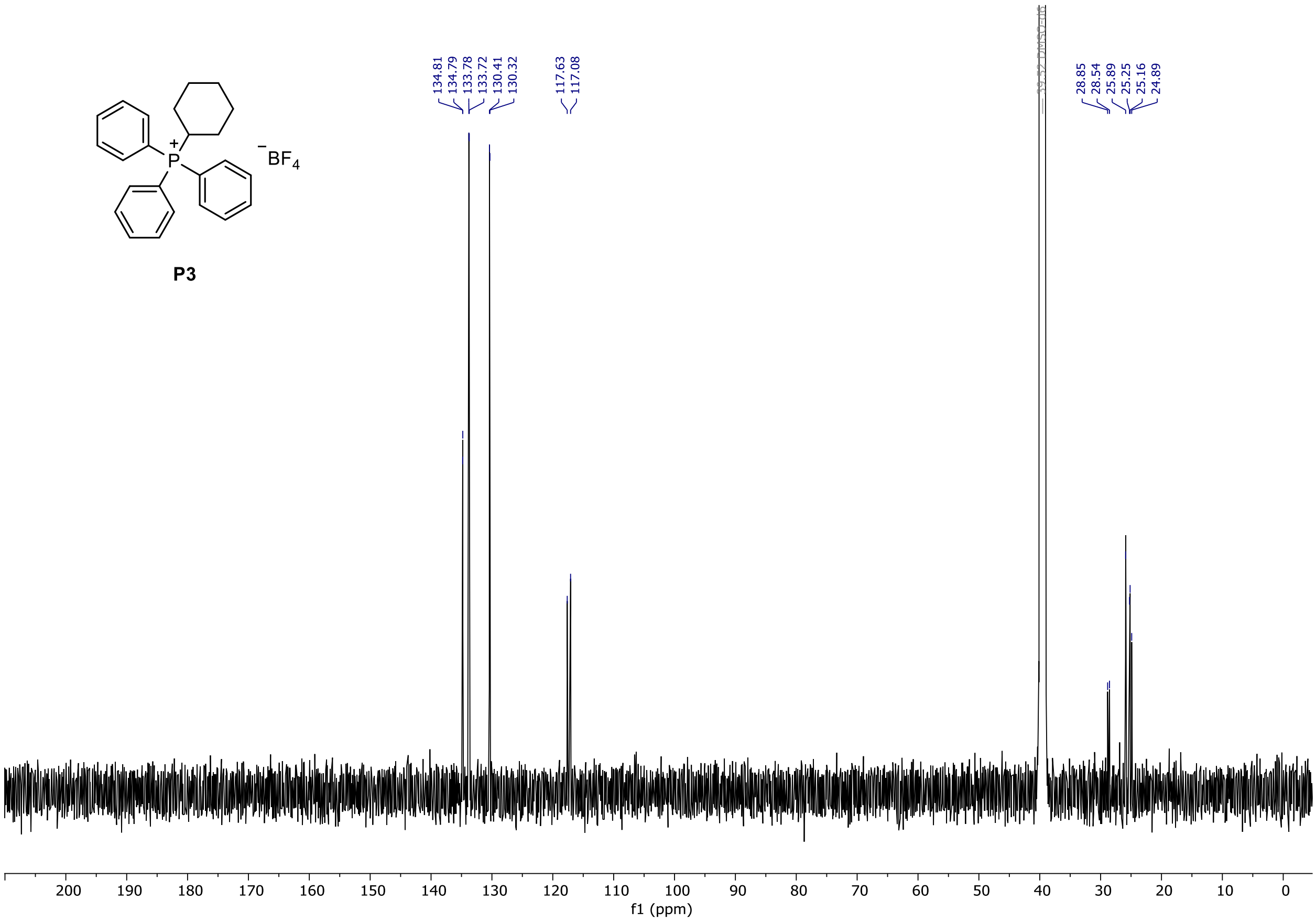

P3

$^{19}F\{^{1}H\}$ NMR (376 MHz, DMSO) of **P3**

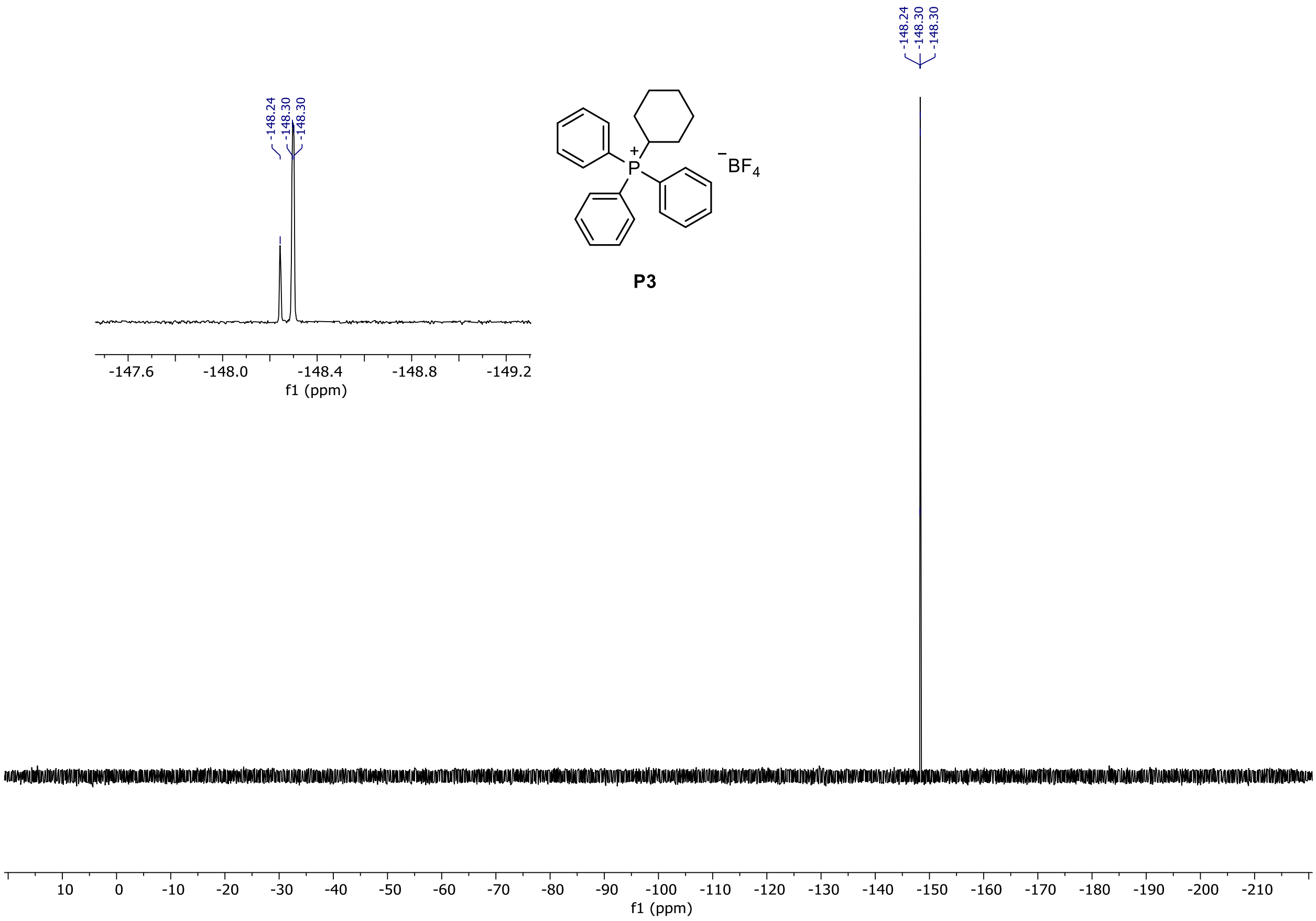

^{31}P NMR (162 MHz, DMSO) of **P3**

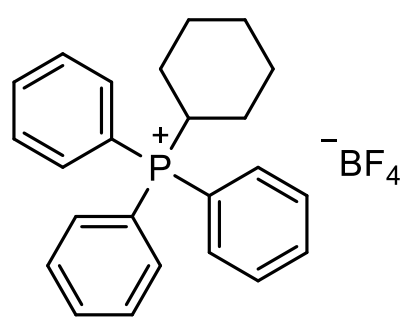

P3

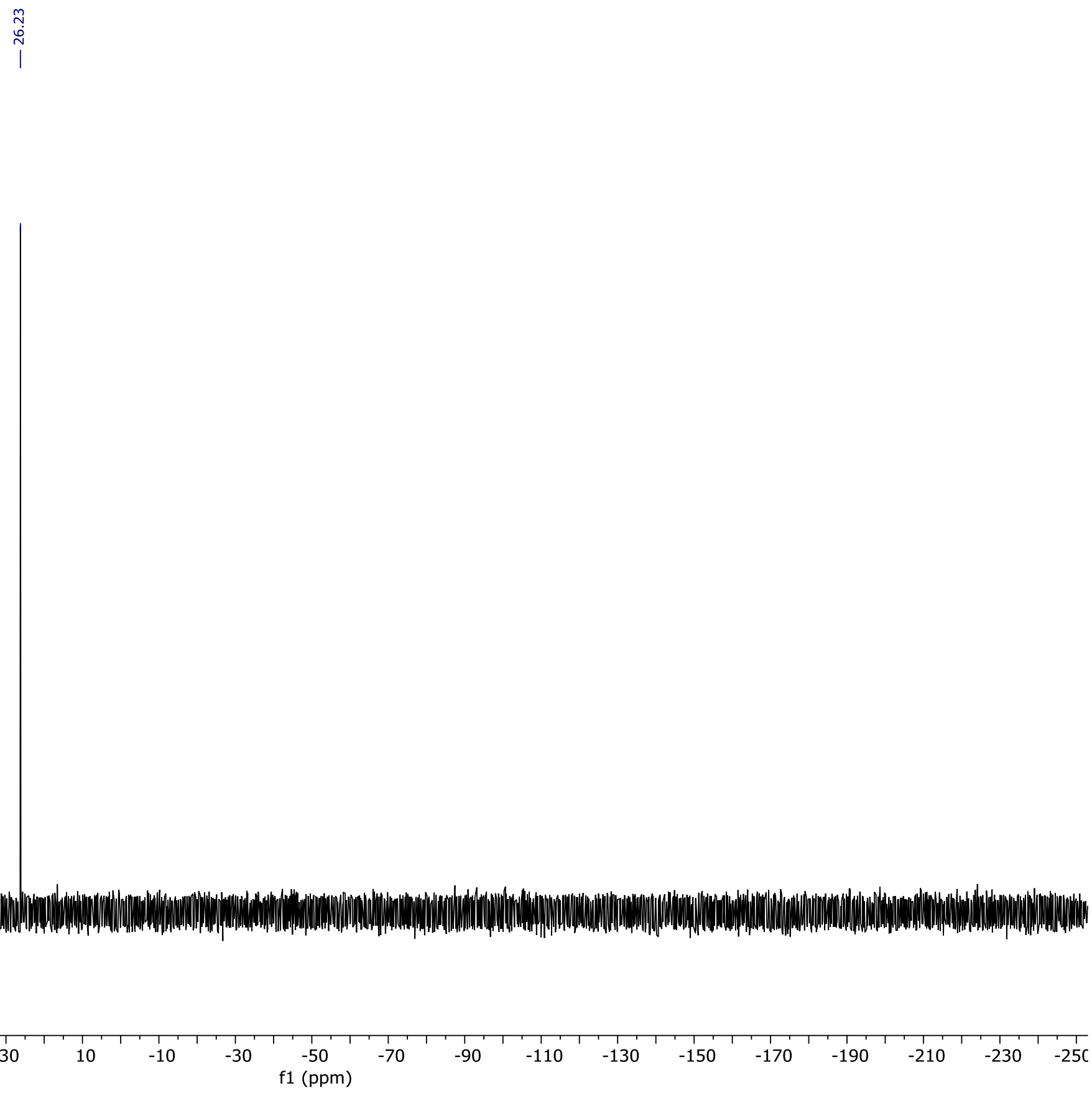

^{1}H NMR (400 MHz, $CDCl_3$) of **S9**

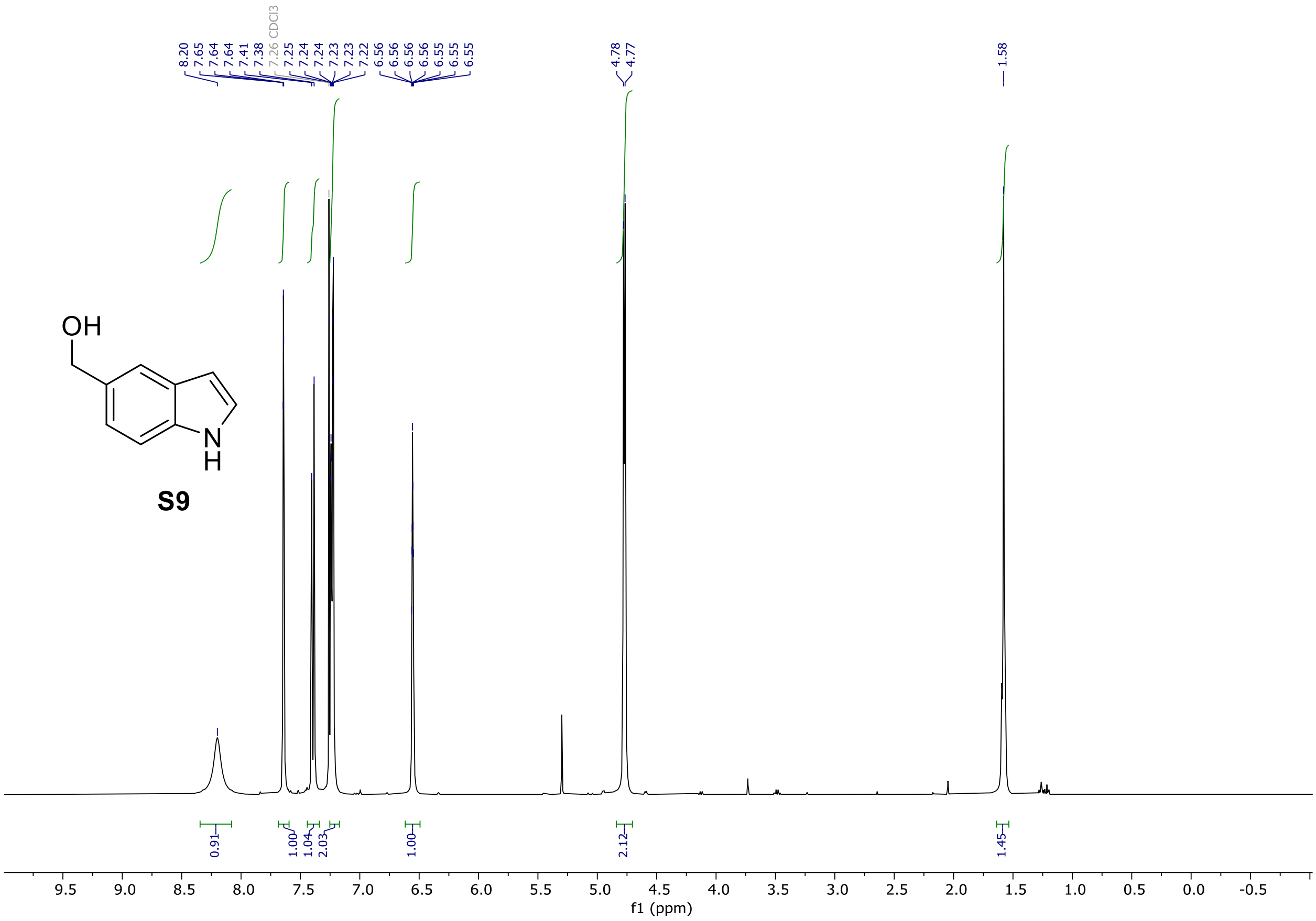

$^{13}C\{^{1}H\}$ NMR (100 MHz, $CDCl_3$) of **S9**

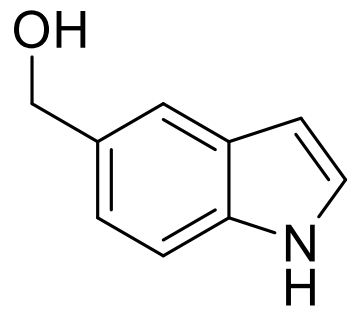

S9

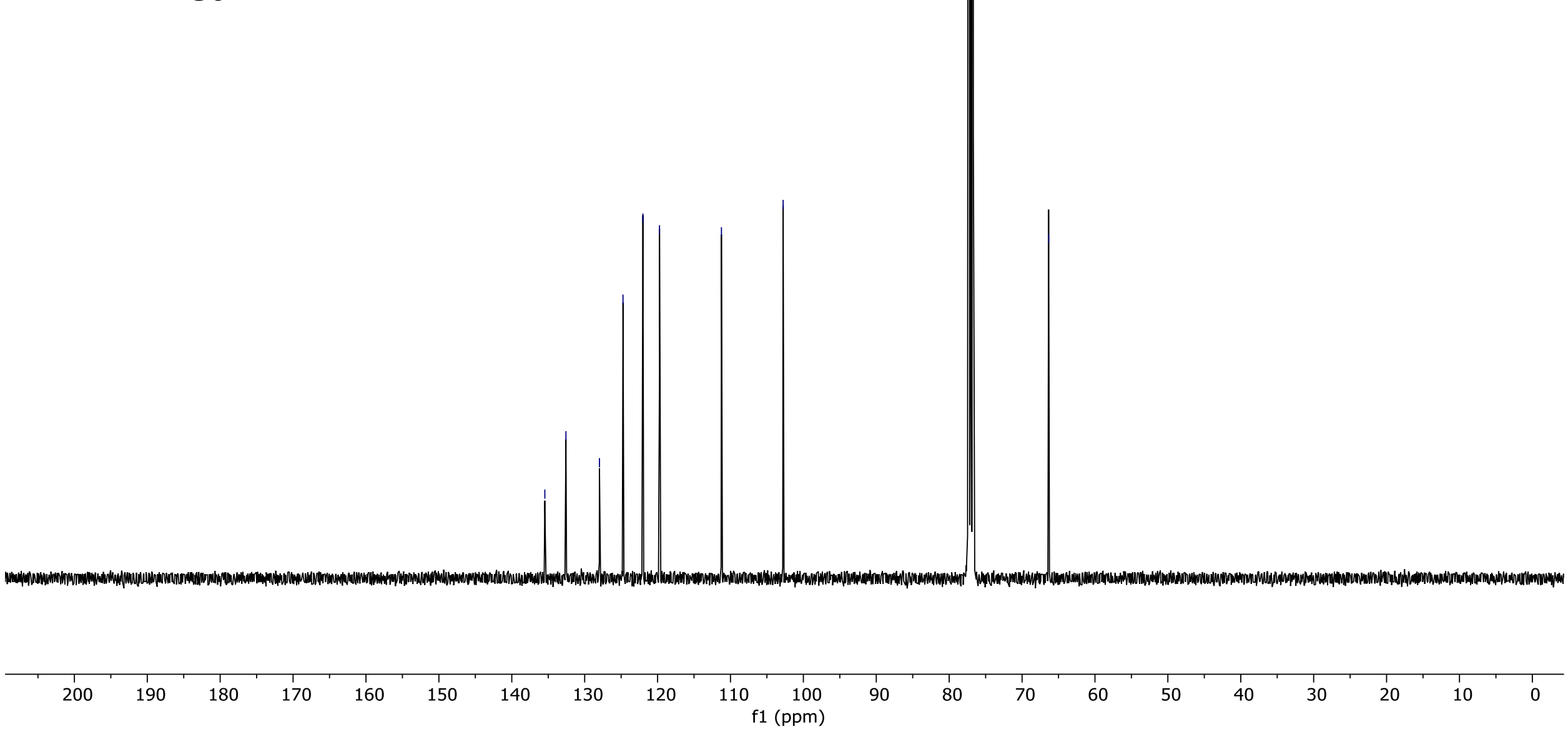

^{1}H NMR (400 MHz, $CDCl_3$) of **S11**

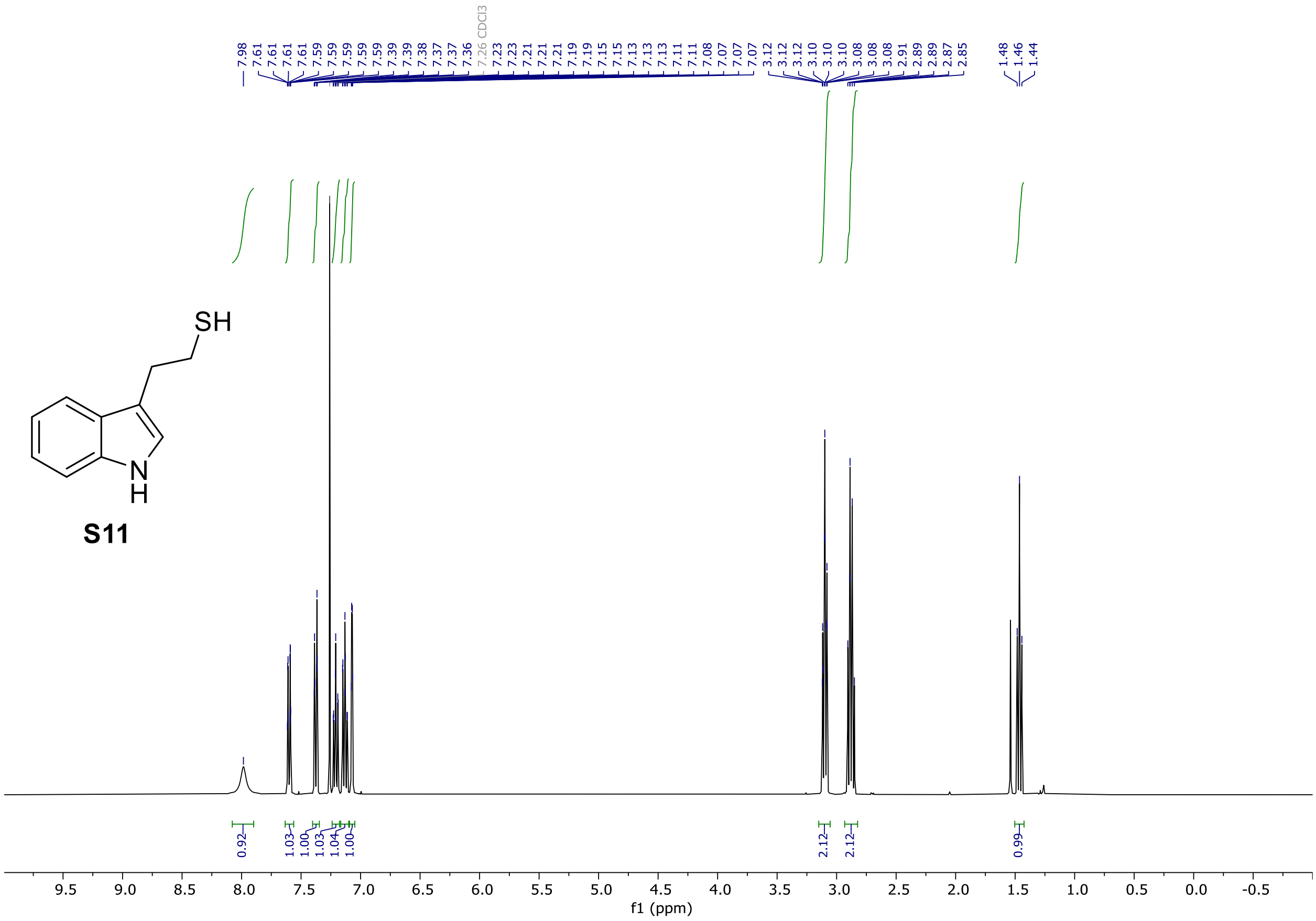

$^{13}C\{^1H\}$ NMR (100 MHz, $CDCl_3$) of **S11**

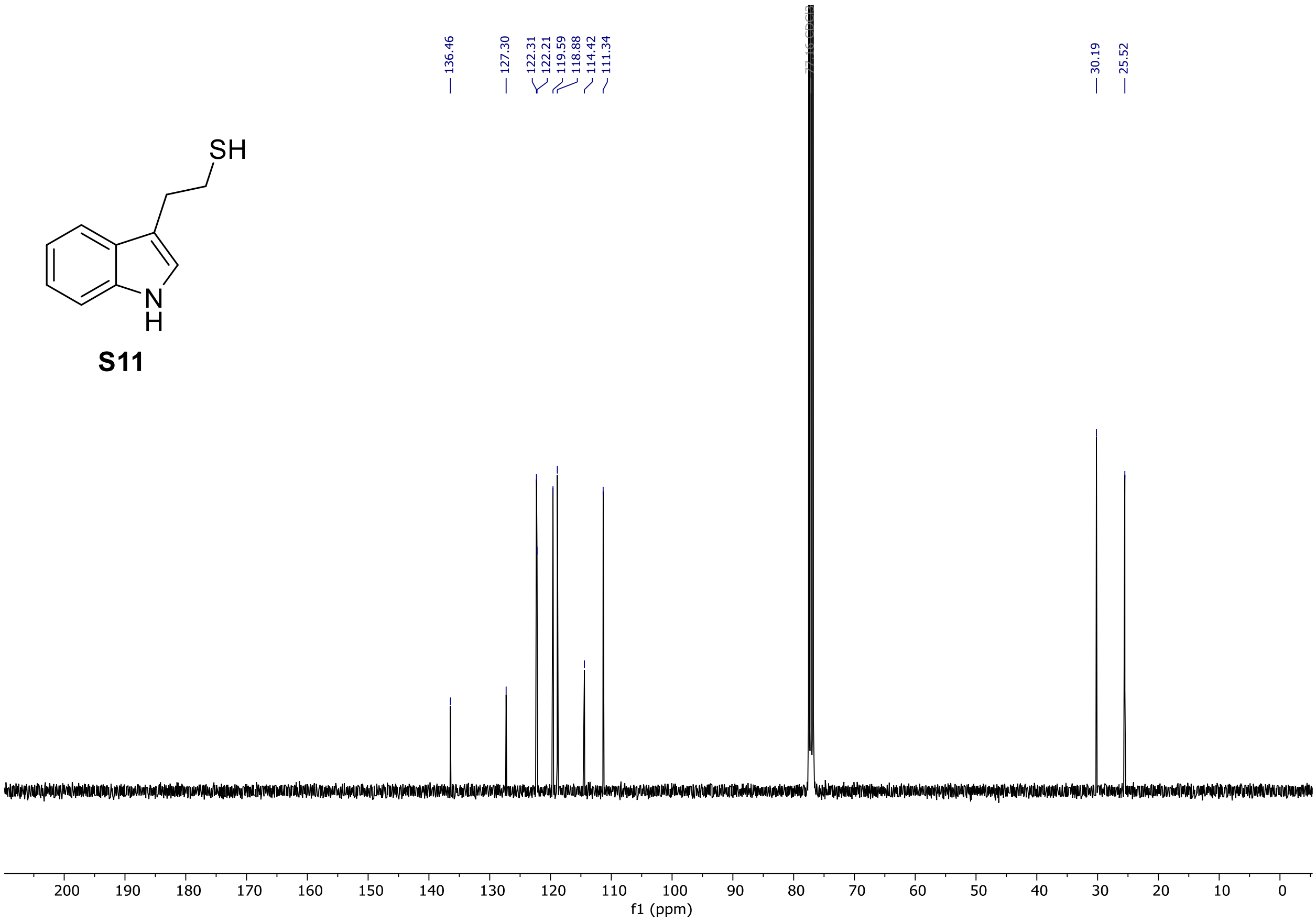

^{1}H NMR (400 MHz, $CDCl_3$) of **S13**

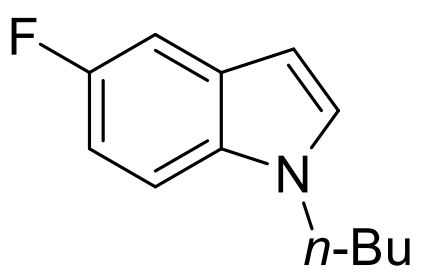

S13

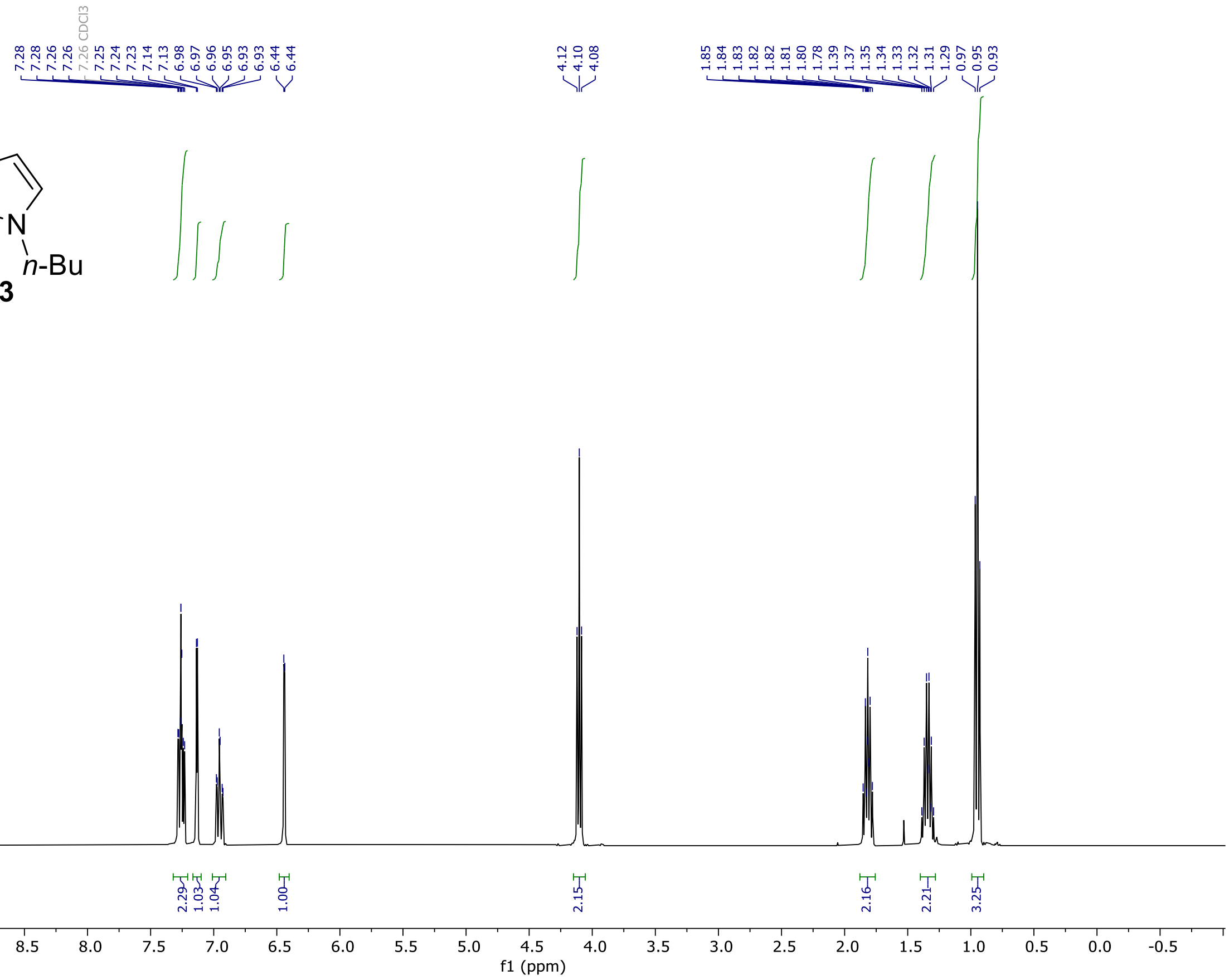

$^{13}C\{^1H\}$ NMR (100 MHz, $CDCl_3$) of **S13**

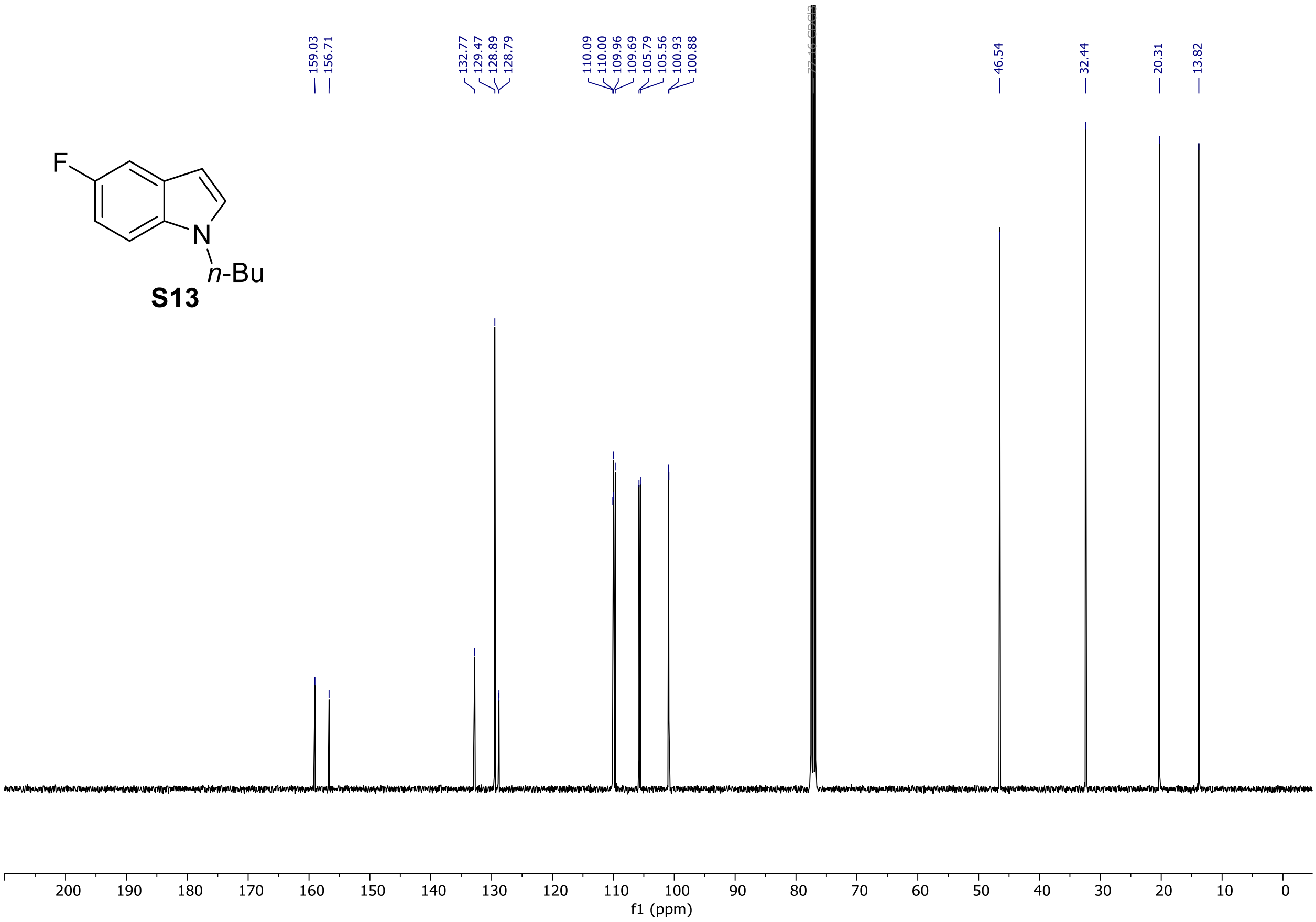

$^{19}F\{^{1}H\}$ NMR (376 MHz, $CDCl_3$) of **S13**

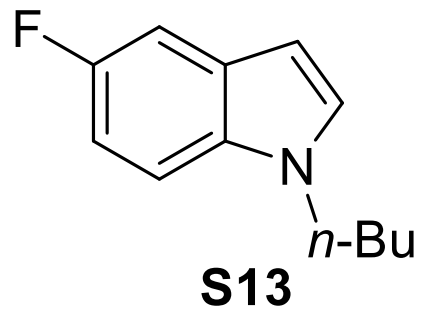

S13

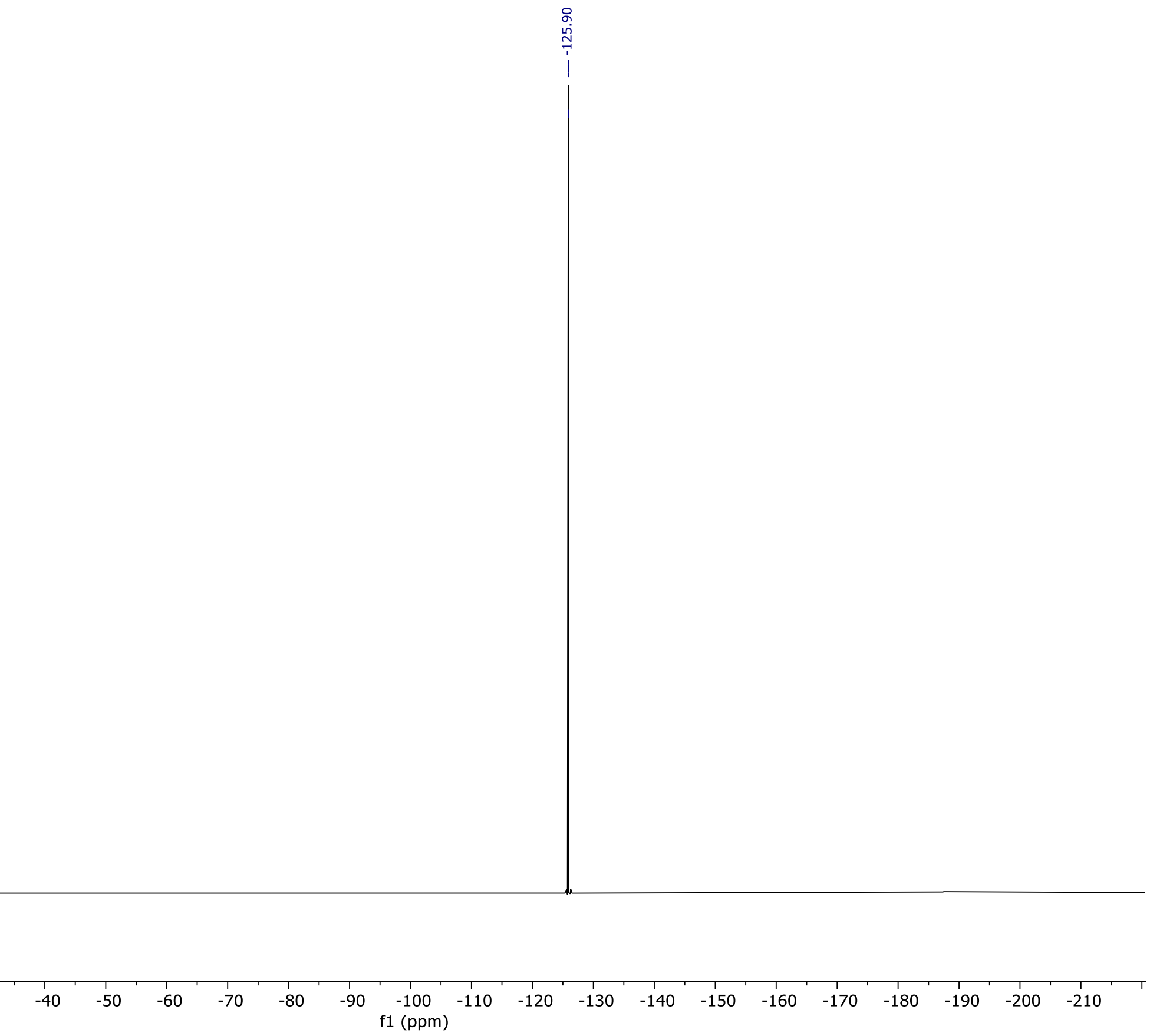

^{1}H NMR (400 MHz, $CDCl_3$) of **S14**

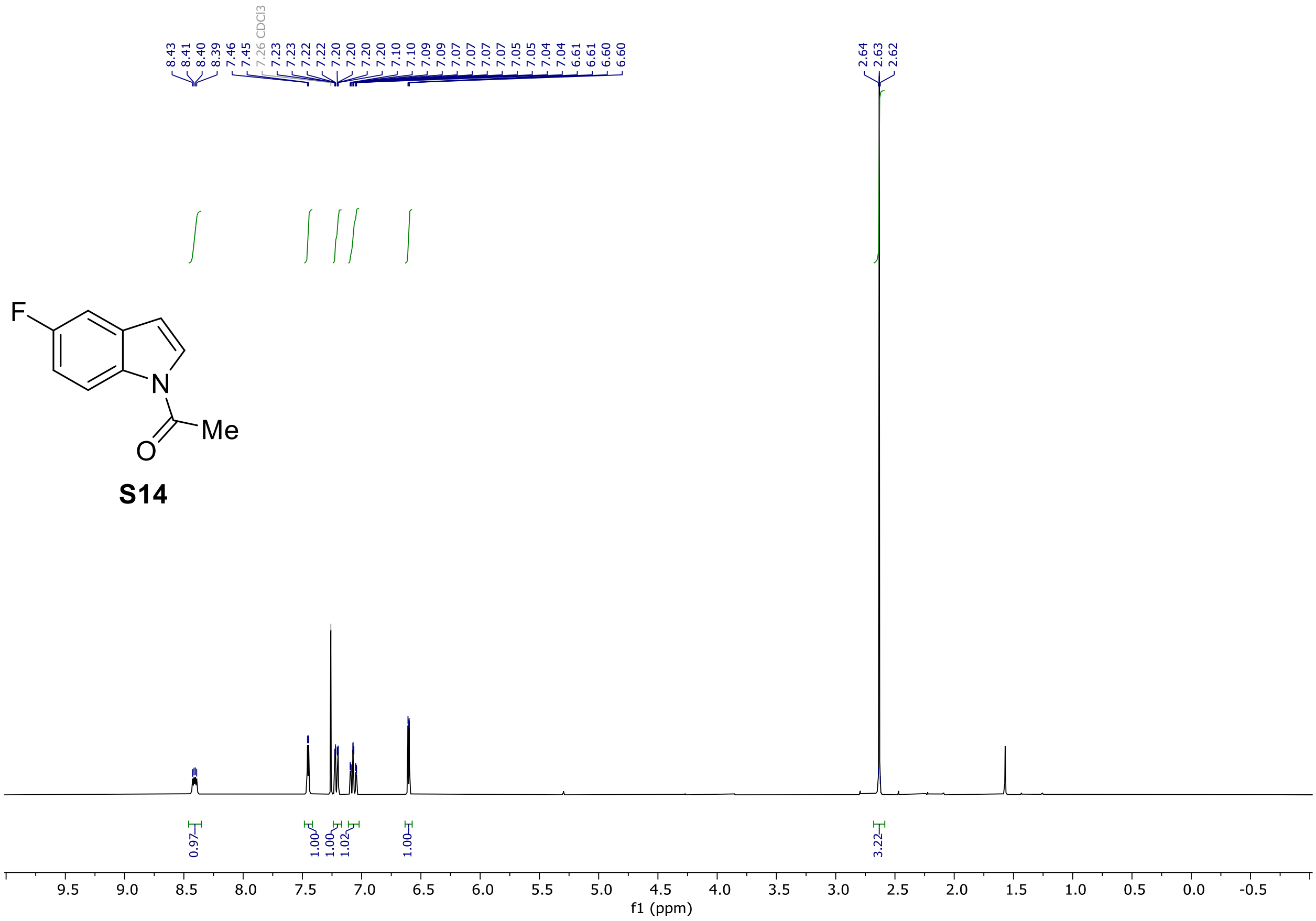

S14

$^{13}C\{^1H\}$ NMR (100 MHz, $CDCl_3$) of **S14**

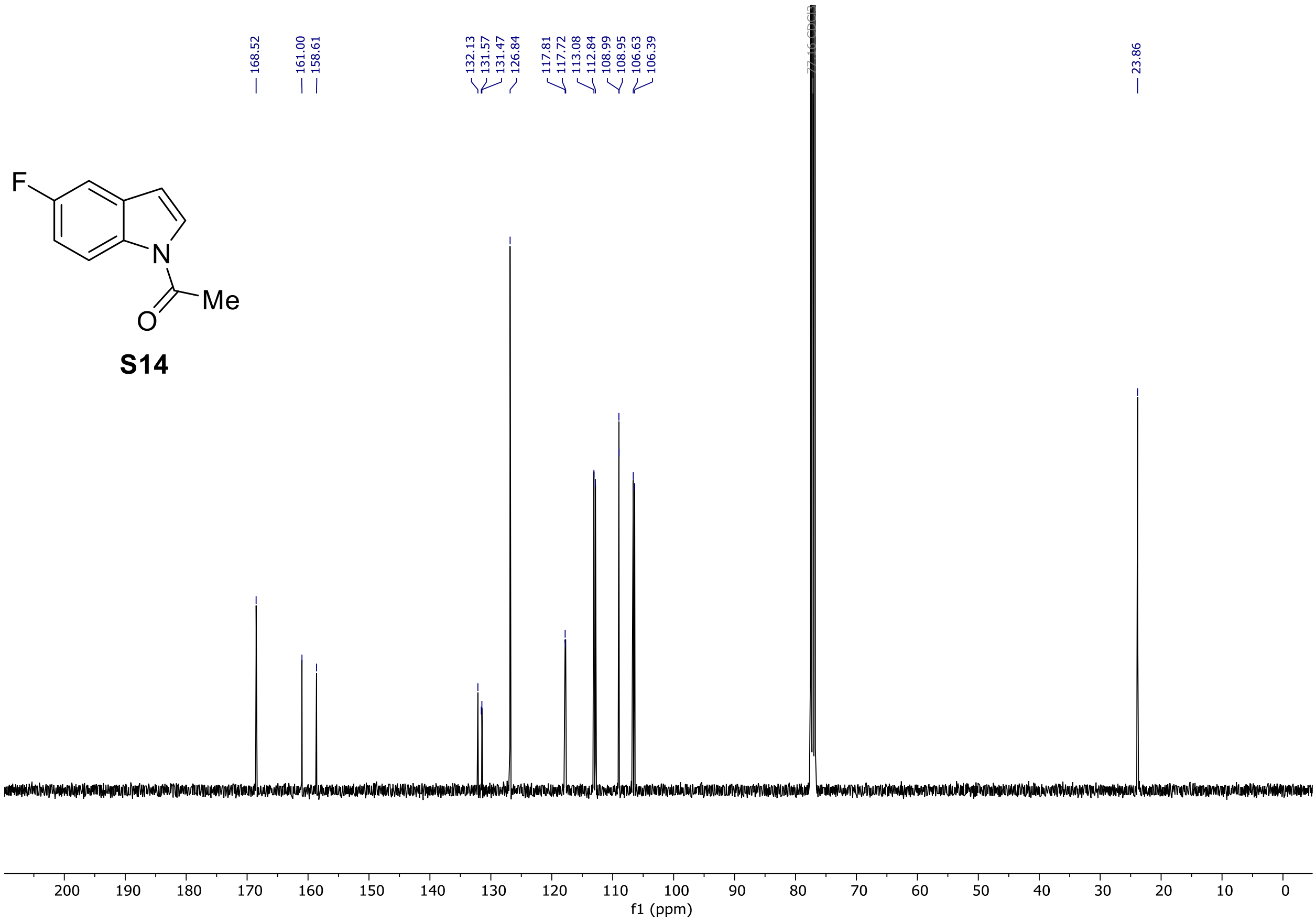

$^{19}F\{^1H\}$ NMR (376 MHz, $CDCl_3$) of **S14**

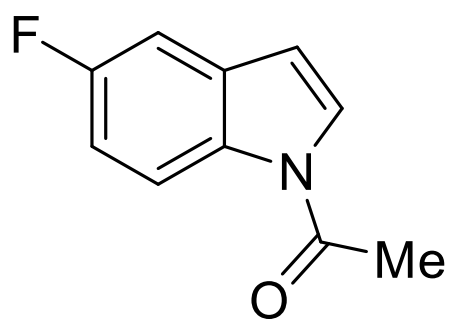

S14

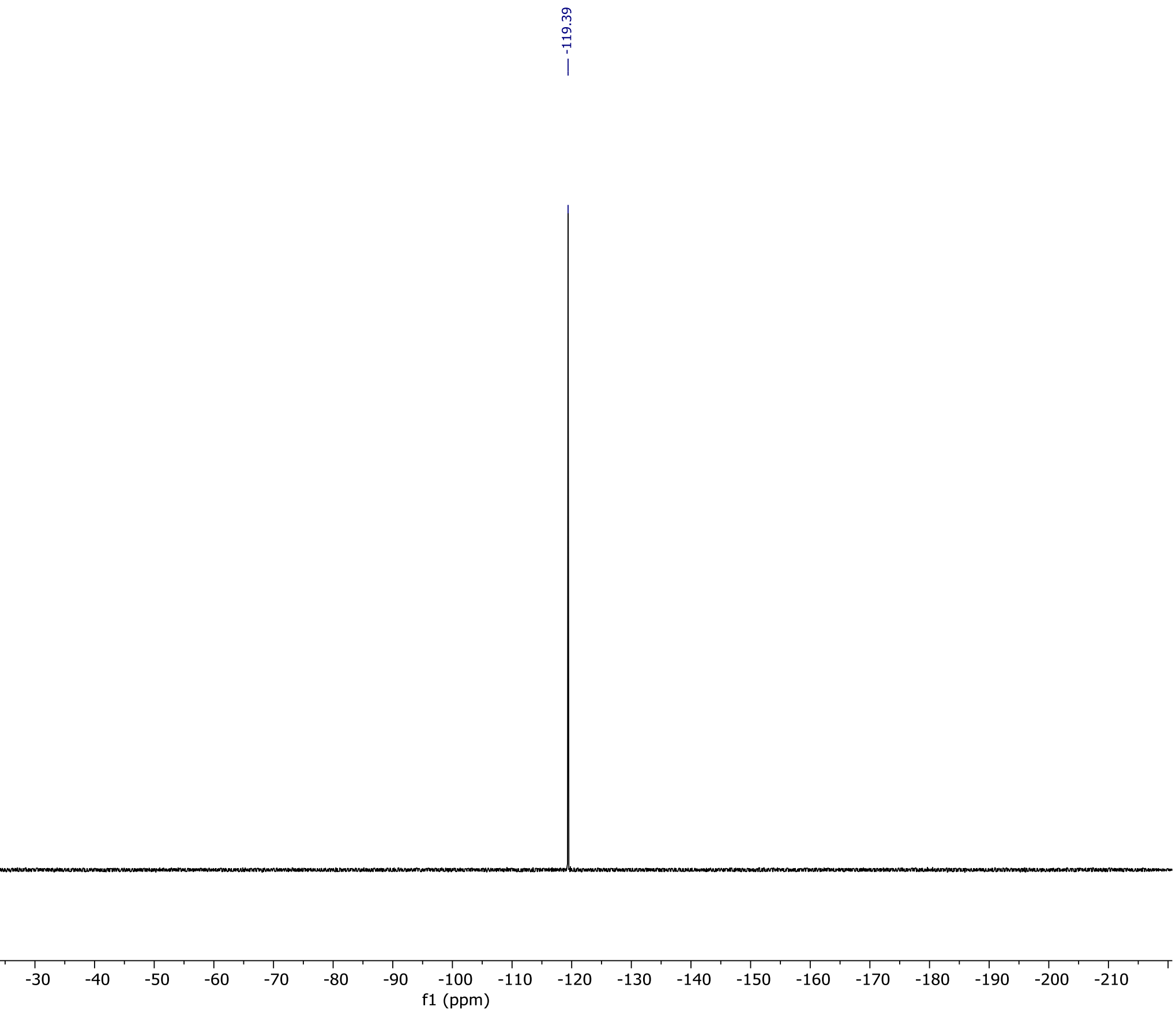

^1H NMR (600 MHz, DMSO) of **S16**

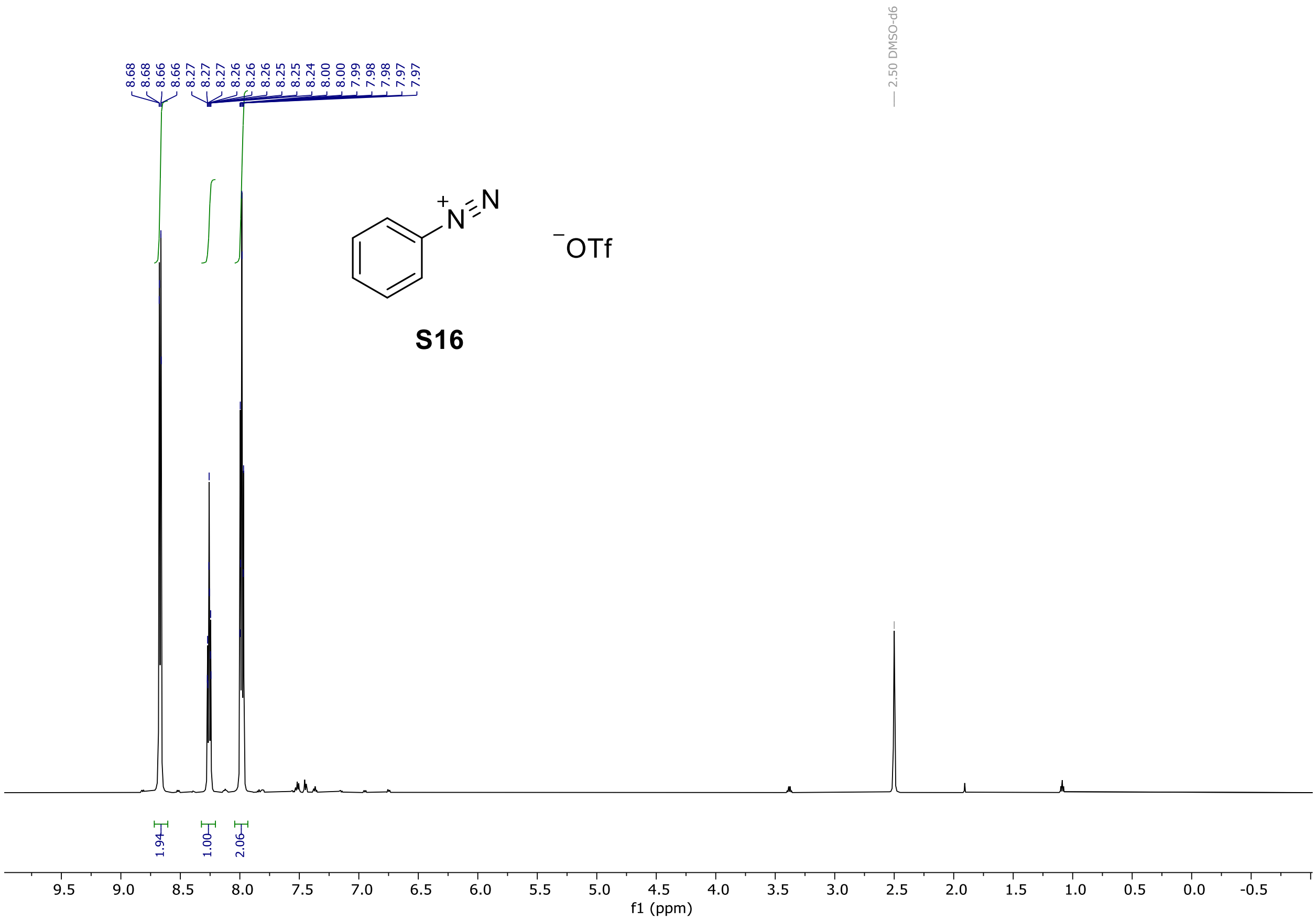

^{13}C NMR (151 MHz, DMSO) of **S16**

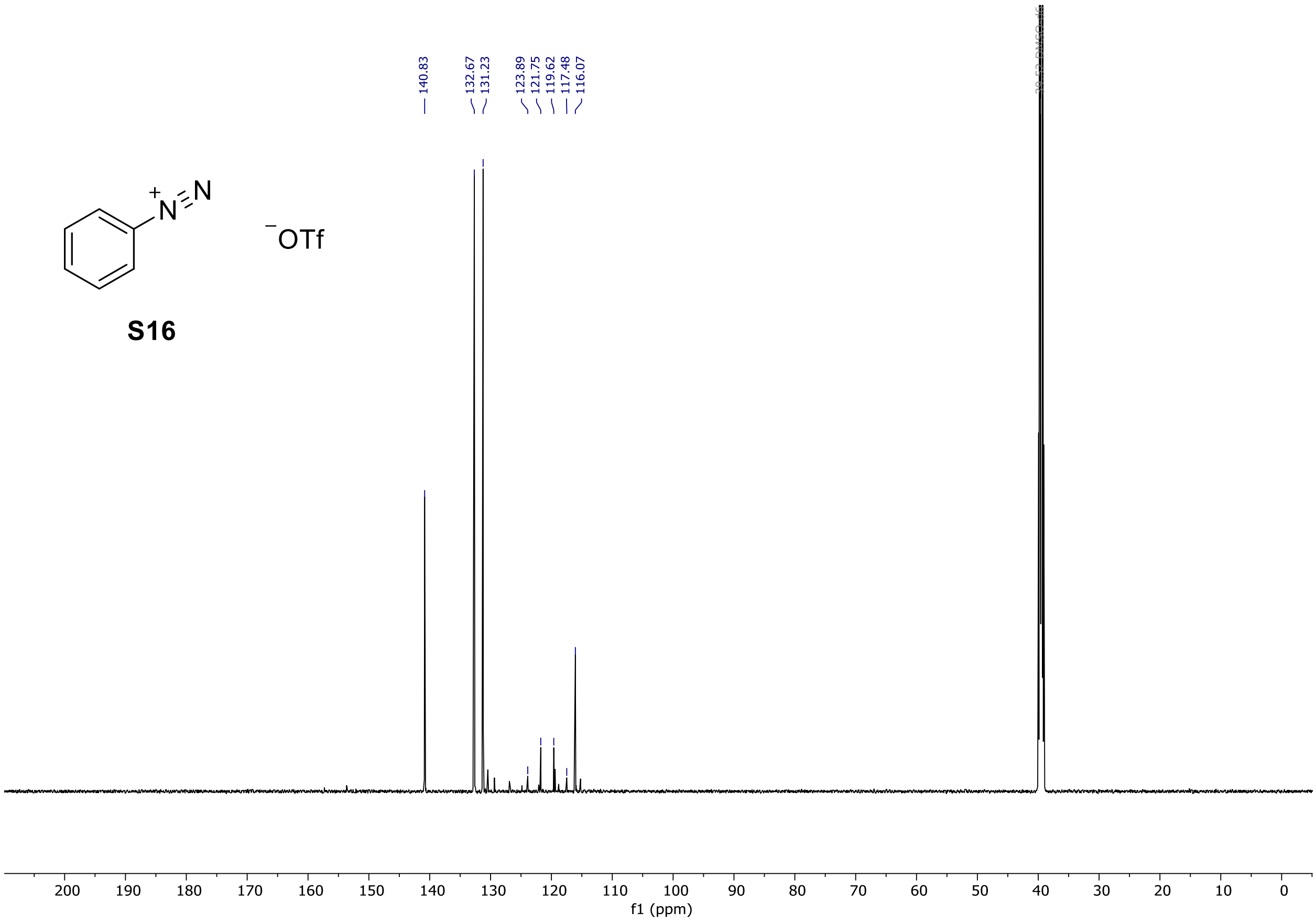

S16

^1H NMR (400 MHz, $CDCl_3$) of **S18**

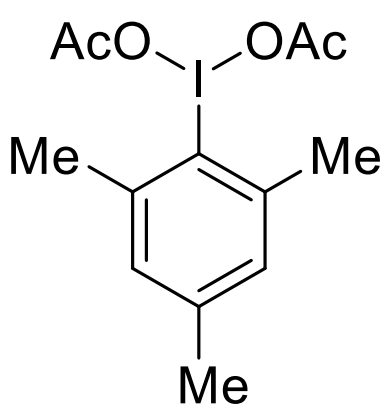

S18

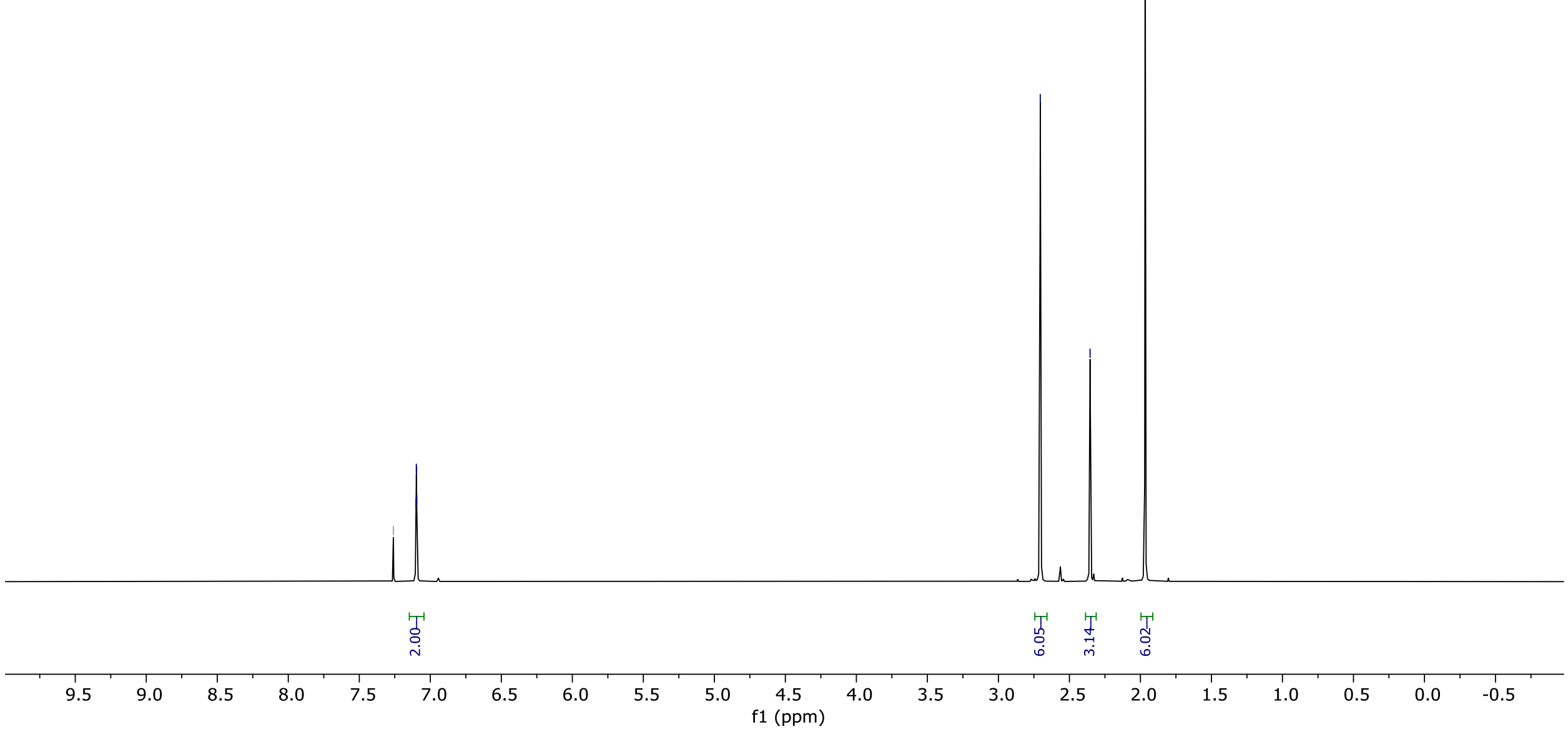

$^{13}C\{^{1}H\}$ NMR (100 MHz, $CDCl_3$) of **S18**

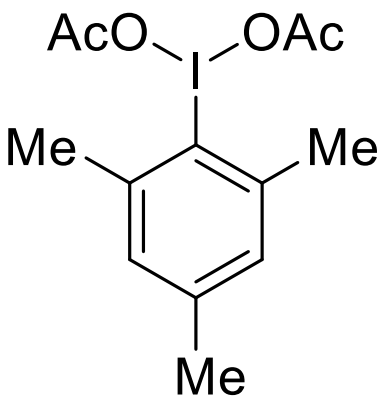

S18

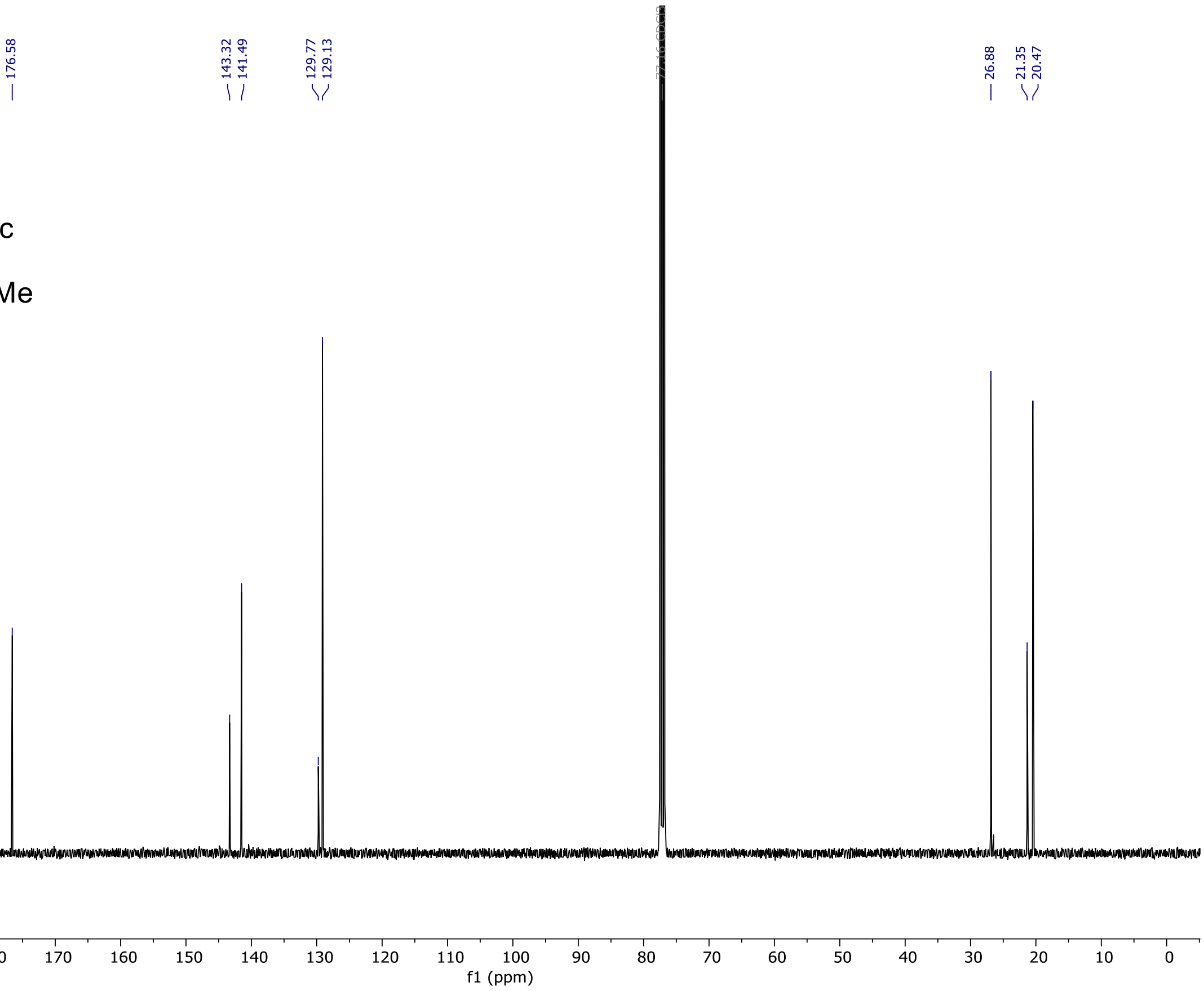

^{1}H NMR (400 MHz, DMSO) of **S19**

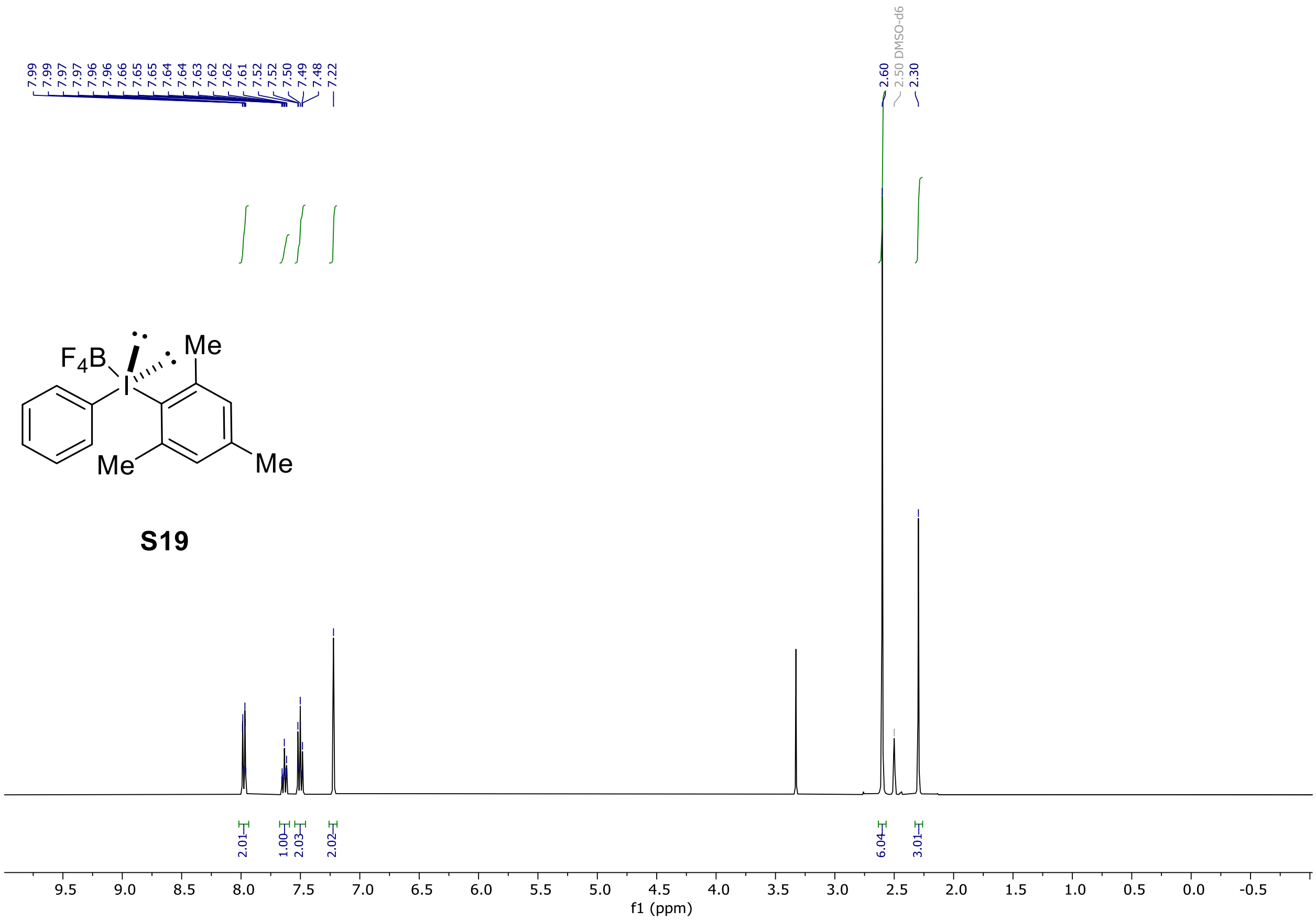

$^{13}C\{^{1}H\}$ NMR (100 MHz, DMSO) of **S19**

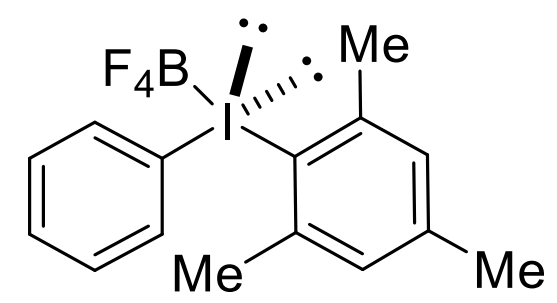

S19

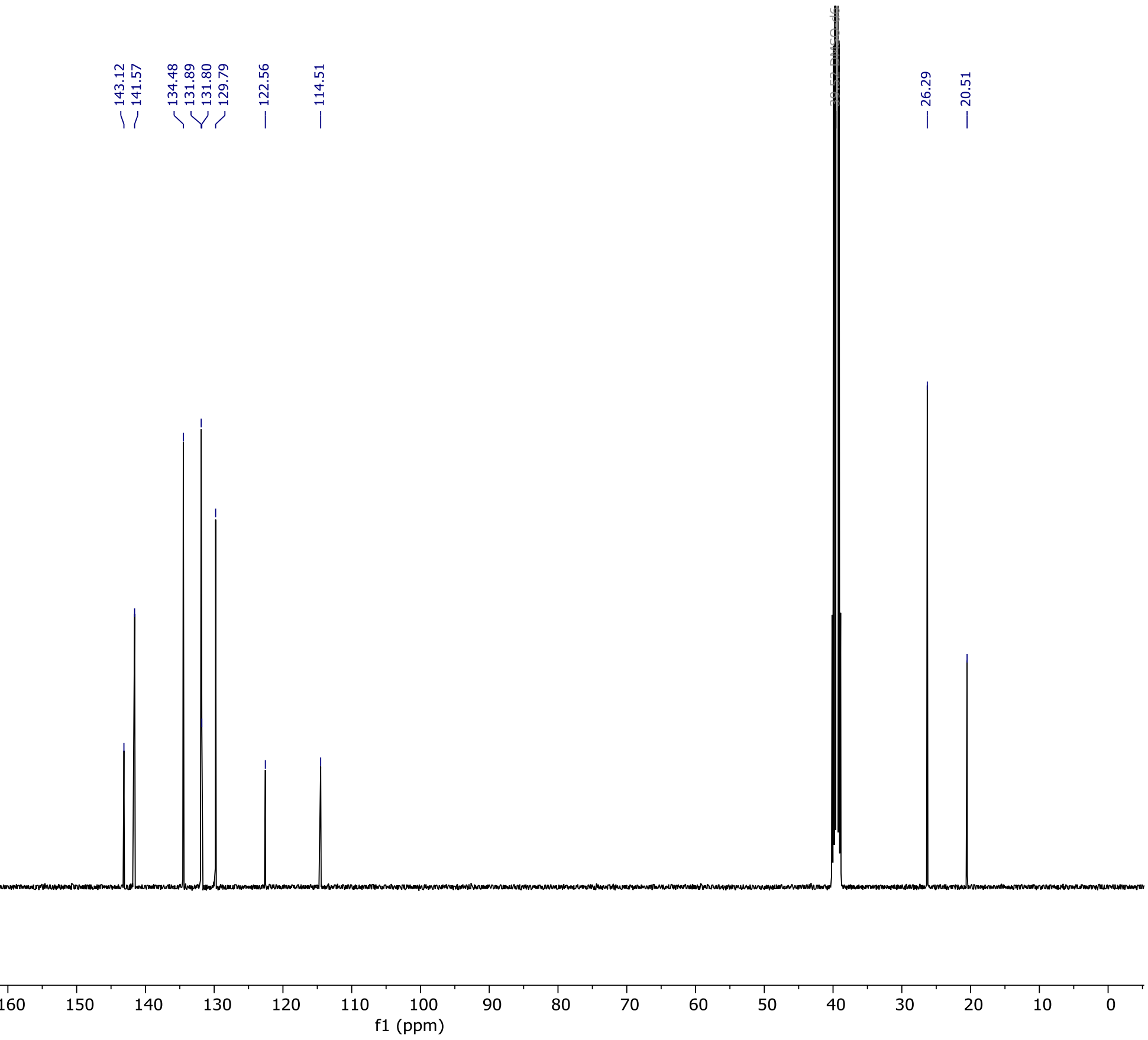

$^{19}F\{^{1}H\}$ NMR (376 MHz, DMSO) of **S19**

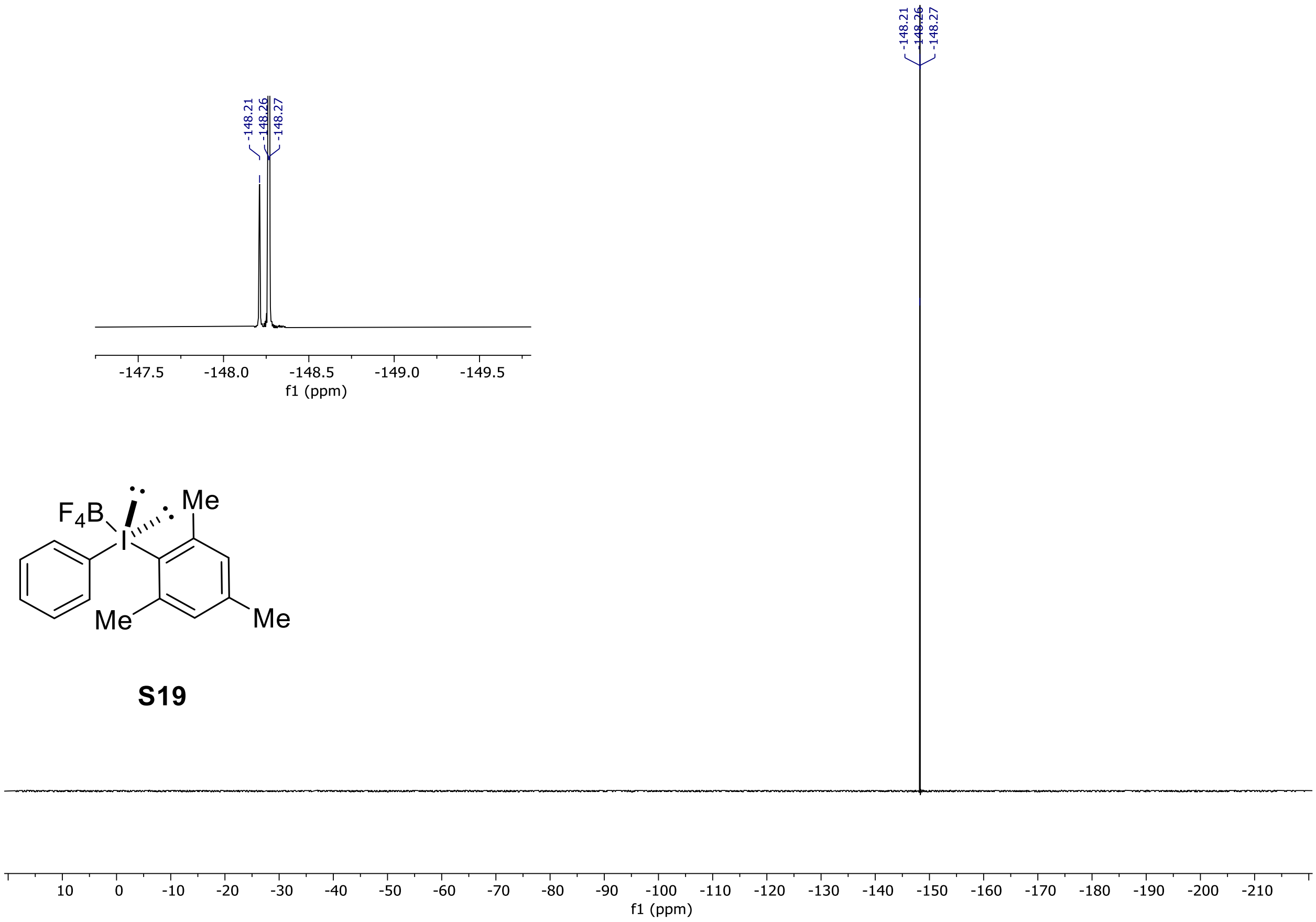

^{1}H NMR (400 MHz, $CDCl_3$) of **S21**

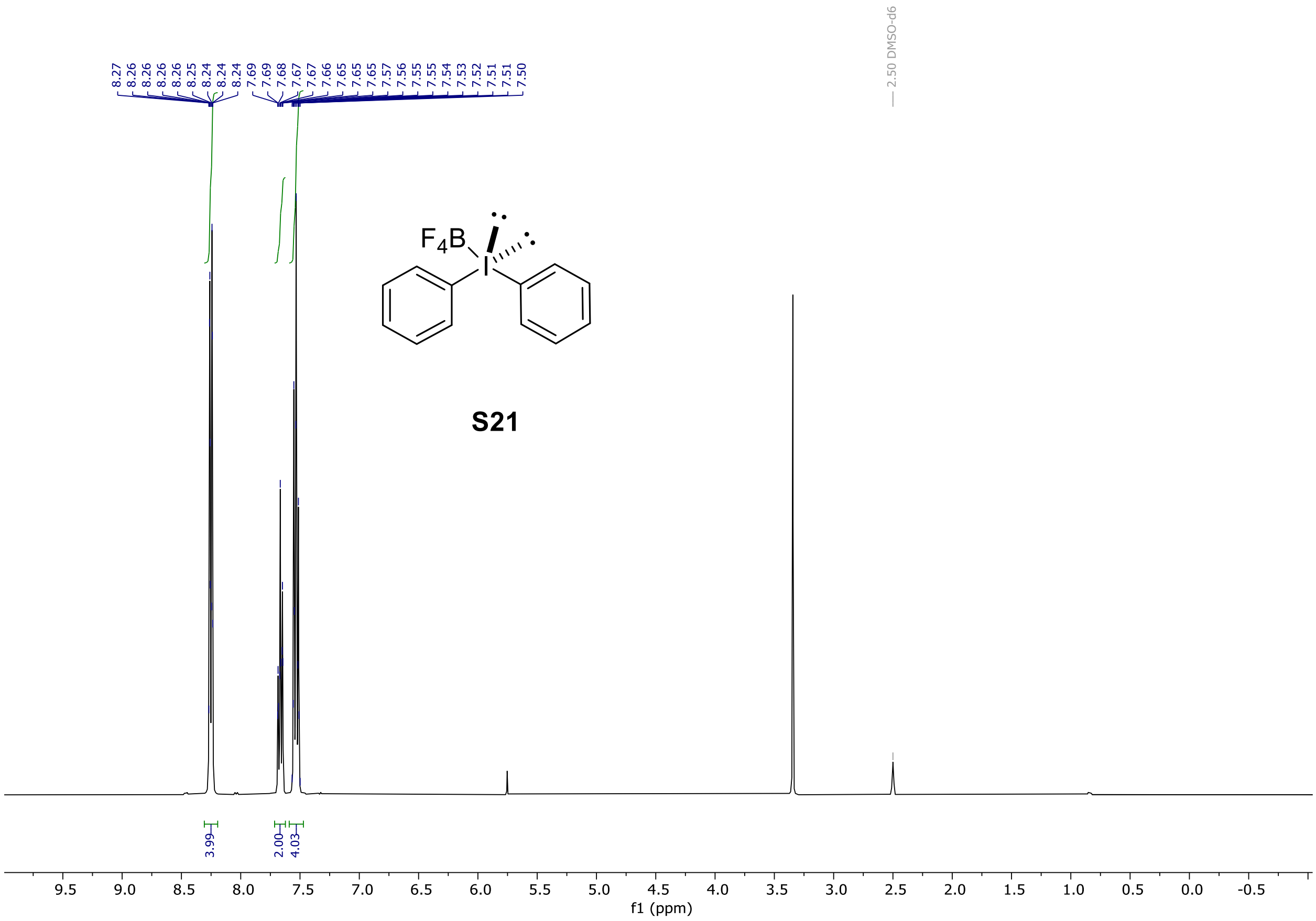

$^{13}C\{^{1}H\}$ NMR (100 MHz, $CDCl_3$) of **S21**

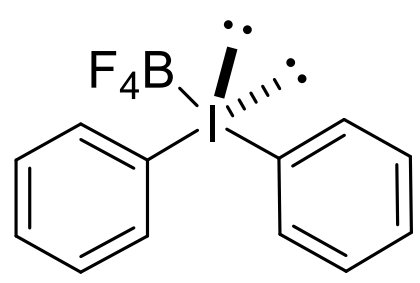

S21

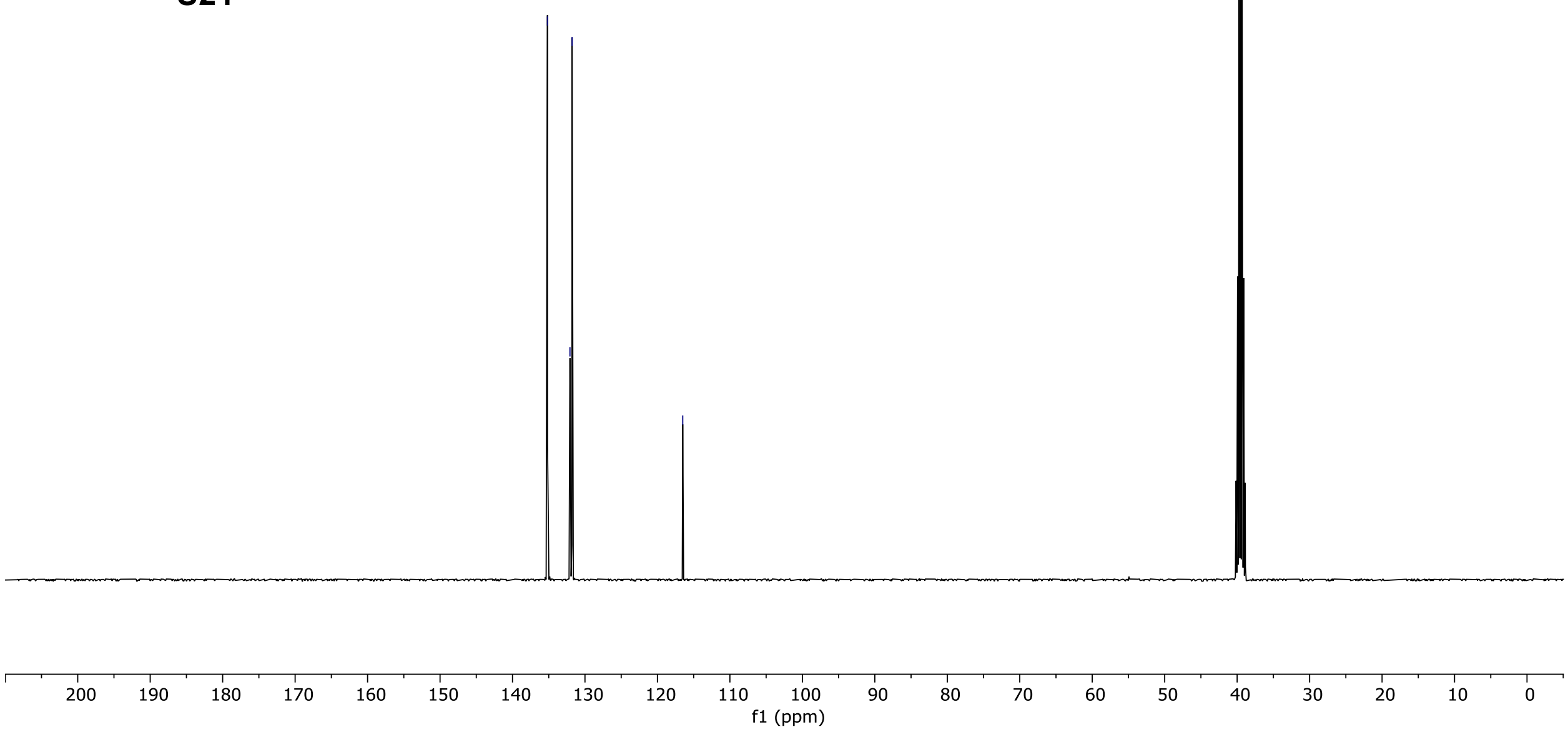

$^{19}F\{^{1}H\}$ NMR (376 MHz, DMSO) of **S21**

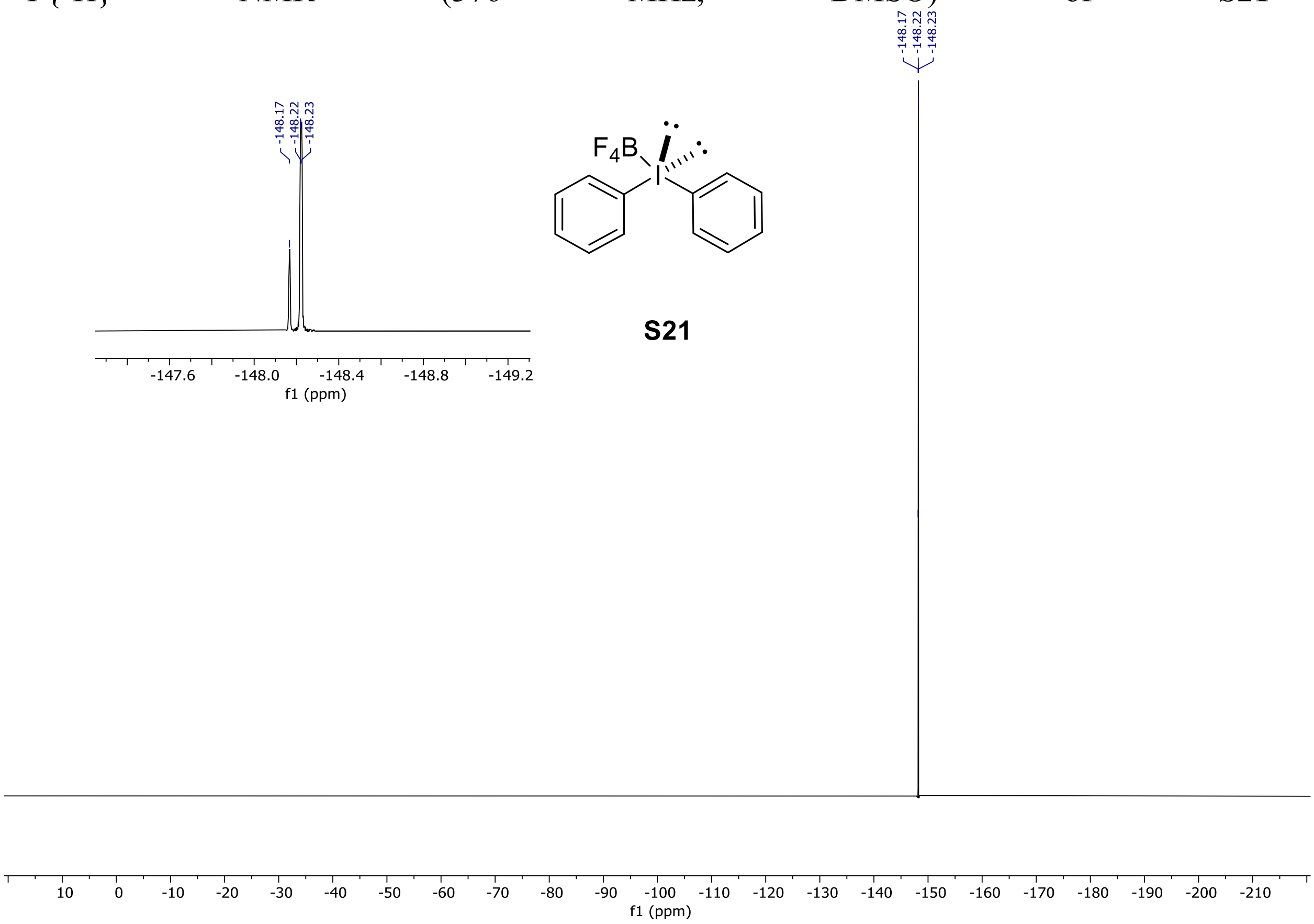

^{1}H NMR (400 MHz, DMSO) of **S22**

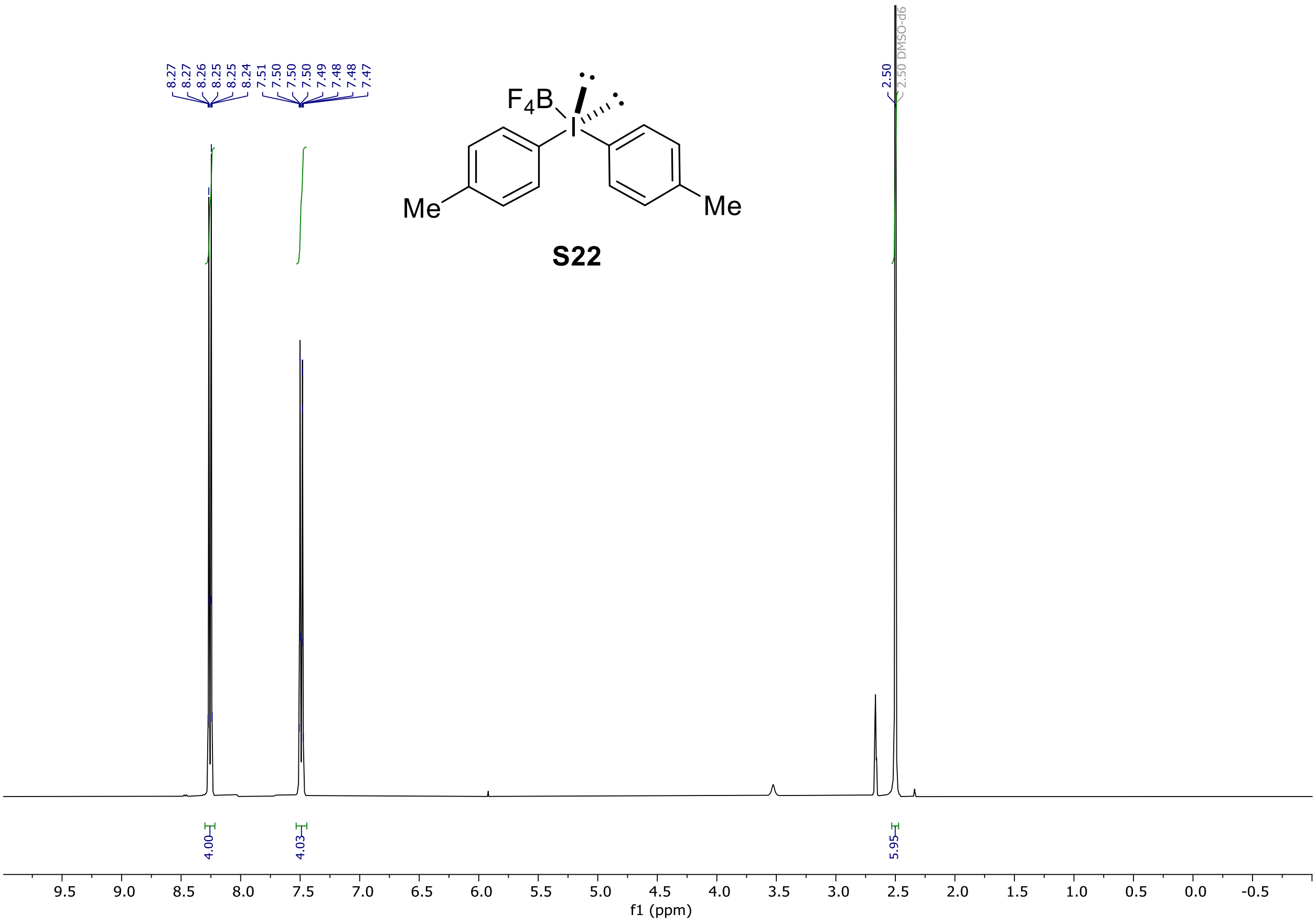

$^{13}C\{^1H\}$ NMR (100 MHz, $CDCl_3$) of **S22**

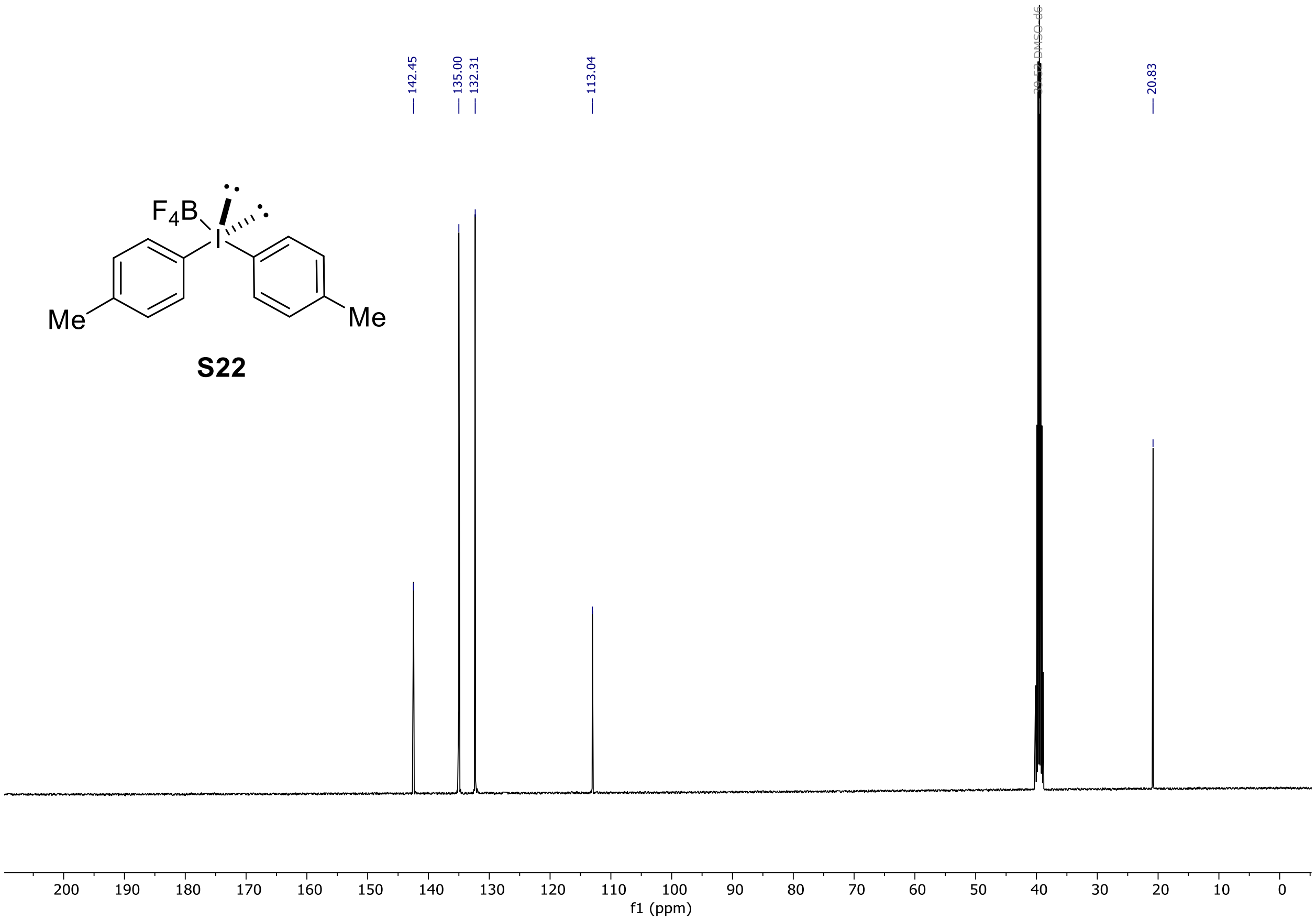

$^{19}F\{^{1}H\}$ NMR (376 MHz, DMSO) of **S22**

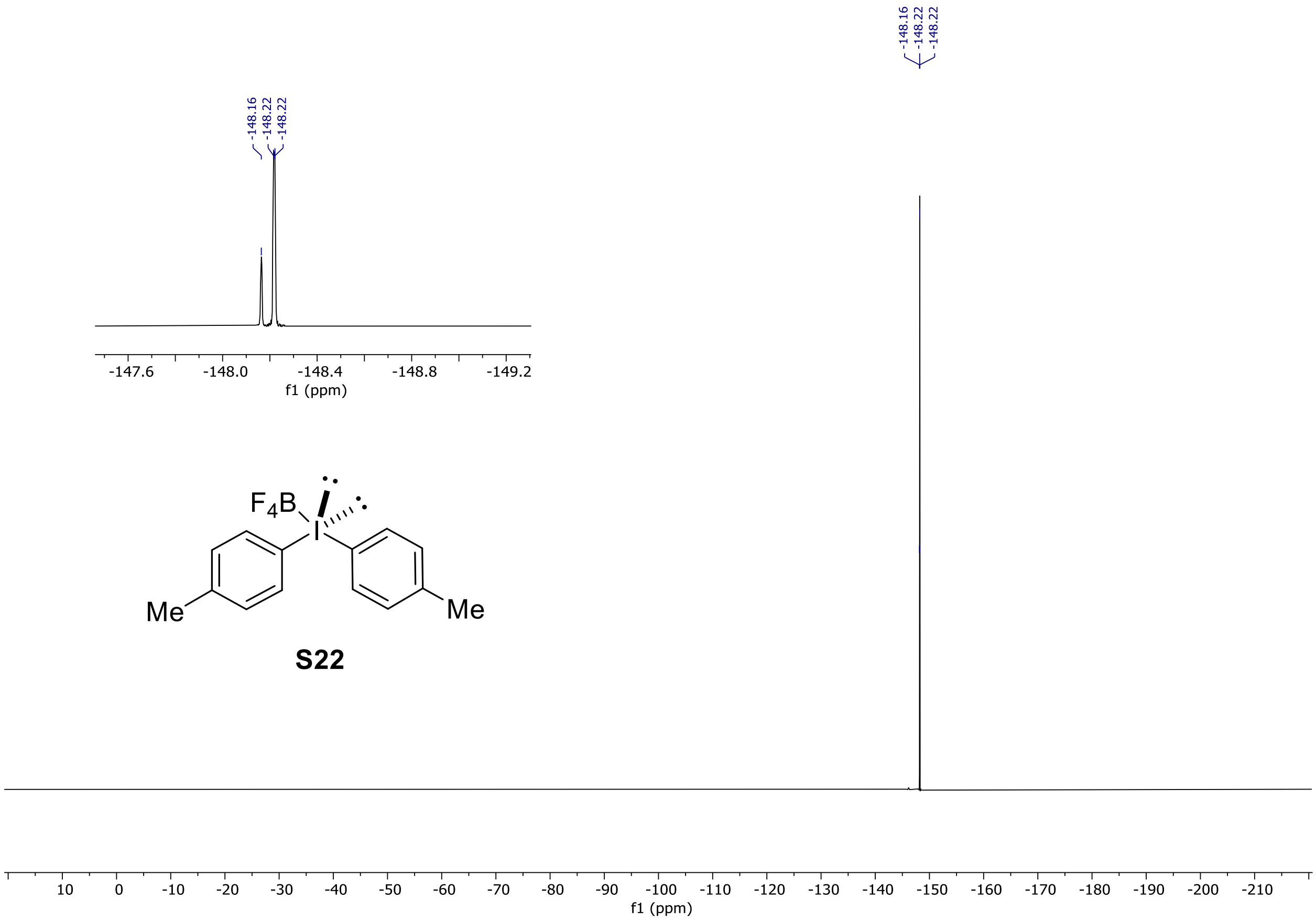

^{1}H NMR (400 MHz, DMSO) of **S23**

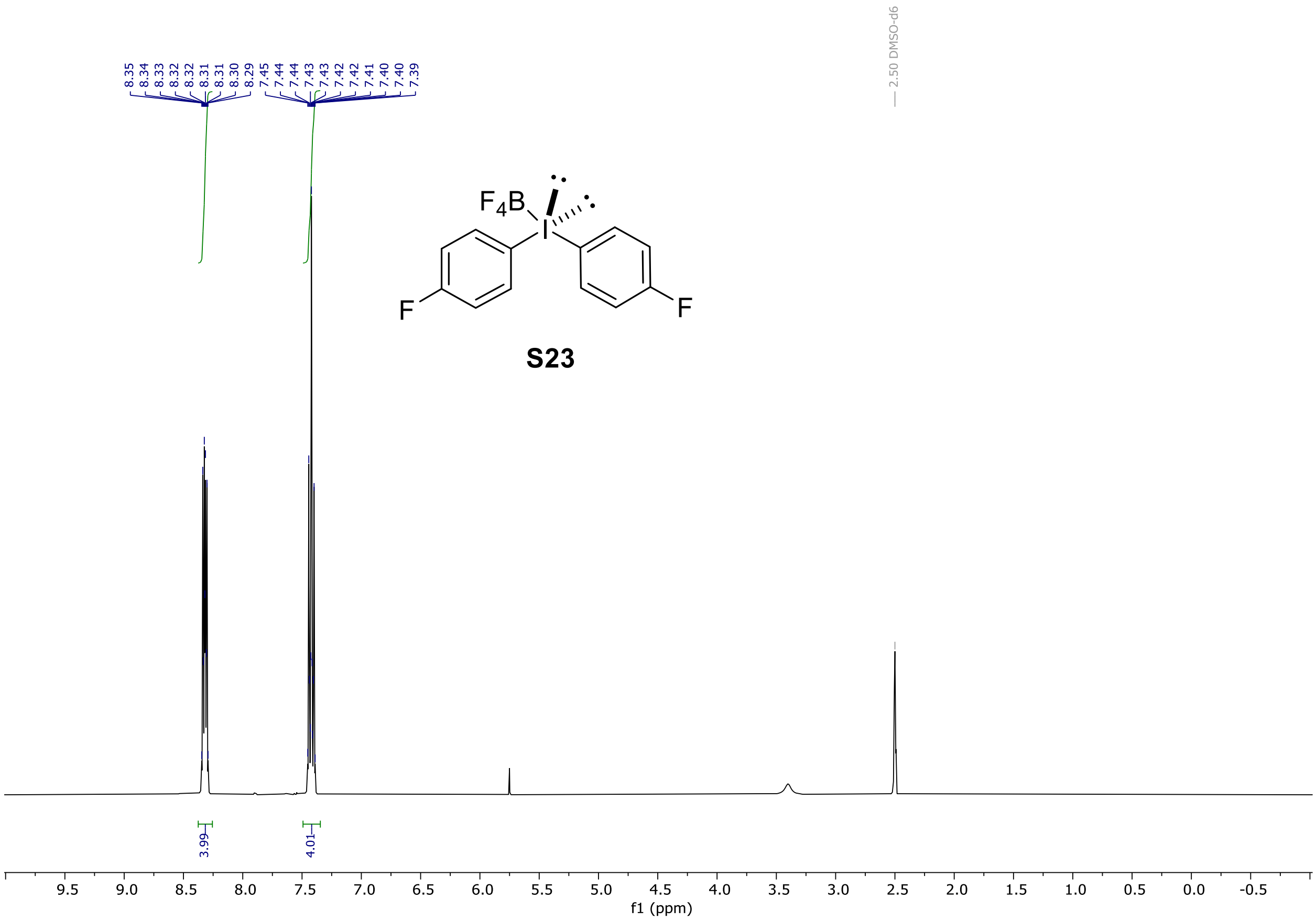

$^{13}C\{^1H\}$ NMR (100 MHz, DMSO) of **S23**

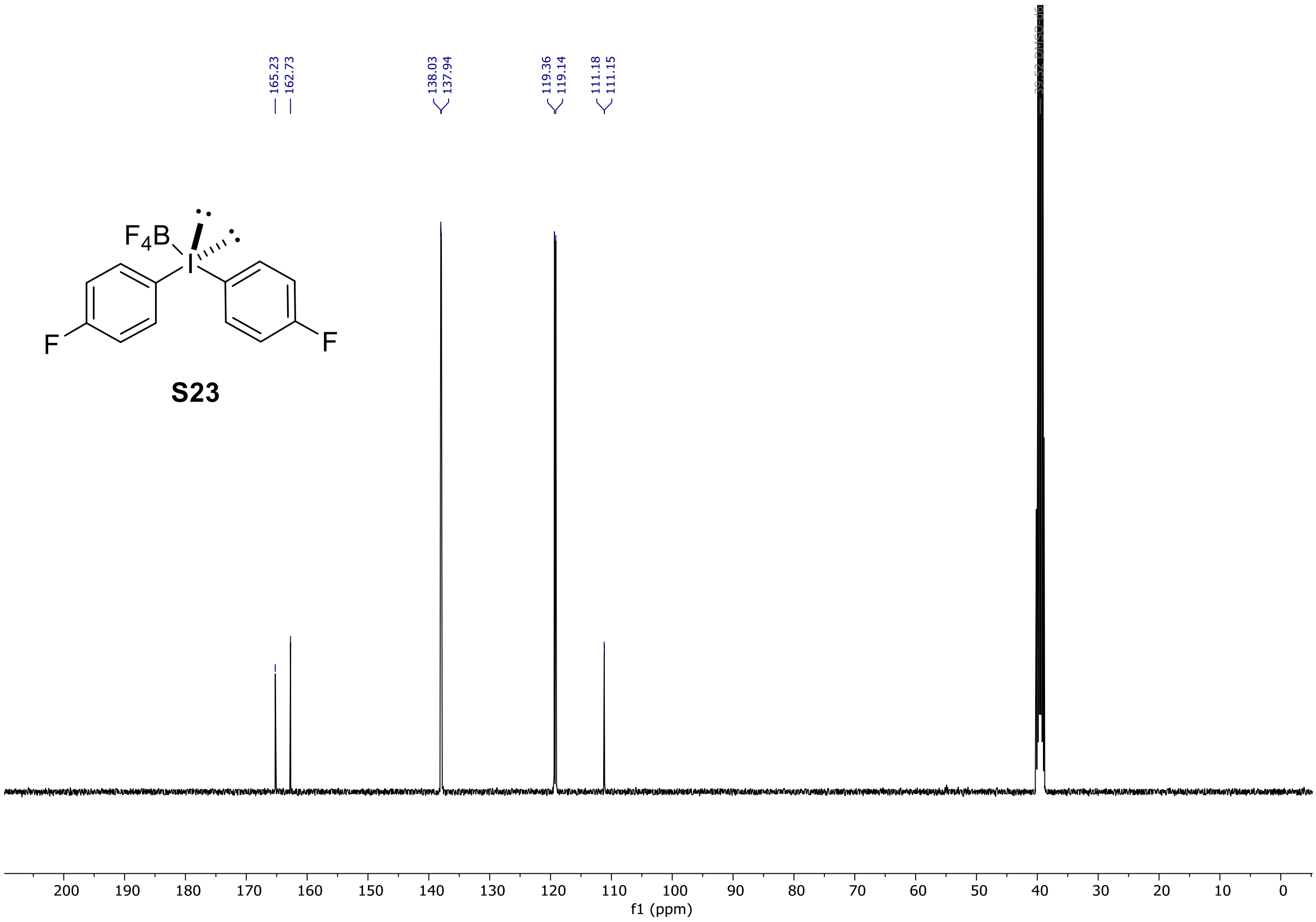

$^{19}F\{^{1}H\}$ NMR (376 MHz, DMSO) of **S23**

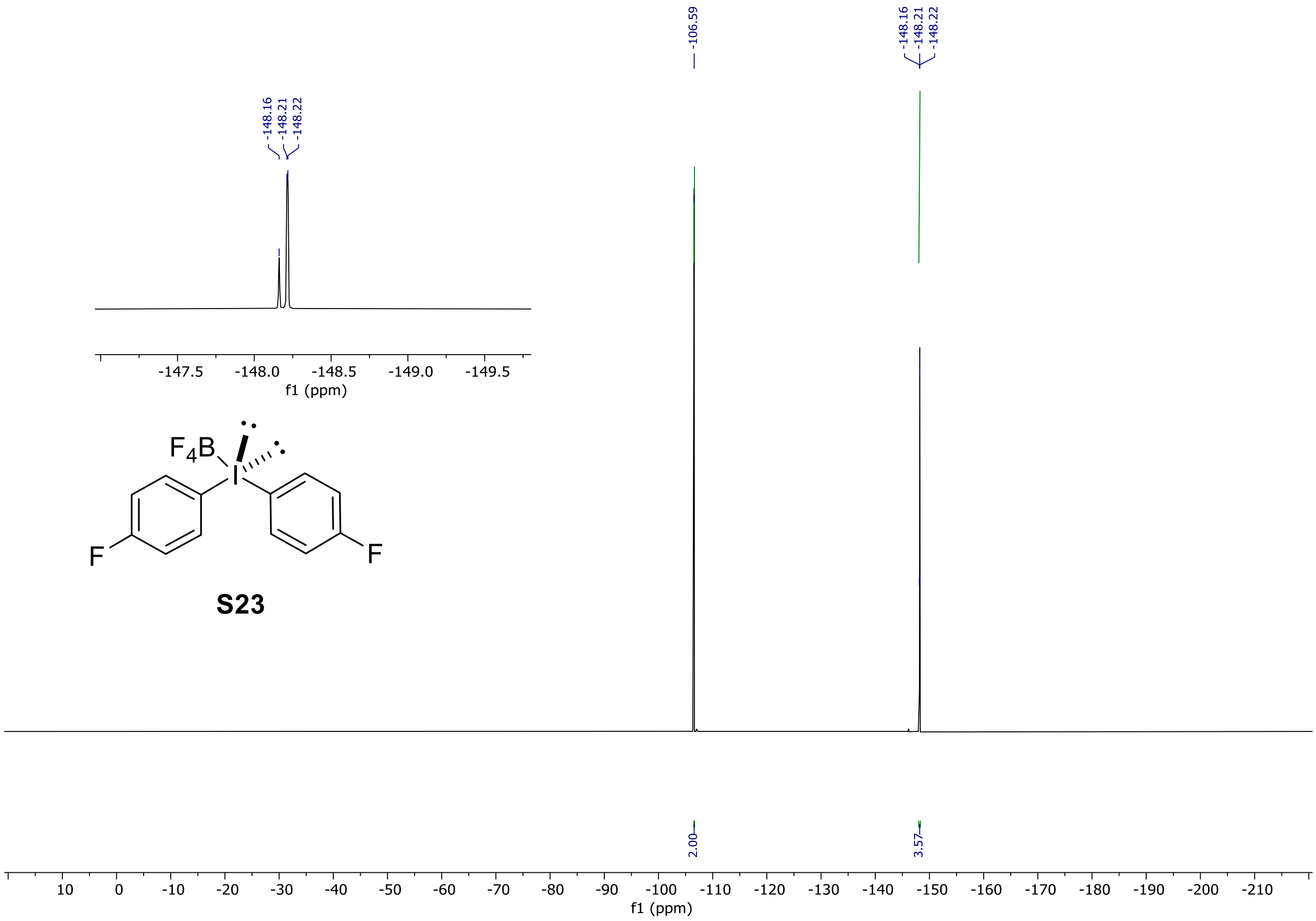

^{1}H NMR (400 MHz, DMSO) of **S24**

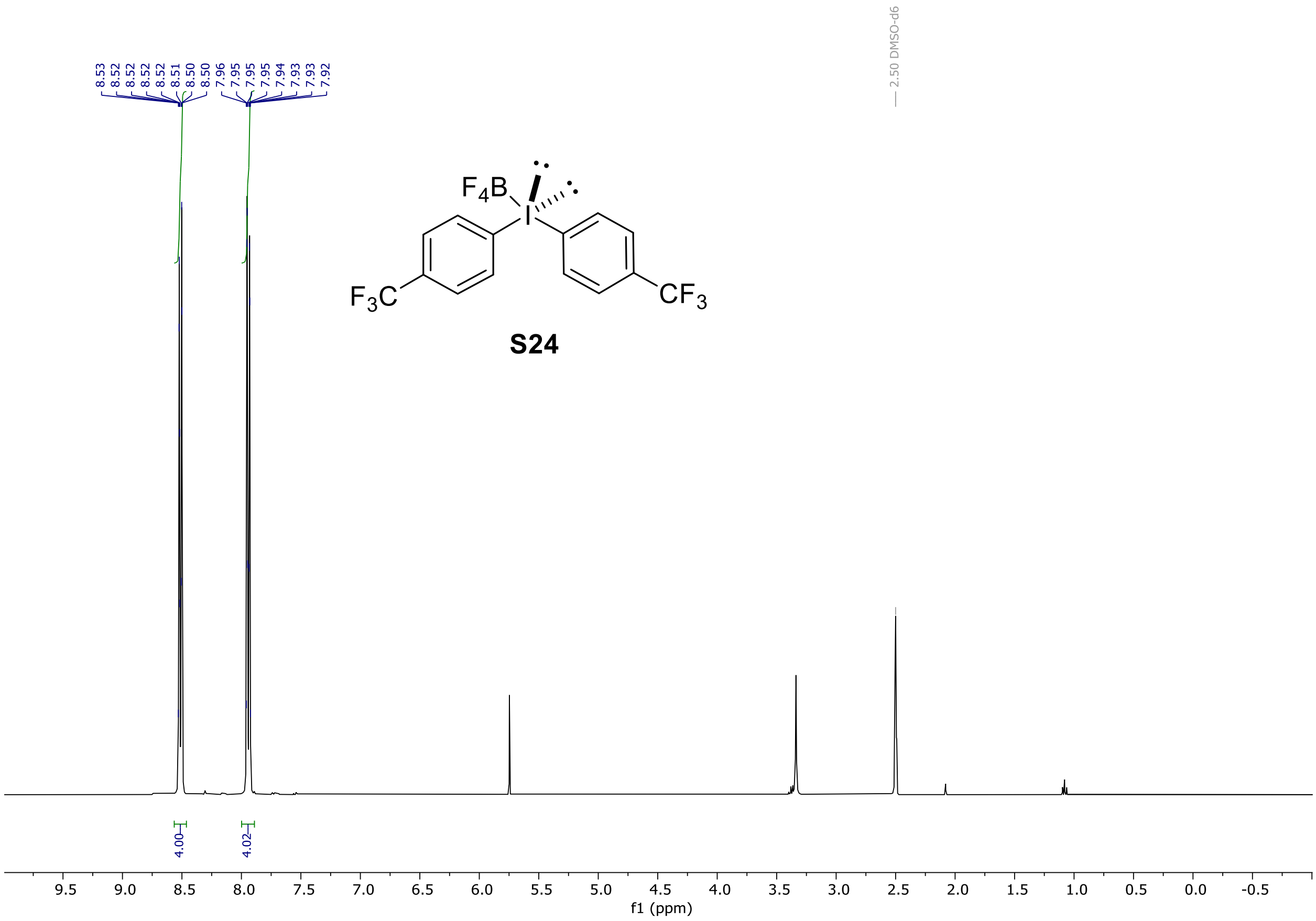

$^{13}C\{^{1}H\}$ NMR (100 MHz, DMSO) of **S24**

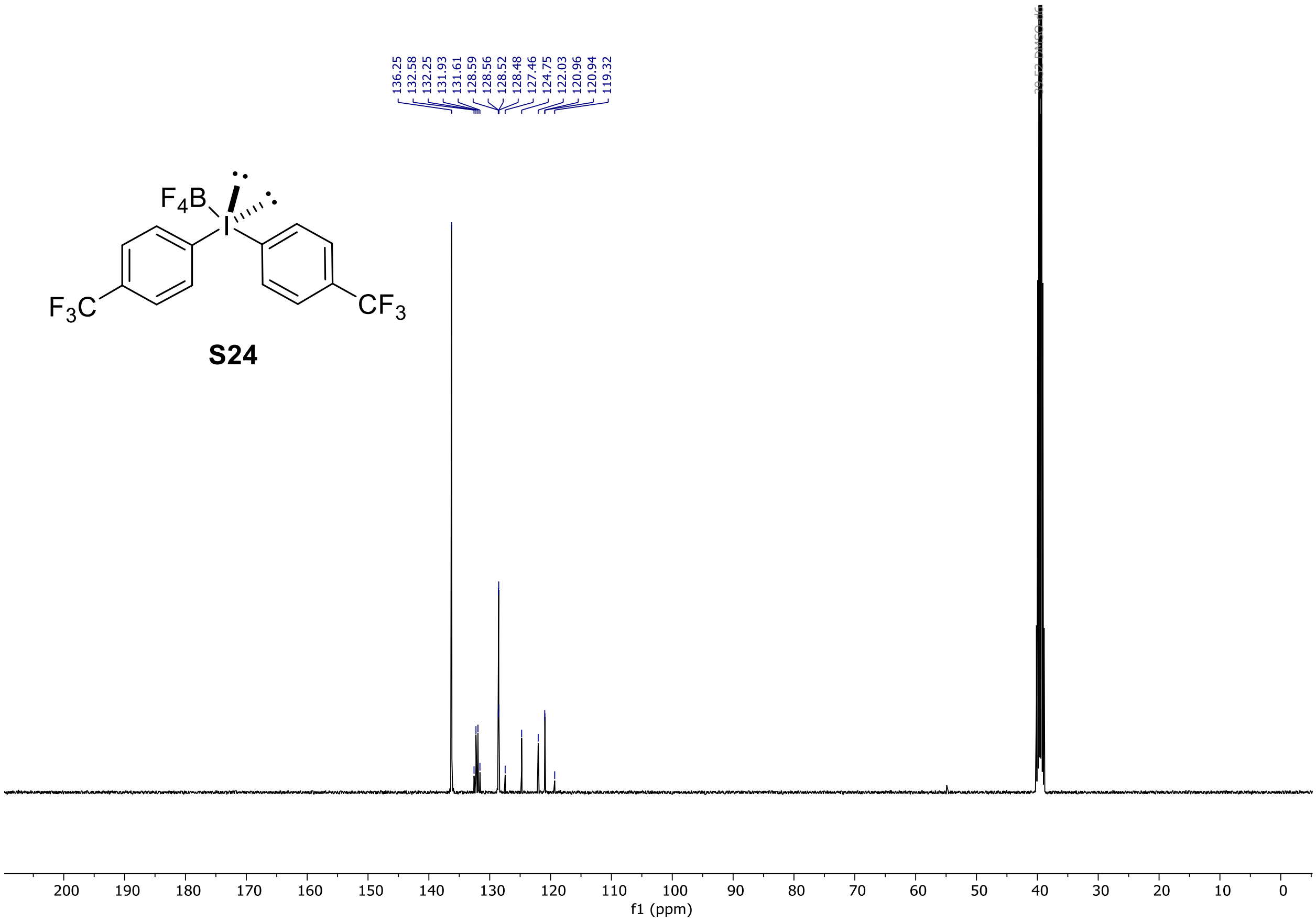

$^{19}F\{^1H\}$ NMR (376 MHz, DMSO) of **S24**

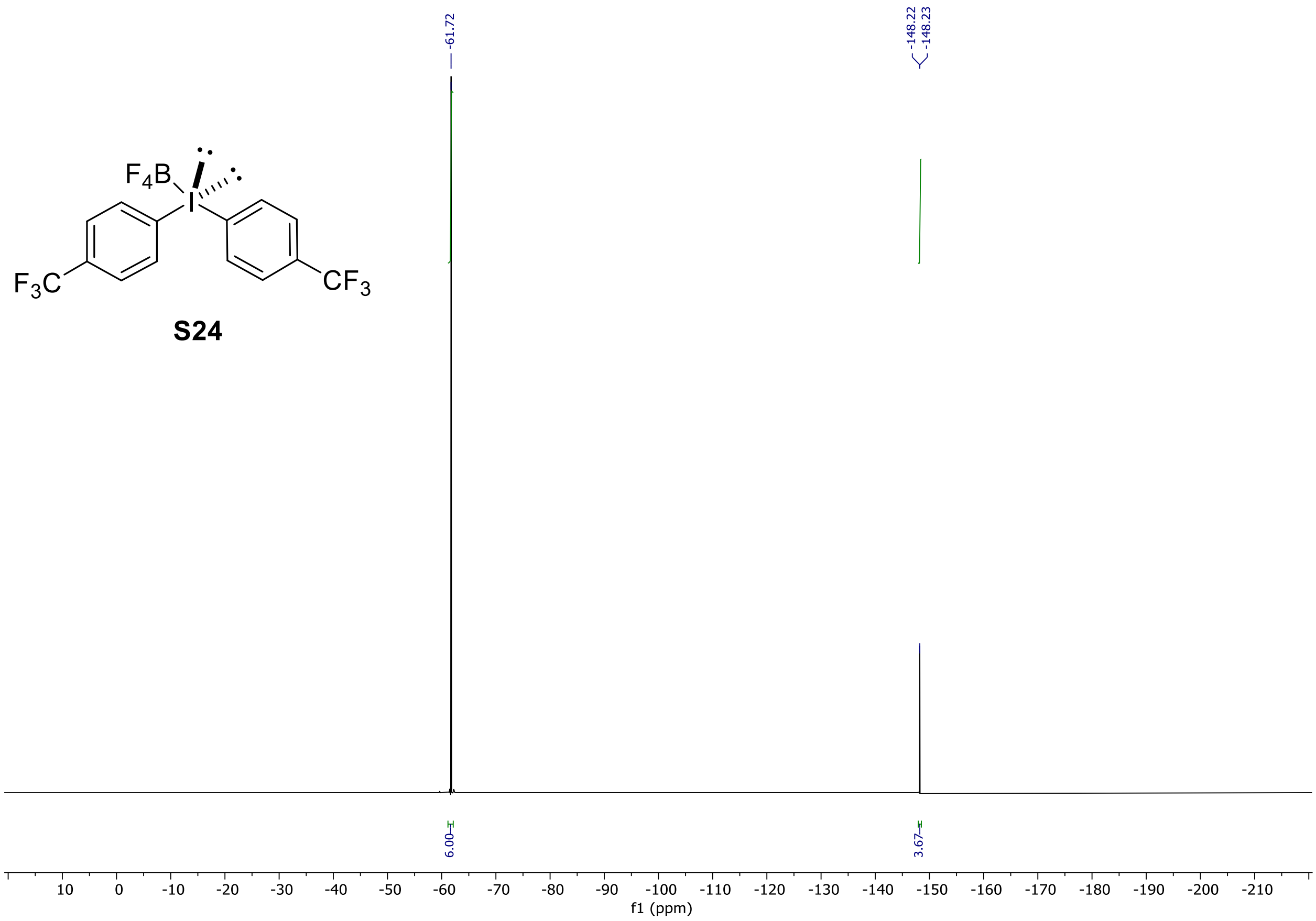

^{1}H NMR (400 MHz, $CDCl_3$) of **S25**

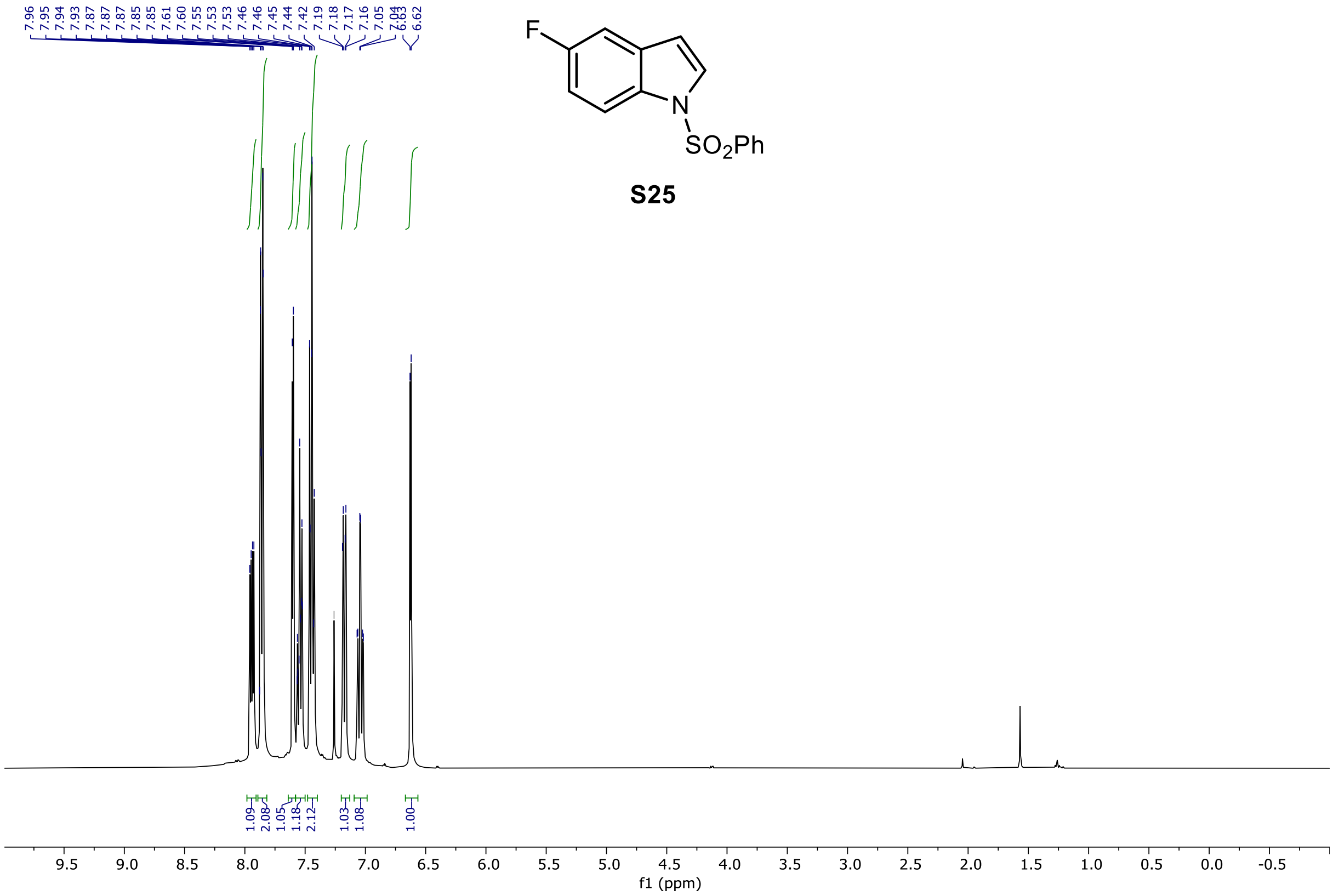

$^{13}C\{^{1}H\}$ NMR (100 MHz, $CDCl_3$) of **S25**

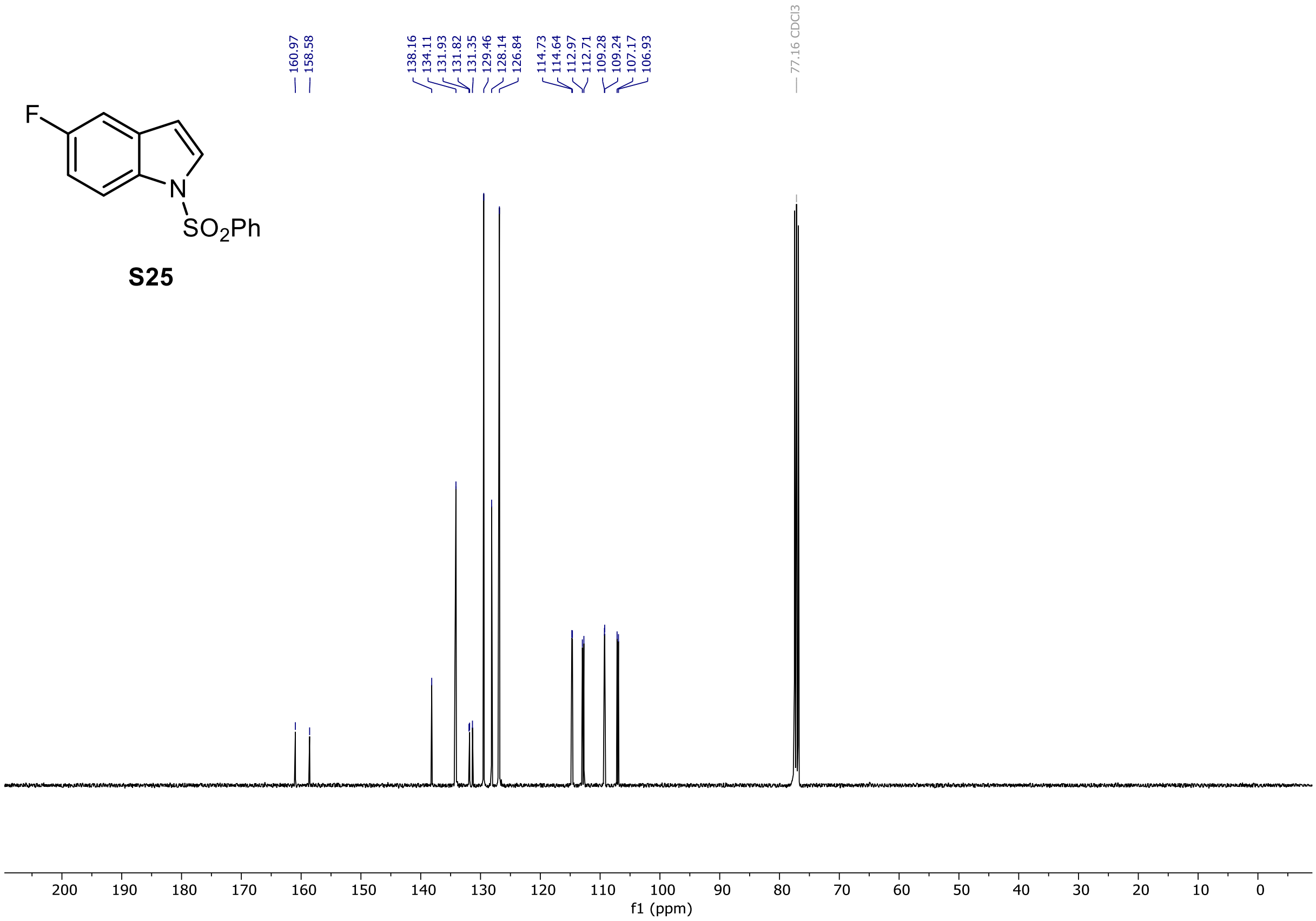

$^{19}F\{^1H\}$ NMR (376 MHz, $CDCl_3$) of **S25**

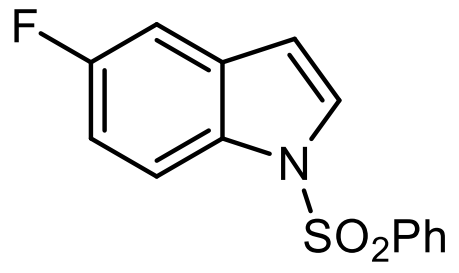

S25

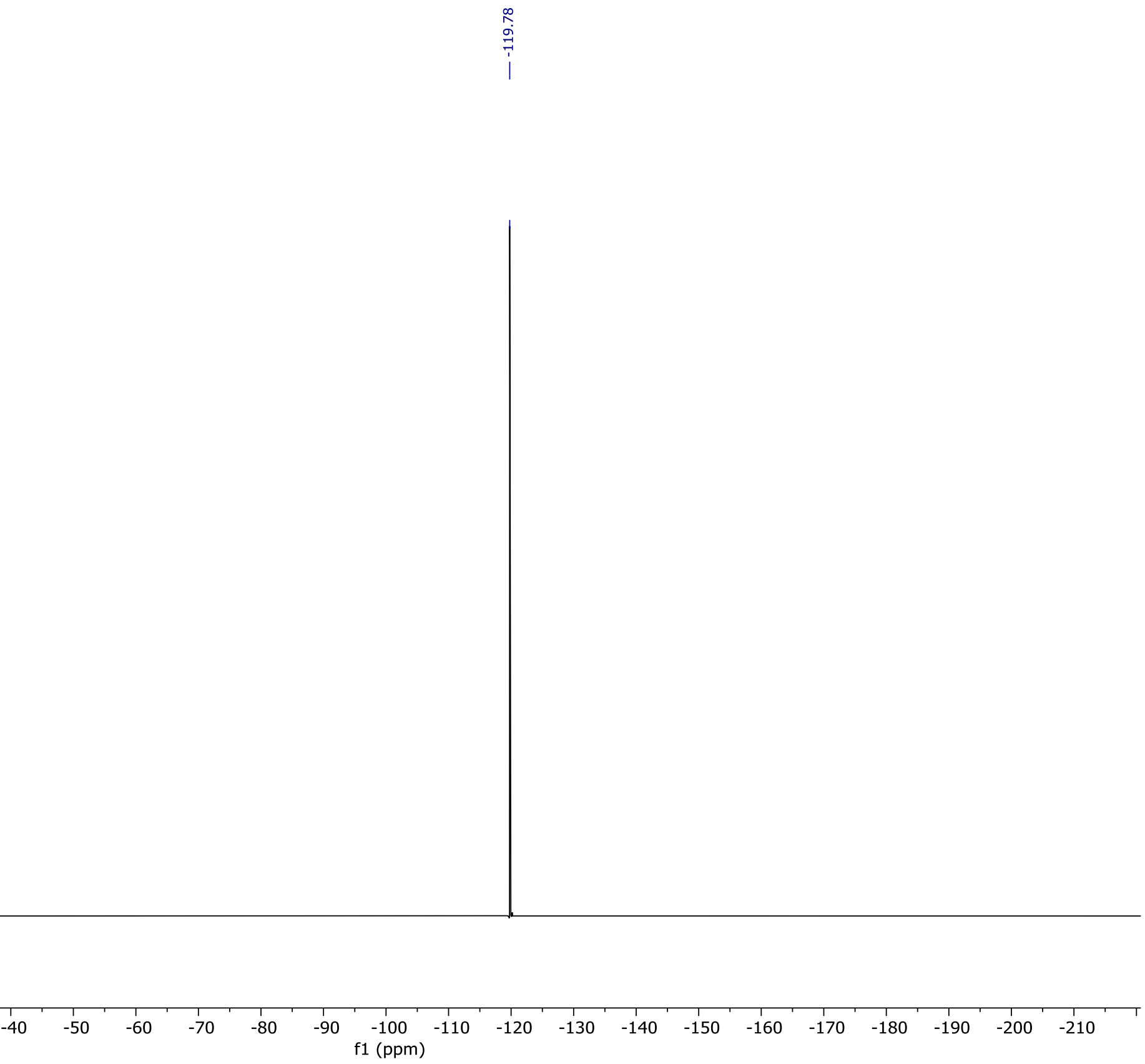

^{1}H NMR (400 MHz, DMSO) of ***d*-S12**

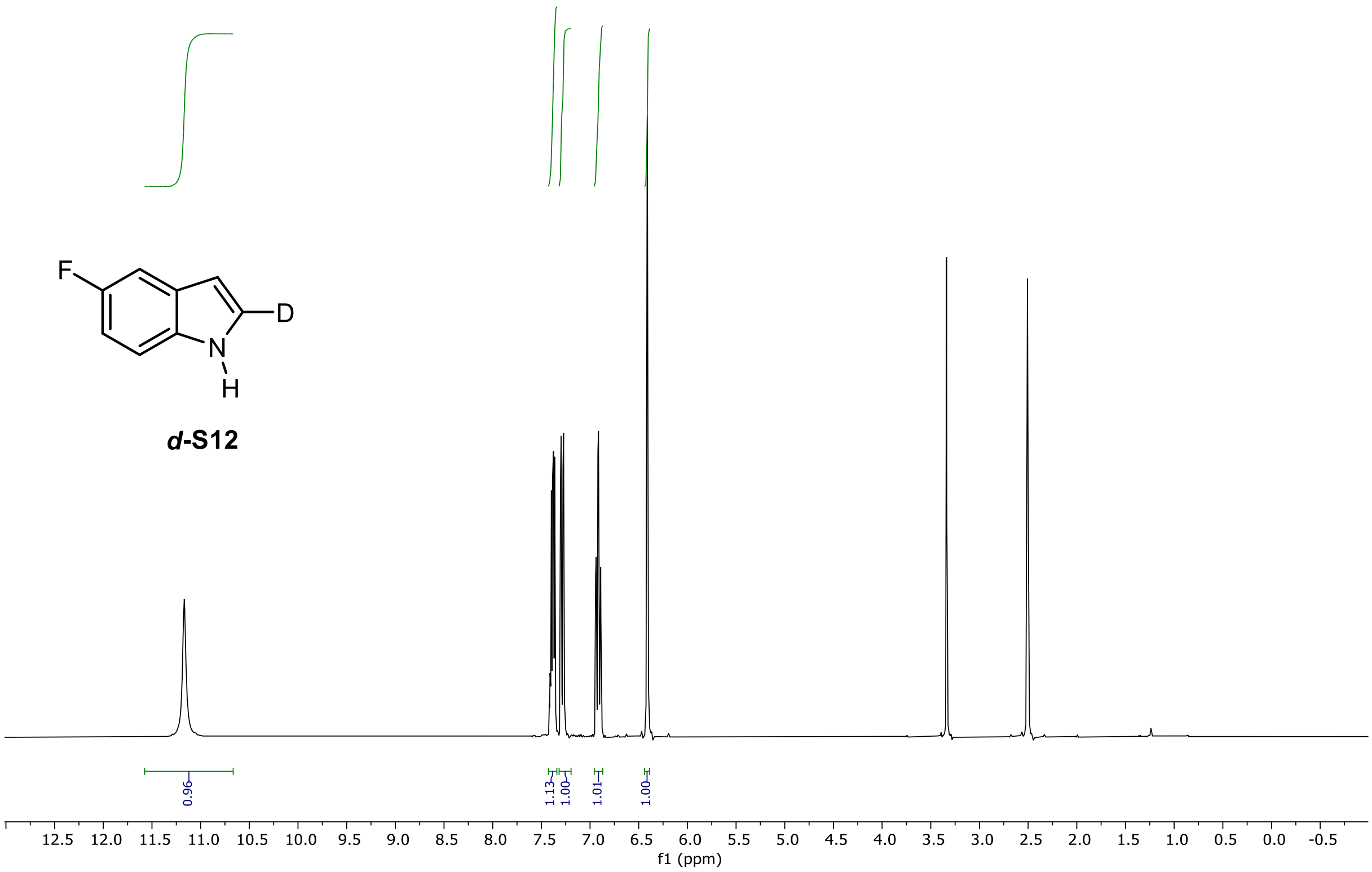

$^{13}C\{^{1}H\}$ NMR (100 MHz, DMSO) of ***d*-S12**

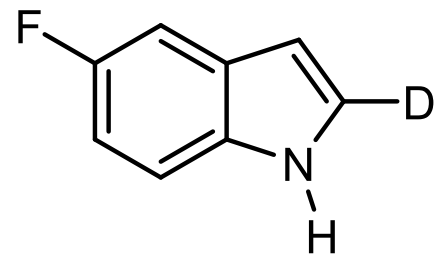

***d*-S12**

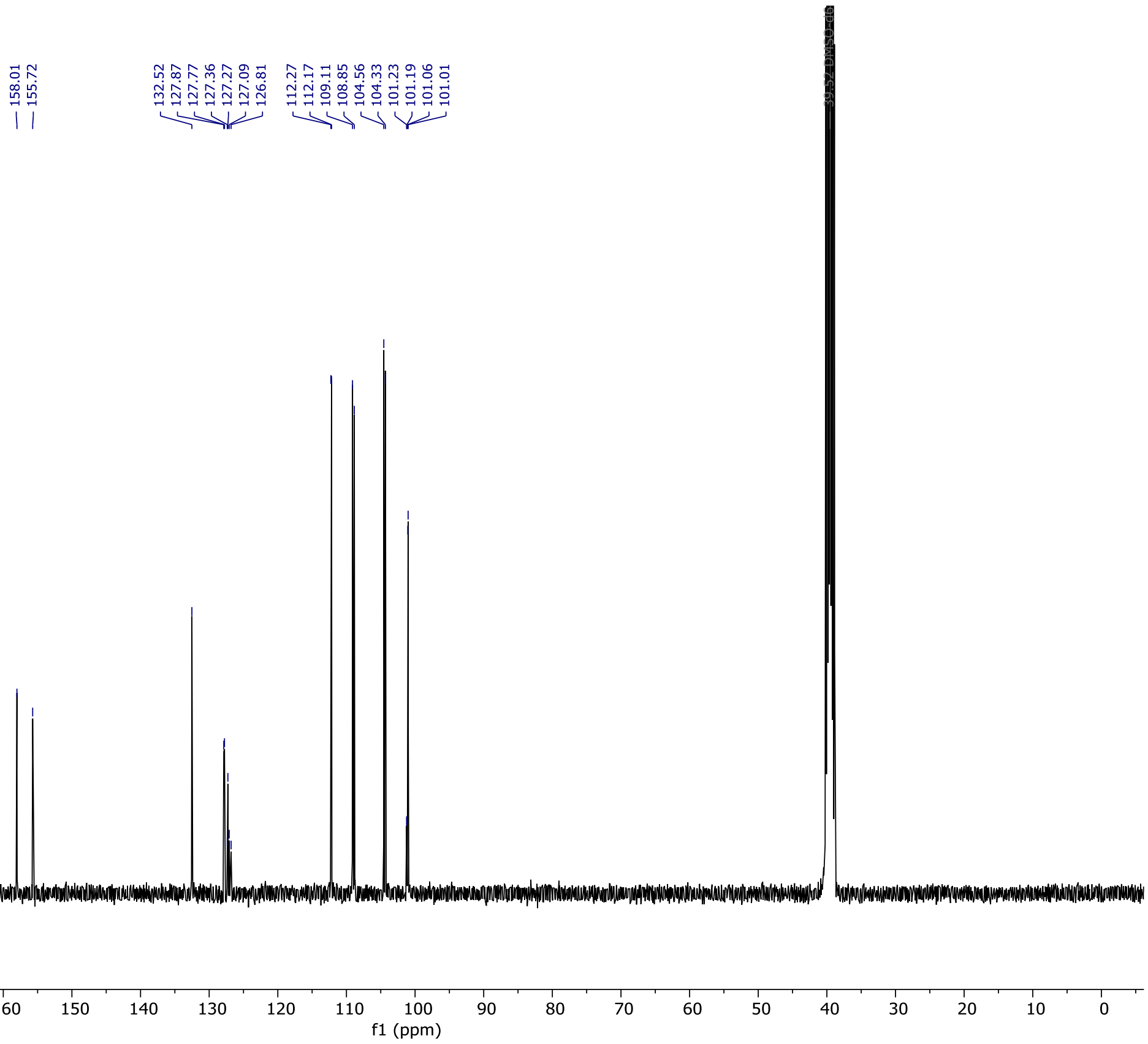

$^{19}F\{^{1}H\}$ NMR (376 MHz, DMSO) of ***d*-S12**

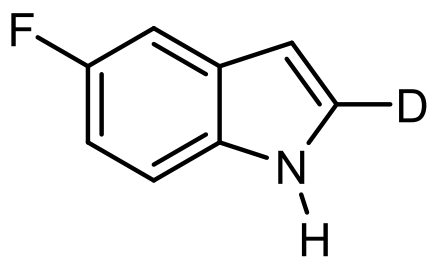

***d*-S12**

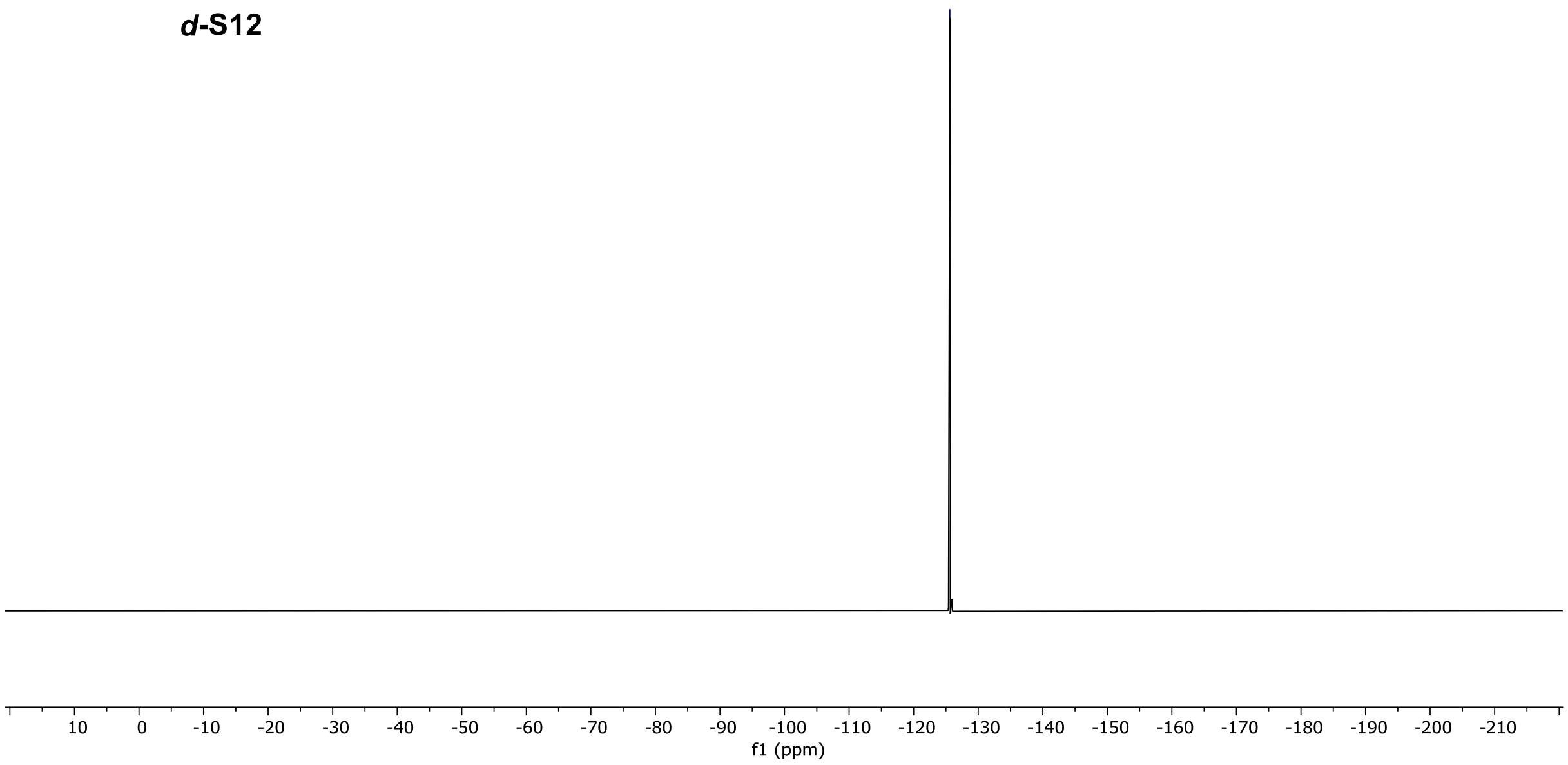

Supplement: Supplementary file 1 [file ja6c11697_si_001.pdf]
